# Catalytic Asymmetric *trans*-Selective Hydrosilylation of Bisalkynes to Access AIE and CPL-Active Silicon-Stereogenic Benzosiloles

**DOI:** 10.1016/j.isci.2020.101268

**Published:** 2020-06-15

**Authors:** Ren-He Tang, Zheng Xu, Yi-Xue Nie, Xu-Qiong Xiao, Ke-Fang Yang, Jia-Le Xie, Bin Guo, Guan-Wu Yin, Xue-Min Yang, Li-Wen Xu

**Affiliations:** 1 Key Laboratory of Organosilicon Chemistry and Material Technology of Ministry of Education, and Key Laboratory of Organosilicon Material Technology of Zhejiang Province, Hangzhou Normal University, Hangzhou 311121, P. R. China; 2 State Key Laboratory for Oxo Synthesis and Selective Oxidation, Suzhou Research Institute (SRI), Lanzhou Institute of Chemical Physics (LICP), University of the Chinese Academy of Sciences (UCAS), Lanzhou 730000, P. R. China

**Keywords:** Chemistry, Organic Synthesis, Stereochemistry

## Abstract

Chirality widely exists in a diverse array of biologically active molecules and life forms, and the catalytic constructions of chiral molecules have triggered a heightened interest in the fields of chemistry and materials and pharmaceutical sciences. However, the synthesis of silicon-stereogenic organosilicon compounds is generally recognized as a much more difficult task than that of carbon-stereogenic centers because of no abundant organosilicon-based chiral sources in nature. Herein, we reported a highly enantioselective rhodium-catalyzed *trans*-selective hydrosilylation of silicon-tethered bisalkynes to access chiral benzosiloles bearing a silicon-stereogenic center. This protocol featured with chiral Ar-BINMOL-Phos bearing hydrogen-bond donors as a privileged P-ligand for catalytic asymmetric hydrosilylation that is operationally simple and has 100% atom-economy with good functional group tolerability as well as high enantioselectivity (up to >99:1 *er*). Benefiting from the *trans*-selective hydrosilylation with the aid of Rh/Ar-BINMOL-Phos-based asymmetric catalysis, the Si-stereogenic benzosiloles exhibited pronounced aggregation-induced emission (AIE) and circularly polarized luminescence (CPL) activity.

## Highlights

• Precise and facile construction of silicon-stereogenic center by desymmetrization
• Highly enantioselective Rh-catalyzed hydrosilylation controlled by MFMC P,O,O-ligand
• Catalytic asymmetric synthesis of AIE- and CPL-active chiral Si-center benzosiloles
• The proposed mechanism is supported by NMR and kinetic studies

## Graphical Abstract

**Figure:**
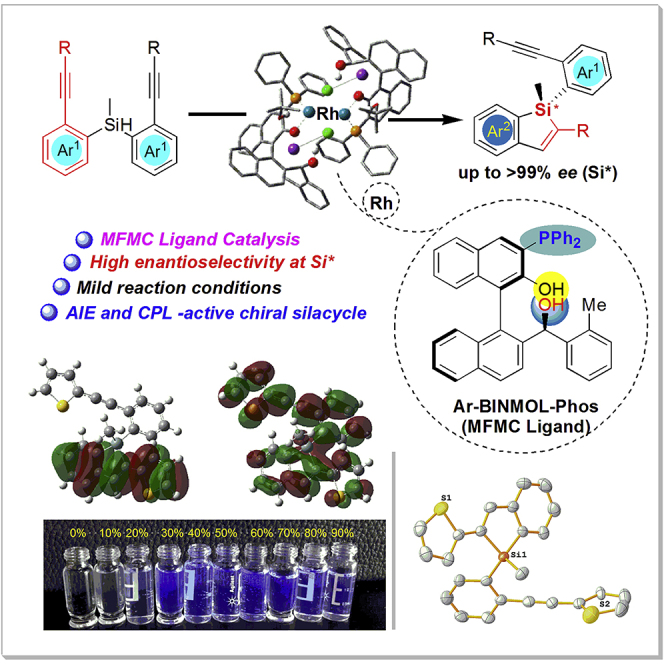

## Introduction

**Scheme 1:**
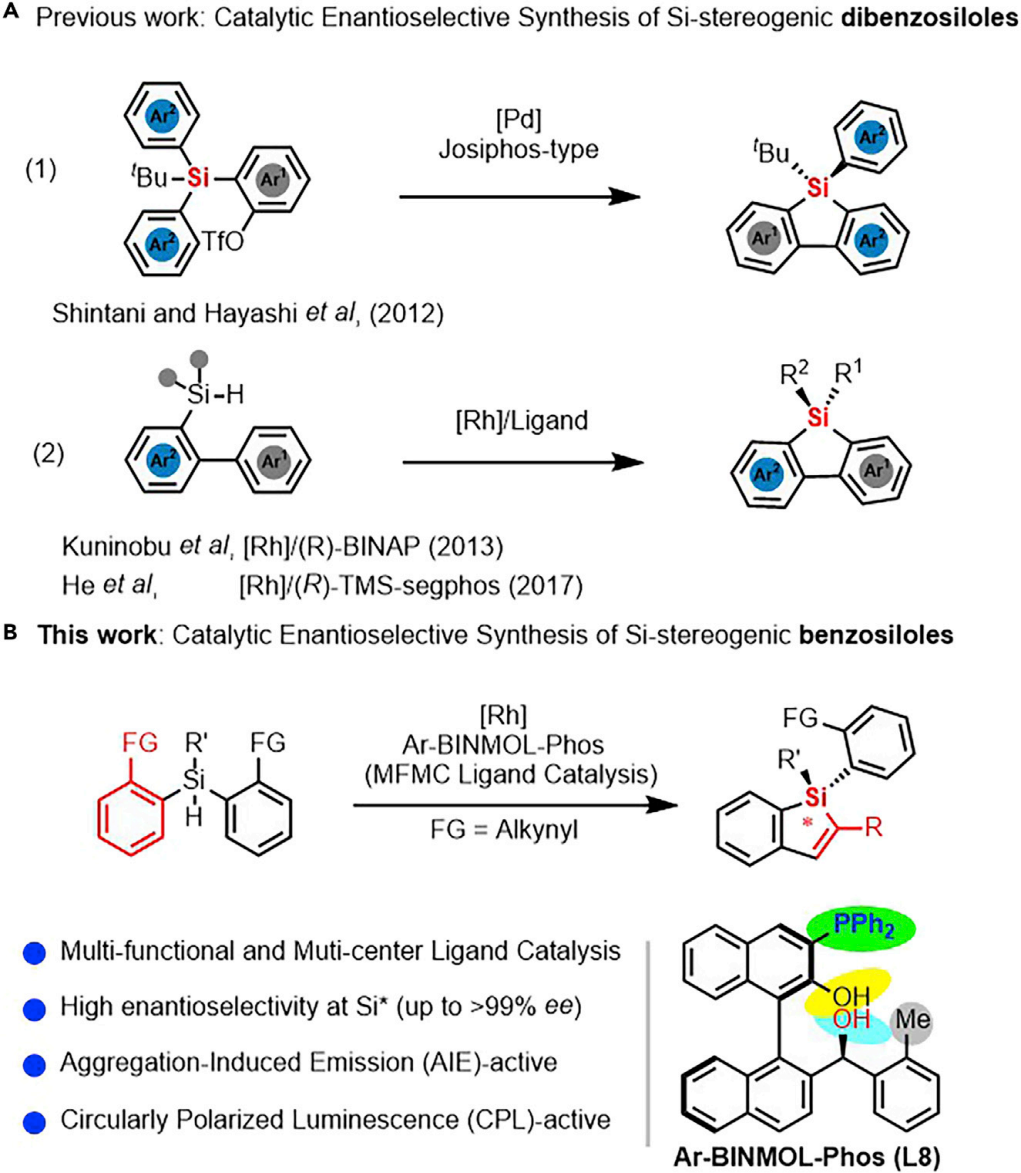
Catalytic Enantioselective Synthesis of Silicon-Stereogenic Silole Analogues (A) Previous reports on the catalytic asymmetric synthesis of silicon-stereogenic dibenzosiloles via C-H activation or silylations. (B) Our strategy with MFMC ligand catalysis via Ar-BINMOL-Phos-controlled Rh-catalyzed intramolecular hydrosilylation to access silicon-stereogenic benzosiloles. MFMC is multi-functional and multi-center.

Silacycles are considered as a new kind of σ∗-π∗ conjugated organic material with low-lying lowest unoccupied molecular orbital (LUMO) energy levels (Chen and Cao, 2007; Fu and Cheng, 2012; Zhao et al., 2015; Pop et al., 2019; Dhbaibi et al., 2019), deriving from the interaction between the σ∗ orbital of two exocyclic silicon-carbon σ-bonds and the π∗ orbital of the butadiene moiety (Yamaguchi and Tamao, 1996). As one of the most important types of silacycles, siloles exhibit unique electronic structure with low-lying LUMO level with intriguing optical and electronic properties due to high electron affinity and fast electron mobility, enabling them to function as luminescent core and electron transporters in optoelectronic devices (Tamao et al., 1996a, 1996b; Uchida et al., 2001, Son et al., 2009), such as organic light-emitting diodes (Chen et al., 2002; Cai et al., 2017 Nie et al., 2018), fluorescent bioprobes (Wu et al., 2010; Zhuang et al., 2017), chemosensors (Toal et al., 2005; Li et al., 2009; Dedeoglu et al., 2014), and circular polarized luminescence (CPL) (Liu et al., 2012; Ng et al., 2014a and 2014b; Li et al., 2016). Therefore, the development of practical methods for the synthesis of silole scaffolds is highly important in both synthetic and materials chemistry, which has attracted increasing attention (Ohmura et al., 2008; Matsuda et al., 2007; Ureshino et al., 2010; Liang et al., 2011; Shimizu et al., 2008; Zhao et al., 2012; Liang et al., 2012; Zhang et al., 2014 and 2015; Ilies et al., 2008; Tobisu et al., 2009; Onoe et al., 2012; Minami et al., 2017; Gimferrer et al., 2018; Yang et al., 2018). Especially, the construction of a stereogenic Si-center of siloles in a catalytic enantioselective manner is an appealing yet challenging task, although there have been some efforts for the catalytic synthesis of chiral Si-centers (Oestreich, 2007; Weickgenannt et al., 2010; Xu et al., 2011; Xu, 2012; Shintani, 2015; Tamao et al., 1996a, 1996b; Igawa et al., 2012; Naganawa et al., 2015; Zhan et al., 2018; Wen et al., 2018; Guo et al., 2019). In this regard, the catalytic enantioselective synthesis of silicon-stereogenic heterocycles have been achieved via Pd-catalyzed C-H arylation (Shintani et al., 2012) or amination (Sato et al., 2017) of prochiral 2-(arylsilyl)aryl triflates or Rh-catalyzed aromatic C-H silylation (Scheme 1A) (Kuninobu et al., 2013; Zhang et al., 2016, 2017). Moreover, the rhodium-catalyzed [2 + 2 + 2] cycloaddition of silicon-containing prochiral triynes with internal alkynes was also a facile approach to the construction of silicon-stereogenic DBS (Shintani et al., 2015). All these methods mentioned above provided complementary processes to the preparation of enantio-enriched chiral DBS bearing a silicon-stereogenic center. However, the construction of Si-chirality on unsymmetrical benzosiloles (BS) via catalytic hydrosilylation is still unknown to date.

Very recently, we reported the catalytic asymmetric synthesis of sila-bicyclo[4.1.0]heptanes via palladium-catalyzed [4 + 2] annulation of cyclopropenes with benzosilacyclobutanes (Wang et al., 2020), in which a variety of chiral bicyclic sila-heterocycle derivatives could be achieved with good enantioselectivity (up to 95.5:4.5 *er*). However, the construction of corresponding silacycles bearing a Si-stereogenic center is not successful in this reaction. Considering the powerful potential of rhodium catalysts for hydrosilylation of alkynes that can provide *E*- and *Z*-isomers depending on the precise nature of catalyst systems, substrates, and reaction conditions (Takeuchi and Tanouchi, 1993, 1994; Faller and D'Alliessi, 2002; Sato et al., 2004; Mori et al., 2004; Huckaba et al., 2013; Mancano et al., 2014; Diachenko et al., 2015), we envisioned that the Rh-catalyzed intramolecular and *trans*-type hydrosilylation might open the door to the enantioselective Si-C bond-forming construction of silicon-stereogenic benzosiloles. Although the desymmetrizative hydrosilylation of alkenes has been achieved to construct silicon-stereogenic center (Naganawa et al., 2015), this reaction suffered limited substrate scope and imperfect enantioselectivity. Comparably, the intramolecular hydrosilylation of Si-tethered bisalkynes is much challenging. This lack of available *trans*-selective methods with high enantioselectivity is presumably due to the difficulty in the chirality induction during desymmetrization of dihydrosilanes (R^1^R^2^SiH_2_) or silicon-tethered bulky bisalkynes in catalytic asymmetric hydrosilylation. Herein, we reported such a *trans*-selective intramolecular hydrosilylation reaction with our strategy with Rh/Ar-BINMOL-Phos-based MFMC ligand catalysis (MFMC is multi-functional and multi-center) that can generate silicon-stereogenic siloles with good enantioselectivity (Scheme 1B). This P,O,O-ligand (Ar-BINMOL-Phos) -controlled approach affords a range of alkyne-substituted silicon-stereogenic siloles as potentially useful fluorescent probes from easily available alkynes and hydrosilanes. In addition, it should be noted that the extra alkynyl group on benzosiloles offered a potential functionalization with group transformations.

## Results

**Figure 1:**
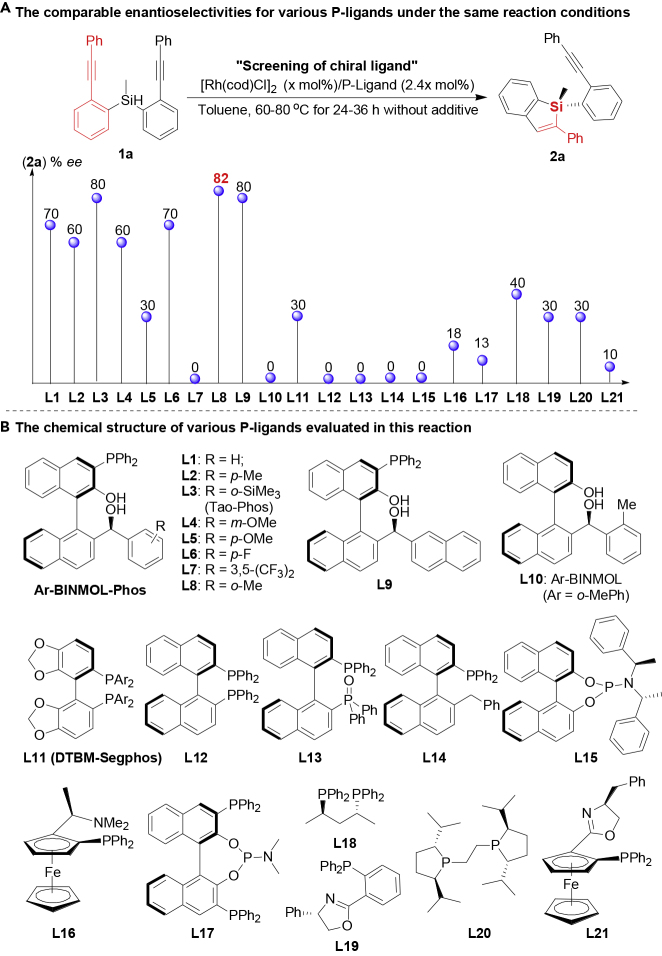
Representative Results on Enantioselectivity for Chiral Ligand-Controlled [Rh(cod)Cl]_2_-Catalyzed Intramolecular Hydrosilylation (A) The comparable enantioselectivities for various P-ligands (**L1-L21**) under the same reaction conditions (without any additive and no optimization of reaction conditions). (B) The chemical structure of various P-ligands (**L1-L21**) evaluated in this reaction.

To construct the silicon-stereogenic BS via catalytic asymmetric hydrosilylation, we are confident that the Rh-catalyzed intramolecular and *trans*-type hydrosilylation would provide a robust and practical method to the synthesis of this type of alkynyl benzosiloles. Then we began our studies by examining the model hydrosilylation of Si-tethered bisalkyne **1a** using Rh catalysts. At 80°C in toluene, various phosphine ligands were evaluated in the [Rh(cod)Cl]_2_-catalyzed intramolecular hydrosilylation (see Figure 1 and Table S1). Gratifyingly, most of our MFMC Ar-BINMOL-Phos (Song et al., 2014) gave silicon-stereogenic benzosilole **2a** in promising conversion (up to >99%) with moderate enantioselectivity (80:20 to 91:9 *er* in most cases) in the absence of any additives, demonstrating the good feasibility of this Rh-catalyzed hydrosilylation reaction with the aid of chiral P,O,O-ligand; especially, our Tao-Phos (**L3**) and methyl-substituted Ar-BINMOL-Phos (**L8**) (Song et al., 2015) gave the desired product **2a** with 89.5:10.5 *er* and 91:9 *er*, respectively. Notably, other phosphine ligands evaluated in this work, such as BINAP and Segphos, exhibited relatively low enantioselectivity (70:30 er as the best). Then with Tao-Phos in hand, we continued to optimize the reactions by changing rhodium precursors, additives, solvents, and temperature (for representative experimental data, see Tables S2–S4). And finally, the optimized reaction conditions were determined as follows (see Table 1, entry 1): [Rh(cod)Cl]_2_ (5 mol%), Ar-BINMOL-Phos (*o*-Me) (12 mol%, simplified as **L8** in Supplemental Information), KO*t*Bu (5 mol%), at 70°C. The corresponding product **2a** could be obtained in 95.5:4.5 *er*, albeit the decrease of isolated yield because of low and almost same polarity in comparison with that of starting material **1a**. As shown in Table 1, representative reaction results were also important to understand the challenging Rh-catalyzed intramolecular hydrosilylation. The effect of temperature (70°C or 80°C) on enantioselectivity is not obvious as imagined (entry 2). Under the optimized reaction conditions, other rhodium sources did not give better results in term of conversion and enantioselectivity (entry 3). To support the importance of three functional groups (P atom, chiral secondary alcohol, and phenol moiety) on the MFMC P,O,O-ligand (Ar-BINMOL-Phos), we investigated the effect of four representative ligands (**CL1**-**CL4**) on the conversion and enantioselectivity (entries 5–9). In sharp contrast, these P-ligands without additional chirality at the carbon of secondary alcohol (**CL1** and **CL4**) gave inferior enantioselectivity (88:12 *er* for **CL1** and 67:33 *er* for **CL4**, respectively, entries 6, 9). And more importantly, ligands **CL2** and **CL4** without secondary alcohol or phenol were not effective in the Rh-catalyzed hydrosilylation because of only moderate conversion (entries 7 and 9). Replacing two OHs (both secondary alcohol and phenol) with MOM and OBn, respectively, deactivated the catalyst (entry 8), probably because of strong hydrogen-bonding interaction between Rh/ligand and substrate. It is easy to understand that Ar-BINMOL without phosphine center is not suitable ligand, which supports the importance of P-atom in the coordination with Rh catalyst. In addition, KO*t*Bu was proved to be an effective additive to promote the intramolecular hydrosilylation. As shown in Table 1 (entries 10–16), the use of other additives, including similar inorganic bases, resulted in inferior results in terms of conversion and enantioselectivity. In fact, we have also investigated the effect of the amount of KO*t*Bu on the Rh-catalyzed intramolecular hydrosilylation of **1a** (Table S5). The experimental results showed that the hydrogen-bonding activation from free chiral secondary alcohol is beneficial to the activation of Rh catalyst because large amount of KO*t*Bu could inhibit the hydrosilylation. To our delight, when cobalt and palladium catalysts instead of rhodium catalyst were used in this reaction under the same conditions, only a trace amount of product **2a** was detected, and poor enantioselectivity was observed in these experiments (entries 17–19).

**Table 1:** Optimization of Reaction Conditions for Rh-catalyzed Desymmetric Hydrosilylation of Bisalkyne **1a**


Entry	Variation from Standard Conditions^a^	T (°C/h)	2a/1a^b^	*er*^c^
1	None	70/72	>99:1 (50)	95.5:4.5
2	None	80/34	>99:1	94.5:5.5
3	[Rh(cod)_2_]BF_4_ instead of [Rh(cod)Cl]_2_	80/34	n.r	–
4	[Rh(OAc)_2_]_2_ instead of [Rh(cod)Cl]_2_	80/34	n.r	–
5	**Tao-Phos** instead of **L8**	80/22	80:20	92.5:7.5
6	**CL-1** instead of **L8**	70/72	>99:1	88:12
7	**CL-2** instead of **L8**	70/72	54:46	60:40
8	**CL-3** instead of **L8**	70/72	n.r	–
9	**CL-4** instead of **L8**	70/72	34:66	67:33
10	NaHBEt_3_ instead of KO*t*Bu	80/34	98:2	92.5:7.5
11	NaO*t*Bu instead of KO*t*Bu	80/34	>99:1	90:10
12	NaSbF_6_ instead of KO*t*Bu	80/34	n.r	–
13	CuI instead of KO*t*Bu	80/34	46:54	90:10
14	Ag_3_PO_4_ instead of KO*t*Bu	80/34	90:10	92.5:7.5
15	K_2_CO_3_ instead of KO*t*Bu	80/34	58:42	90.5:9.5
16	NaOEt instead of KO*t*Bu	80/34	98:2	53.5:46.5
17	OIP Co instead of [Rh]^d^	80/14	10:90	50:50
18	Pd_2_(dba)_3_	80/14	10:90	62.5:37.5
19	(η_3_-C_3_H_5_)_2_Pd_2_Cl_2_	80/14	n.r	–


a Unless otherwise noted, the standard reaction conditions were as follows: **1a** (0.2 mmol), and solvent (2.0 mL). The structure of Tao-Phos with *o*-trimethylsilyl group is different from that of *o*-methyl substituent on phenyl ring (**L8**), the conversion is >99% for a family of Ar-BINMOL-Phos.

b It was difficult to isolate the product **2a** from the reaction mixture if the reaction was not completed because of the same polarity of **2a** and the starting material **1a**. The ratio of **2a/1a** was determined by HPLC. n.r = no reaction.

c The er value of **3a** was determined by chiral HPLC analysis.

d The catalyst OIP Co complex was used instead of [Rh(cod)Cl]_2_/Ar-BINMOL-Phos catalyst system.

**Scheme 2:**
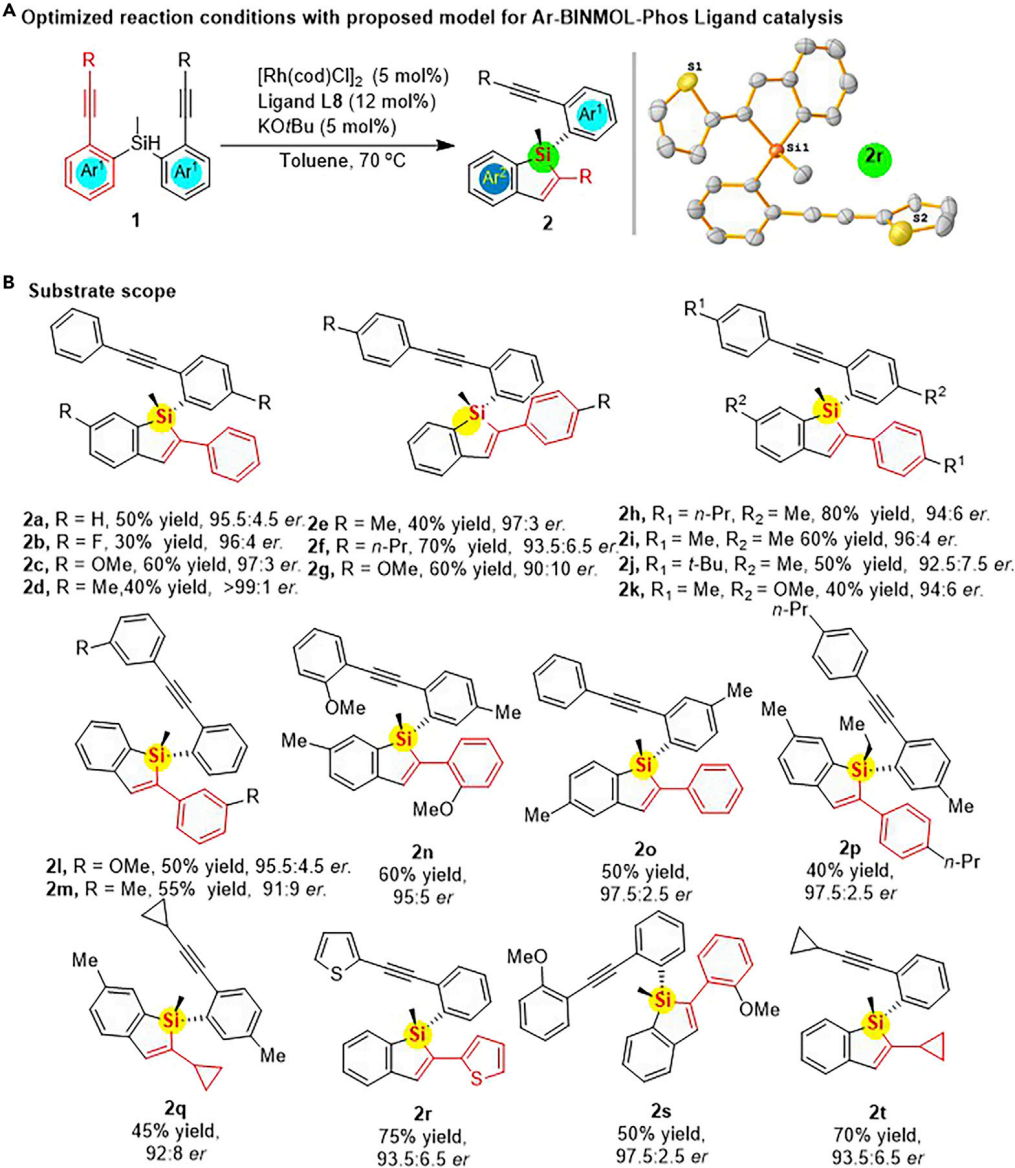
Scope of MFMC Ar-BINMOL-Phos (L8)-controlled Rh-catalyzed Intramolecular Hydrosilylation of Si-Tethered Bisalkynes (A) The optimized reaction conditions for the Ar-BINMOL-Phos-based MFMC ligand catalysis based on the experimental results. (B) Substrate scope for the *trans*-selective hydrosilylation driven by the MFMC Ar-BINMOL-Phos ligand-controlled desymmetrization of bisalkynes.

With the optimized reaction conditions in hand, the substrate scope was next explored with respect to the variation of the silicon-tethered bisalkynes (Scheme 2). Si-linked bisalkynes with varied substitution patterns (Me, OMe, F, *i*-Pr, or *t*-Bu, etc.) could be smoothly converted to their corresponding hydrosilylation products in moderate to good yields (up to 87%) with high enantioselectivities (up to >99:1 *er*). Notably, this potassium-assisted Rh-catalyzed hydrosilylation reaction worked well with various types of substrates with electron-neutral, electron-withdrawing or electron-donating groups, having little influence on the Si-centered stereochemistry. When small ring or S-containing heterocycle-substituted bisalkynes were employed, the reaction also worked well under Rh/Ar-BINMOL-Phos catalyst system. For example, the hydrosilylation of **1r** proved to be highly enantioselective (93.5:6.5 *er*) with good yield (75%), and the cyclopropanyl group linked with the terminal position of alkyne on substrate **1q** or **1t** resulted in the corresponding alkynyl benzosilole **2q** or **2t** in good yield with high er value, respectively. In addition, the *ortho*-substituted group did not block the intramolecular hydrosilylation, as evidenced by the reaction of **1n** to **2n** with 60% yield and 95:5 *er*. The same level of experimental result was also provided by the intramolecular hydrosilylation reaction of Si-linked bisalkyne **1s**, giving the corresponding **2s** with 50% yield and 97.5:2.5 *er*. When the methyl group on silicon atom was replaced by ethyl group, the reaction was also proven to be enantioselective and gave the desired benzosilole **2p** in 97.5:2.5 *er*, albeit with some yield loss in comparison with that of methyl-substituted **2h** (80% yield, 94:6 *er*). Unfortunately, it was found that another bulky group on silicon center, such as *t*-Bu and phenyl, was not suitable for the construction of their corresponding benzosiloles because of the steric hindrance. Notably, the configuration of the silicon-stereogenic alkynyl benzosiloles (**2r**) was confirmed by X-ray diffraction pattern (Figure S1). Notably, these compounds were very stable for a long period of time (0.5 year), and its ee value was not changed after reflux in toluene for several days.

**Figure 2:**
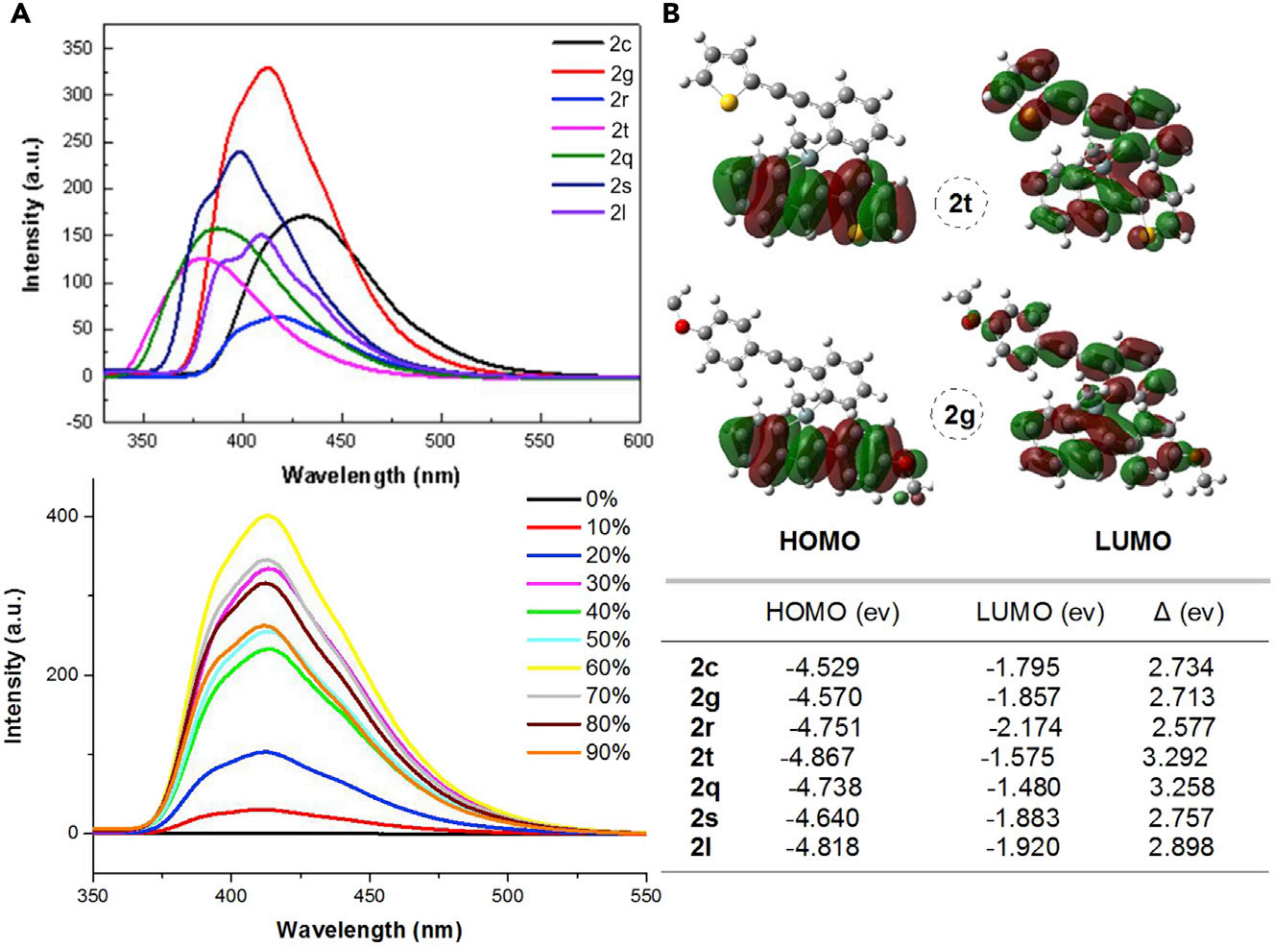
The Fluorescence Property of Silicon-Stereogenic Benzosiloles (A) Left: Fluorescence spectra (top) of seven representative and enantioenriched benzosiloles and AIE phenomenon of benzosilole **2g**, the fluorescence emission spectra of 2g (5 μM) was achieved in THF/water mixtures (fw = 0% to 90%). λ_ex_ = 300 nm, λ_es_ = 550 nm. (B) Right: Molecular orbital diagrams of HOMO and LUMO of **2t** and **2g**, and the energy levels of HOMO and LUMO with their difference (ΔE) of representative benzosiloles shown in this table.

Then, the further investigation of such alkynyl benzosiloles as optical material was highly attractive. The fluorescence property of benzosiloles (BS) was subsequently evaluated using **2c, 2g, 2r, 2t, 2q, 2s, and 2l** as candidates. As shown in Figure 2, the BS emission was enhanced by the introduction of electron-donating group (-OMe) on the aromatic ring, and *p*-OMe substituted BS **2g** exhibited a very distinctive behavior. The highest occupied molecular orbital (HOMO)-LUMO data might be useful information to distinguish the structural difference. Thus, we then checked the aggregation-induced emission (AIE) property of **2g** according to the standard method (Zhou et al., 2019). As expected, the benzosilole **2g** showed a blue fluorescence color when the water fraction was above 30% in the THF-water mixture (Figure S7 of Supporting Information). Notably, the relationship of AIE and chirality was also evaluated and it was found that there is no obvious effect for the fluorescence intense.

The phenomenon of circularly polarized luminescence (CPL) has attracted considerable attention owing to its wide applications in various research fields (Gao et al., 2019). Therefore, the circular dichroism (CD) and CPL analyses were next performed at 300 nm to evaluate the Si-centered chirality. To our delight, in the test of benzosilole **2g**, intensive CPL signs were observed in this case (Figure S8). We anticipated that CPL effect of benzosilole augments its great potential of enantioselective Rh-catalyzed intramolecular hydrosilylation of bisalkynes in the development of a CPL-active material linked with silole backbone.

## Discussion

**Figure 3:**
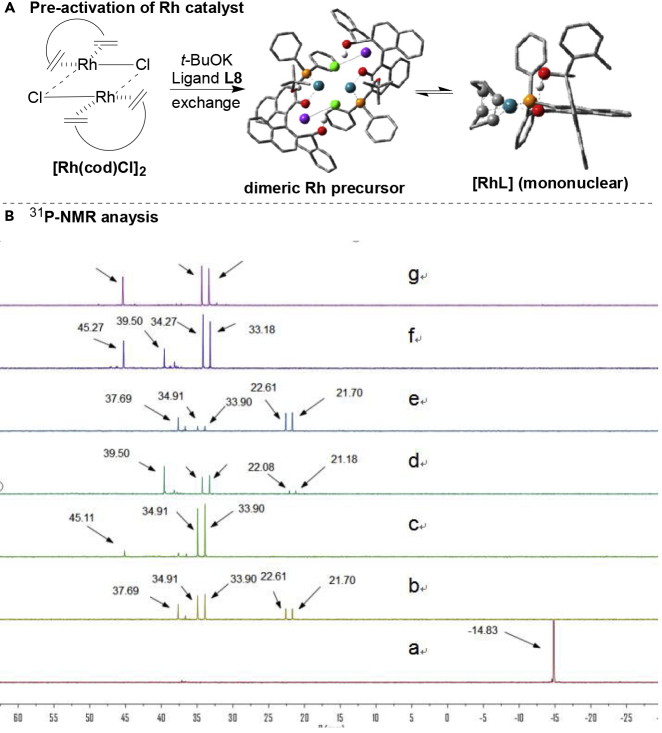
The Structural Analysis of the Active Rh Species by ^31^P NMR (A) The illustrative view of the *in situ* formed dimeric Rh complex and mononuclear Rh complex from [Rh(cod)Cl]_2_ and Ar-BINMOL-Phos L8 in CDCl_3_. (B) Comparison of ^31^P NMR of ligand and Rh complex. (a) only **L8**, a single peak at −14.83 ppm; (b) mixture of [Rh(cod)Cl]_2_ and **L8** (5 min), two double peaks for the Rh/Ar-BINMOL-Phos complex appeared at 22.16 ppm with ^1^*J*_P-Rh_ = 183 Hz and 34.40 ppm with ^1^*J*_P-Rh_ = 204 Hz, and another single peak appeared at 37.69 ppm, respectively; (c) mixture of **L8** and [Rh(cod)Cl]_2_ (20 min), the double peak at 22.16 ppm disappeared, and another single peak appeared at 45.11 ppm; (d) mixture of substrate **1a**, **L8** and [Rh(cod)Cl]_2_ (20 min), a new and single peak appeared at 39.50 ppm; (e) mixture of **L8**, KO*t*Bu, and [Rh(cod)Cl]_2_ (5 min); (f) mixture of **L8**, KO*t*Bu, and [Rh(cod)Cl]_2_ (20 min); (g) mixture of **L8**, KO*t*Bu, [Rh(cod)Cl]_2_, and substrate **1a** (20 min), a double peak appeared at 33.72 ppm with ^1^*J*_P-Rh_ = 220 Hz and a single peak with 45.27 ppm.

**Figure 4:**
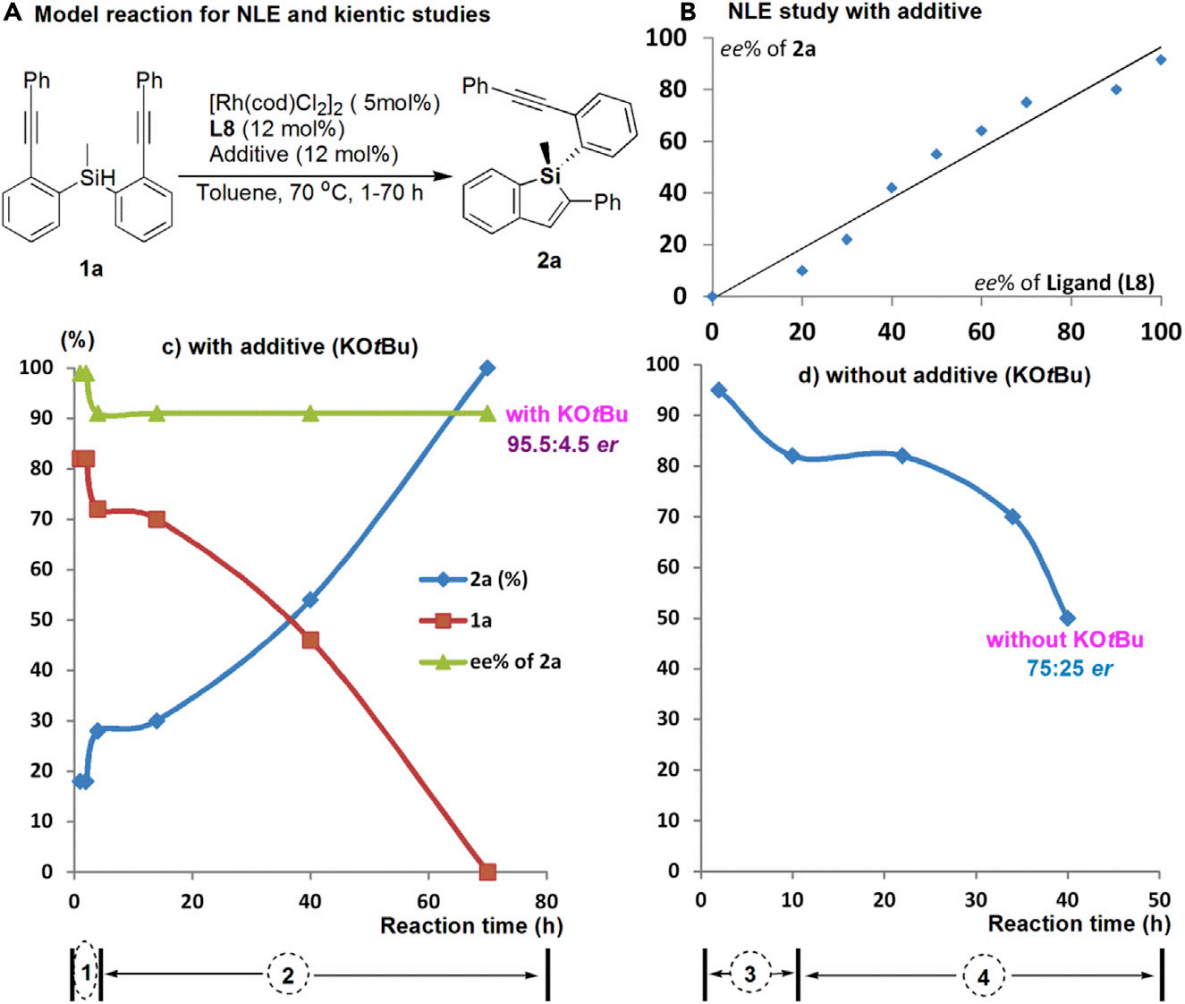
Experimental Results for Relationship of ee_prod_/ee_ligand_ and the Reaction Rate and Enantioselectivity Data with or without KO*t*Bu (A) The model reaction with intramolecular hydrosilylation of 1a under the optimized reaction conditions. (B) The NLE result revealed that a mononuclear complex structure with single Rh(I) catalytic center with one ligand acted probably as the true active species. (C) KO*t*Bu-activated Rh catalysis. There are two stages (before and after 5 h, respectively) for catalytic cycles in the Rh-catalyzed hydrosilylation that detected by *ee* values and reaction rate under the optimized reaction conditions. (D) Without KO*t*Bu as additive. When no use of KO*t*Bu for this reaction, the corresponding ee value gradually decreases with reaction time.

In metal-catalyzed hydrosilylation (Zaranek and Pawluc, 2018; Wen et al., 2019), including the Rh catalysts (Sakaki et al., 2002; Wu et al., 2013 and 2014; Doyle et al., 1991; Sanada et al., 2006; Morales-Ceron et al., 2017) employed for alkyne hydrosilylation, cationic metal complexes usually give predominately (*E*)-isomer depending on the precise nature of substrates and reaction conditions (Ojima et al., 1990; Trost and Ball, 2005; Ding et al., 2013). In this work, we believed the Rh-catalyzed alkyne *trans*-hydrosilylation reactions are similar to that of previous reports (Ojima et al., 1990; Matsuda and Ichioka, 2012; Crabtree, 2003) on the isomerization of the M-vinyl complex intermediate to the less sterically congested isomer via an η^2^-vinyl metal species. In addition, to understand what makes Rh/Ar-BINMOL-Phos (**L8**) a successful catalyst in the desymmetrization of Si-linked bisalkynes via hydrosilylation, its structure was examined by ^31^P-NMR and ESI-MS (see Figure 3, and for ^31^P-NMR analysis, see Figure 3B, and others see Figures S3–S5). And these experimental results indicate that the various types of Rh/**L8** complexes might be *in situ* formed in this reaction, and a dimeric Rh catalyst comprising a dirhodium core is a possible and active species in the pre-activation process (Meiβner et al., 2015a, 2015b; Mannu et al., 2018), which generated through dissociation of cod (1,5-cyclooctadiene) with two coordinating phosphorous centers from Ar-BINMOL-Phos ligands. It is generally accepted that treatment of [Rh(cod)Cl]_2_ with diphosphine ligands smoothly affords neutral μ_2_-bridged dimeric/dinuclear rhodium complexes (Meiβner et al., 2015a, 2015b); thus, accordingly, the μ_2_-bridged dimeric Rh complex is formed probably in the reaction mixtures (the double peaks appeared probably at 33–34 ppm with ^1^*J*_P-Rh_ = 204 or 220 Hz in Figure 3B). However, it is difficult to confirm the true structure of dimeric Rh/**L8** complex that formed in the pre-activation stage by NMR analysis. Furthermore, the *in situ* analysis of reaction mixtures with ESI-MS and NLE (Satyanarayana et al., 2009) and kinetic study (Figure 4) showed the mononuclear complex with single Rh(I) catalytic center with one ligand acted probably as majorly active species during the full reaction process. In addition, the stable P/O-coordination of Ar-BINMOL-Phos with [Rh(cod)Cl]_2_ to give mononuclear Rh complex is supported by ^31^P-NMR spectra data in which the related ^31^P signal of such stable mononuclear Rh complex was observed at 41 ppm (Rani et al., 2008).

It should be noted that the potassium *tert*-butoxide (KO*t*Bu) played an important role in the *in situ* formation of the active Rh species to promote the catalytic asymmetric hydrosilylation. As shown in Figure 4, the absence of KO*t*Bu led to decreased enantioselectivity (only 75:25 er), in which the negative result revealed reaction between KO*t*Bu and chiral ligand **L8** could form a more stable mononuclear rhodium catalyst that is responsible for the high level of enantioselective induction shown in Figure 4C. When the reaction was performed without KO*t*Bu, enantioselectivity (ee value of **2a**) of the same intramolecular hydrosilylation was gradually decreased with time because of irreversible reactions of the active dimeric Rh catalyst with substrate in the catalytic cycle. And notably, excess amount of KO*t*Bu (>24 mol%) decreased the enantioselectivity and much more amount of KO*t*Bu (>36 mol%) inhibited the catalytic activities of all the Rh species reaction to result in almost no reaction. These results provided an indirect evidence for the importance of the chiral secondary alcohol of Ar-BINMOL-Phos (**L8**) in the enhancement of enantioselectivity and catalytic activity of Rh complex.

**Figure 5:**
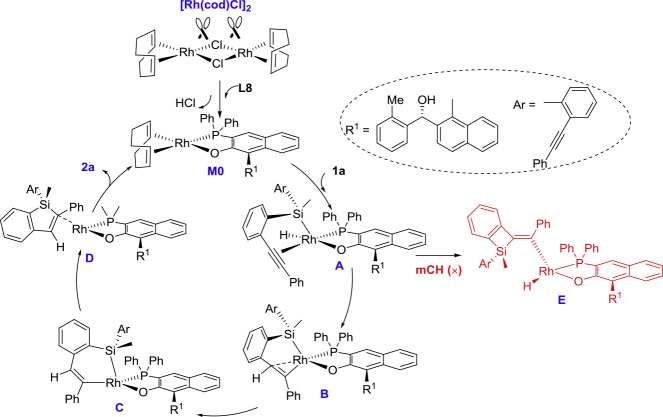
A Proposed Catalytic Cycle for Rh(I)/**L8** Complex-Catalyzed Intramolecular Hydrosilylation with Monoalkyne as a Model Substrate

Therefore, based on the experimental results and related NMR and ESI-MS analysis, we proposed a reaction mechanism for the asymmetric Rh-catalyzed hydrosilylation (Figure 5). It is expected that the dirhodium core in the chlorine-bridged dimeric rhodium precursor is easily broken by a proton abstraction reaction with ligand **L8**, releasing HCl with the aid of KO*t*Bu and generating mononuclear precursor complex **M0**. The cod ligand in the four-coordinated neutral Rh(I) complex will further be replaced by the substrate and leads to an Rh intermediate, in which the alkenyl group and Si-H group of substrate is coordinated to the Rh center in η^2^ and η^1^ manner. Then the Rh intermediate underwent Si-H oxidative addition to form a five-coordinated Rh(III) intermediate **A**. The pre-coordinated alkynyl group is exactly the one to proceed migratory insertion, followed by the Si-H oxidative addition. That is, the two symmetric alkynyl groups in **1a** have already been discriminated during the formation of this precursor complex **B**, in which the reactive alkynyl group and Si-H group linked to the Rh center in enantioselective manner. And then the subsequent process is only related to the Z/E-selectivity but not the enantioselectivity. Similarly to previous Ru catalysis for the trans-selective hydrosilylation of alkynes (Ding et al., 2013), **B** can be further isomerized to the more stable **C** (metallacyclohexene intermediate). At this time, the H atom is completely inverted to the *trans* position, and the alkenyl group is at the *trans* position of the O-ligand. For the origin of stereoselective induction of such rhodium catalyst, more theoretic studies would be continuously undergoing in our laboratory to gain much more accurate understanding of the hydrosilylation reaction mechanism.

### Conclusion

In summary, we accomplished a highly enantioselective Rh-catalyzed intramolecular and *trans*-type hydrosilylation of silicon-tethered bisalkynes, which provided a practical approach to the construction of AIE and CPL-active benzosiloles bearing silicon-stereogenic center. For this purpose, our chiral Ar-BINMOL-Phos bearing hydrogen-bond donors could be efficiently used as a privileged MFMC P,O,O-ligand in the desymmetrization process of silicon-tethered bisalkynes. The reaction is operationally robust and atom-economic with good functional group tolerability as well as high enantioselectivity (up to >99:1 *er*) with the aid of Rh/Ar-BINMOL-Phos-based MFMC ligand catalysis. More specially, the use of the basic additive KO*t*Bu is crucial to maintaining of high level of enantioselectivity in this reaction because the catalytic amount KO*t*Bu is responsible for the formation of active Rh/**L8** complex. Although the true reaction mechanism for the stereoselective induction of Rh catalyst system is still unclear, the highly enantioselective synthesis of chiral benzosiloles and corresponding construction of silicon-stereogenic center by desymmetric hydrosilylation opens a great opportunity to create the next generation of organosilicon material or possibly better targeted Si-containing biologically active molecules that bring silicon to material and life (Kan et al., 2016). At present, the DFT calculation studies are undergoing in our laboratory to gain a more accurate understanding of the mechanism of *trans*-selective hydrosilylation and will be reported elsewhere. In addition, the reactive alkyne groups as side chains in the silicon-stereogenic benzosiloles are expected to undergo further functionalization and hold promise for synthesis of conjugate polymers or cross-linked materials.

### Limitations of the Study

Terminal alkynes were not applicable in the construction of silicon-stereogenic benzosiloles by the intramolecular hydrosilylation. And the reaction mechanism and the origin of enantioselectivity that is controlled by the Ar-BINMOL-Phos need to be clarified in more reliable manner.

### Resource Availability

#### Lead Contact

Li-Wen Xu, liwenxu@hznu.edu.cn.

#### Material Availability

No unique reagents or no restrictions to the availability of chemicals.

#### Data and Code Availability

The related figures and data in this article can be found at the Supplemental Information.

## Methods

All methods can be found in the accompanying Transparent Methods supplemental file.

## Supplemental Information

Document S1. Transparent Methods, Figures S1–S112, and Tables S1–S6

## Footnotes

Supplemental Information can be found online at https://doi.org/10.1016/j.isci.2020.101268.
