# Supplementary material for: Catalytic Asymmetric trans-Selective Hydrosilylation of Bisalkynes to Access AIE and CPL-Active Silicon-Stereogenic Benzosiloles
Source: iScience. 2020 Jun 15;23(7):101268. doi: 10.1016/j.isci.2020.101268 (PMC7326740; doi:10.1016/j.isci.2020.101268)
Supplement: Document S1. Transparent Methods, Figures S1–S112, and Tables S1–S6 [file mmc1.pdf]

iScience, Volume *23*

Supplemental Information

Catalytic Asymmetric *trans*-Selective Hydrosilylation of Bisalkynes to Access AIE and CPL-Active Silicon-Stereogenic Benzosiloles

Ren-He Tang, Zheng Xu, Yi-Xue Nie, Xu-Qiong Xiao, Ke-Fang Yang, Jia-Le Xie, Bin Guo, Guan-Wu Yin, Xue-Min Yang, and Li-Wen Xu

More details for reaction condition optimization:

Table S1. Optimization of reaction conditions by screening of chiral ligands. [a] Related to Figure 1.

Entry	Rh (X mol%)	Ligand	Temp (^{o}C)	Time (h)	**2a/1a**[b]	er (%)[c]
1	2.5	**L1**	80	36	>99:1	85:15
2	2.5	**L2**	80	34	60:40	80:20
3	10	**L3**	60	24	80:20	90:10
4	2.5	**L4**	80	34	62:38	80:20
5	2.5	**L5**	80	34	94:6	70:40
6	2.5	**L6**	80	36	54:46	85:15
7	2.5	**L7**	80	36	60:40	50:50
8	2.5	**L8**	80	34	60:40	85:15
9	2.5	**L9**	80	34	70:30	87.5:12.5
10	2.5	**L10**	80	36	60:40	50:50
11	10	**L11**	60	24	-	65:35
12	10	**L12**	60	24	-	50:50
13	2.5	**L13**	80	36	20:80	50:50
14	10	**L14**	60	24	-	50:50
15	10	**L15**	60	24	-	50:50
16	10	**L16**	60	24	-	59:41
17	10	**L17**	60	24	-	56:44
18	10	**L18**	60	24	-	70:30
19	10	**L19**	60	24	-	65:35
20	10	**L20**	60	24	-	65:35
21	2.5	**L21**	80	24	10:90	55:45
22	5	**L9**	80	24	76:24	90:10
23	5	**L8**	80	24	82:18	91:9

[a] Reaction conditions: **1a** (0.2 mmol), $[Rh(cod)Cl]_2$ (10 mol%), and solvent (1 mL) at 60-80 ^{o}C. [b] The ratio of **2a/1a** was determined by HPLC. [c] The er value was determined by chiral HPLC.

Table S2. Optimization of reaction conditions by screening of solvents.[a] Related to Figure 1.

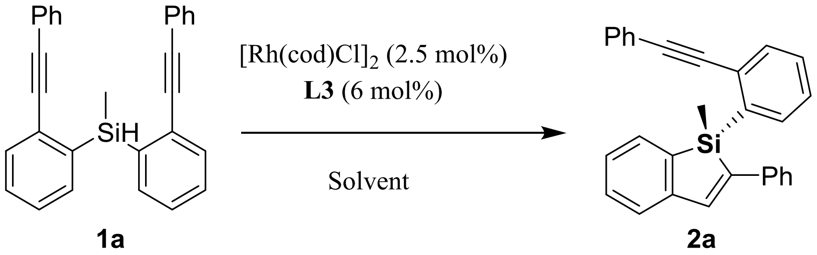

Entry	Solvent	Temp (°C)	Time (h)	**2a/1a**[b]	er (%)[c]
1	Dioxane	70	36	NR	-
2	CH_3CN	70	36	-	50:50
3	Ethanol	70	36	NR	-
4	THF	70	36	-	77.5:25.5
5	DCE	70	36	NR	50:50
6	Benzene	80	22	>99:1	91:9
7	*m*-Xylene	80	22	>99:1	90:10
8	*p*-Xylene	80	22	80:20	85:15
9	*o*-Xylene	80	22	94:6	82.5:17.5
10	Toluene	70	24	80:20	90:10
11	1,2,4-Trimethylbenzene	80	22	88:12	90:10

[a] Reaction conditions: **1a** (0.2 mmol), $[Rh(cod)Cl]_2$ (10 mol%), and solvent (1 mL) at 70-80 °C. [b] Determined by HPLC. [c] The er value was determined by chiral HPLC with a chiral stationary phase.

Table S3. Evaluation of catalytic activity of transition-metal catalysts. [a] Related to Table 1.

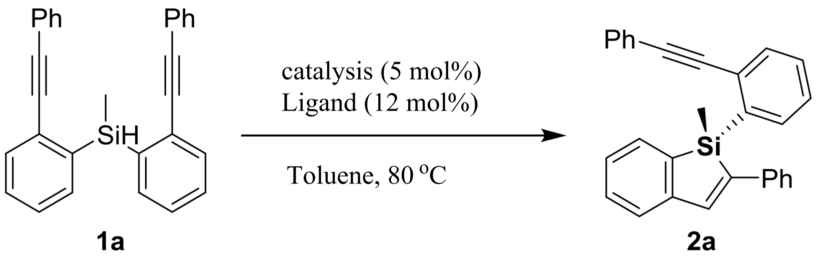

Entry	Catalyst	Ligand	Time (h)	**2a/1a**[b]	er (%)[c]
1	$[RhCl(C_2H_2)]_2$	**L3**	34	NR	-
2	$[(C_6H_5)_3P]_3RhCl$	**L3**	34	NR	-
3	$Rh_2(OOCCH_3)_4$	**L3**	34	NR	-
4	$[Rh(cod)_2]BF_4$	**L3**	34	NR	-
5	$[Rh(OAc)_2]_2$	**L3**	34	NR	-
6	$[Rh(nbd)_2]Cl_2$	**L3**	34	NR	-
7	$Rh(CO)_2(C_5H_7O_2)$	**L3**	34	NR	-
8	$RhCl_2(CO)_4$	**L3**	34	NR	-
9	$(\eta_3\text{-}C_3H_5)_2Pd_2Cl_2$	**L8**	14	NR	-
10	$PdCl_2$	**L8**	14	NR	-
11	$Pd_2(dba)_3$	**L8**	14	10:90	62.5:37.5
12	$PdCl_2(dppb)$	**L8**	14	14:86	50:50
13	$Pd(PPh_3)Cl_2$	**L8**	14	22:78	65:35
14	OIP Co	-	14	10:90	50:50
15[d]	$[Rh(cod)Cl]_2$.	**L8**	14	20:80	90:10
16[e]	$[Rh(cod)Cl]_2$.	**L8**	14	8:92	95.5:4.5

[a] Reaction conditions: **1a** (0.2 mmol), $[Rh(cod)Cl]_2$ (10 mol%), and solvent (1 mL) at 80 °C. [b] Determined by HPLC. [c] The er value was determined by chiral HPLC with a chiral stationary phase. [d] At 80 °C. [e] At 60 °C.

Table S4. Optimization of reaction conditions by screening of additives. [a] Related to Table 1.

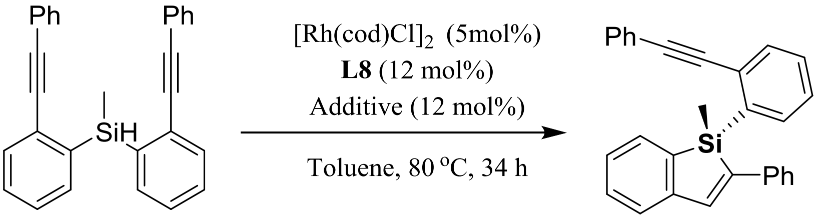

Entry	Additive	**2a/1a**[b]	er (%)[c]
1	$AlCl_3$	NR	
2	$NaHBEt_3$	>99:1	92.5:7.5
3	$NaSbF_6$	NR	
4	K_2CO_3	60:40	90.5:9.5
5	$AlCl_3$	30:70	85:15
6	CuI	60:40	90:10
7	KO*t*Bu	>99:1	95:5
8	Ag_3PO_4	90:10	92:8
9	NEt_3	90:10	92:8
10	NaO^tBu	>99:1	90:10
11	NaOEt	>99:1	85:15

[a] Reaction conditions: **1a** (0.2 mmol), $[Rh(cod)Cl]_2$ (5 mol%), and solvent (1 mL) at 70 °C. [b] Determined by HPLC. NR is no reaction. [c] The er value was determined by chiral HPLC with a chiral stationary phase.

Table S5. The effect of KO*t*Bu on the Rh-catalyzed intramolecular hydrosilylation.[a] Related to Table 1.

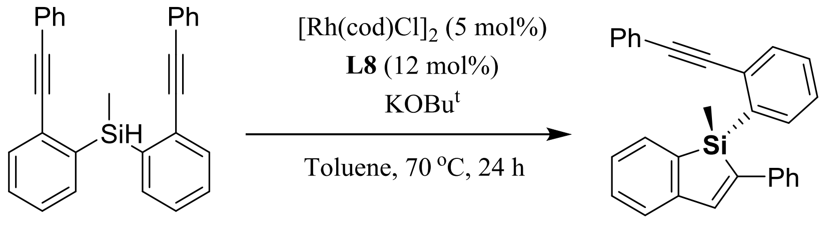

Entry	KO*t*Bu (X mol%)	**2a/1a**[b]	er (%)[c]
1	6	>99:1	95.5:4.5
2	12	>99:1	95.5:4.5
3	24	>99:1	95.5:4.5
4	36	>99:1	75:25
5	48	NR	-
6	60	NR	-

[a] Reaction conditions: **1a** (0.2 mmol), $[Rh(cod)Cl]_2$ (5 mol%), and solvent (1 mL) at 70 °C. [b] Determined by HPLC. [c] The er value was determined by chiral HPLC with a chiral stationary phase.

Table S6a. Kinetic studies on the Rh-catalyzed intramolecular hydrosilylation: a) with KO*t*Bu. [a] Related to Figure 4.

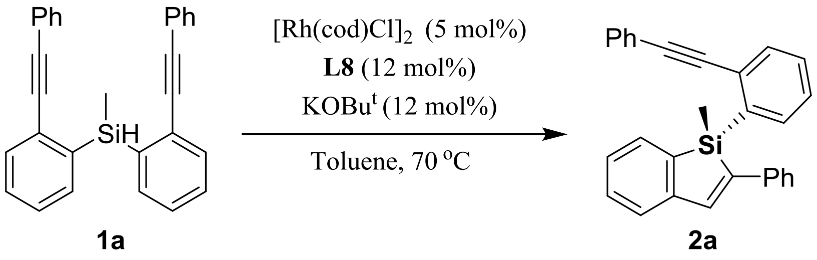

Entry	Catalyst	Temp (°C)	Time (h)	**2a/1a**[b]	*er* (%)[c]
1	$[Rh(cod)Cl]_{2.}$	70	1	18:82	>99:1
2	$[Rh(cod)Cl]_{2.}$	70	2	18:82	>99:1
3	$[Rh(cod)Cl]_{2.}$	70	4	28:72	95.5:4.5
4	$[Rh(cod)Cl]_{2.}$	70	14	30:70	95.5:4.5
5	$[Rh(cod)Cl]_{2.}$	70	40	54:46	95.5:4.5
6	$[Rh(cod)Cl]_{2.}$	70	70	>99:1	95.5:4.5

[a] Reaction conditions: **1a** (0.2 mmol), $[Rh(cod)Cl]_2$ (5 mol%), and solvent (1 mL) at 70 °C. [b] Determined by HPLC. [c] The er value was determined by chiral HPLC with a chiral stationary phase.

Table S6b. Kinetic studies on the Rh-catalyzed intramolecular hydrosilylation: b) without KO*t*Bu. [a] Related to Figure 4.

Entry	Catalyst	Temp (°C)	Time (h)	*er* (%)[b]
1	$[Rh(cod)Cl]_2$	70	2	97.5:2.5
2	$[Rh(cod)Cl]_2$	70	10	91:9
3	$[Rh(cod)Cl]_2$	70	22	91:9
4	$[Rh(cod)Cl]_2$	70	34	85:15
5	$[Rh(cod)Cl]_2$	70	50	75:25

[a] Reaction conditions: **1a** (0.2 mmol), $[Rh(cod)Cl]_2$ (5 mol%), and solvent (1 mL) at 70 °C. [b] The er value was determined by chiral HPLC with a chiral stationary phase.

Figure S1. X-ray structures of **2r** (CCDC 1954490). Related to Scheme 2.

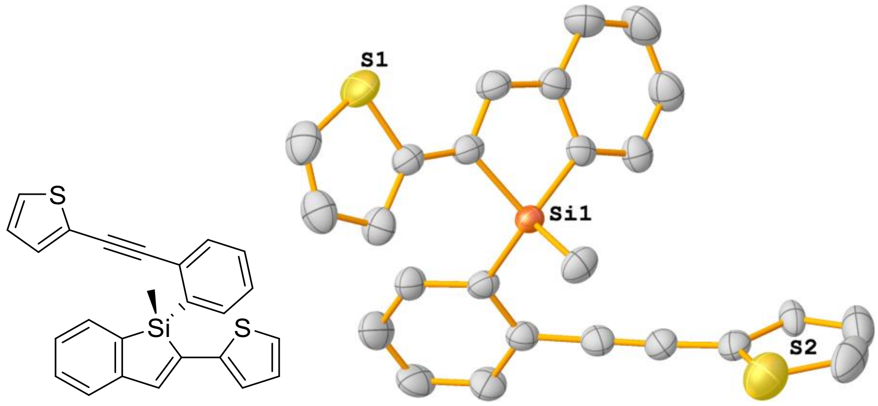

2r

Crystallographic data and data collection for the product **2r**

Formula	$C_{25}H_{18}S_2Si$	*Z*	2
Dcalc./ g cm^{-3}	1.298	*Z'*	2
μ/mm^{-1}	2.887	Wavelength/Å	1.54178
Formula Weight	410.60	Radiation type	CuK
Colour	colourless	θ_{min}/ °	4.076
Shape	prism	θ_{max}/ °	71.022
Size/mm^3	0.15×0.12×0.10	Measured Refl's.	33860
T/K	296.15	Ind't Refl's	7453
Crystal System	triclinic	Refl's with I > 2(I)	7409
Flack Parameter	0.101(7)	R_{int}	0.0342
Hooft Parameter	0.103(5)	Parameters	696
Space Group	*P1*	Restraints	195
a/Å	8.6621(2)	Largest Peak	0.180
b/Å	11.3298(3)	Deepest Hole	-0.221
c/Å	11.8647(3)	GooF	1.050
α/ °	73.2290(10)	wR_2 *(all data)*	0.0871
β/ °	70.5240(10)	wR_2	0.0870
γ/ °	83.5540(10)	R_1 *(all data)*	0.0315
V/Å^3	1050.92(5)	R_1	0.0314

Supplemental Figures for NMR spectrums:

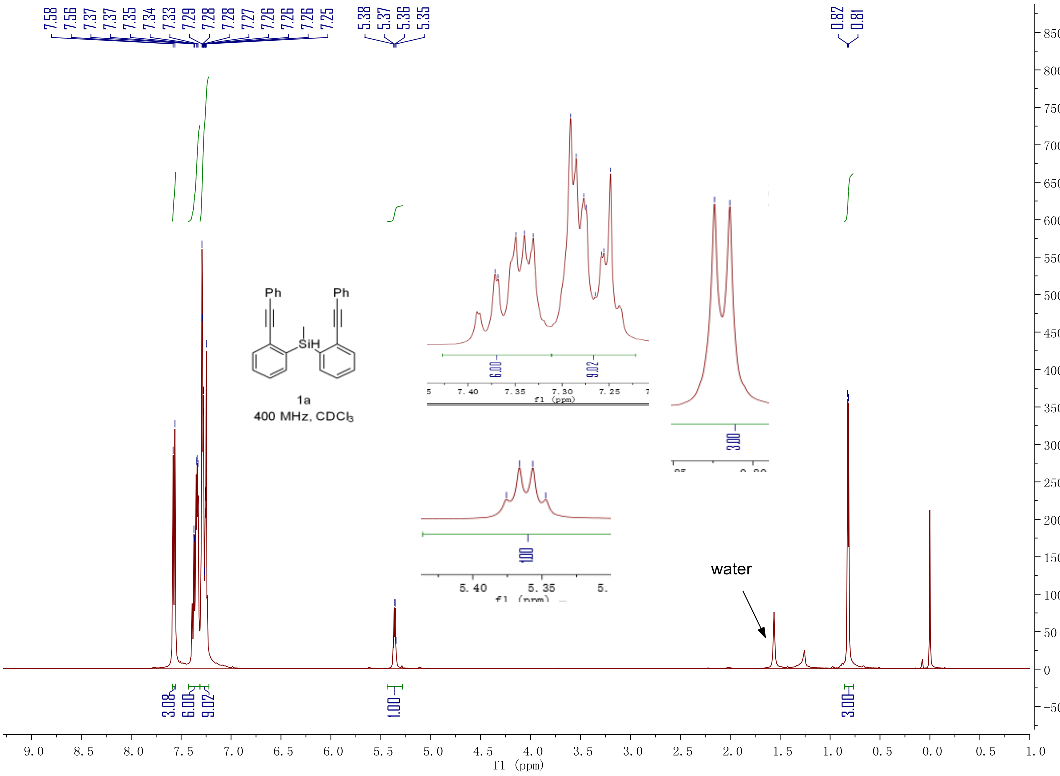

Figure S2. ^{1}H NMR (400 MHz, $CDCl_3$) spectrum of compound **1a**, related to **Scheme 2**

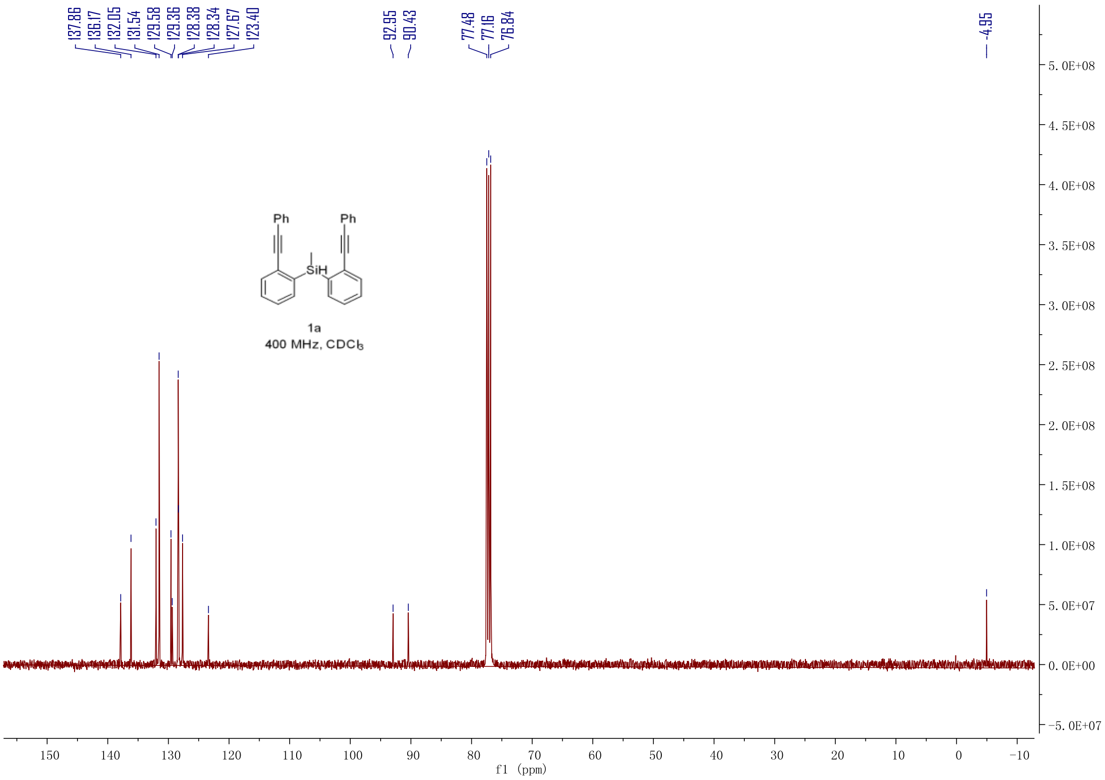

Figure S3. ^{13}C NMR (100 MHz, $CDCl_3$) spectrum of compound **1a**, related to **Scheme 2**

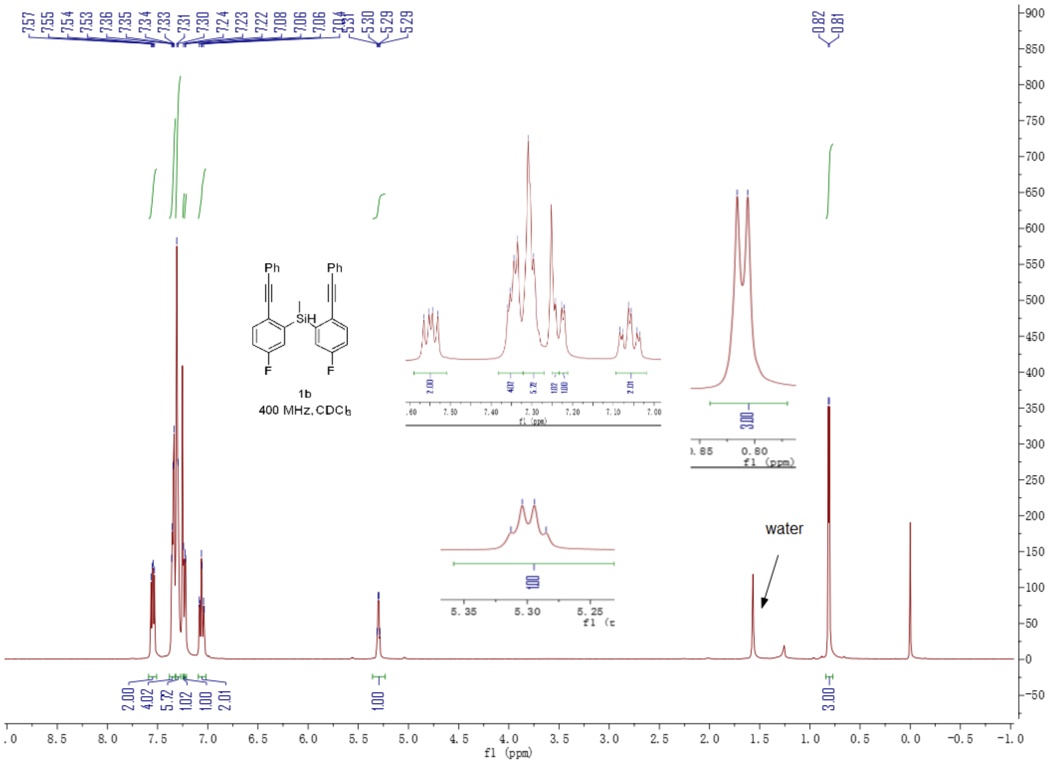

Figure S4. ^{1}H NMR (400 MHz, $CDCl_3$) spectrum of compound **1b** , related to **Scheme 2**

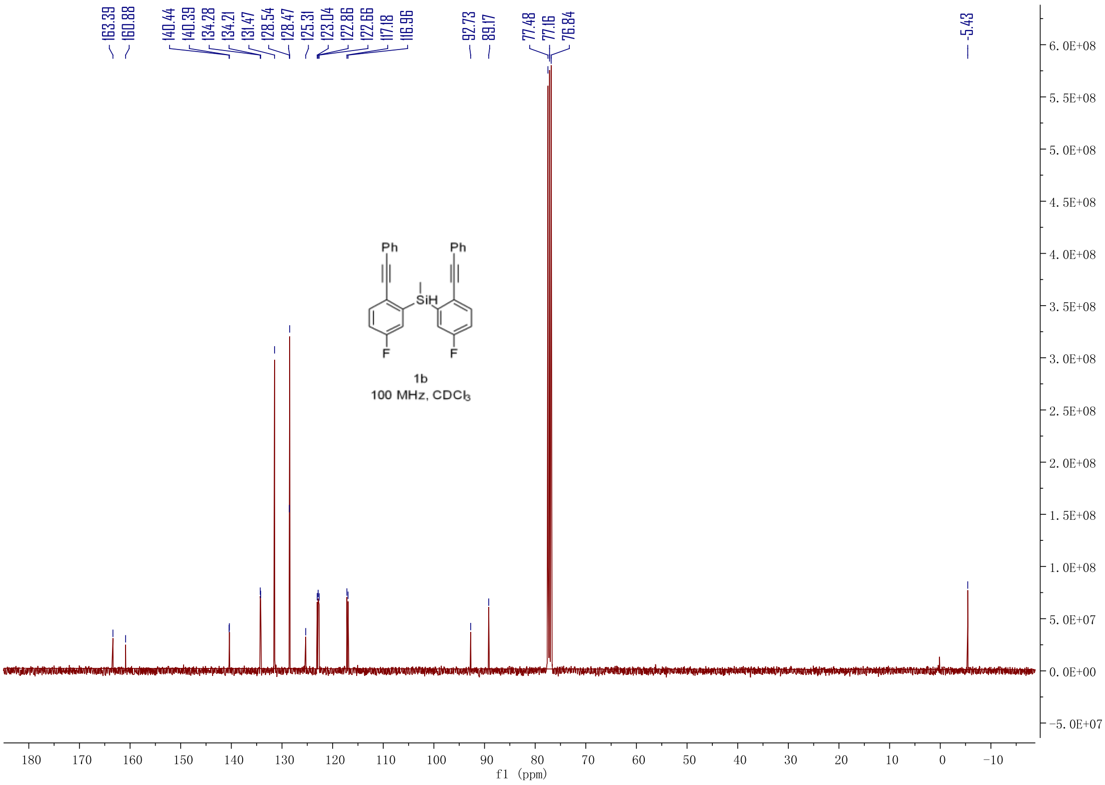

Figure S5. ^{13}C NMR (100 MHz, $CDCl_3$) spectrum of compound **1b**, related to **Scheme 2**

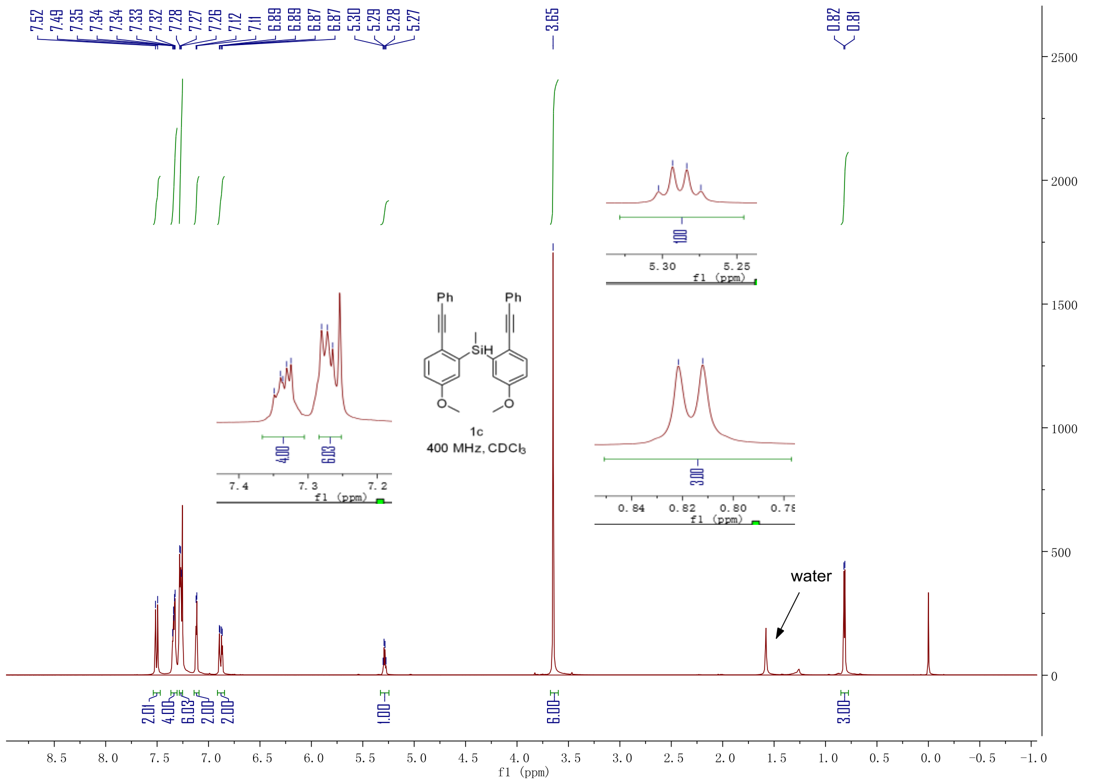

Figure S6. ^{1}H NMR (400 MHz, $CDCl_3$) spectrum of compound **1c**, related to **Scheme 2**

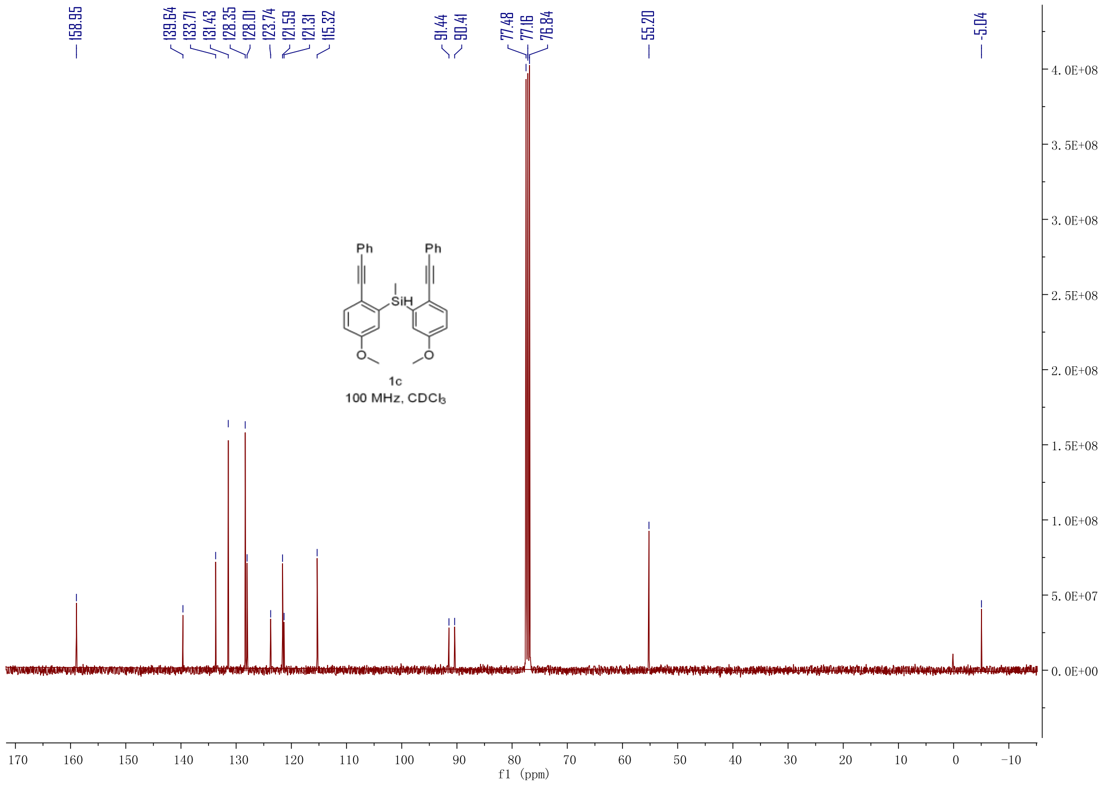

Figure S7. ^{13}C NMR (100 MHz, $CDCl_3$) spectrum of compound **1c**, related to **Scheme 2**

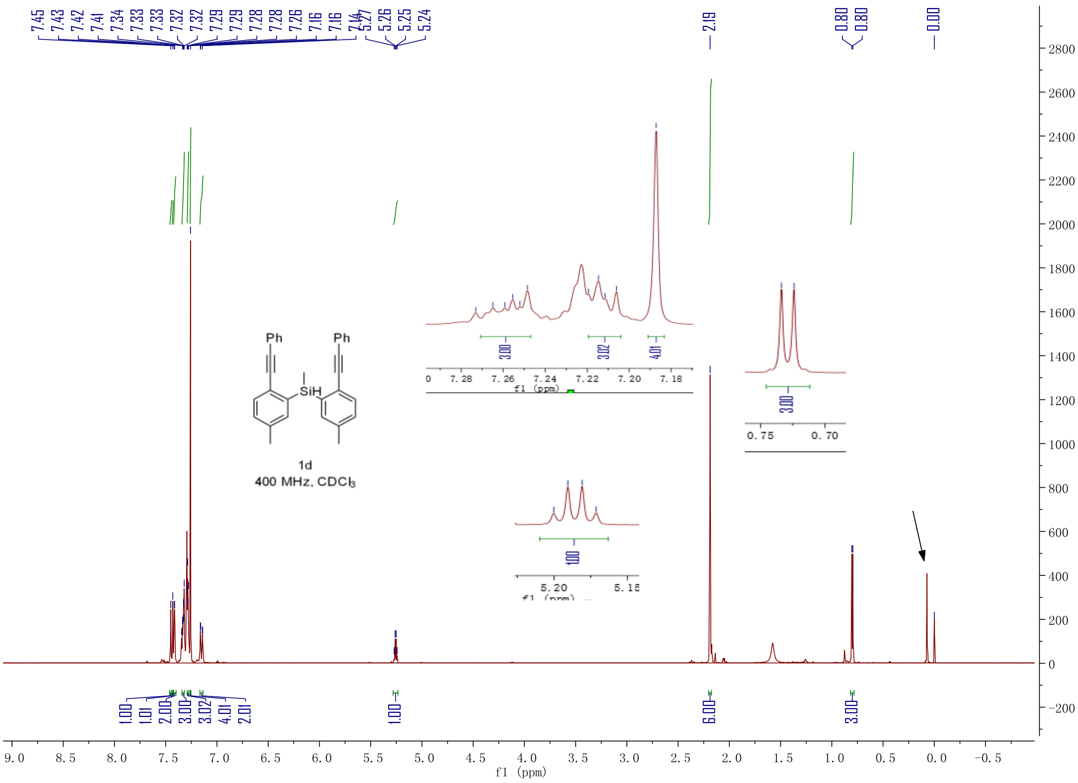

Figure S8. ^{1}H NMR (400 MHz, $CDCl_3$) spectrum of compound **1d**, related to **Scheme 2**

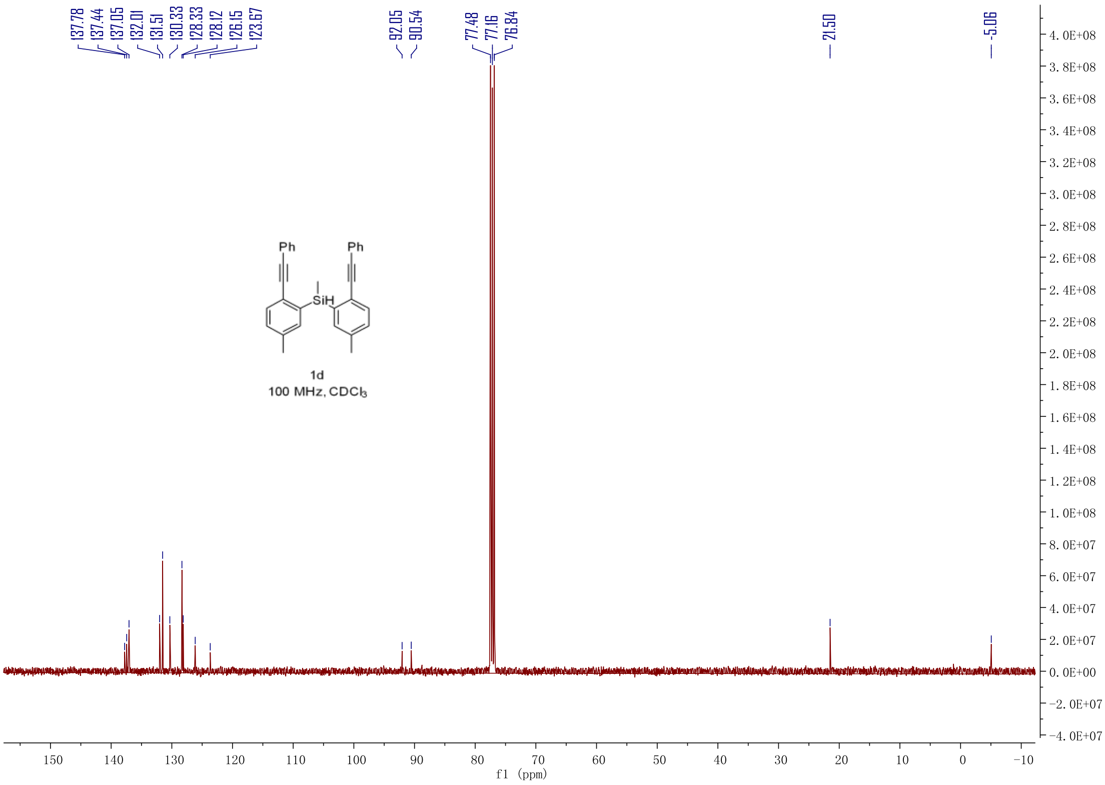

Figure S9. ^{13}C NMR (100 MHz, $CDCl_3$) spectrum of compound **1d**, related to **Scheme 2**

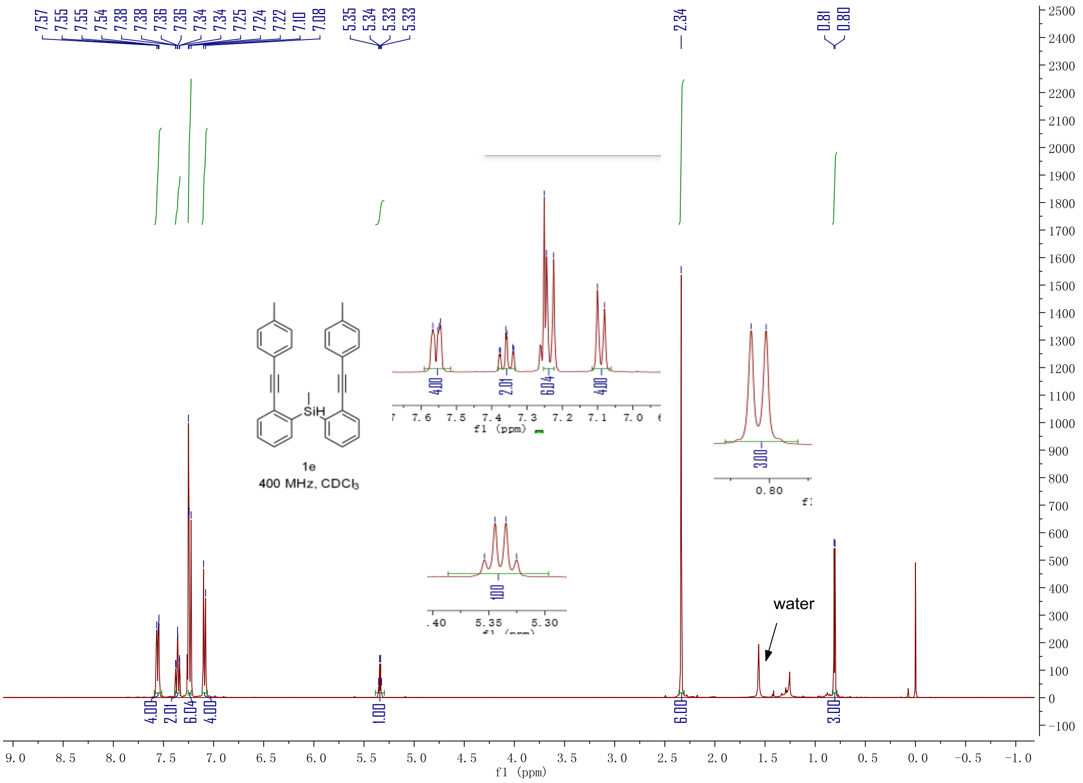

Figure S10. ^{1}H NMR (400 MHz, $CDCl_3$) spectrum of compound **1e**, related to **Scheme 2**

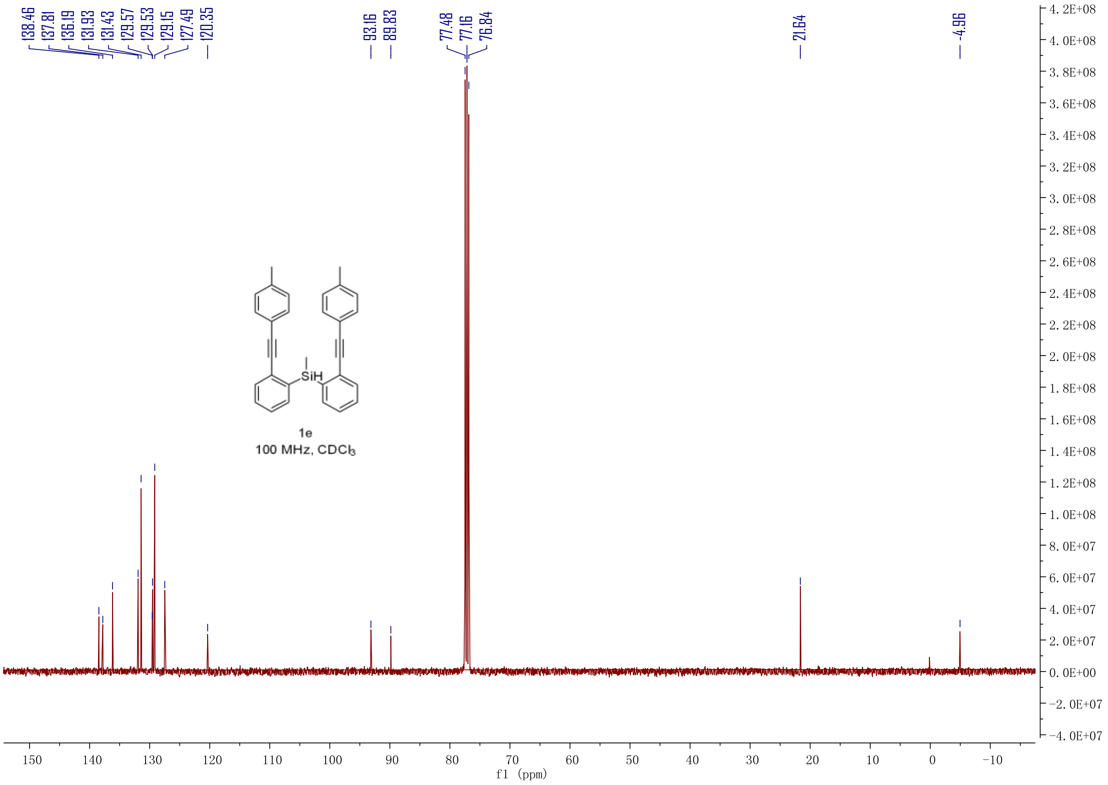

Figure S11. ^{13}C NMR (100 MHz, $CDCl_3$) spectrum of compound **1e**, related to **Scheme 2**

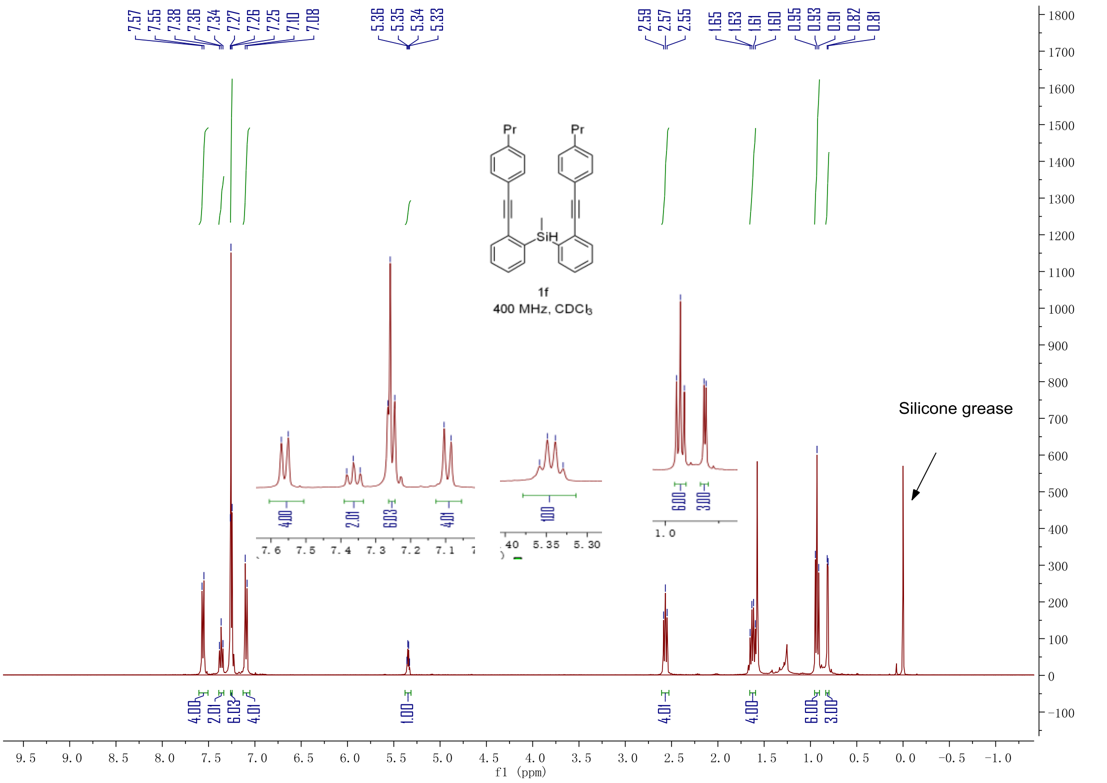

Figure S12. ^{1}H NMR (400 MHz, $CDCl_3$) spectrum of compound **1f**, related to **Scheme 2**

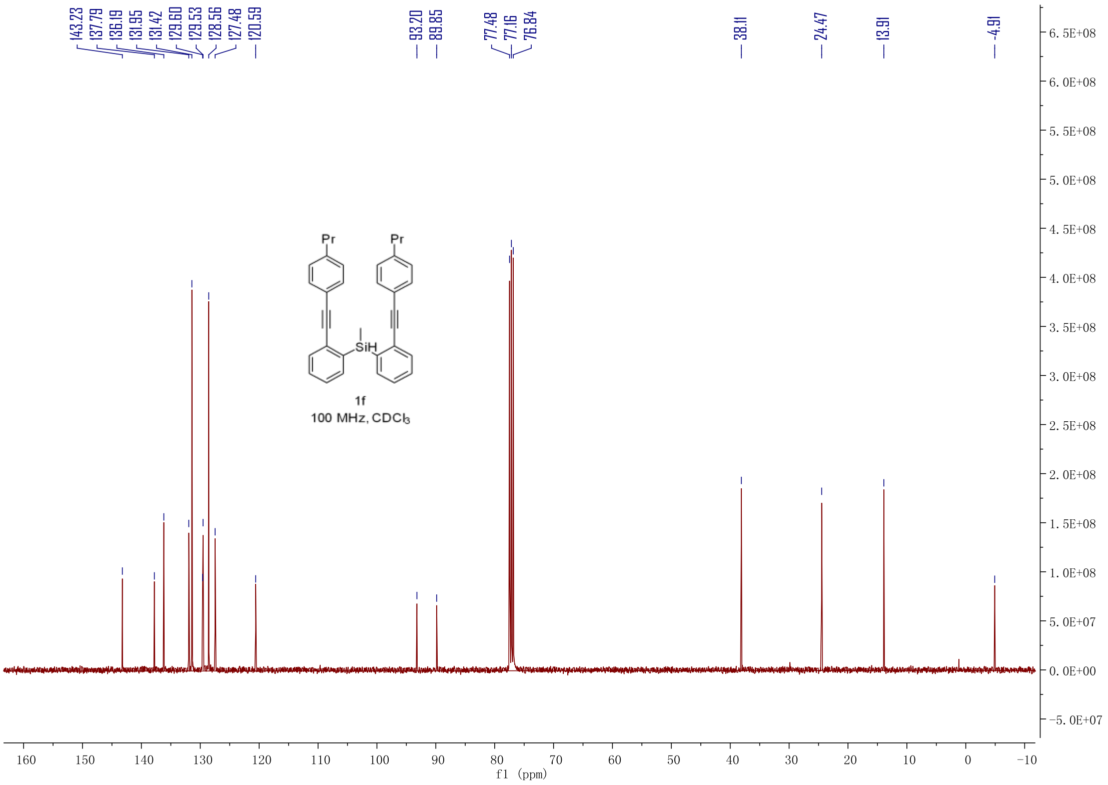

Figure S13. ^{13}C NMR (100 MHz, $CDCl_3$) spectrum of compound **1f**, related to **Scheme 2**

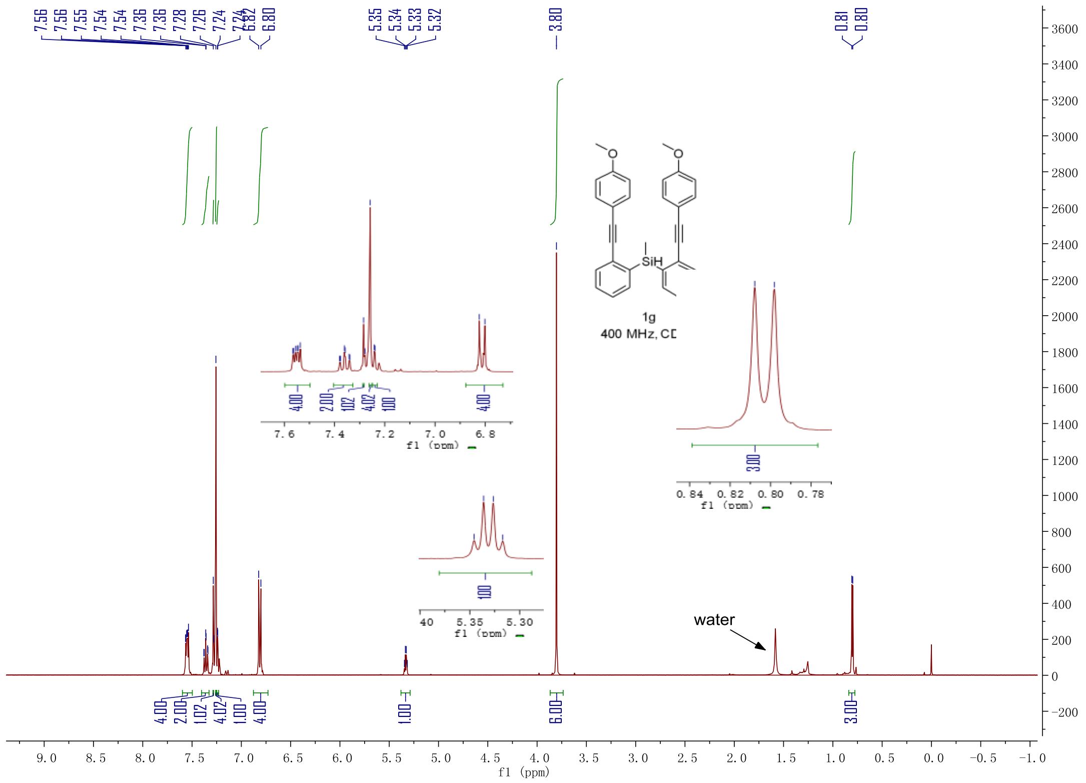

Figure S14. ^{1}H NMR (400 MHz, $CDCl_3$) spectrum of compound **1g**, related to **Scheme 2**

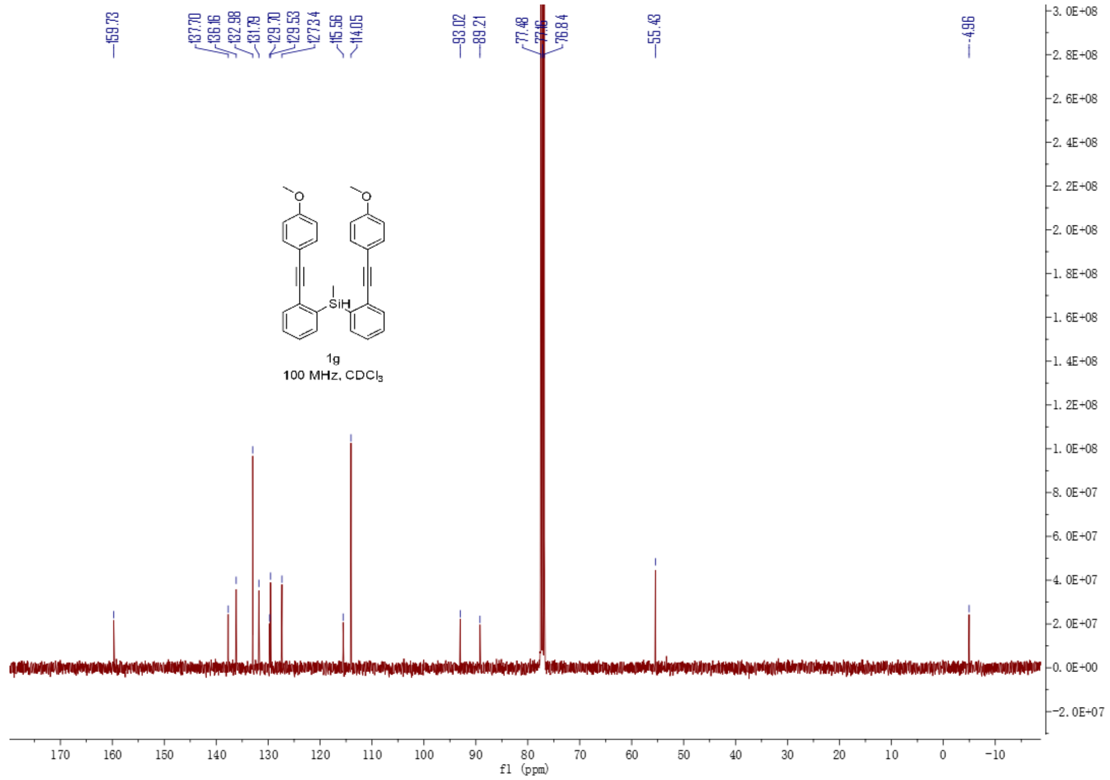

Figure S15. ^{13}C NMR (100 MHz, $CDCl_3$) spectrum of compound **1g**, related to **Scheme 2**

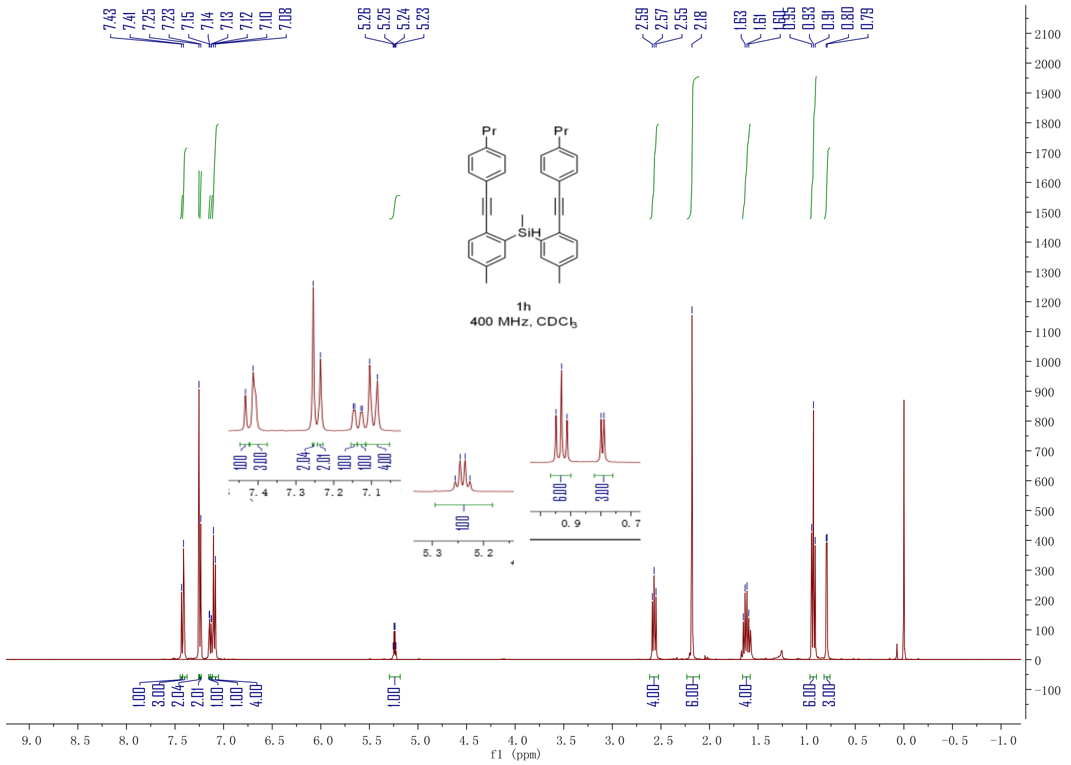

Figure S16. ^{1}H NMR (400 MHz, $CDCl_3$) spectrum of compound **1h**, related to **Scheme 2**

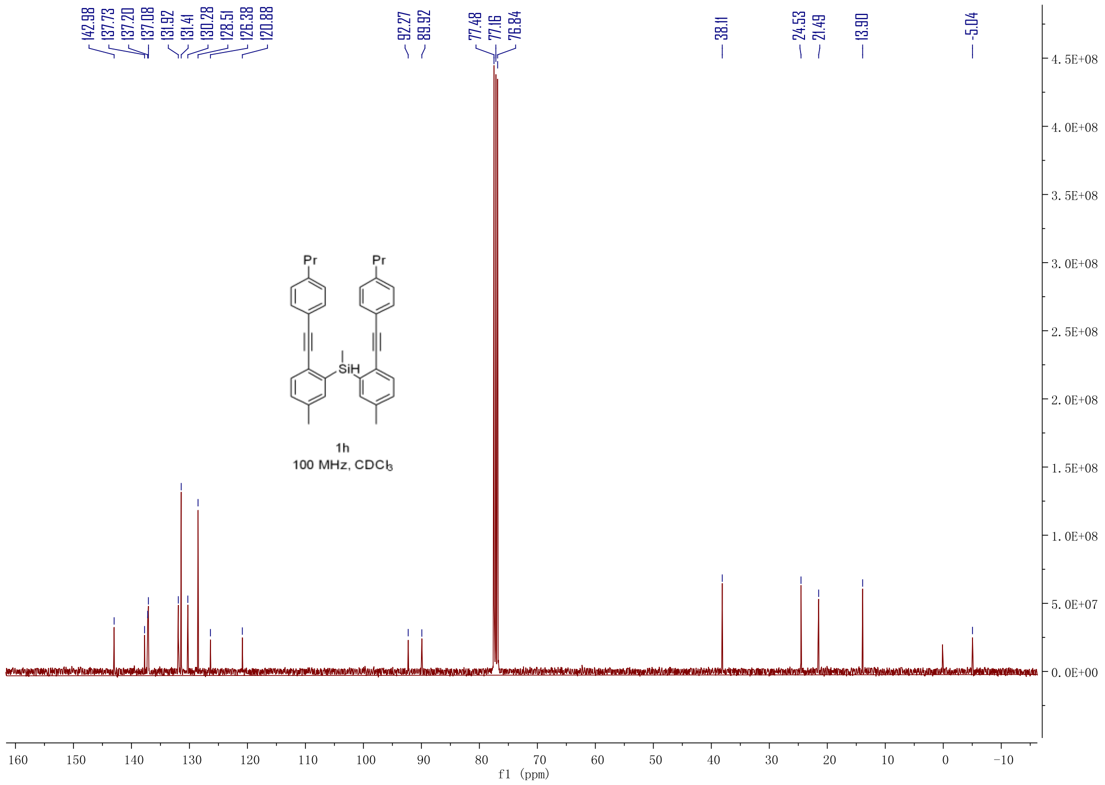

Figure S17. ^{13}C NMR (100 MHz, $CDCl_3$) spectrum of compound **1h**, related to **Scheme 2**

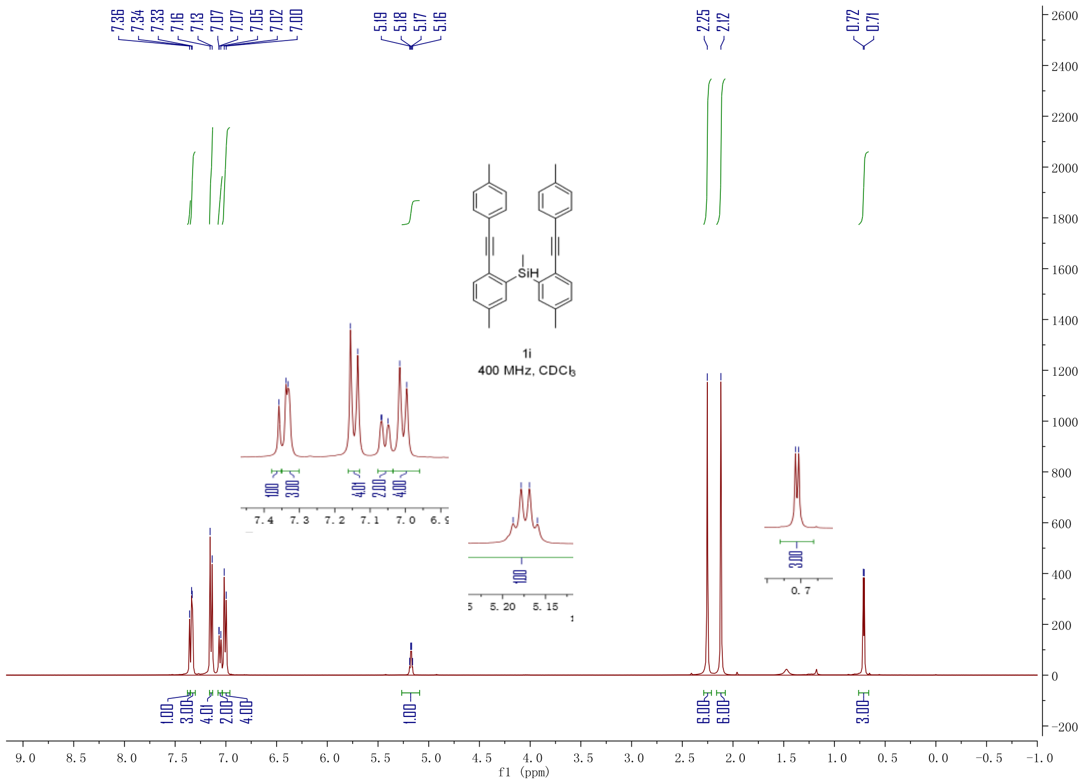

Figure S18. ^{1}H NMR (400 MHz, $CDCl_3$) spectrum of compound **1i**, related to **Scheme 2**

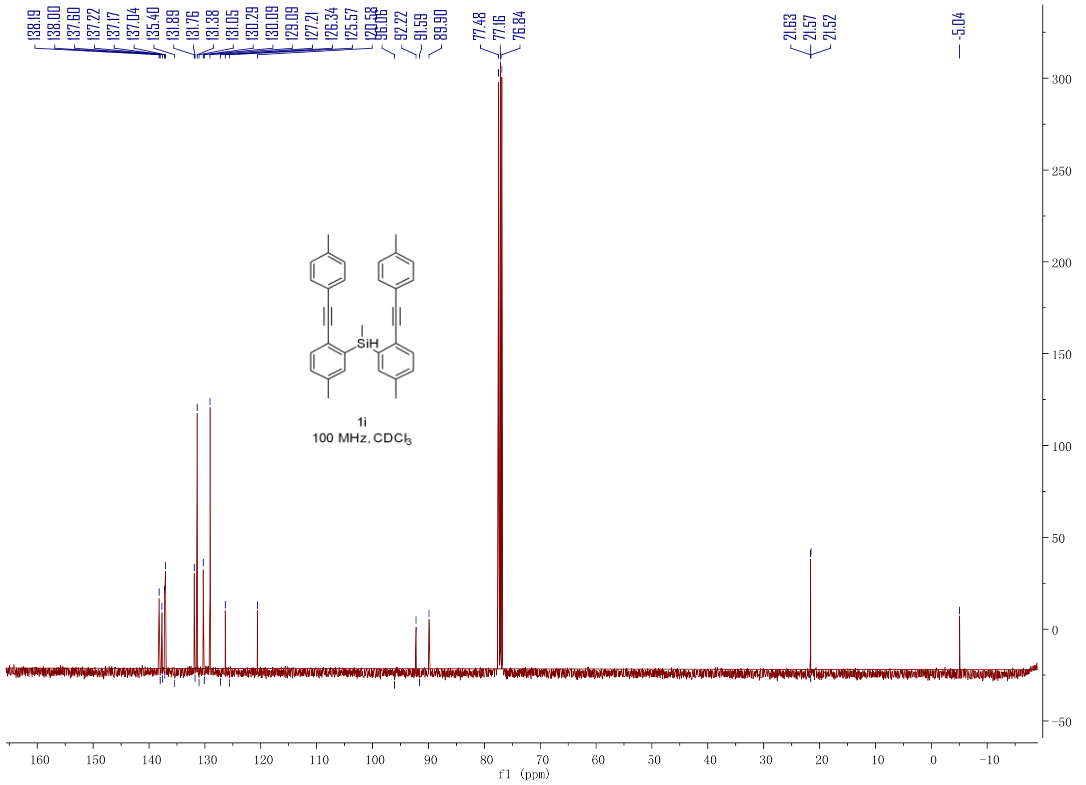

Figure S19. ^{13}C NMR (100 MHz, $CDCl_3$) spectrum of compound **1i**, related to **Scheme 2**

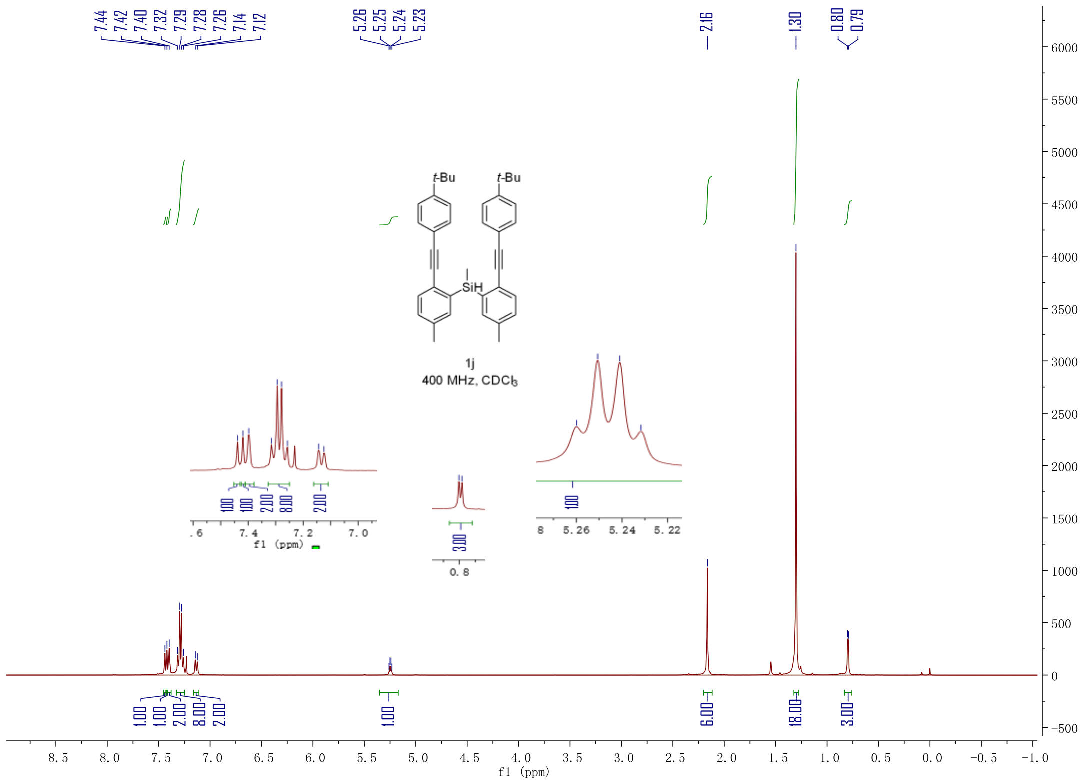

Figure S20. ^{1}H NMR (400 MHz, $CDCl_3$) spectrum of compound **1j**, related to **Scheme 2**

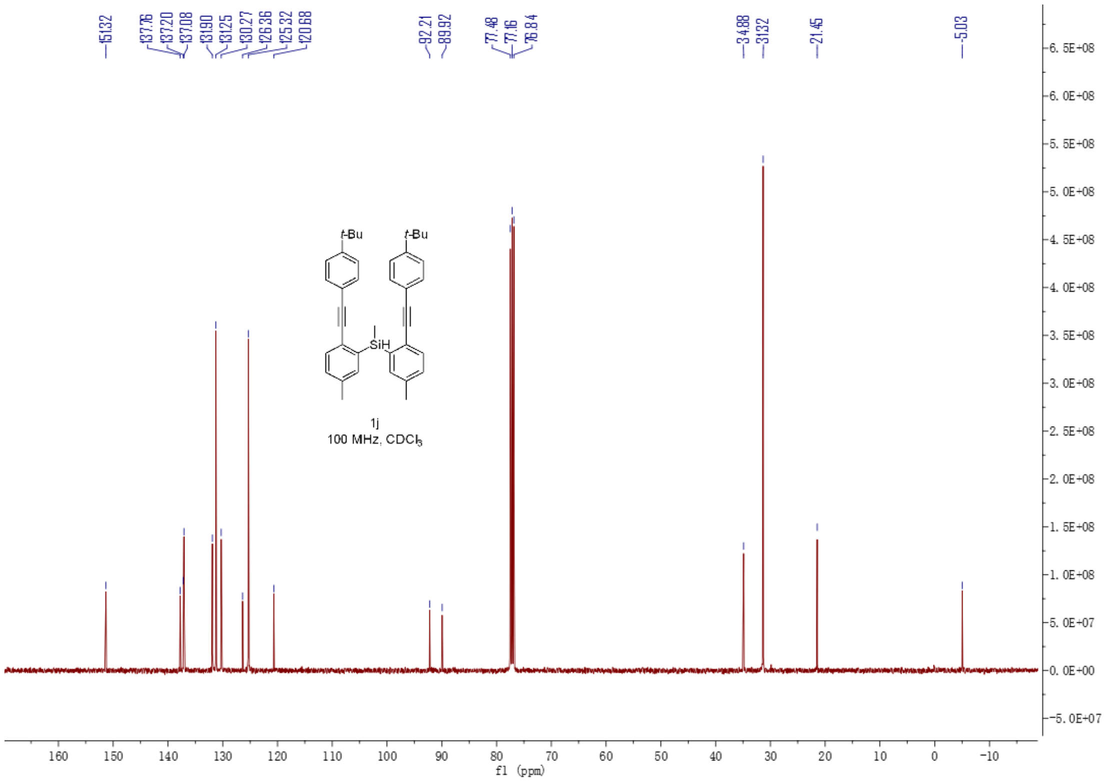

Figure S21. ^{13}C NMR (100 MHz, $CDCl_3$) spectrum of compound **1j**, related to **Scheme 2**

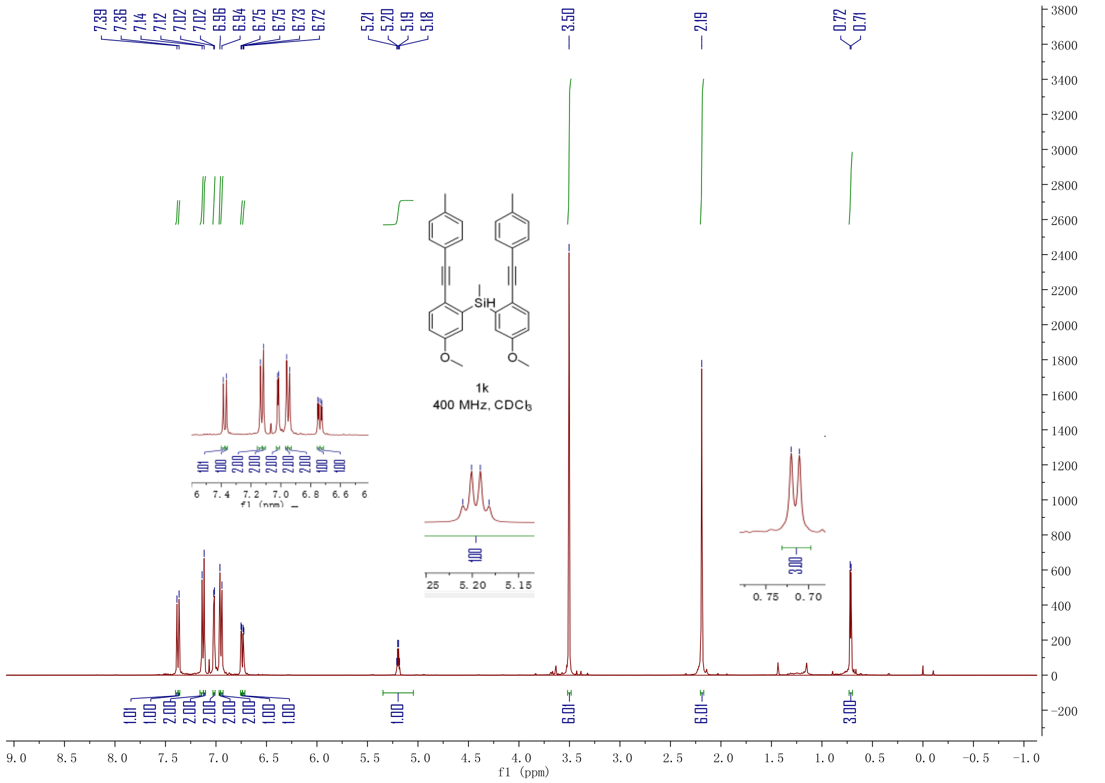

Figure S22. ^{1}H NMR (400 MHz, $CDCl_3$) spectrum of compound **1k**, related to **Scheme 2**

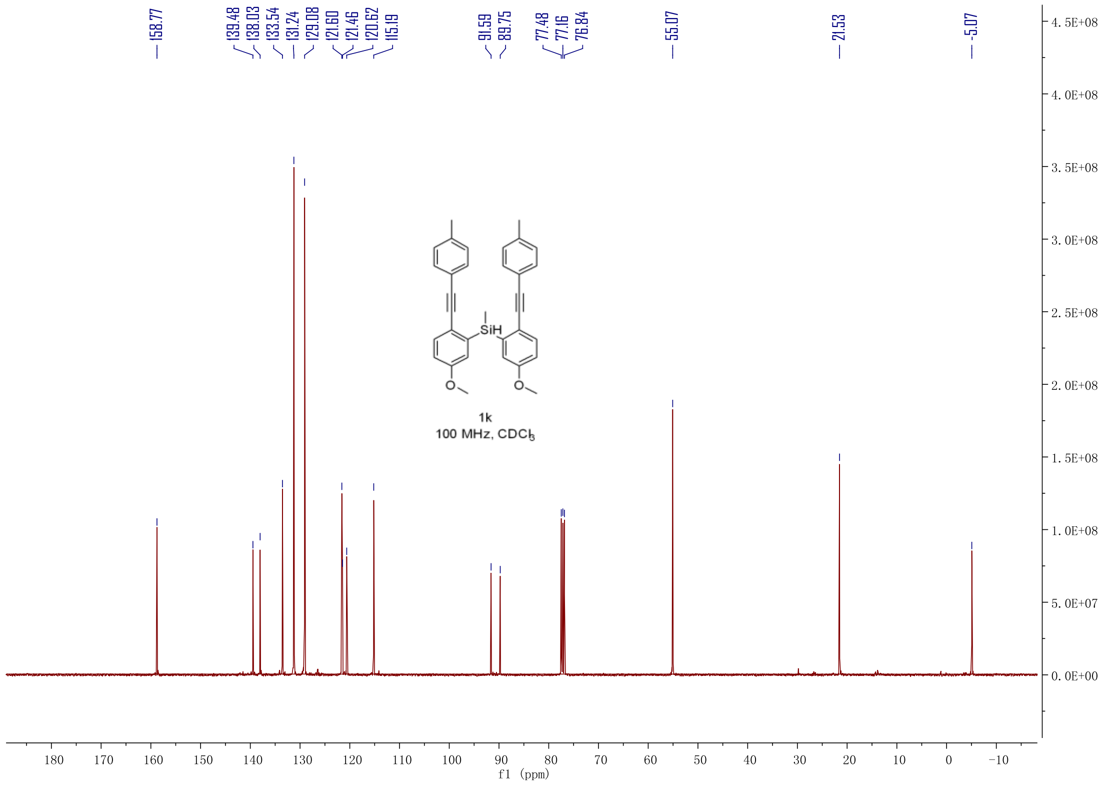

Figure S23. ^{13}C NMR (100 MHz, $CDCl_3$) spectrum of compound **1k**, related to **Scheme 2**

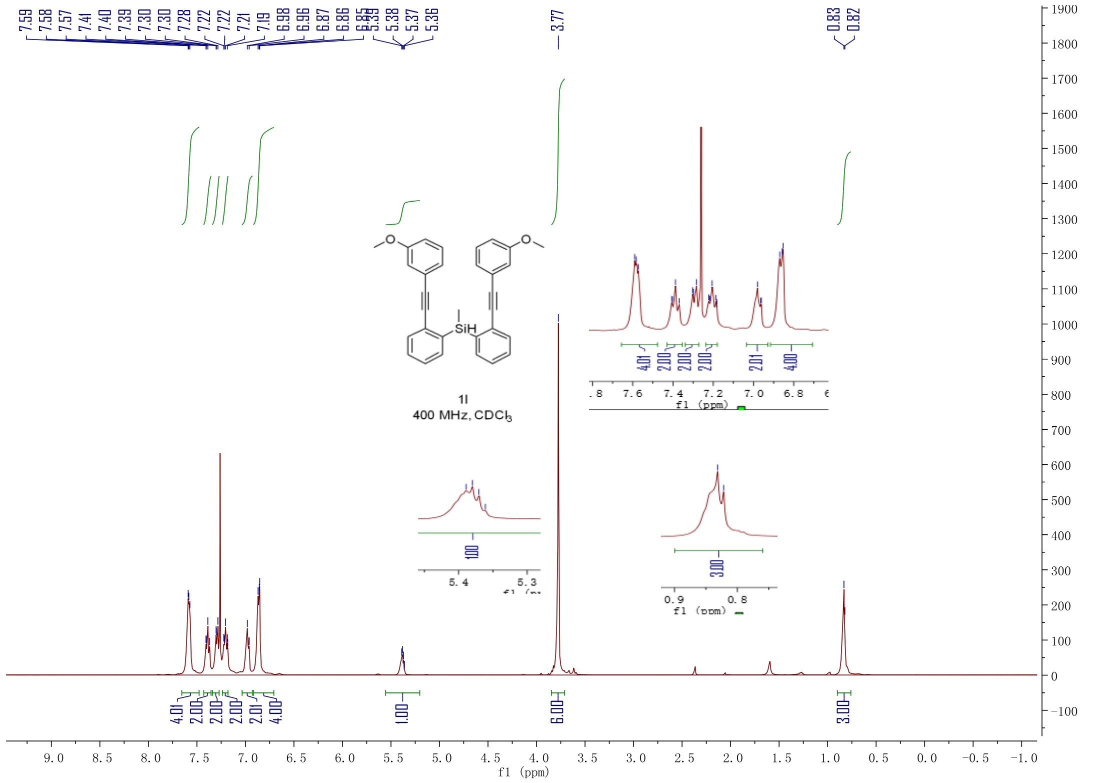

Figure S24. ^{1}H NMR (400 MHz, $CDCl_3$) spectrum of compound **1l**, related to **Scheme 2**

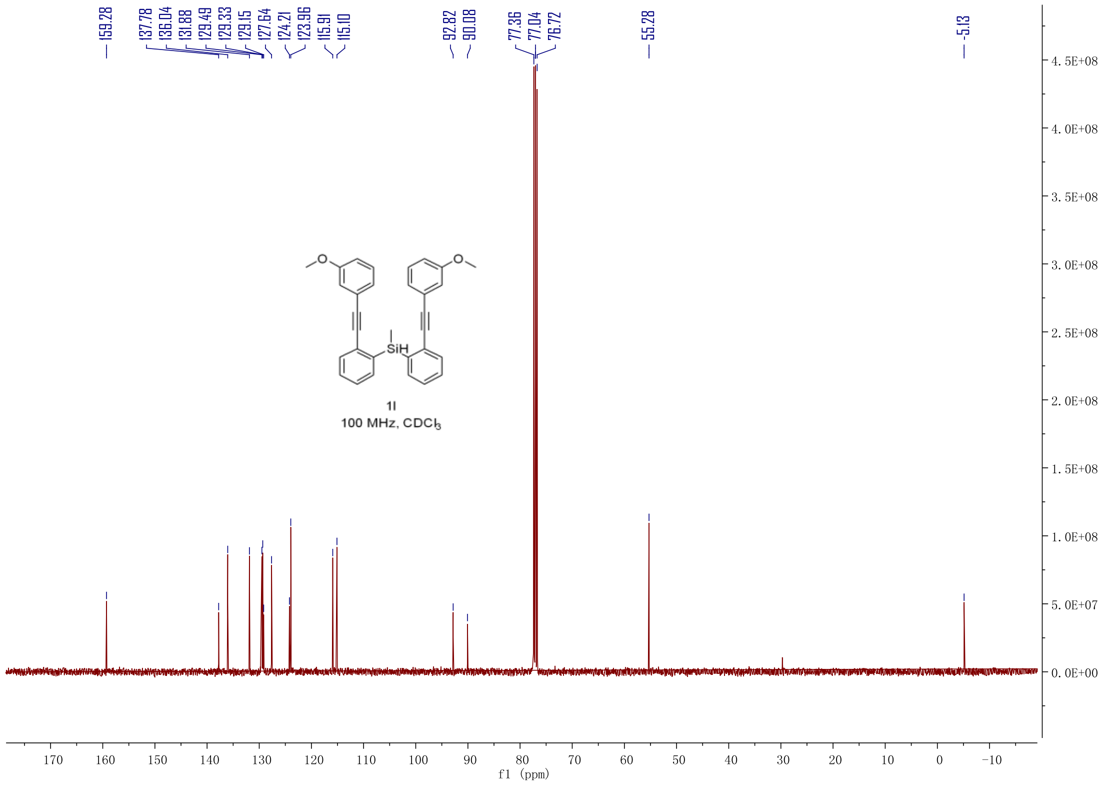

Figure S25. ^{13}C NMR (100 MHz, $CDCl_3$) spectrum of compound **1l**, related to **Scheme 2**

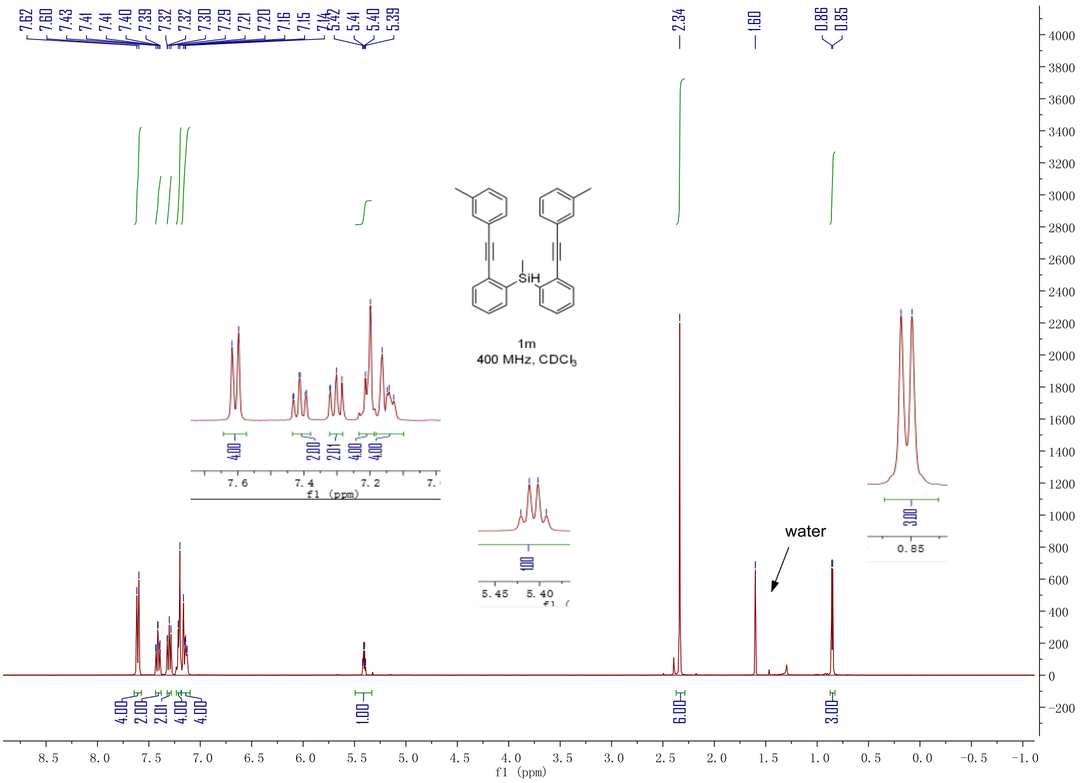

Figure S26. ^{1}H NMR (400 MHz, $CDCl_3$) spectrum of compound **1m**, related to **Scheme 2**

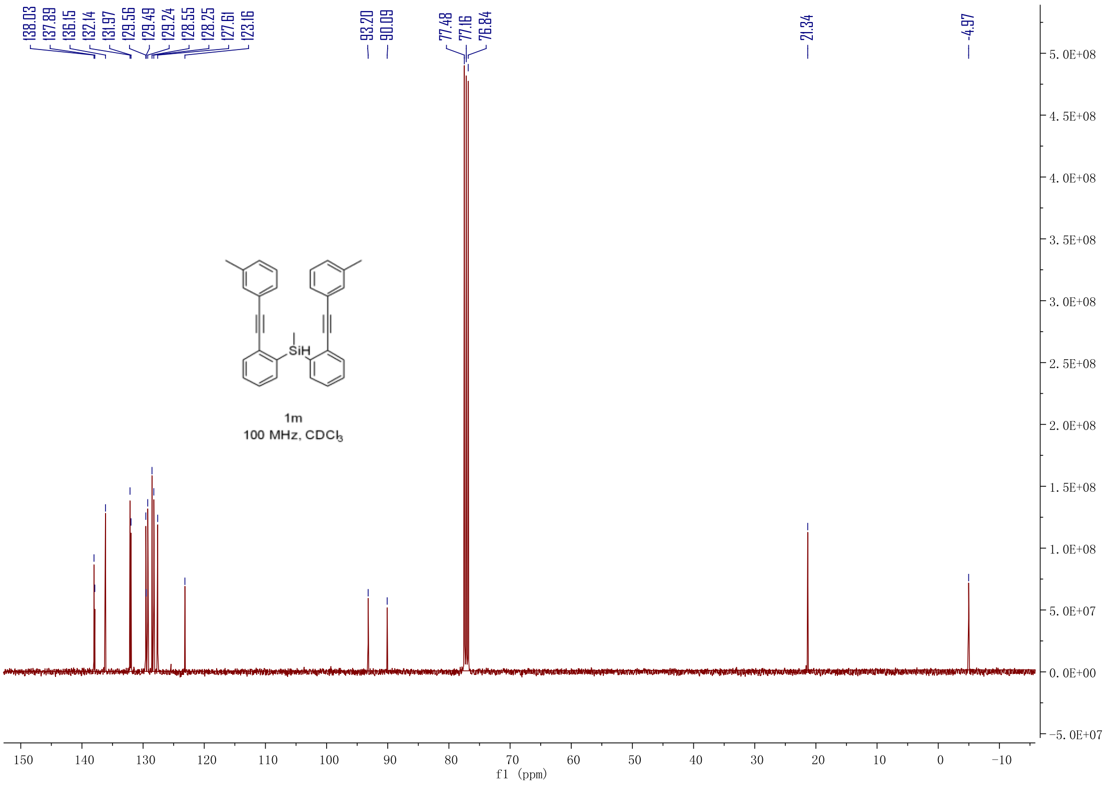

Figure S27. ^{13}C NMR (100 MHz, $CDCl_3$) spectrum of compound **1m**, related to **Scheme 2**

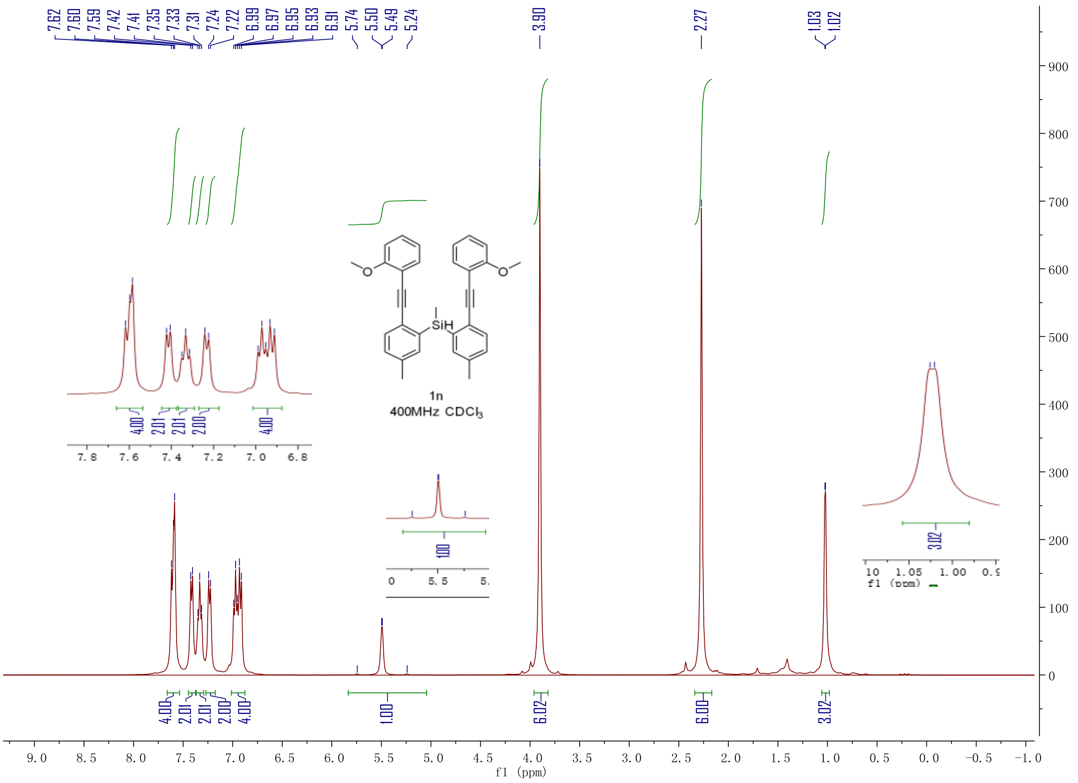

Figure S28. ^{1}H NMR (400 MHz, $CDCl_3$) spectrum of compound **1n**, related to **Scheme 2**

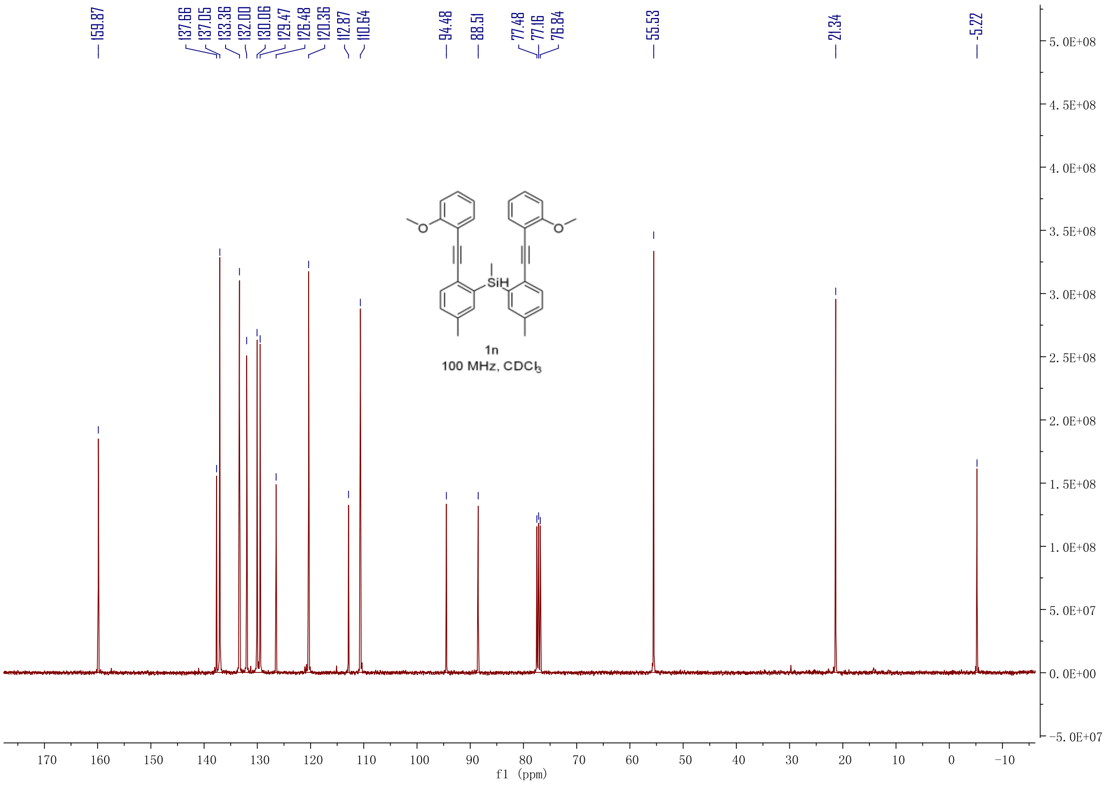

Figure S29. ^{13}C NMR (100 MHz, $CDCl_3$) spectrum of compound **1n**, related to **Scheme 2**

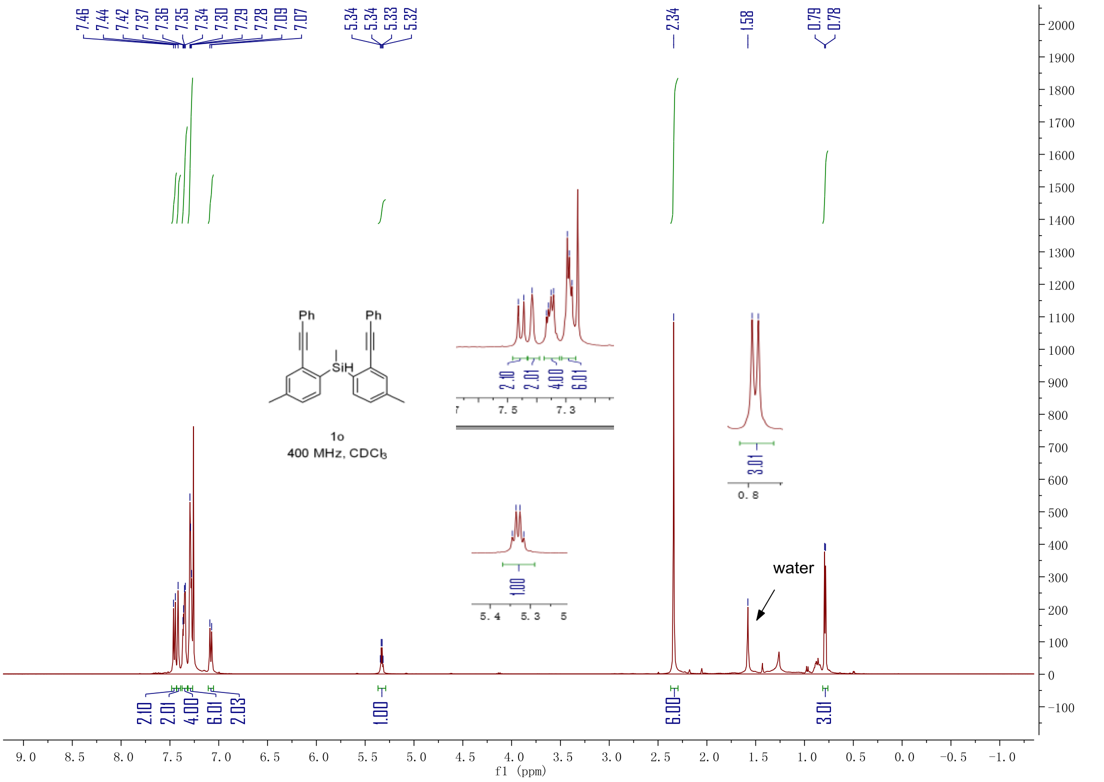

Figure S30. ^{1}H NMR (400 MHz, $CDCl_3$) spectrum of compound **1o**, related to **Scheme 2**

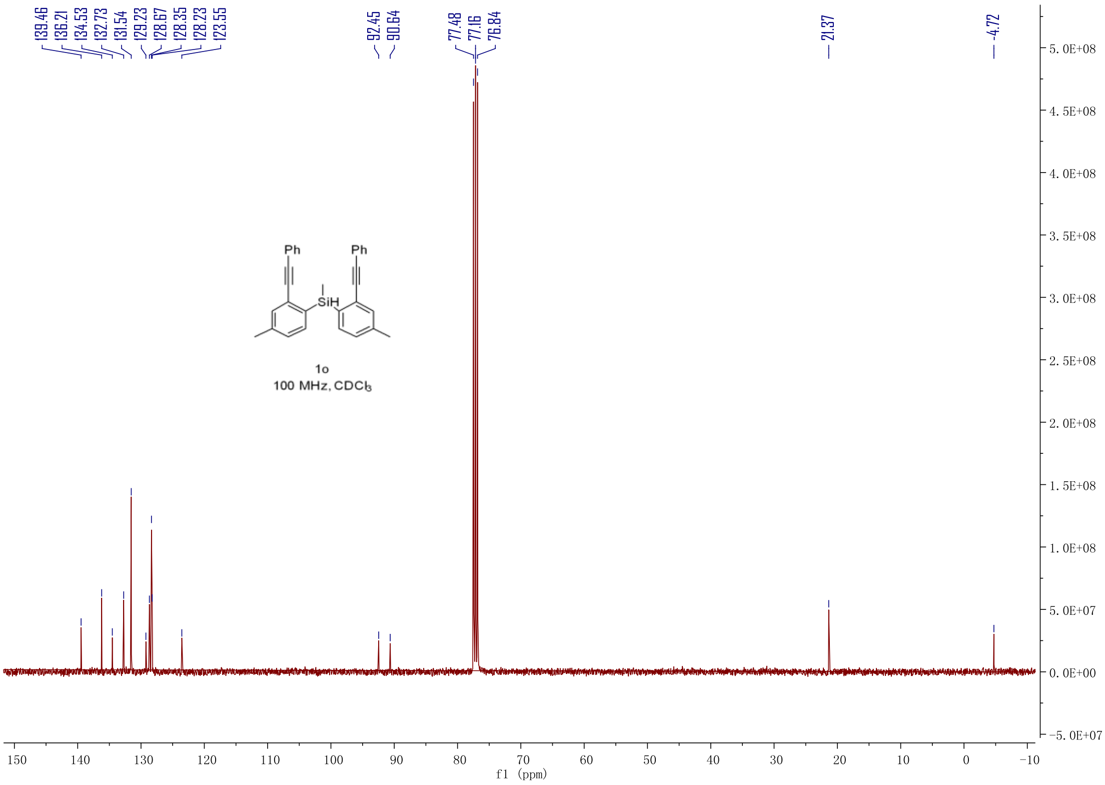

Figure S31. ^{13}C NMR (100 MHz, $CDCl_3$) spectrum of compound **1o**, related to **Scheme 2**

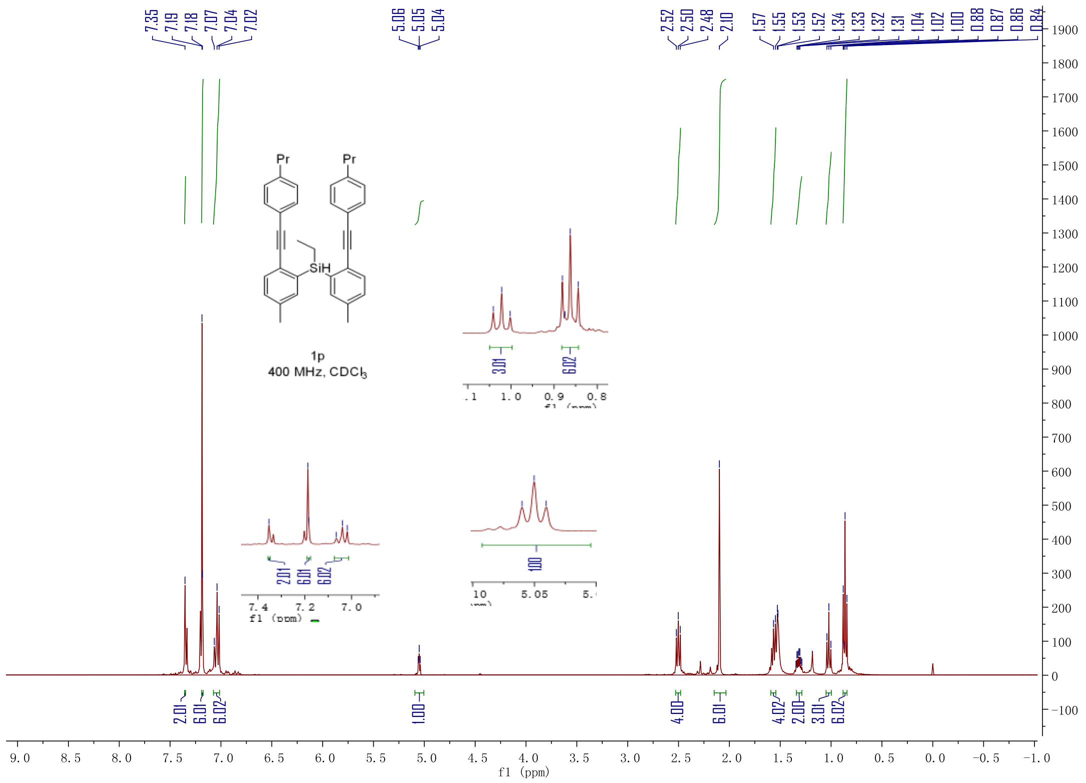

Figure S32. ^{1}H NMR (400 MHz, $CDCl_3$) spectrum of compound **1p**, related to **Scheme 2**

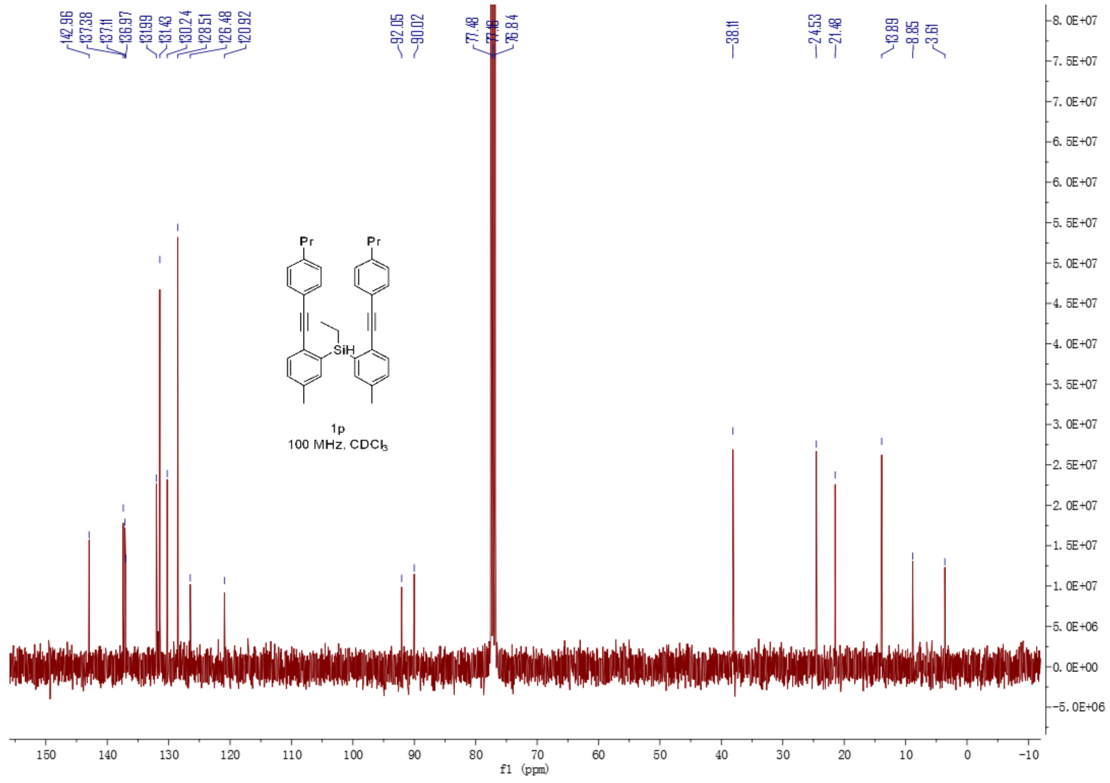

Figure S33. ^{13}C NMR (100 MHz, $CDCl_3$) spectrum of compound **1p**, related to **Scheme 2**

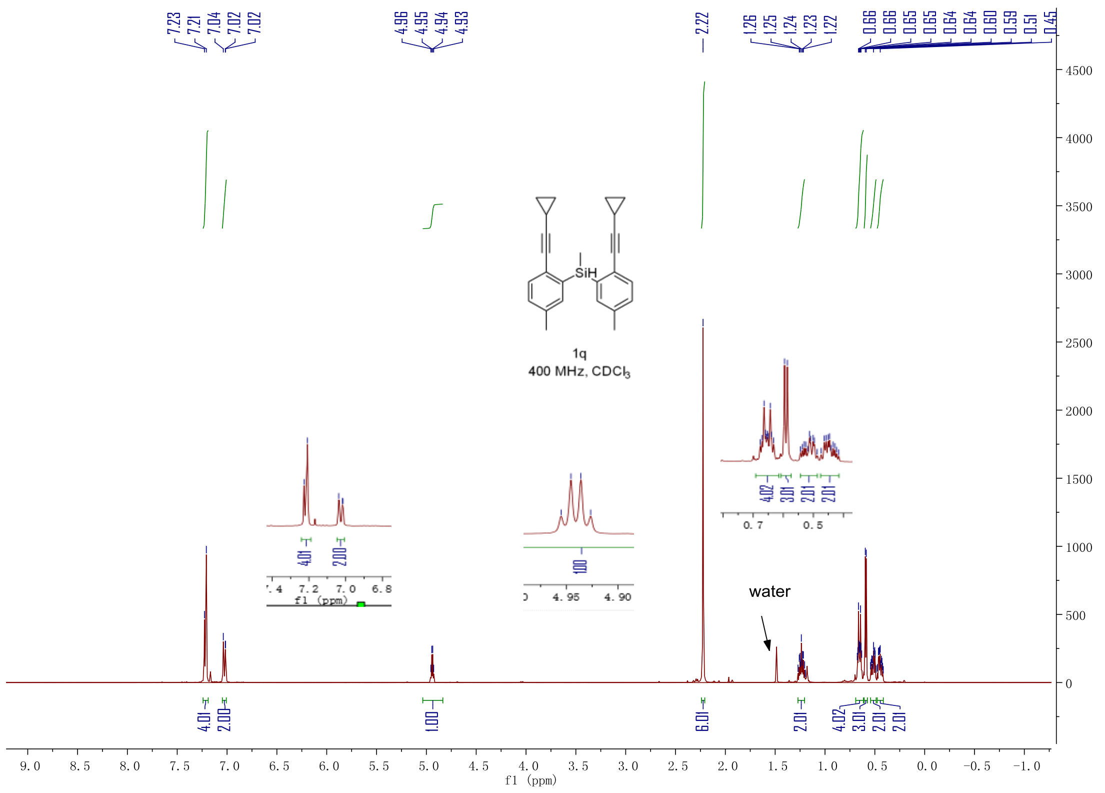

Figure S34. ^{1}H NMR (400 MHz, $CDCl_3$) spectrum of compound **1q**, related to **Scheme 2**

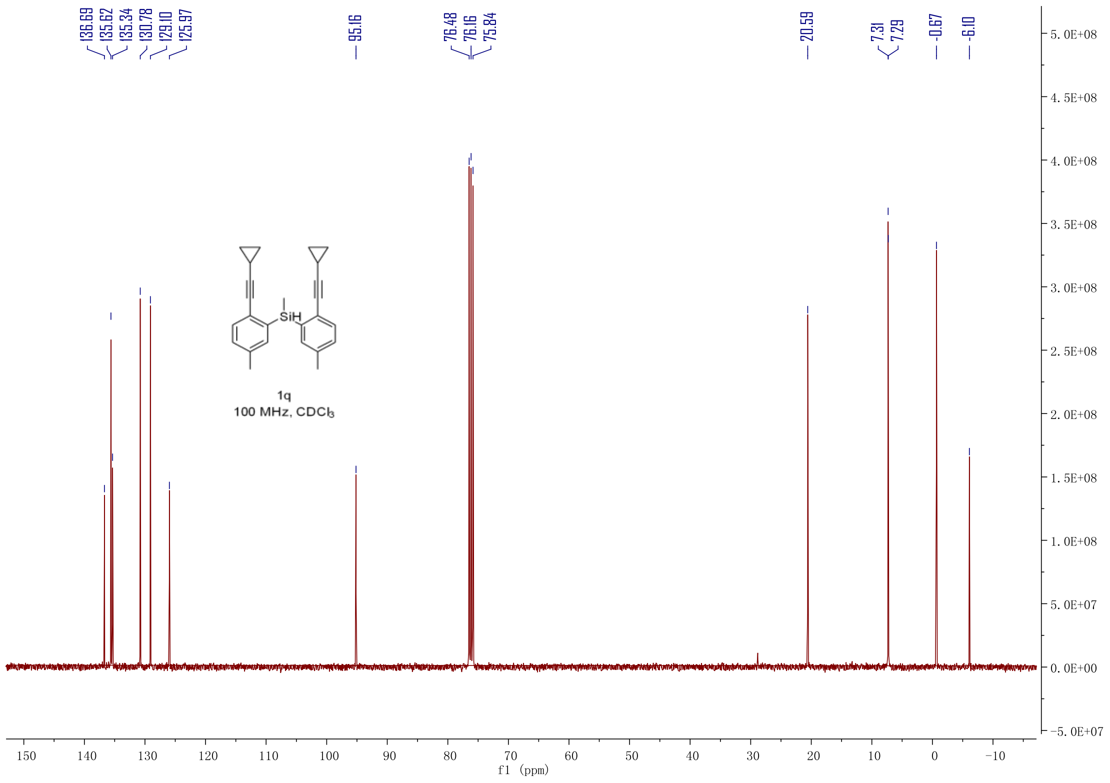

Figure S35. ^{13}C NMR (100 MHz, $CDCl_3$) spectrum of compound **1q**, related to **Scheme 2**

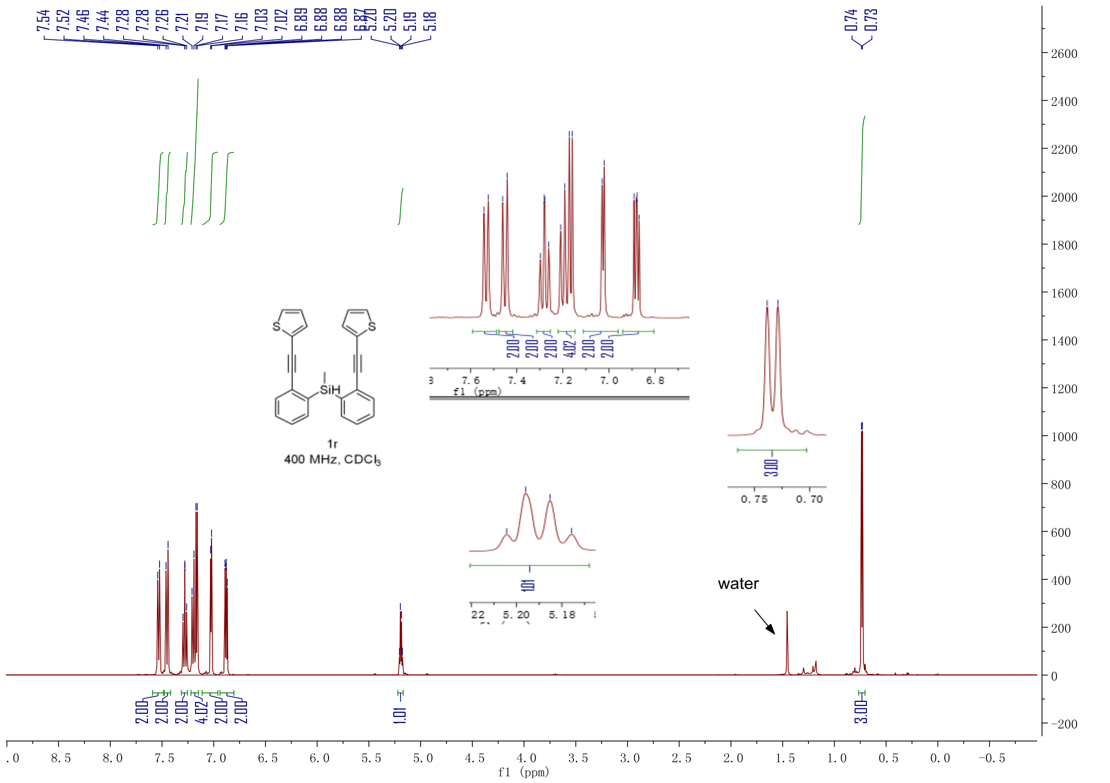

Figure S36. ^{1}H NMR (400 MHz, $CDCl_3$) spectrum of compound **1r**, related to **Scheme 2**

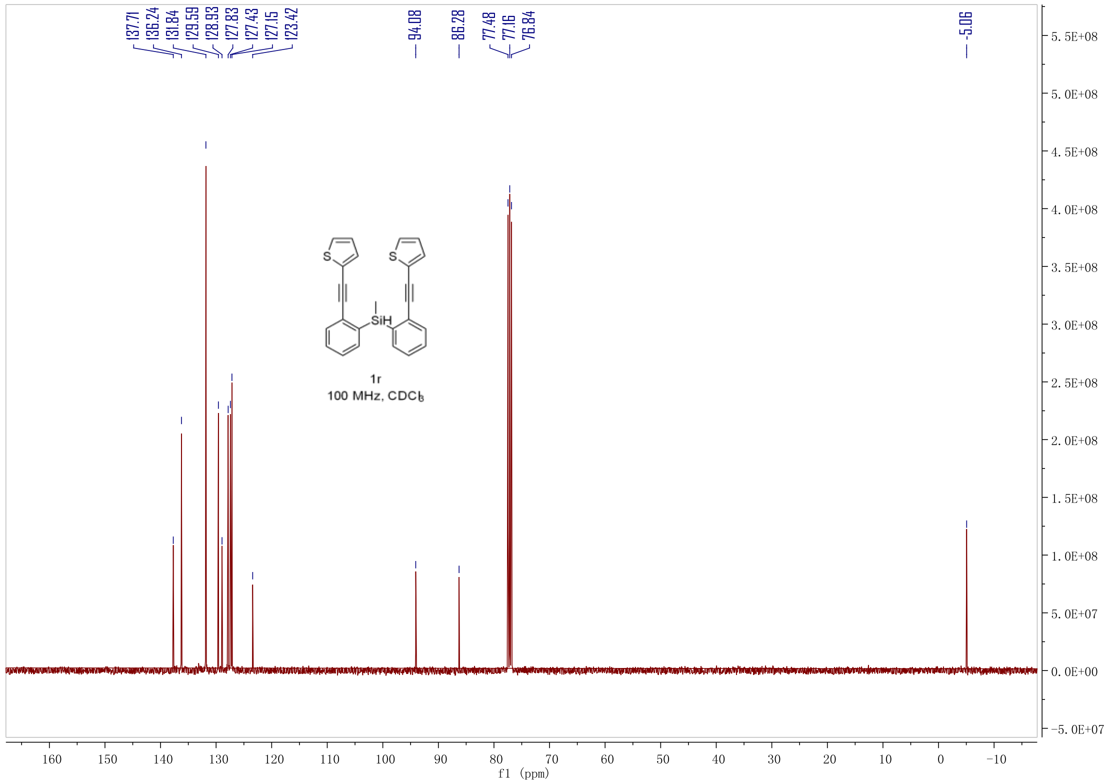

Figure S37. ^{13}C NMR (100 MHz, $CDCl_3$) spectrum of compound **1r**, related to **Scheme 2**

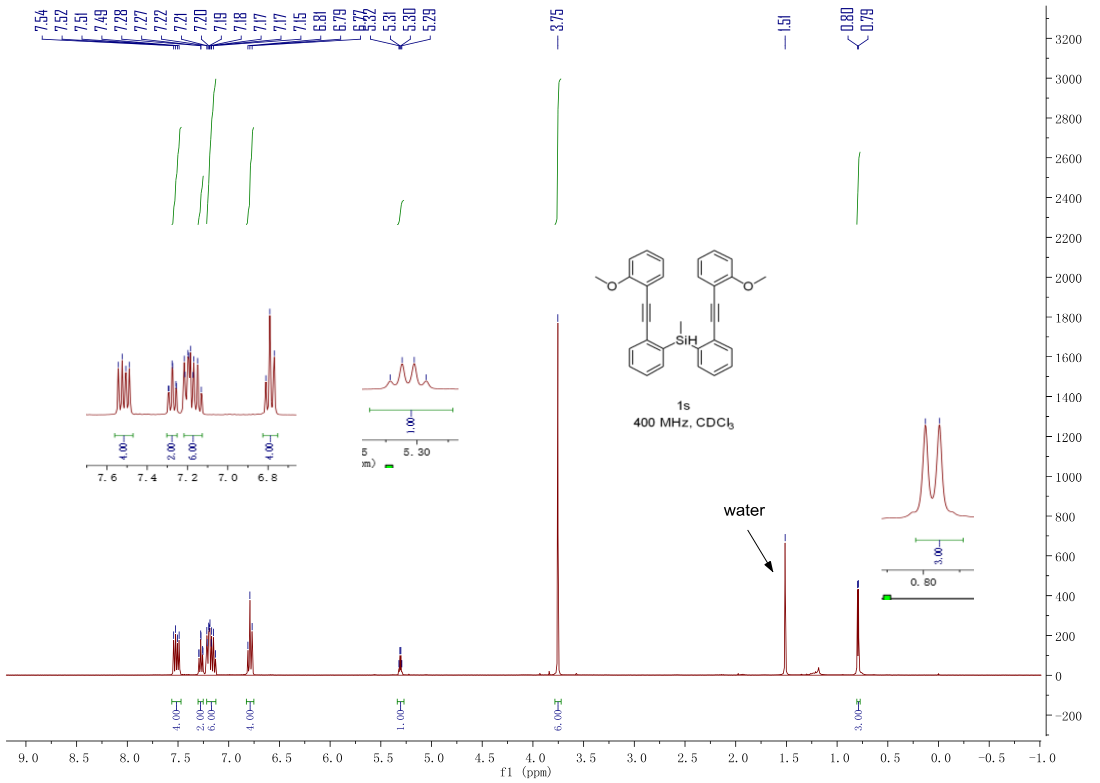

Figure S38. ^{1}H NMR (400 MHz, $CDCl_3$) spectrum of compound **1s**, related to **Scheme 2**

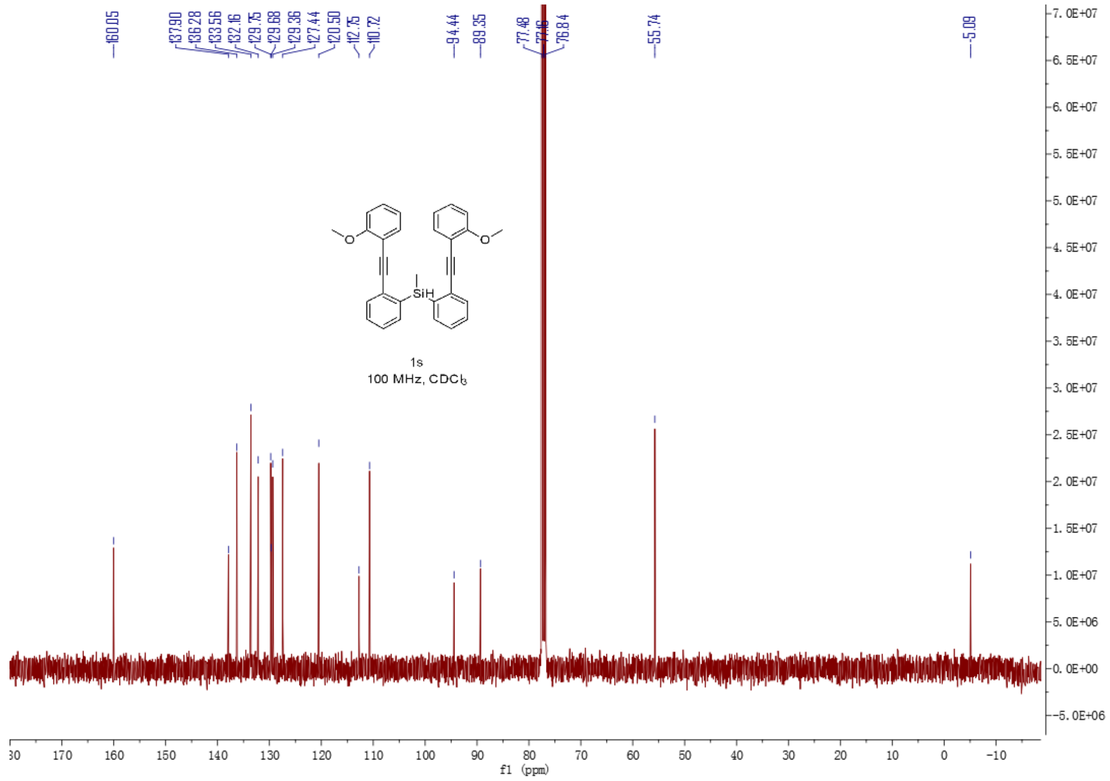

Figure S39. ^{13}C NMR (100 MHz, $CDCl_3$) spectrum of compound **1s**, related to **Scheme 2**

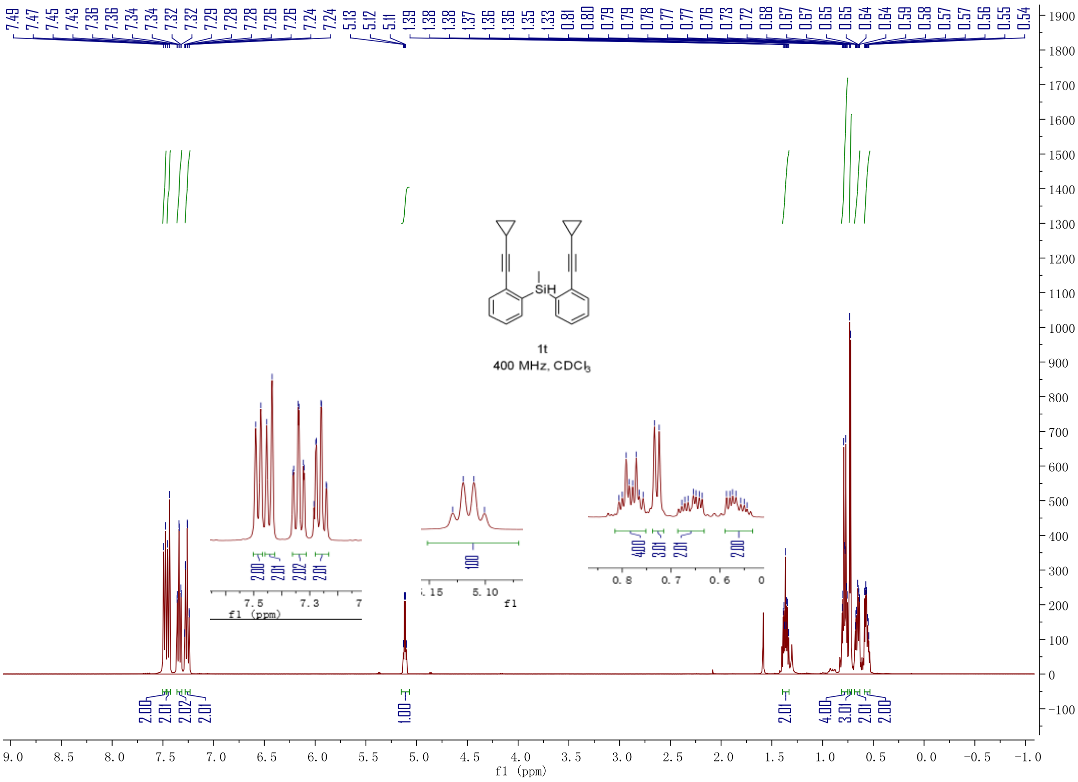

Figure S40. ^{1}H NMR (400 MHz, $CDCl_3$) spectrum of compound **1t**, related to **Scheme 2**

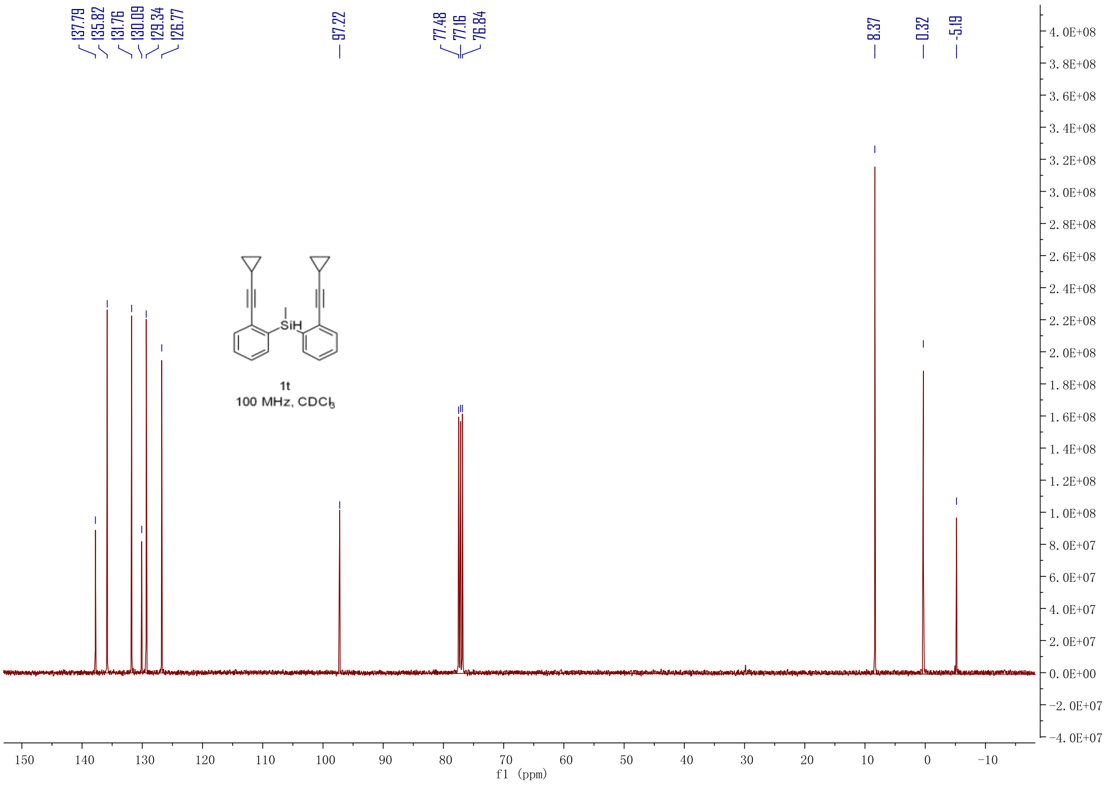

Figure S41. ^{13}C NMR (100 MHz, $CDCl_3$) spectrum of compound **1t**, related to **Scheme 2**

5.2 NMR spectra of products 2 (benzosiloles)

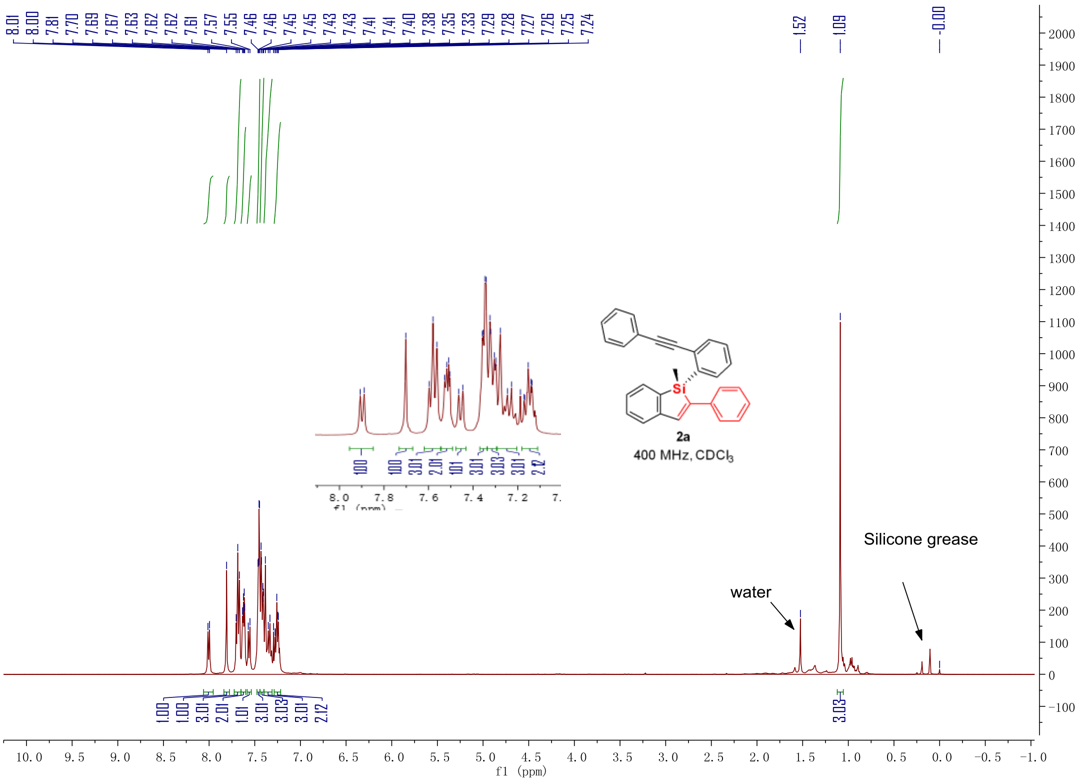

Figure S42. ^{1}H NMR (400 MHz, $CDCl_3$) spectrum of compound **2a** , related to **Scheme 2**

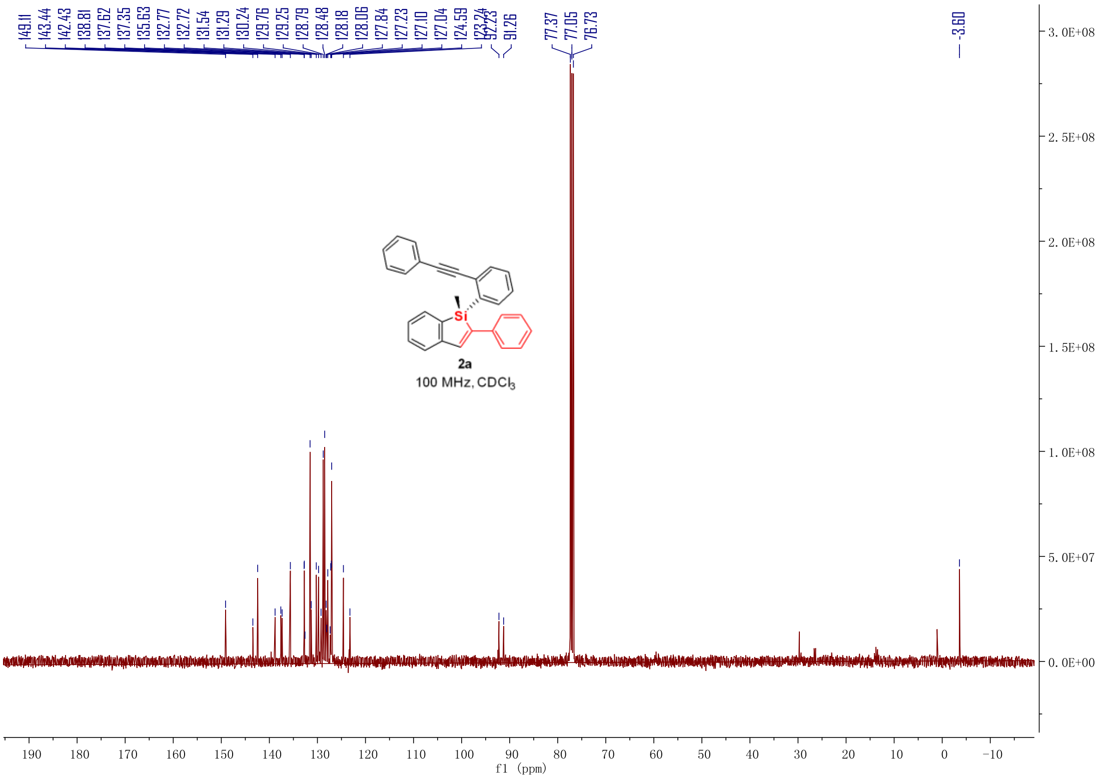

Figure S43. ^{13}C NMR (100 MHz, $CDCl_3$) spectrum of compound **2a,** related to **Scheme 2**

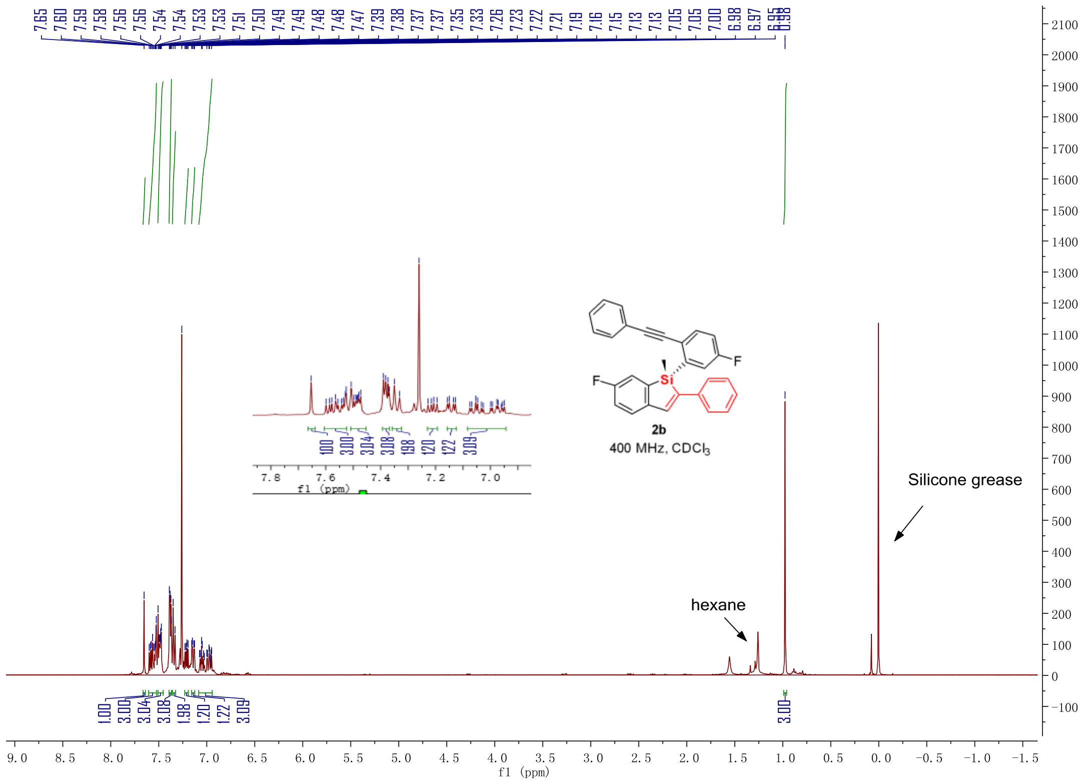

Figure S44. ^{1}H NMR (400 MHz, $CDCl_3$) spectrum of compound **2b** , related to **Scheme 2**

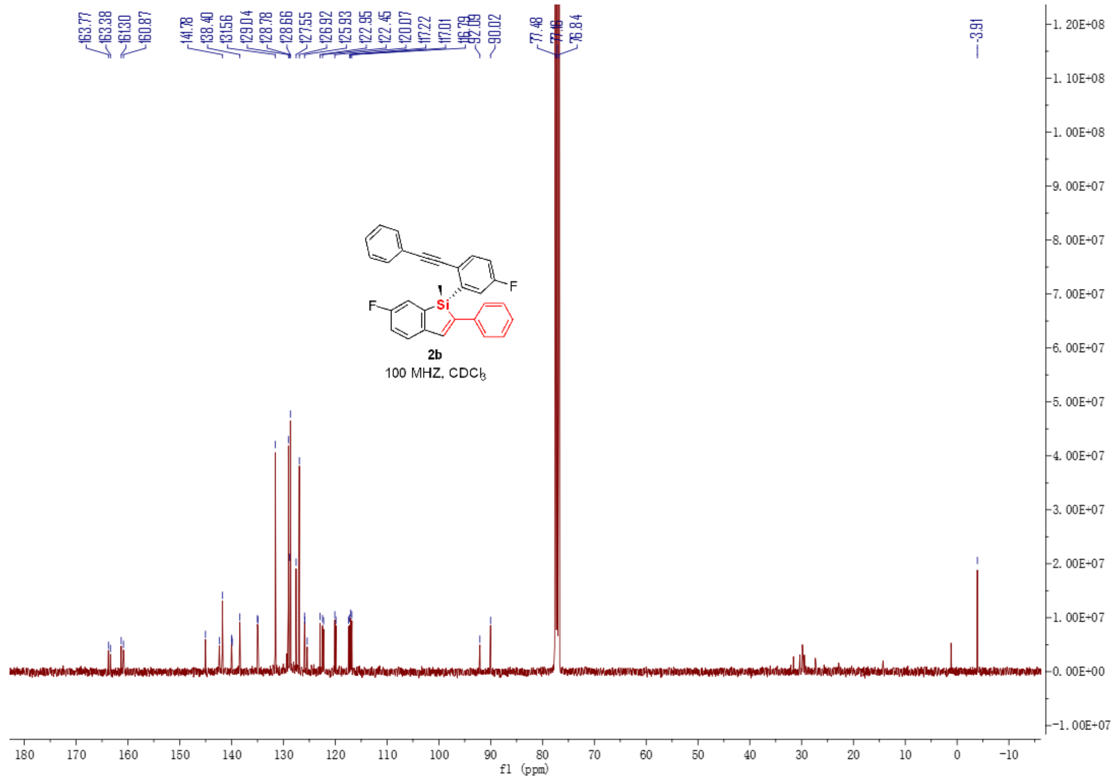

Figure S45. ^{13}C NMR (100 MHz, $CDCl_3$) spectrum of compound **2b,** related to **Scheme 2**

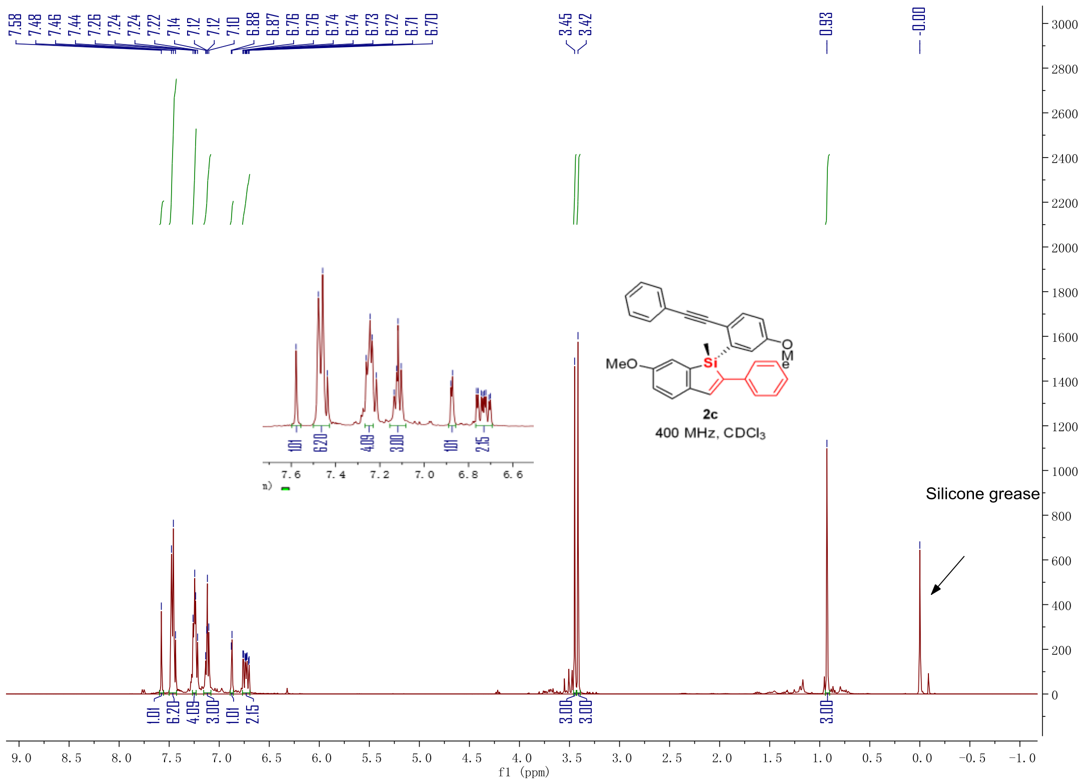

Figure S46. ^{1}H NMR (400 MHz, $CDCl_3$) spectrum of compound **2c** , related to **Scheme 2**

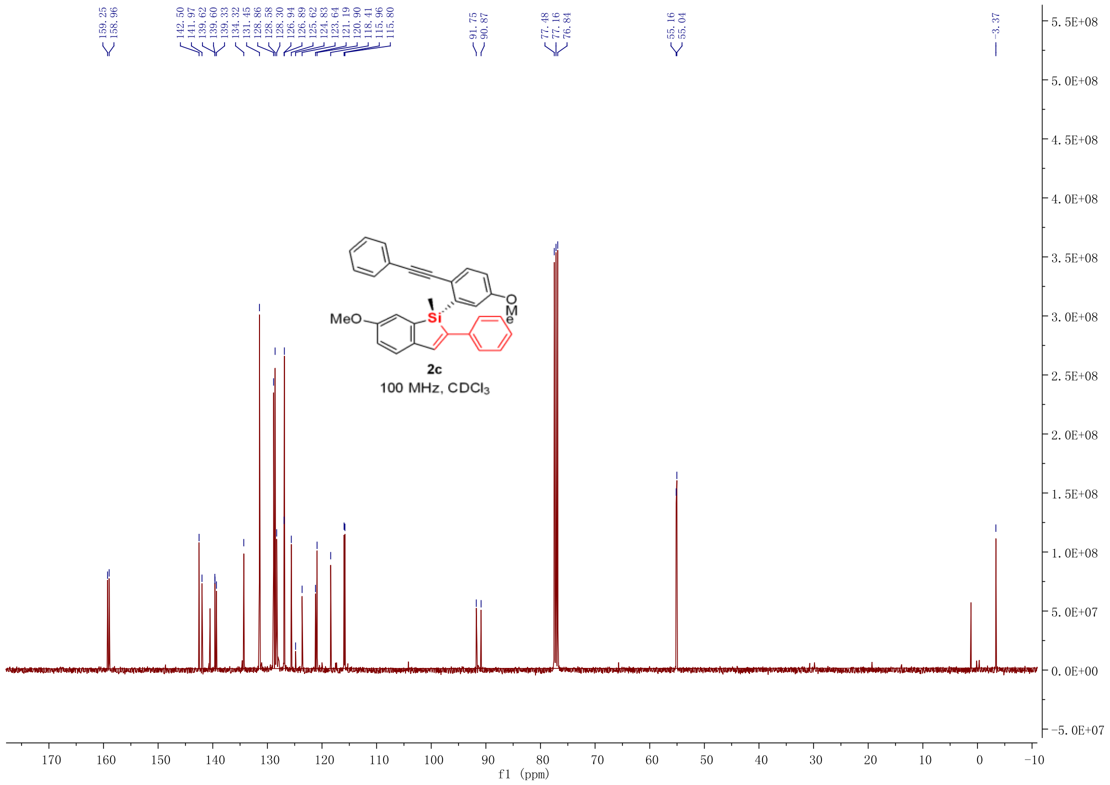

Figure S47. ^{13}C NMR (100 MHz, $CDCl_3$) spectrum of compound **2c,** related to **Scheme 2**

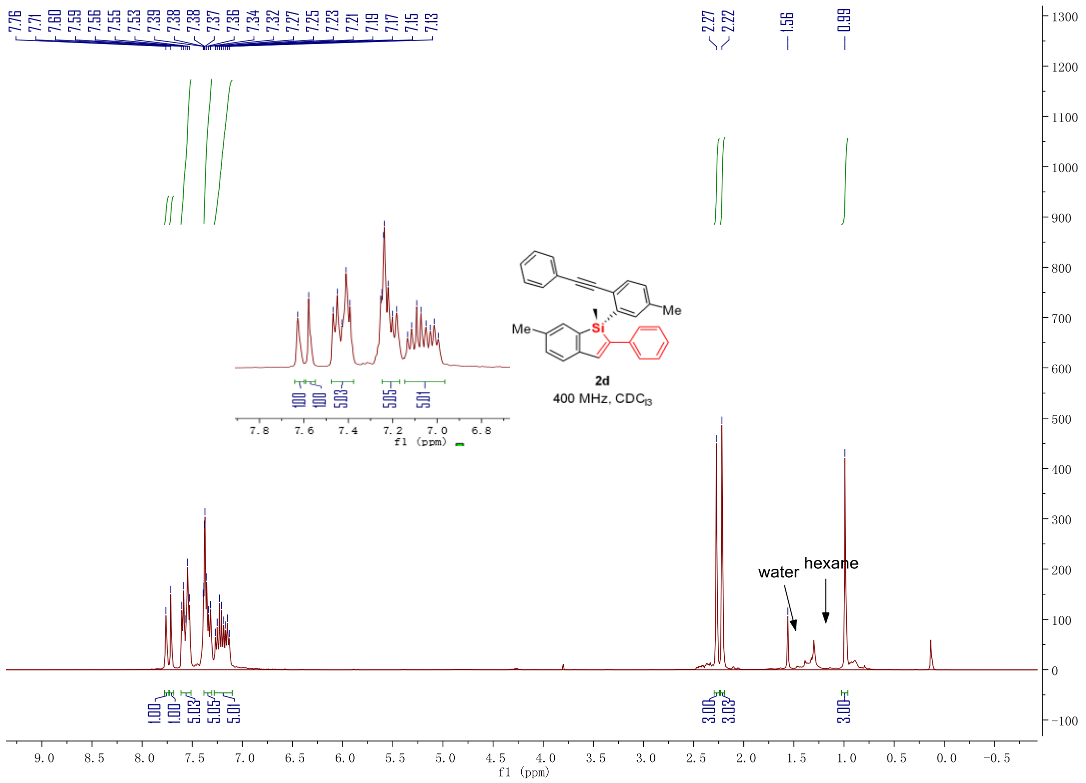

Figure S48. ^{1}H NMR (400 MHz, $CDCl_3$) spectrum of compound **2d** , related to **Scheme 2**

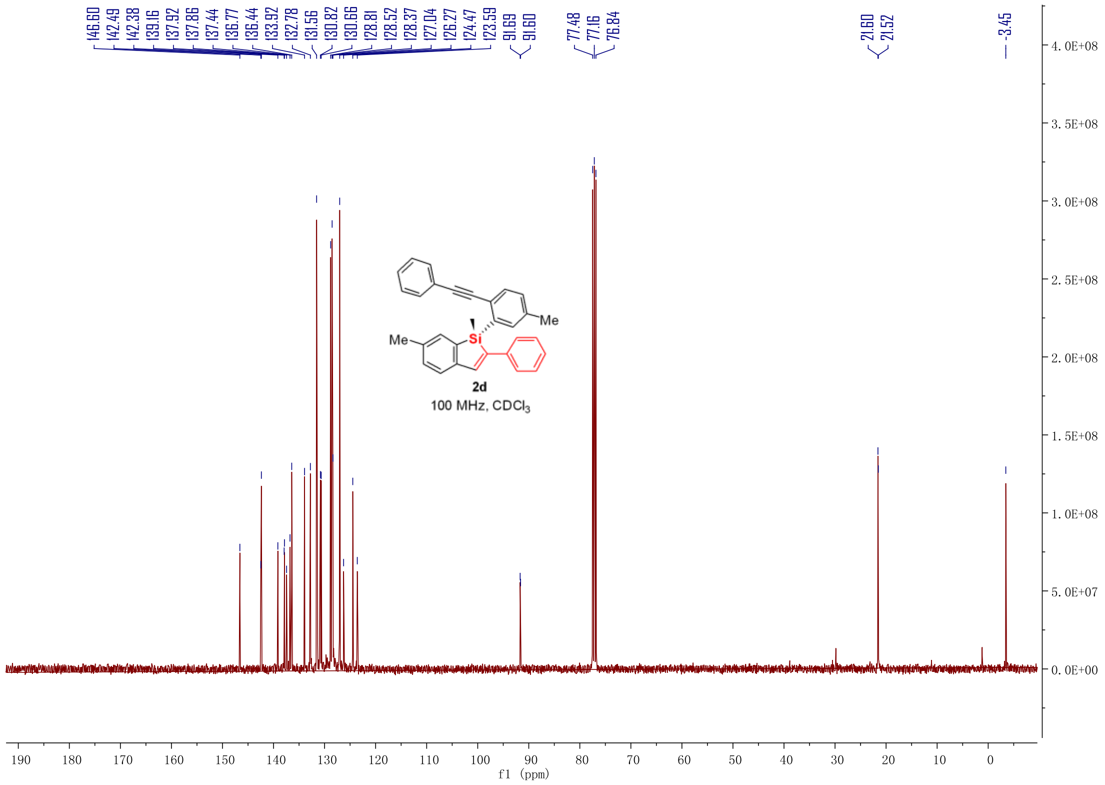

Figure S49 ^{13}C NMR (100 MHz, $CDCl_3$) spectrum of compound **2d,** related to **Scheme 2**

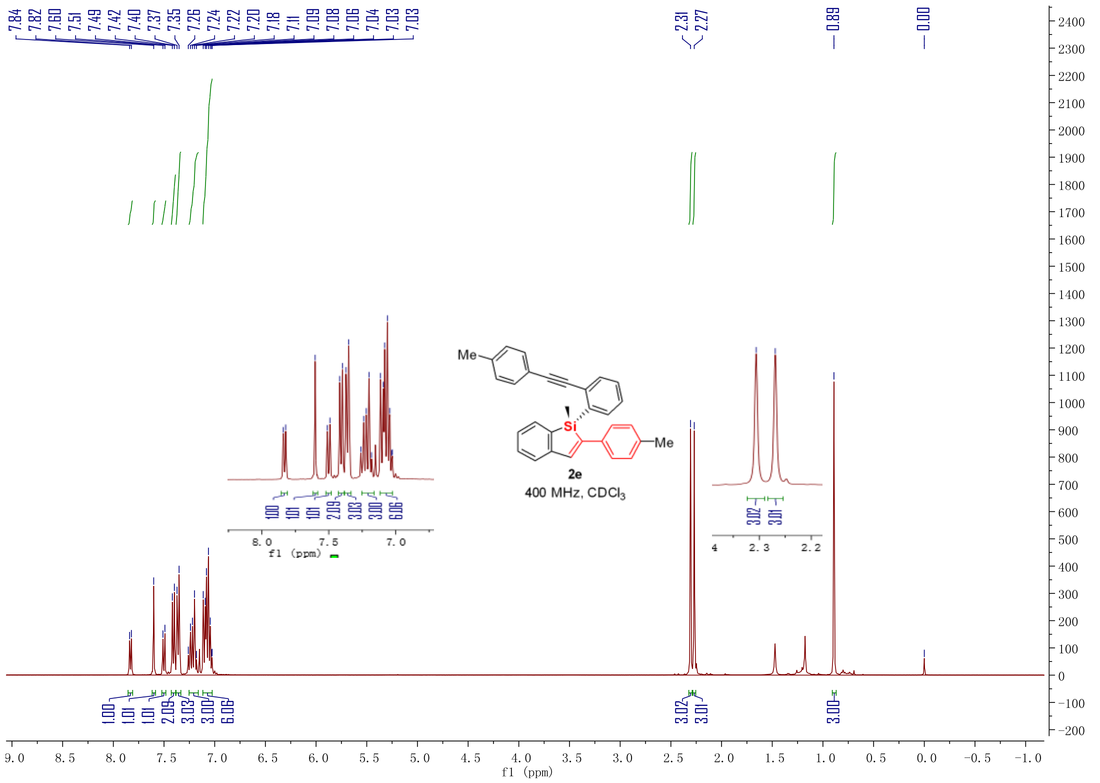

Figure S50. ^{1}H NMR (400 MHz, $CDCl_3$) spectrum of compound **2e** , related to **Scheme 2**

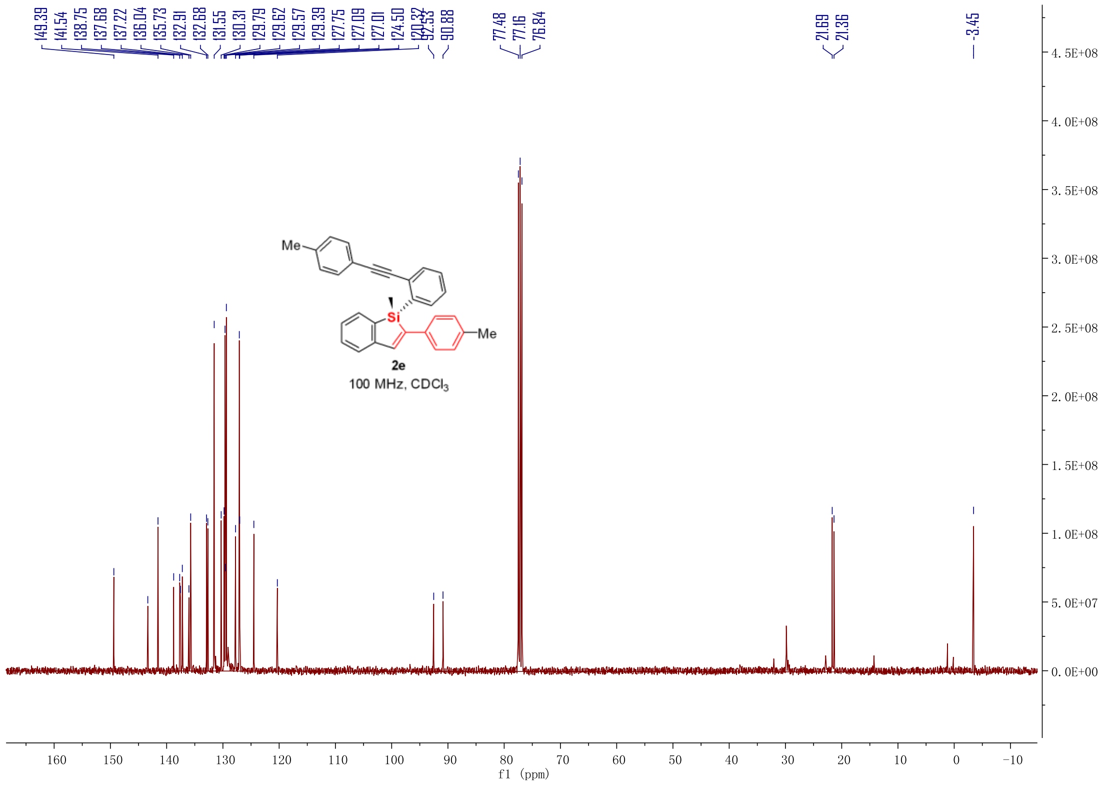

Figure S51. ^{13}C NMR (100 MHz, $CDCl_3$) spectrum of compound **2e,** related to **Scheme 2**

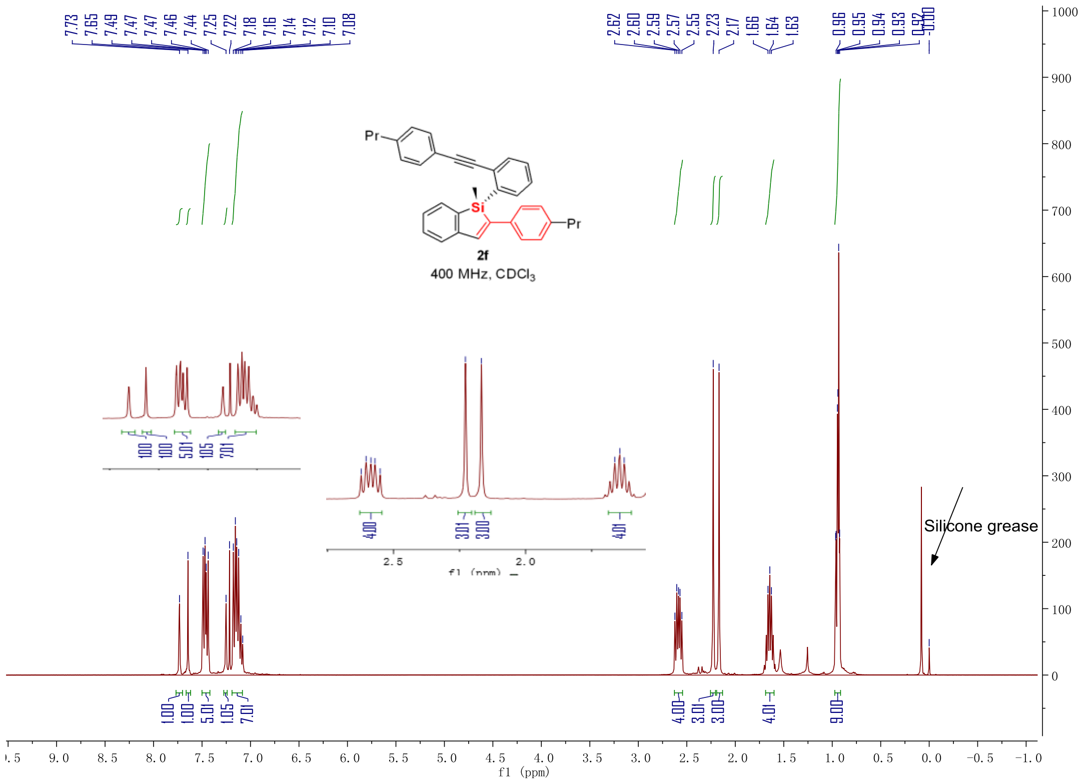

Figure S52. ^{1}H NMR (400 MHz, $CDCl_3$) spectrum of compound **2f** , related to **Scheme 2**

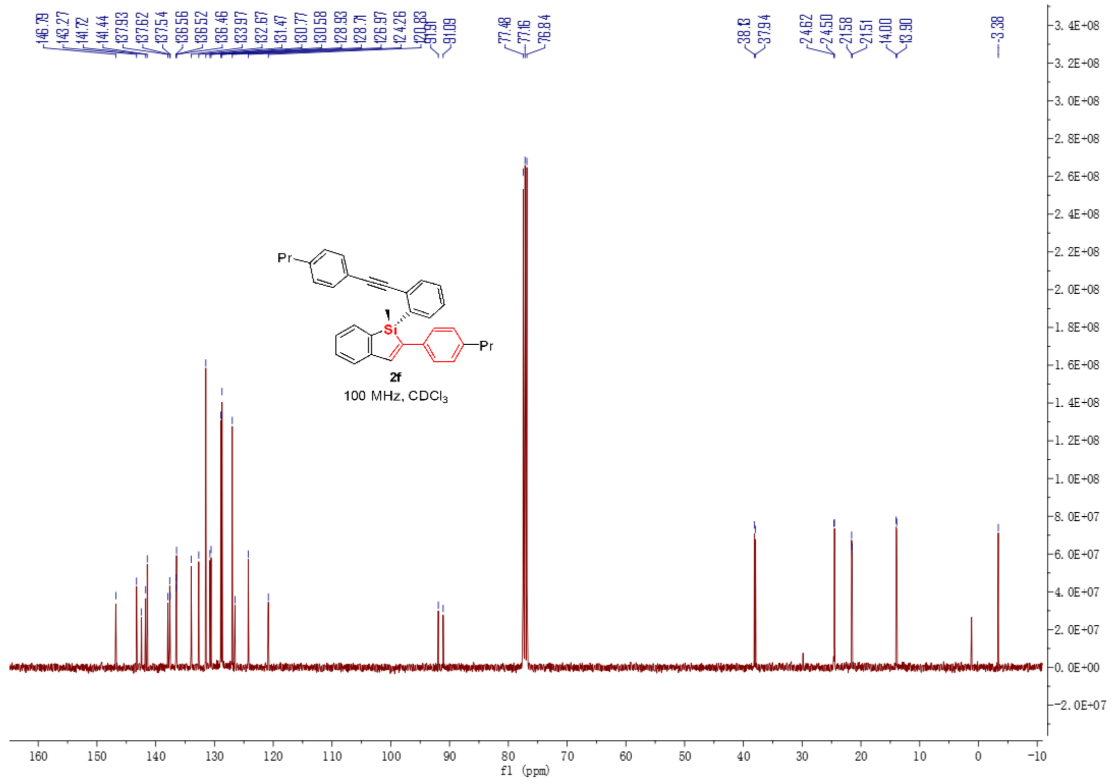

Figure S53. ^{13}C NMR (100 MHz, $CDCl_3$) spectrum of compound **2f,** related to **Scheme 2**

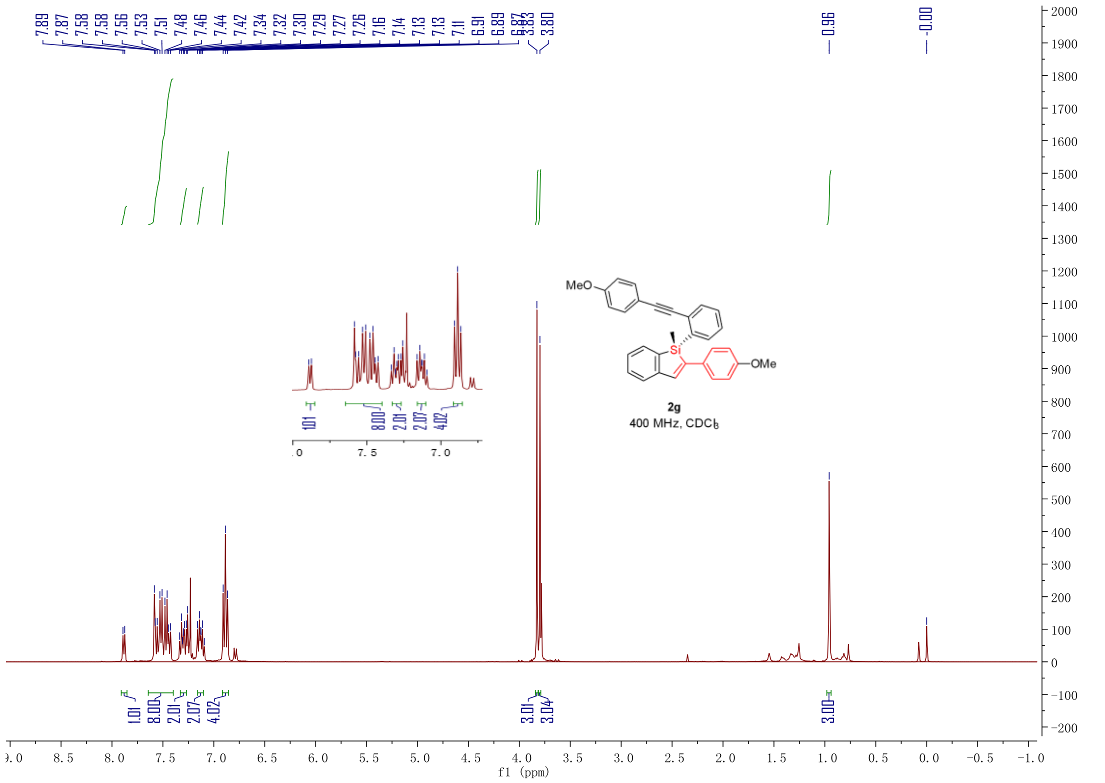

Figure S54. ^{1}H NMR (400 MHz, $CDCl_3$) spectrum of compound **2g** , related to **Scheme 2**

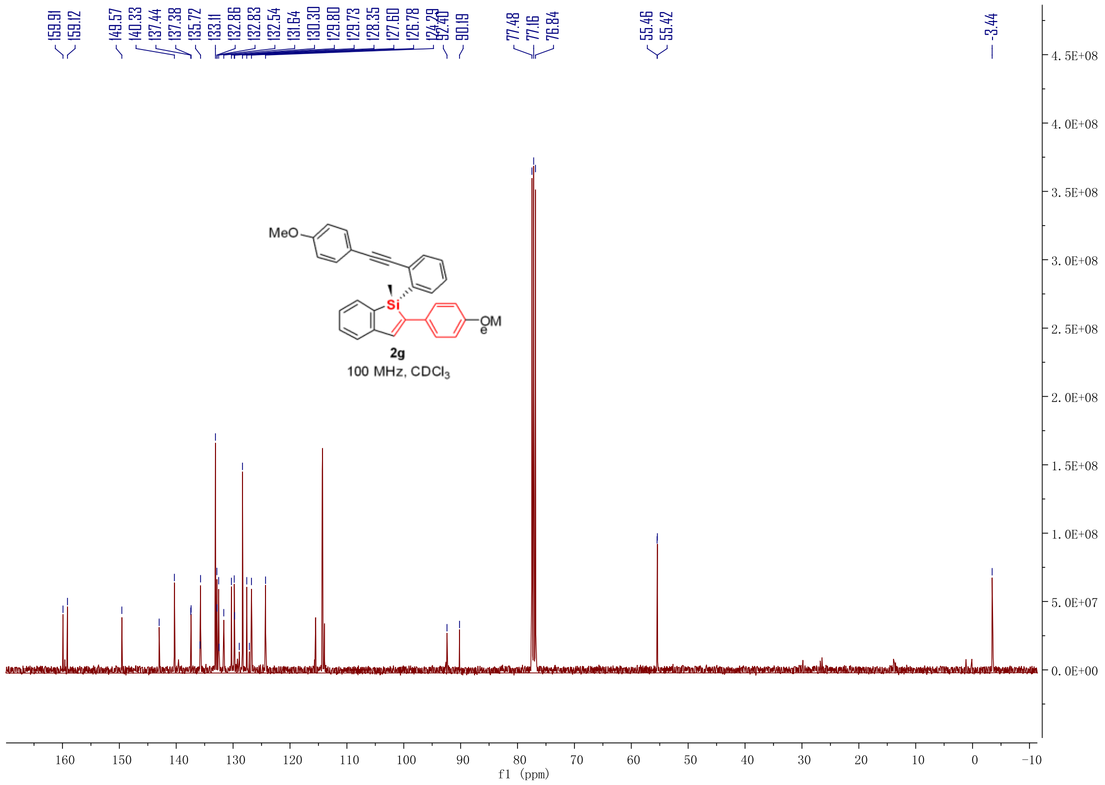

Figure S55. ^{13}C NMR (100 MHz, $CDCl_3$) spectrum of compound **2g,** related to **Scheme 2**

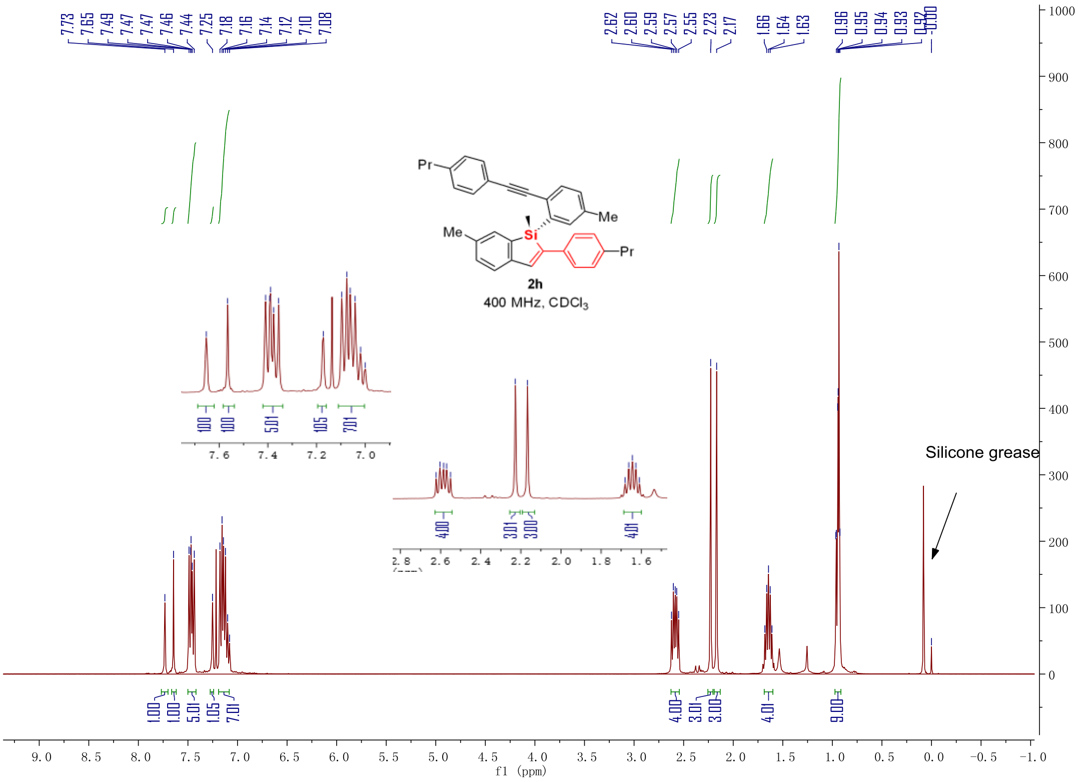

Figure S56. ^{1}H NMR (400 MHz, $CDCl_3$) spectrum of compound **2h** , related to **Scheme 2**

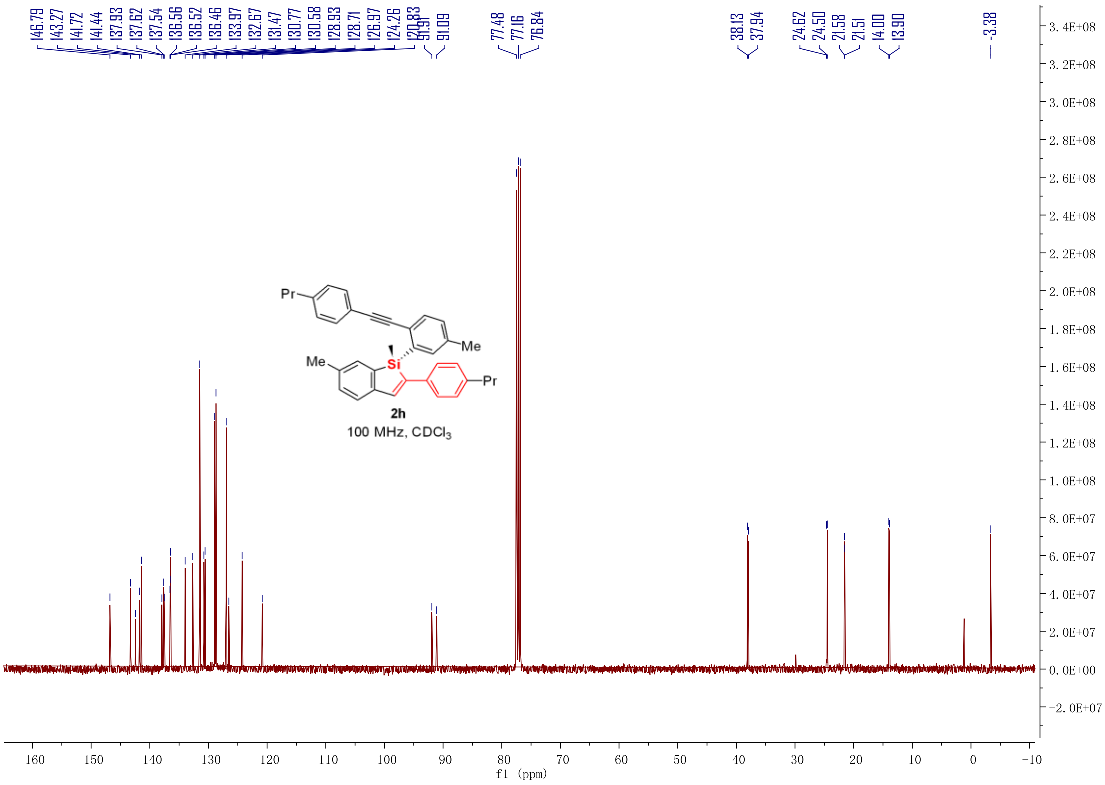

Figure S57. ^{13}C NMR (100 MHz, $CDCl_3$) spectrum of compound **2h,** related to **Scheme 2**

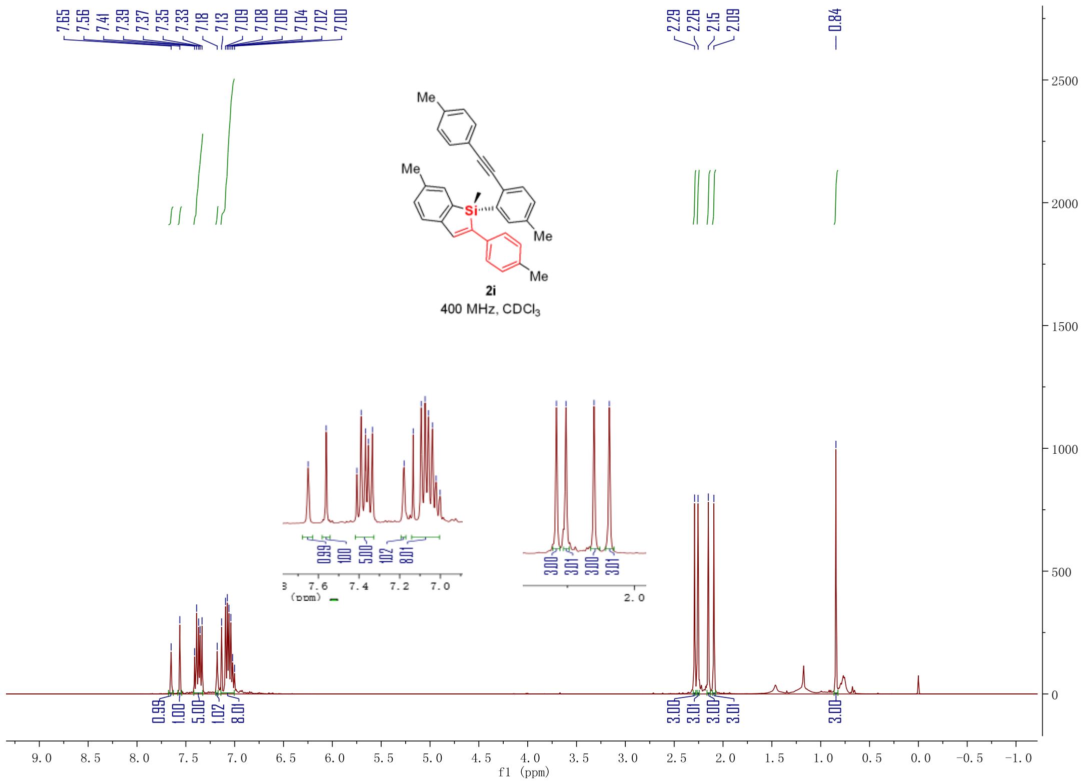

Figure S58. ^{1}H NMR (400 MHz, $CDCl_3$) spectrum of compound **2i** , related to **Scheme 2**

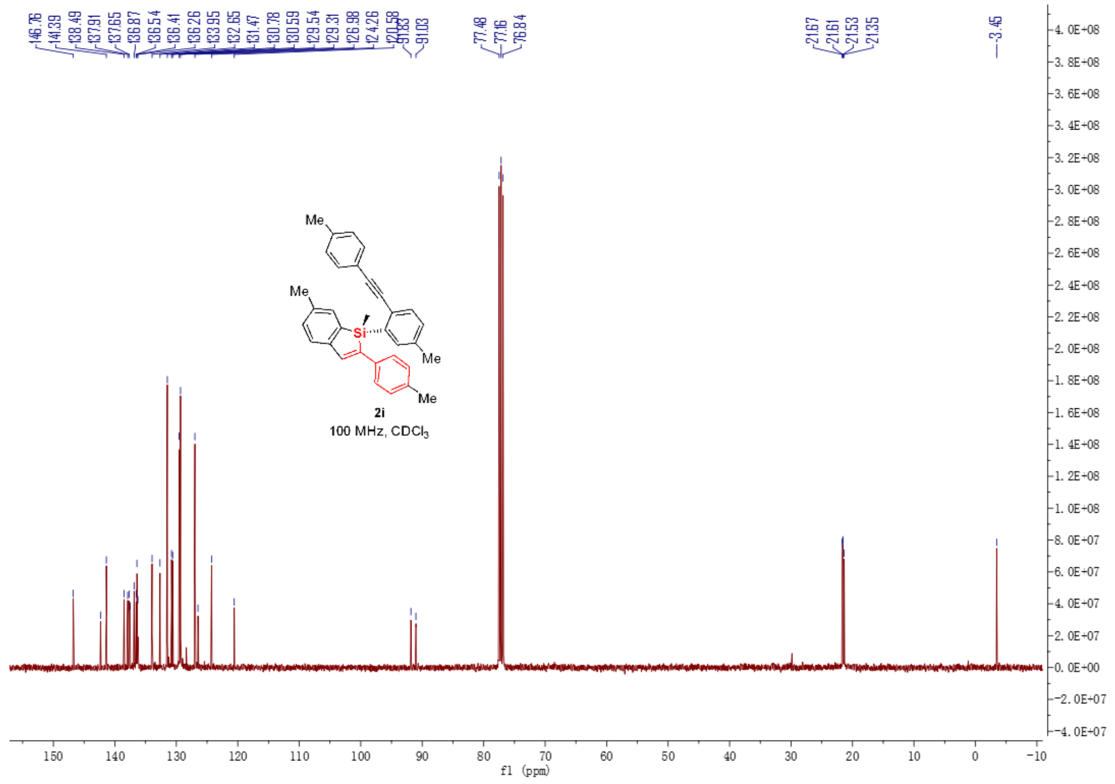

Figure S59. ^{13}C NMR (100 MHz, $CDCl_3$) spectrum of compound **2i,** related to **Scheme 2**

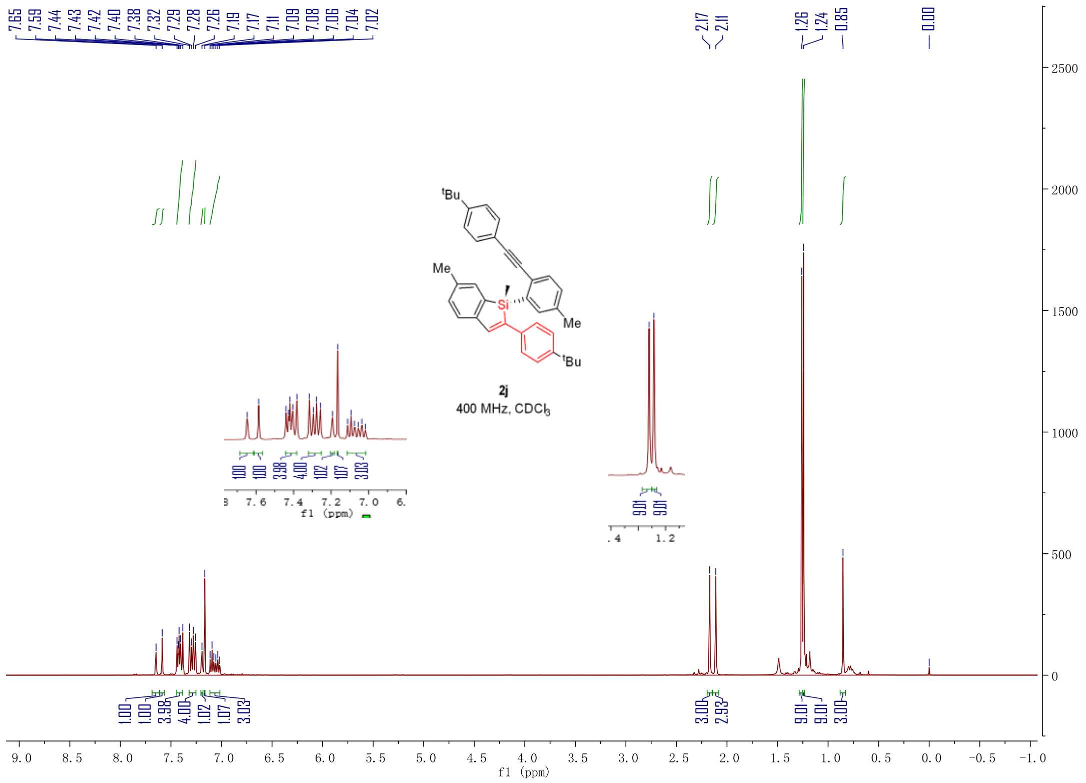

Figure S60. ^{1}H NMR (400 MHz, $CDCl_3$) spectrum of compound **2j**, related to **Scheme 2**

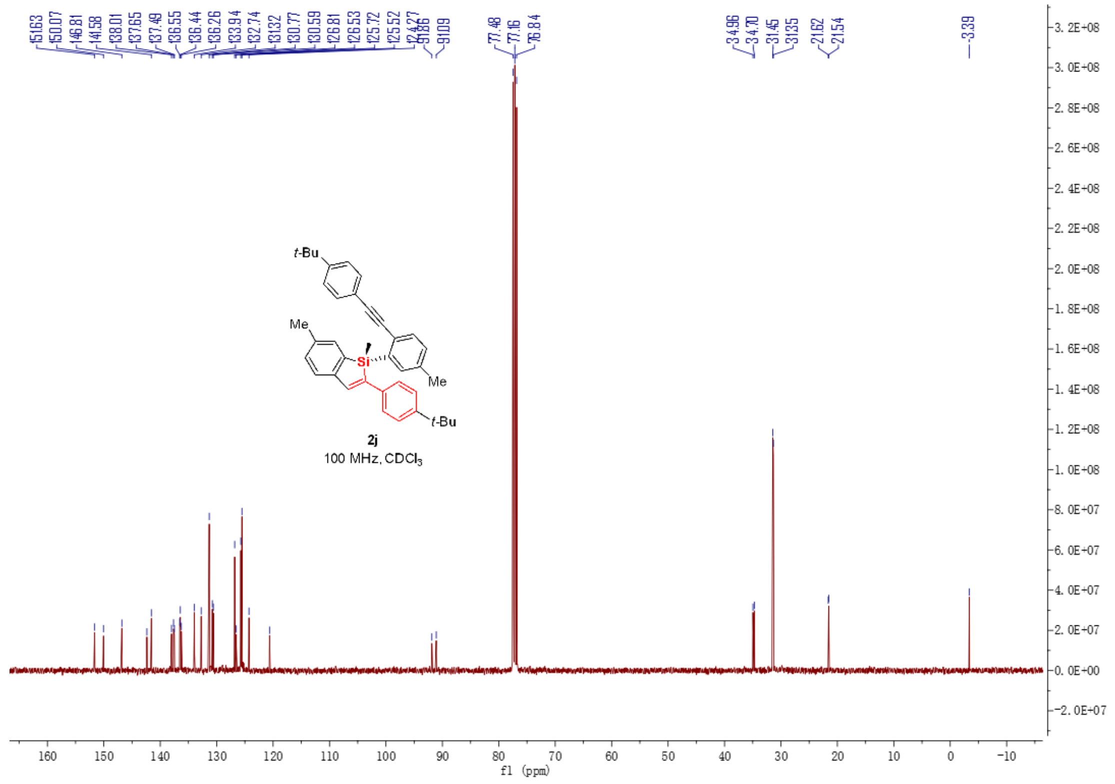

Figure S61. ^{13}C NMR (100 MHz, $CDCl_3$) spectrum of compound **2j,** related to **Scheme 2**

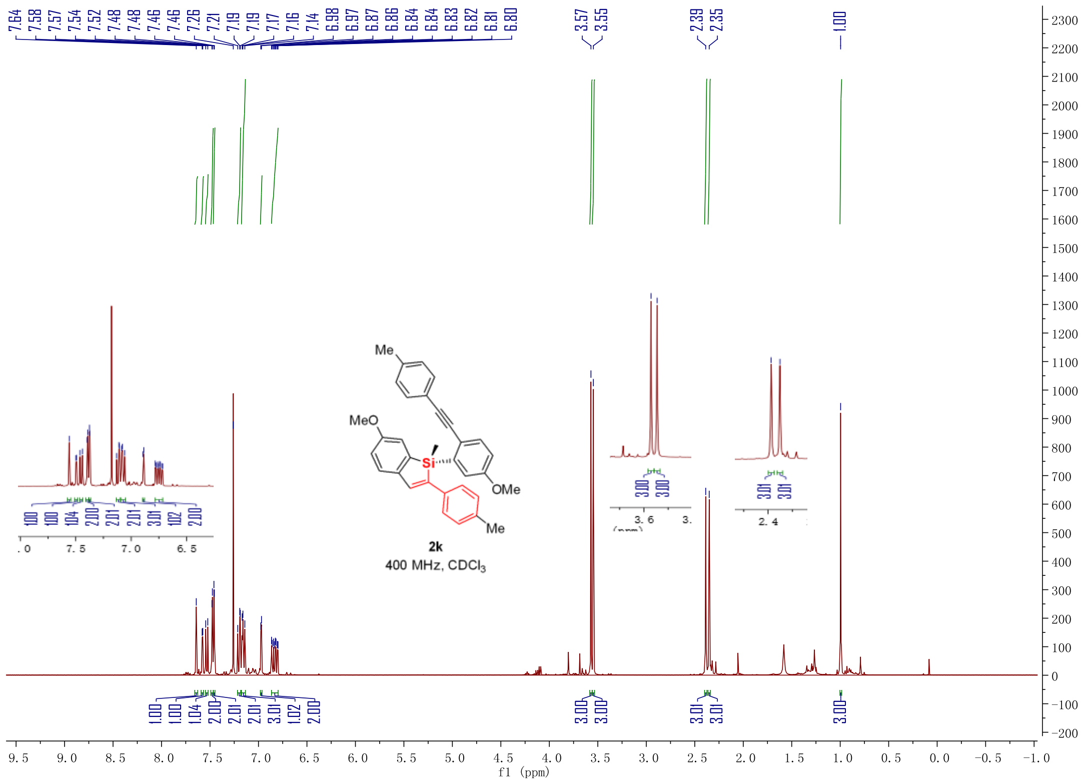

Figure S62. ^{1}H NMR (400 MHz, $CDCl_3$) spectrum of compound **2k** , related to **Scheme 2**

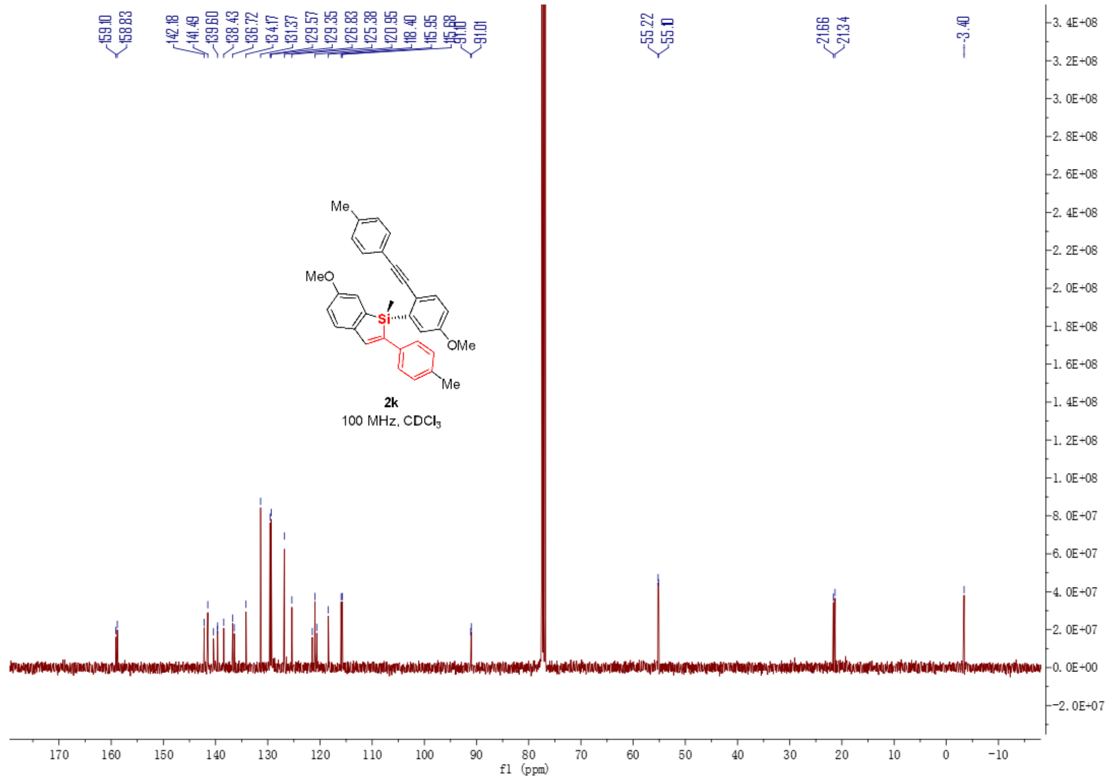

Figure S63. ^{13}C NMR (100 MHz, $CDCl_3$) spectrum of compound **2k,** related to **Scheme 2**

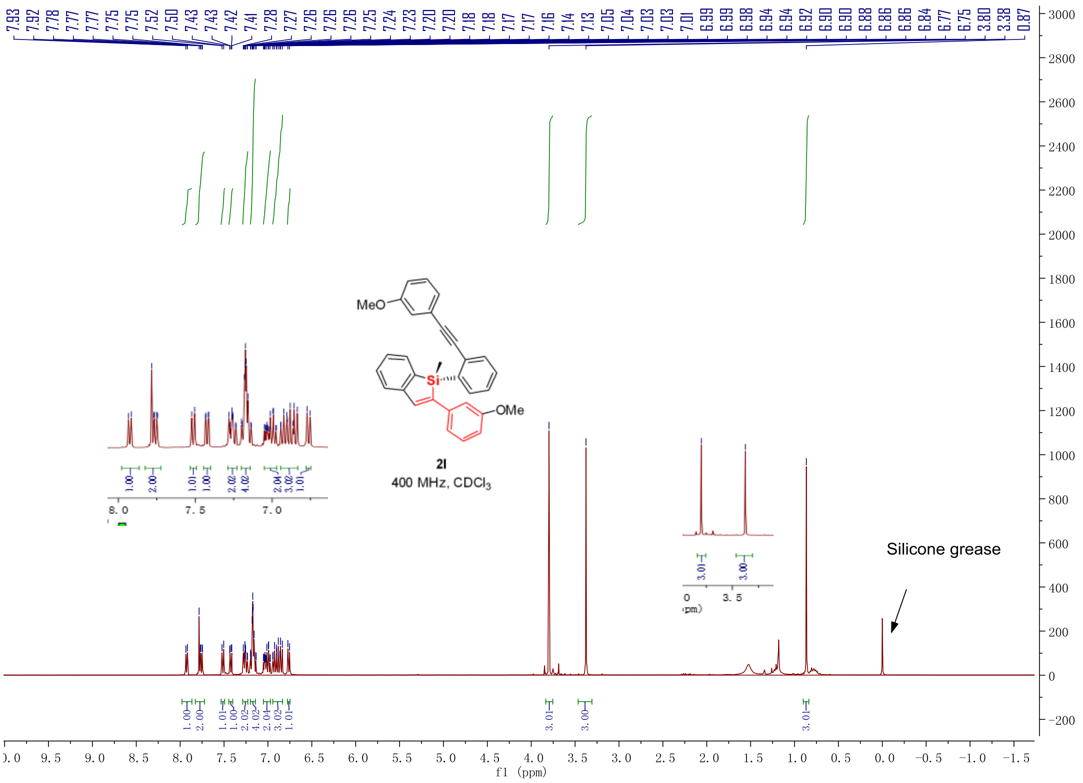

Figure S64. ^{1}H NMR (400 MHz, $CDCl_3$) spectrum of compound **2l** , related to **Scheme 2**

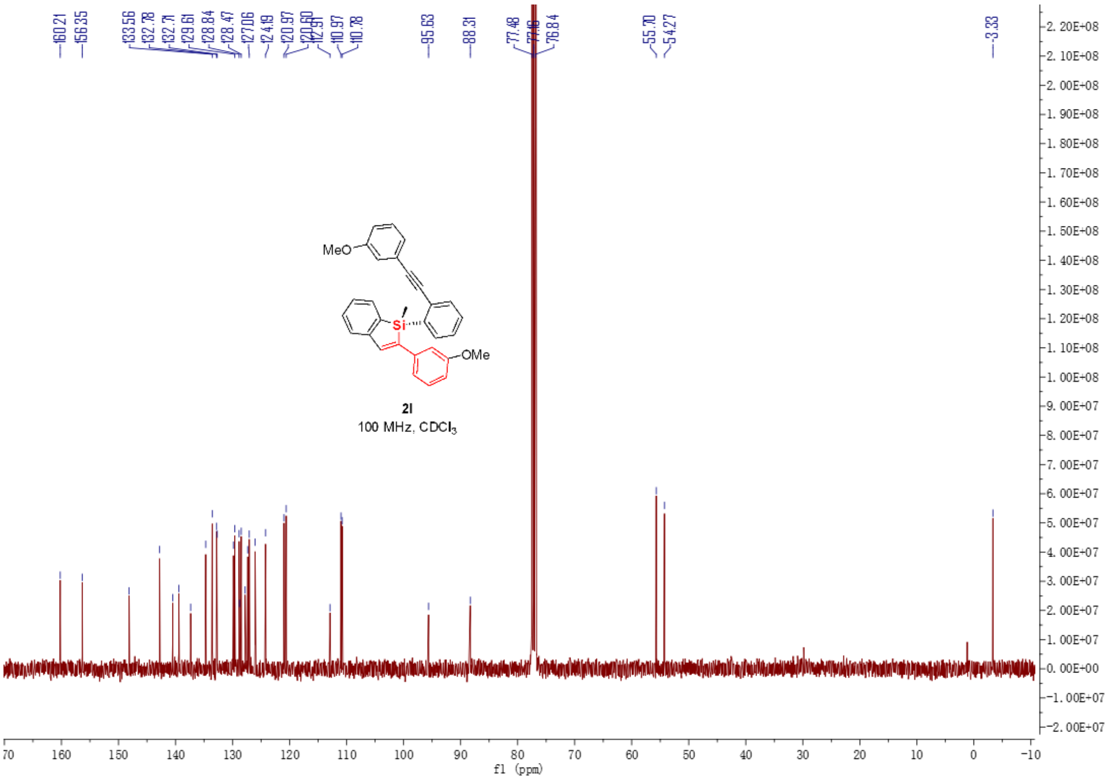

Figure S65. ^{13}C NMR (100 MHz, $CDCl_3$) spectrum of compound **2l,** related to **Scheme 2**

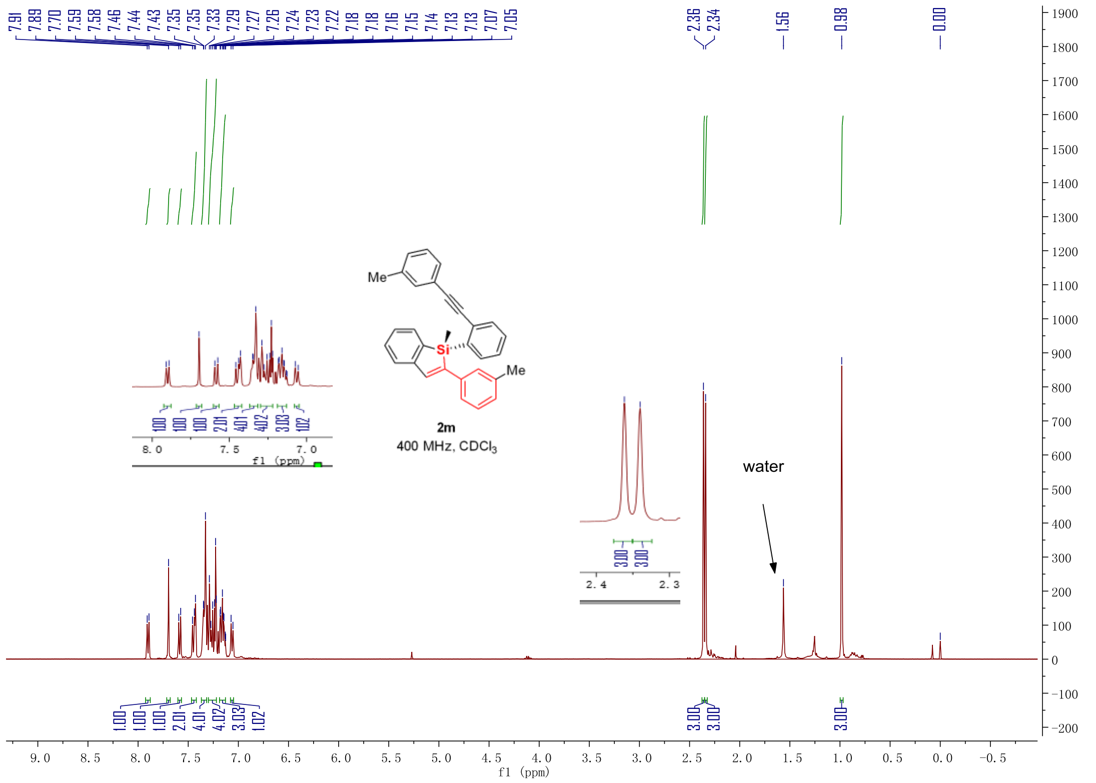

Figure S66. ^{1}H NMR (400 MHz, $CDCl_3$) spectrum of compound **2m** , related to Scheme 2

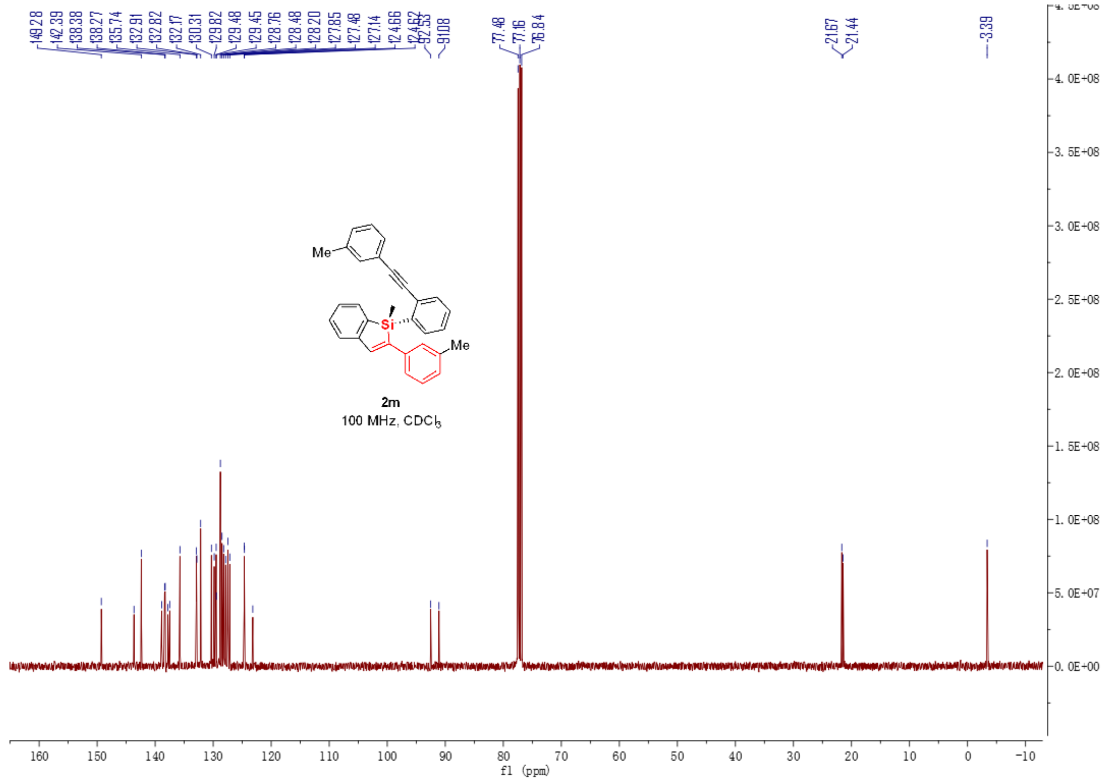

Figure S67. ^{13}C NMR (100 MHz, $CDCl_3$) spectrum of compound **2m,** related to **Scheme 2**

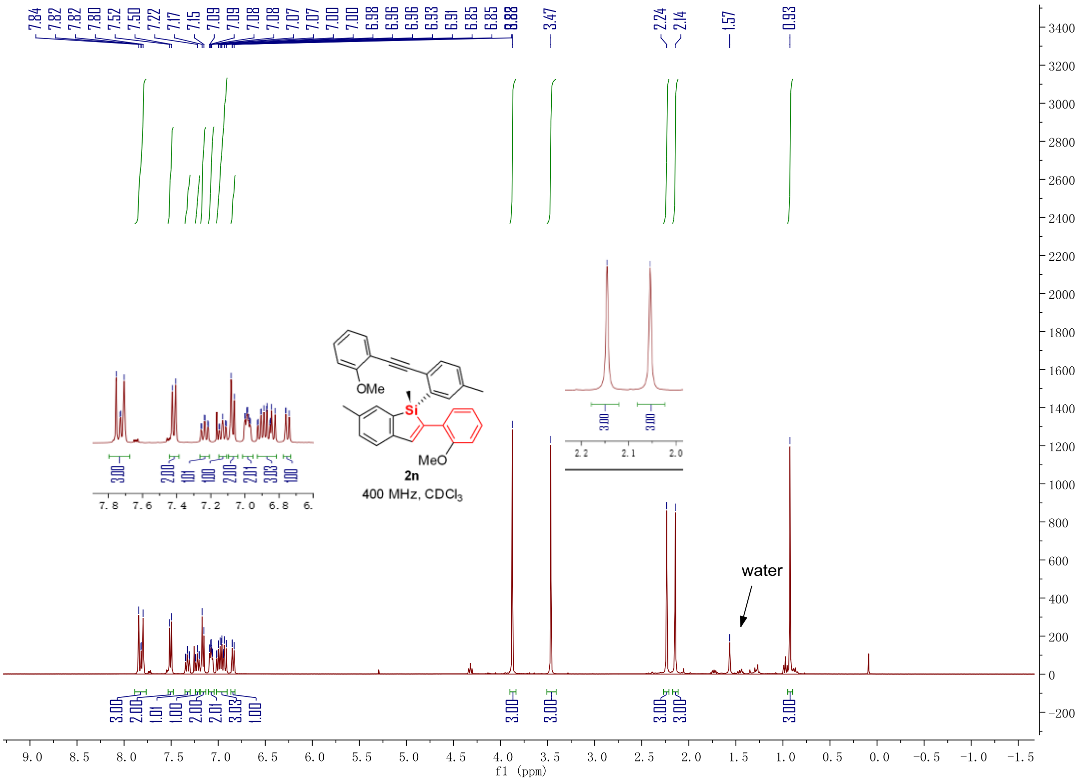

Figure S68. ^{1}H NMR (400 MHz, $CDCl_3$) spectrum of compound **2n** , related to **Scheme 2**

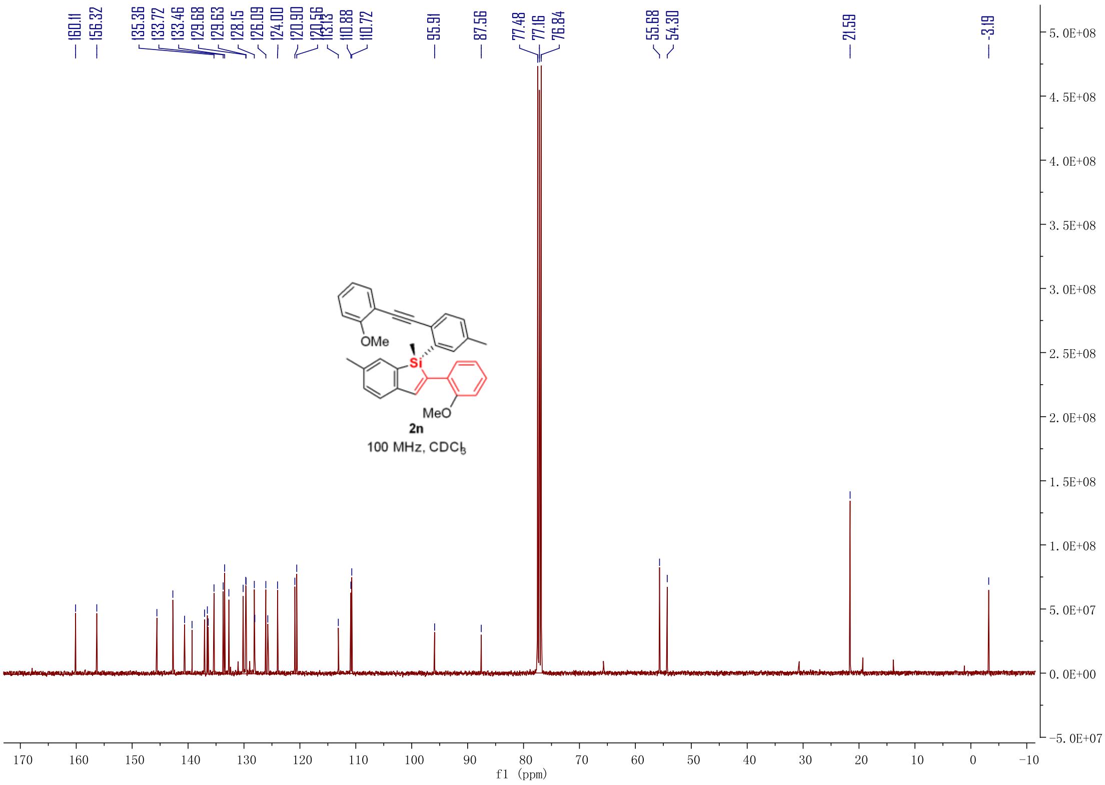

Figure S69. ^{13}C NMR (100 MHz, $CDCl_3$) spectrum of compound **2n,** related to **Scheme 2**

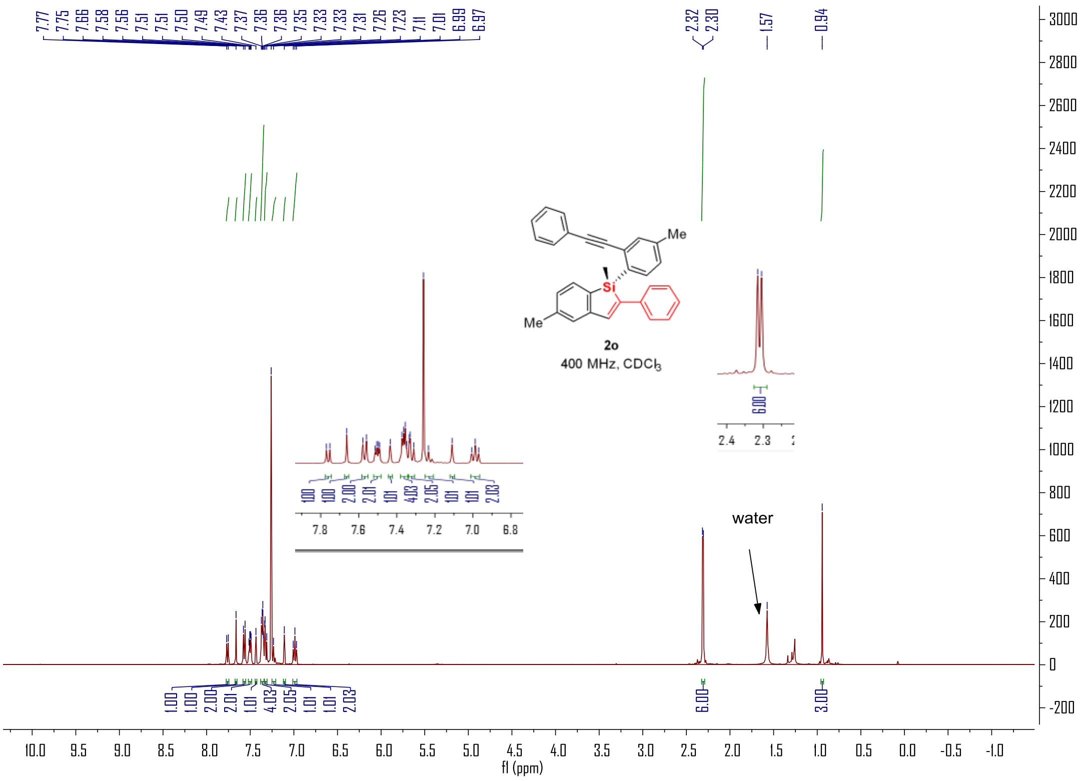

Figure S70. ^{1}H NMR (400 MHz, $CDCl_3$) spectrum of compound **2o** , related to **Scheme 2**

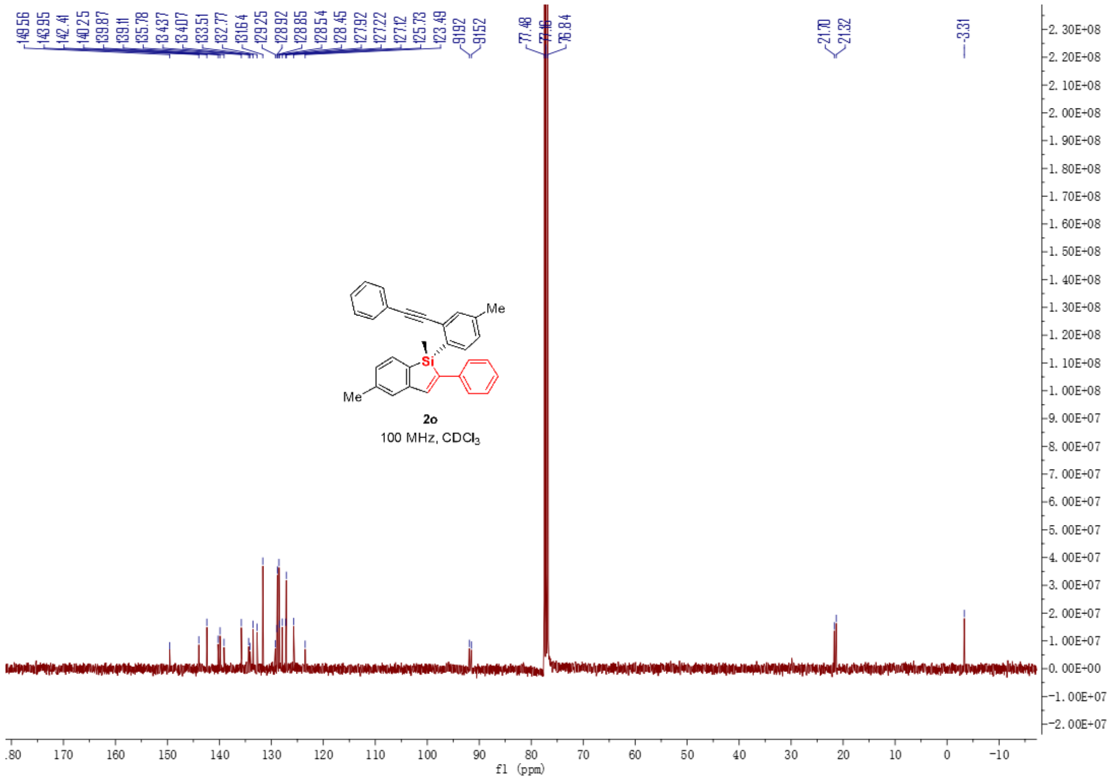

Figure S71. ^{13}C NMR (100 MHz, $CDCl_3$) spectrum of compound **2o,** related to **Scheme 2**

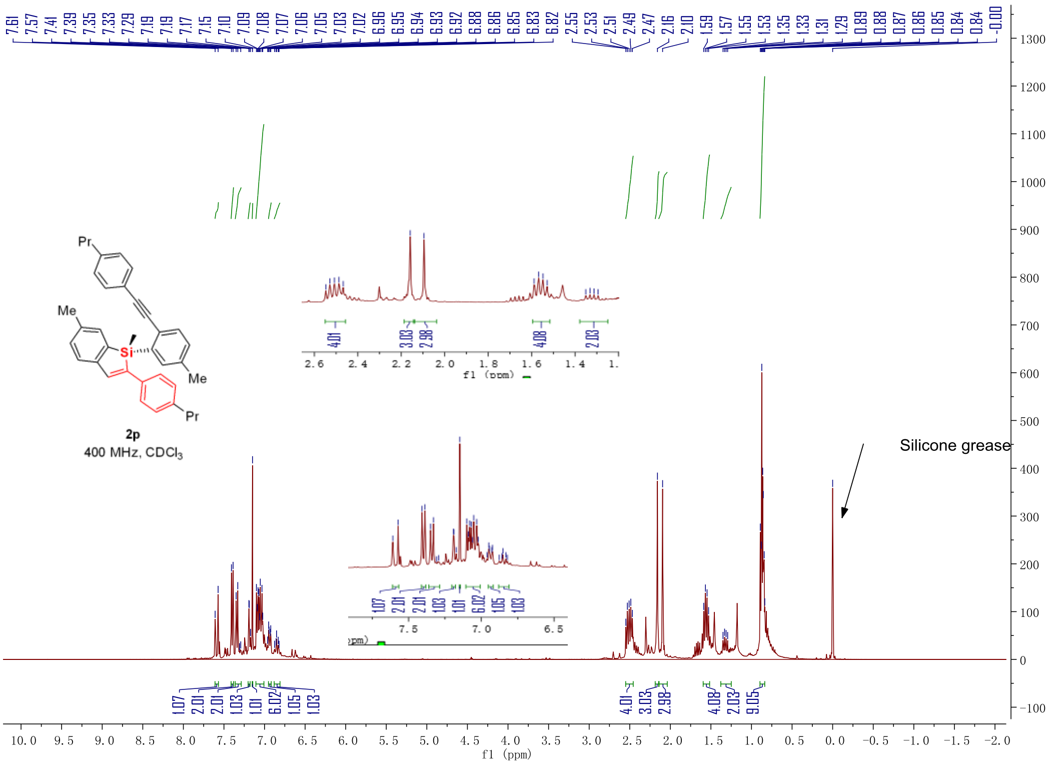

Figure S72. ^{1}H NMR (400 MHz, $CDCl_3$) spectrum of compound **2p** , related to **Scheme 2**

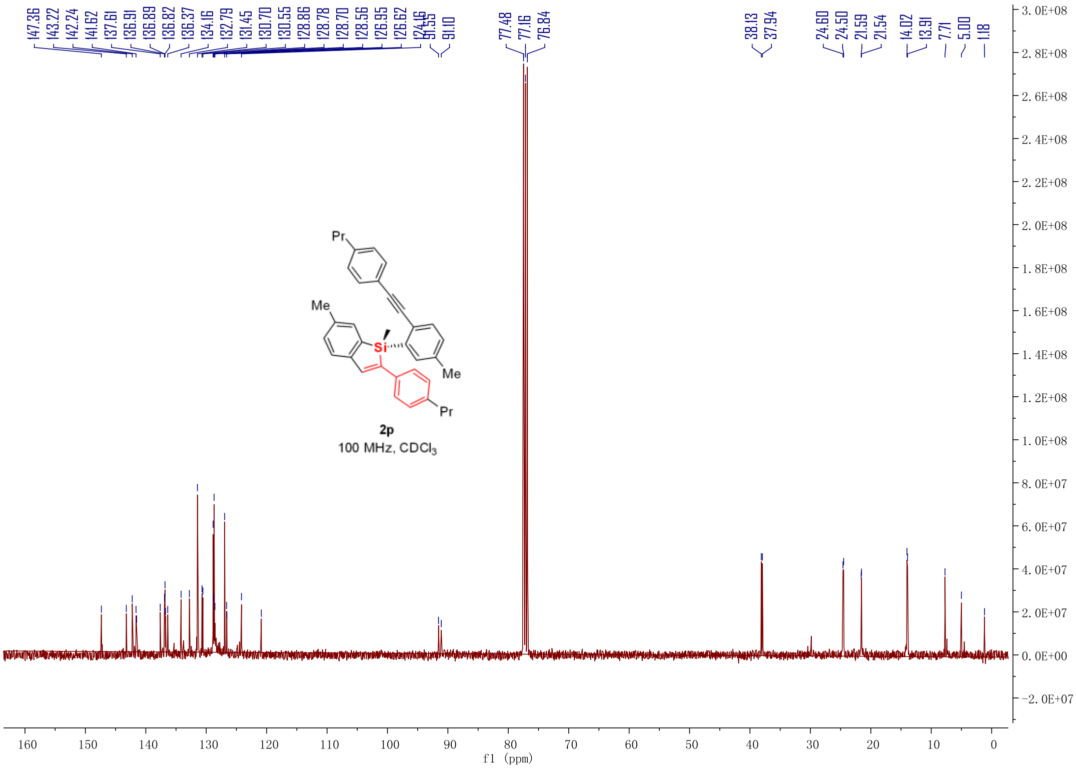

Figure S73. ^{13}C NMR (100 MHz, $CDCl_3$) spectrum of compound **2p,** related to **Scheme 2**

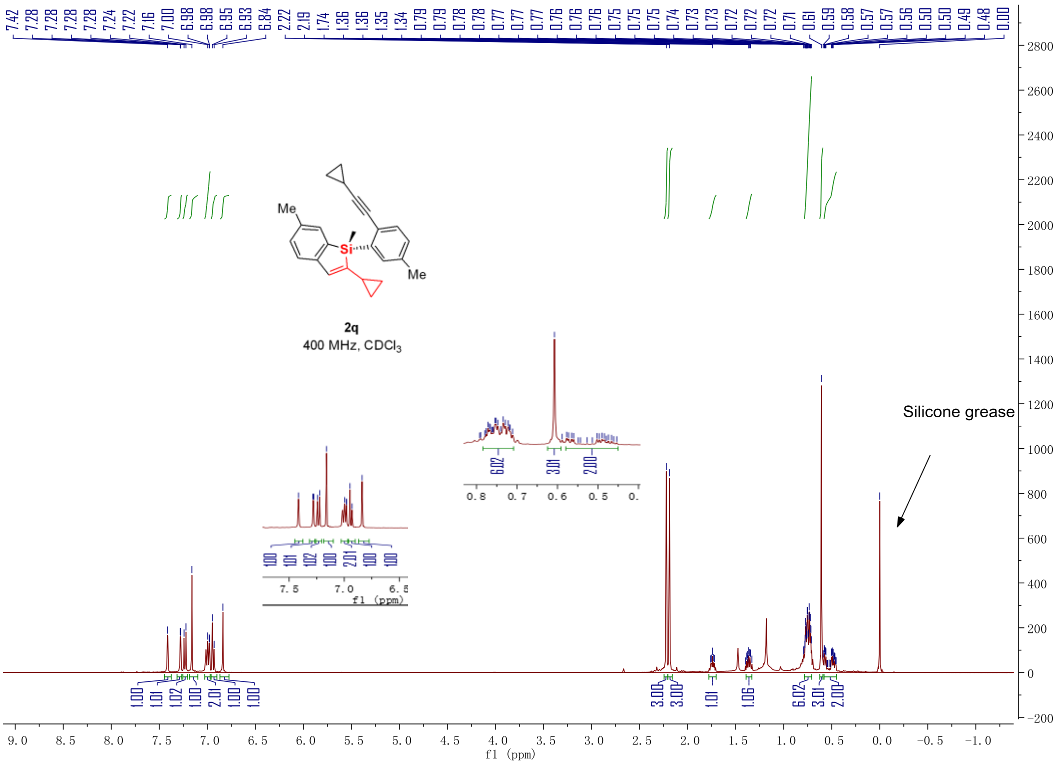

Figure S74. ^{1}H NMR (400 MHz, $CDCl_3$) spectrum of compound **2q** , related to **Scheme 2**

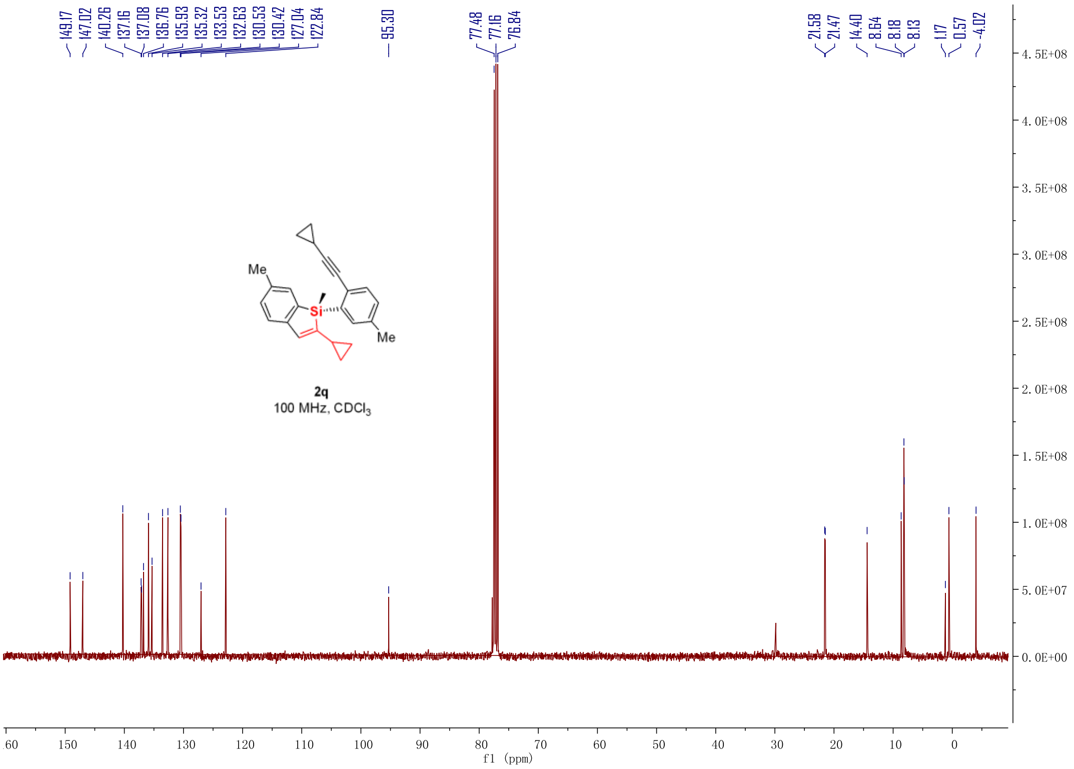

Figure S75. ^{13}C NMR (100 MHz, $CDCl_3$) spectrum of compound **2q,** related to **Scheme 2**

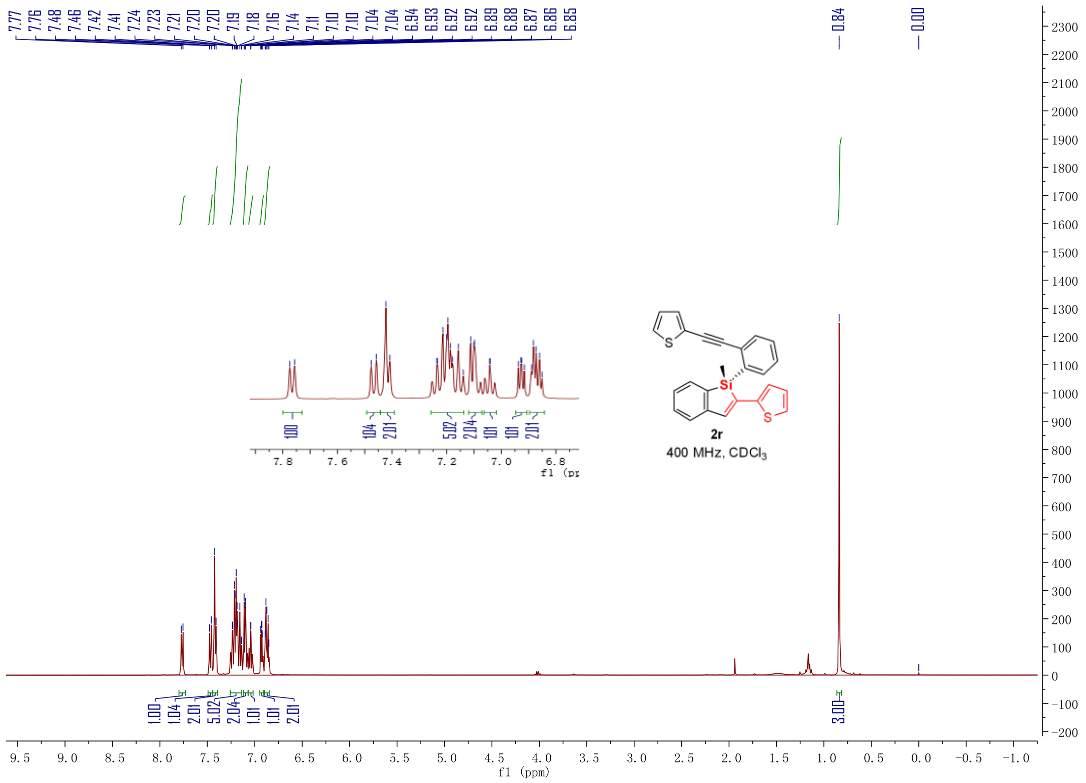

Figure S76. ^{1}H NMR (400 MHz, $CDCl_3$) spectrum of compound **2r** , related to **Scheme 2**

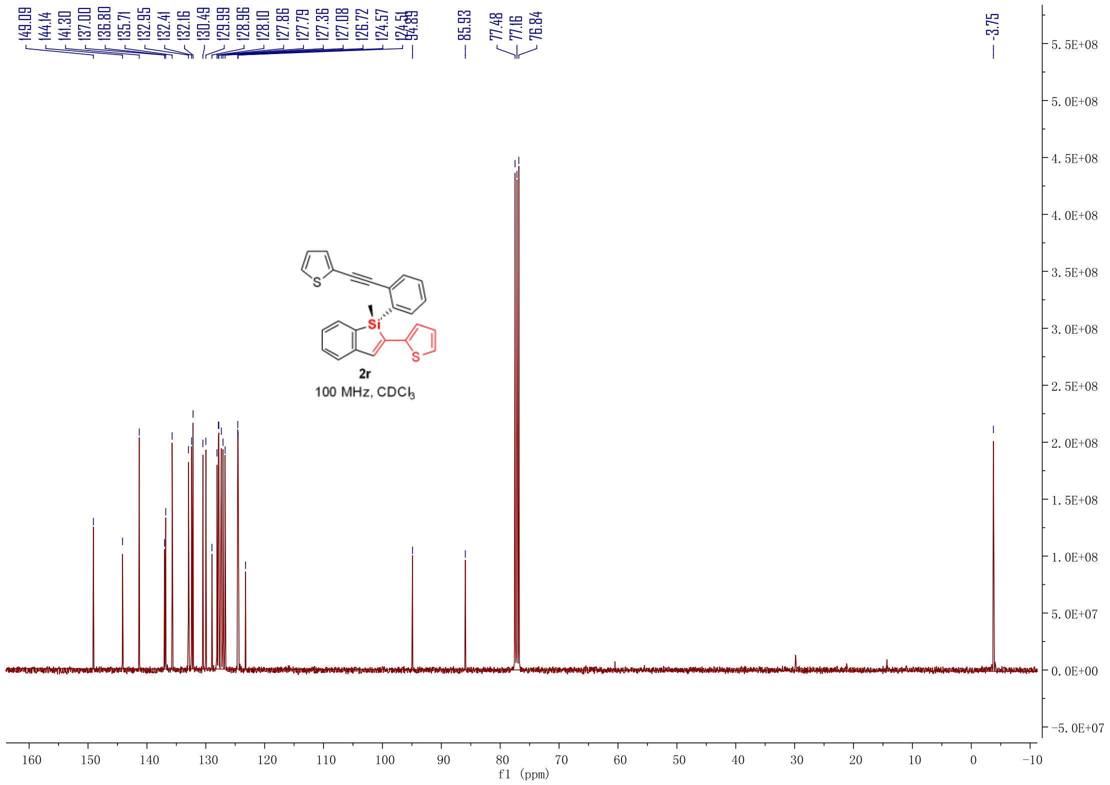

Figure S77. ^{13}C NMR (100 MHz, $CDCl_3$) spectrum of compound **2r,** related to **Scheme 2**

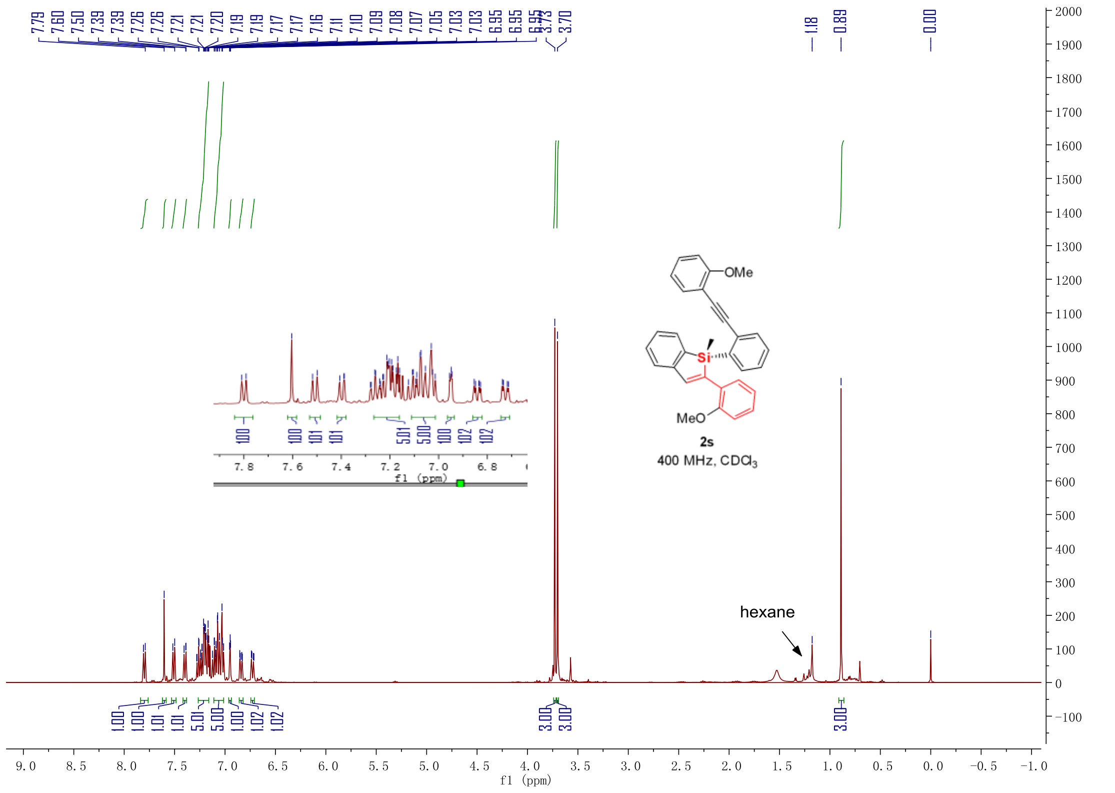

Figure S78. ^{1}H NMR (400 MHz, $CDCl_3$) spectrum of compound **2s** , related to **Scheme 2**

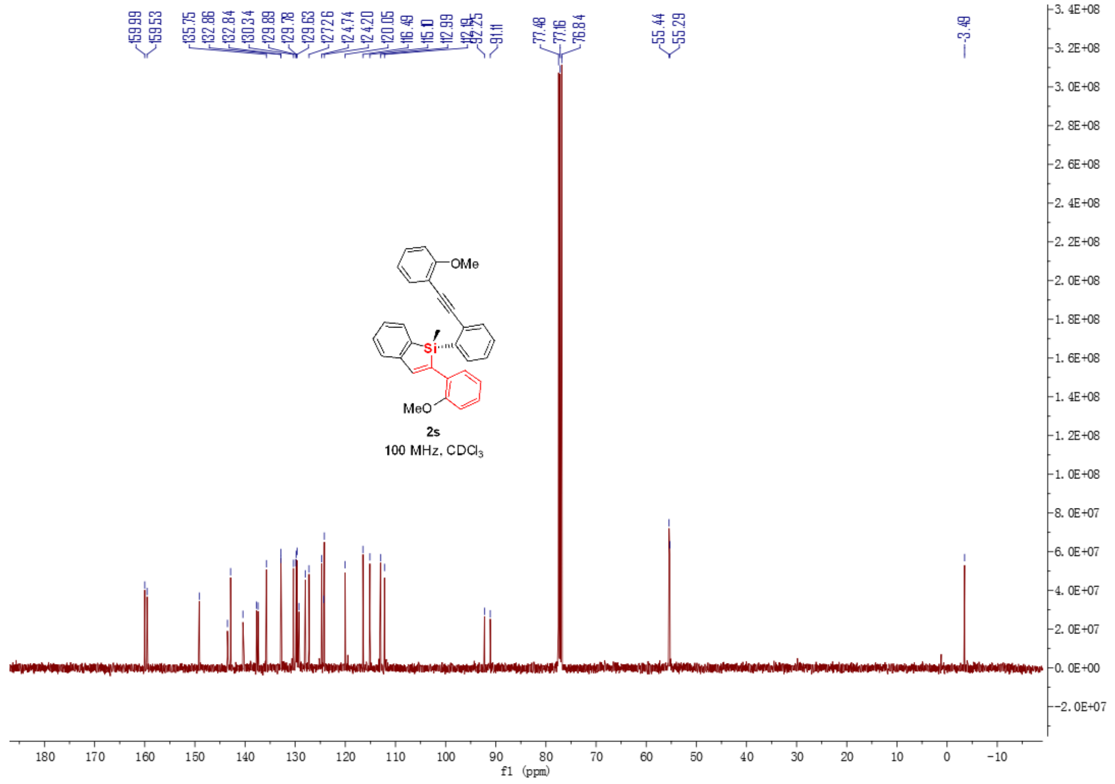

Figure S79. ^{13}C NMR (100 MHz, $CDCl_3$) spectrum of compound **1s,** related to **Scheme 2**

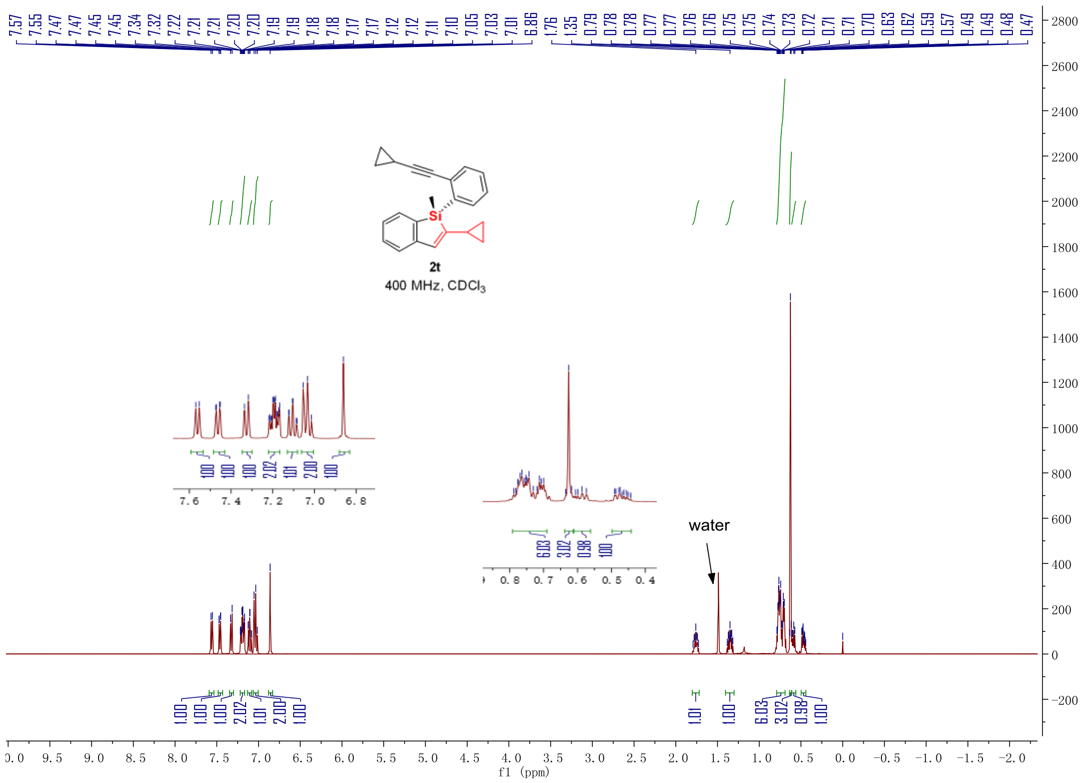

Figure S80. ^{1}H NMR (400 MHz, $CDCl_3$) spectrum of compound **2t** , related to **Scheme 2**

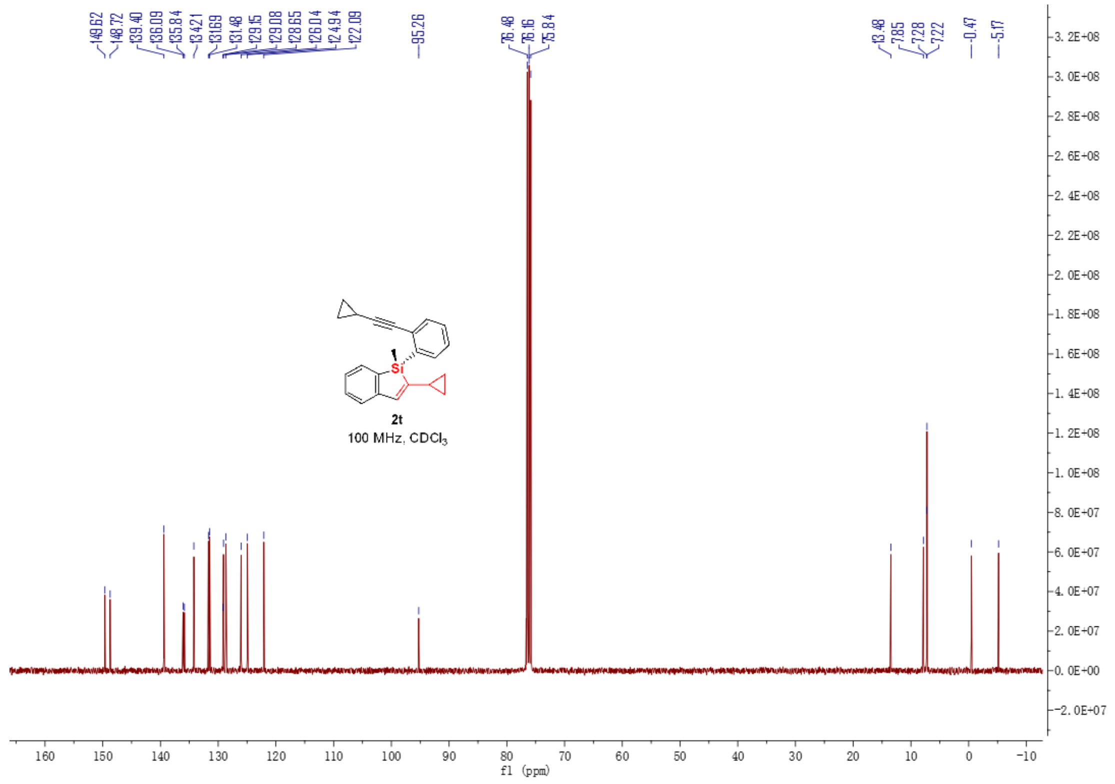

Figure S81. ^{13}C NMR (100 MHz, $CDCl_3$) spectrum of compound **1t,** related to **Scheme 2**

React IR experiment for the detection of benzosilole backbone

IR analysis was carried out under the reaction conditions: Under N_2 atmosphere, $[Rh(cod)Cl]_2$ (4.9 mg, 5 mol%), **L8** (13.8 mg, 12 mol%), KOtBu (2.7 mg, 12 mol%) and evacuated under high vacuum and backfilled with N_2. Toluene (1 mL) was next added and stirred at room temperature for about 0.5 h. Then methylbis(2-(phenylethynyl)phenyl)silane **1a** (79.6 mg, 0.2 mmol) were added sequentially, The mixture was stirred at 70 ^{o}C in a preheated oil. Then the IR probe was inserted and the IR data collection was started. The mixture was stirred at 70 ^{o}C for about 72 h.

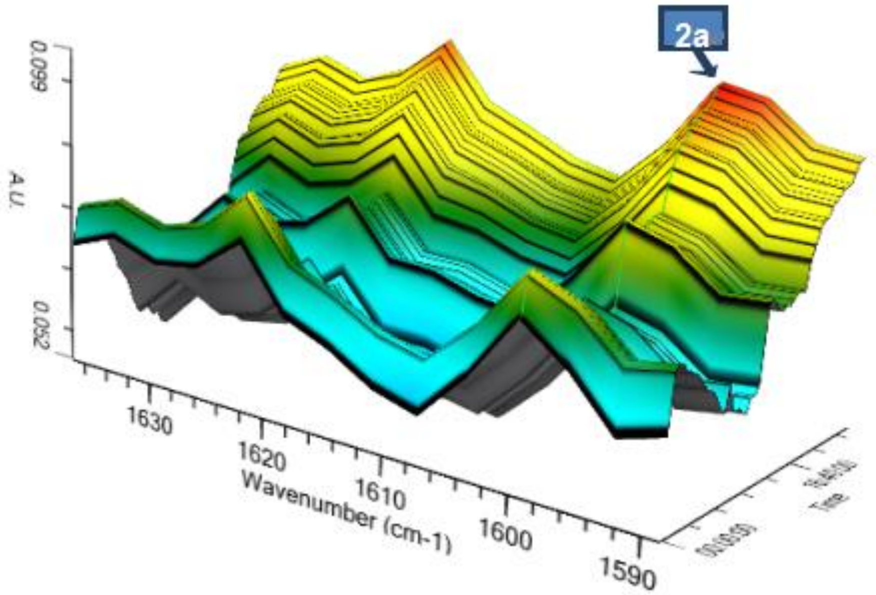

Figure S82. IR spectra for detection of the formation of benzosilole **2a** from the hydrosilylation of alkynyl C(sp)-C(sp) bond. Related to Figure 5

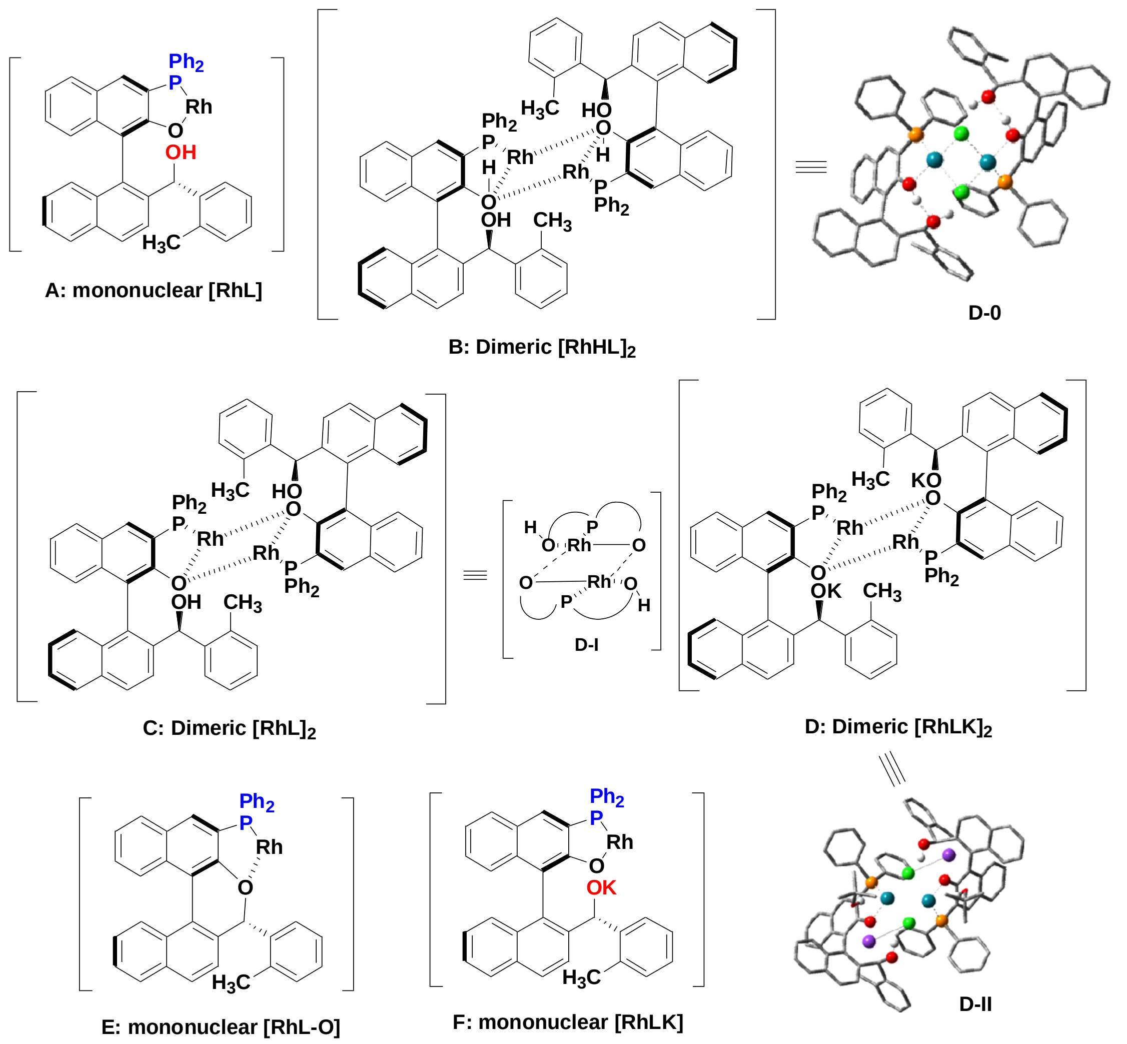

Figure S83 (continued). Possible Rh complex or intermediates in this reaction could be confirmed on the basis of the ^{31}P-NMR and ESI-MS analysis. For the Rh complexes, B-D, the existence of couplings between Rh and P in the ^{31}P NMR spectra. Related to Figure 5

Supplemental Figures for ESI-MS spectrums:

Figure S84. ESI(+)-MS analysis for the mixture of only $[Rh(cod)Cl]_2$ and Ar-BINMOL-Phos (*o*-Me) in toluene. Related to Figure 5

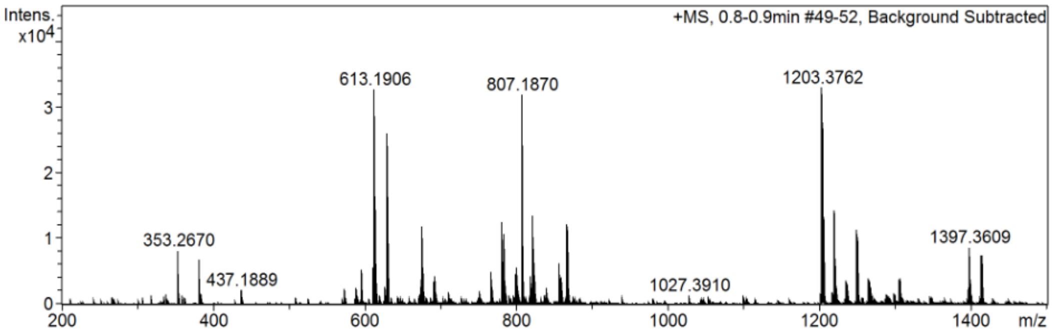

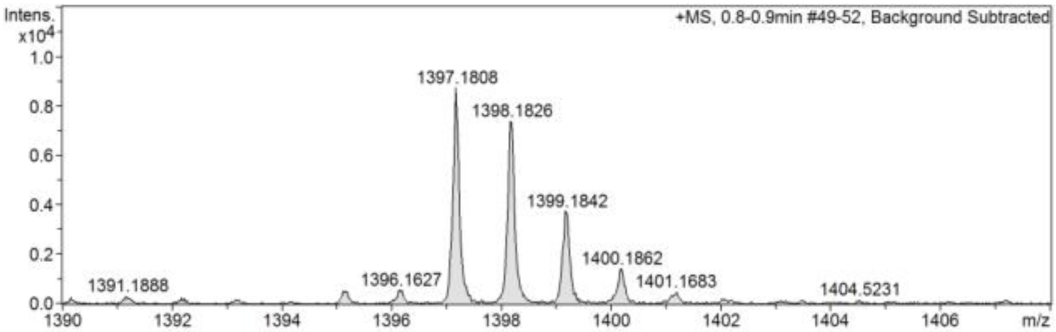

Meas. m/z	#	Ion Formula	m/z	err [ppm]	mSigma	# Sigma	Score	rdb	e⁻ Conf	N-Rule
1397.1808	1	C80H59Na2O4P2Rh2	1397.1789	-1.4	32.8	1	-1.#J	52.5	even	ok

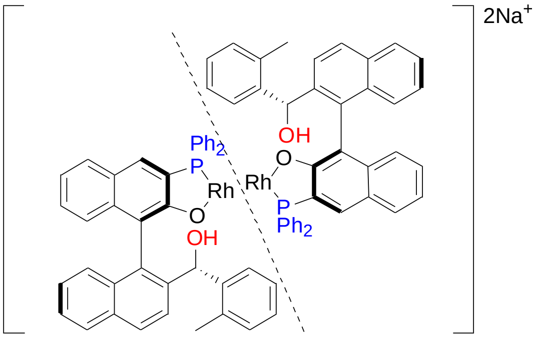

Chemical Formula: $C_{80}H_{60}O_4P_2Rh_2$
Exact Mass: 1352.2077
Molecular Weight: 1353.1145

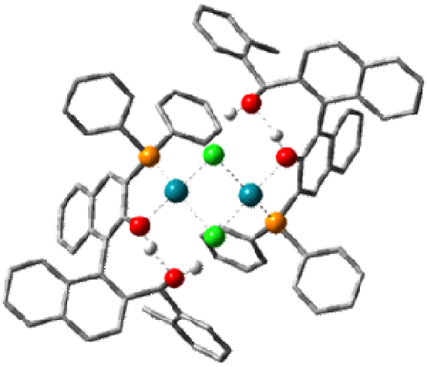

D-0

Figure S85. ESI(+)-MS analysis for the mixture of $[Rh(cod)Cl]_2$, alkyne, Ar-BINMOL-Phos (*o*-Me) in toluenen. Related to Figure 5

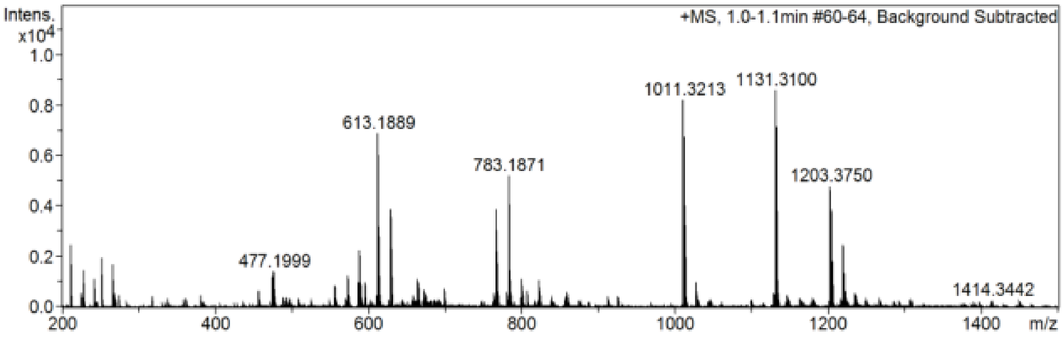

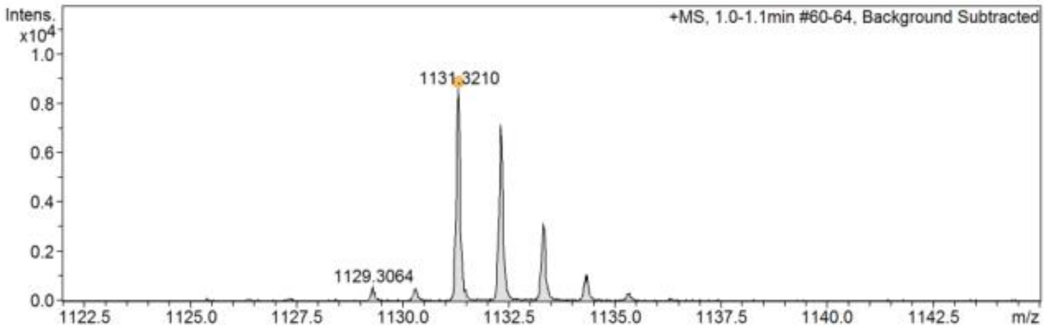

Meas. m/z	#	Ion Formula	m/z	err [ppm]	mSigma	# Sigma	Score	rdb	e⁻ Conf	N-Rule
1131.3210	1	C73H61O2PRhSi	1131.3228	1.5	15.1	1	100.00	45.5	even	ok

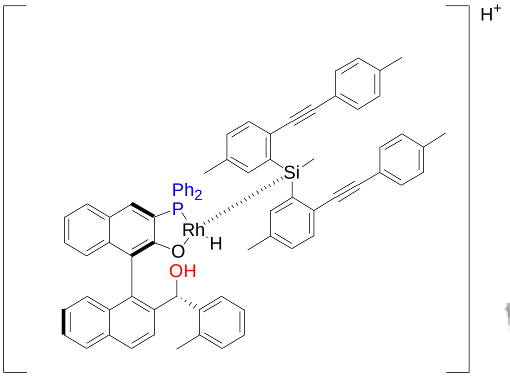

Chemical Formula: $C_{73}H_{61}O_2PRhSi$
Exact Mass: 1131.3233
Molecular Weight: 1132.2533

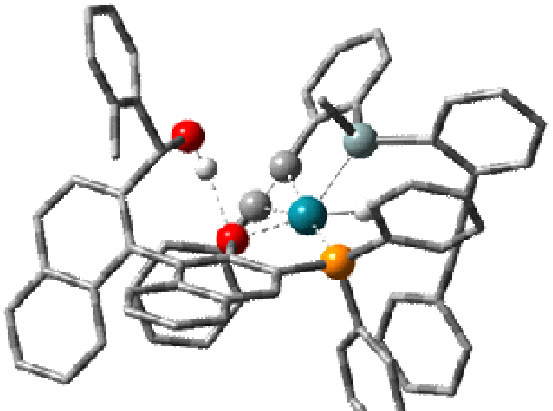

(Int1)

Supplemental Figures for fluorescence spectra of product:

Figure S86. UV-vis absorption (down) and fluorescence emission (up) properties of seven compounds in DCM solvents.(10^{-5} M). related to Figure 2

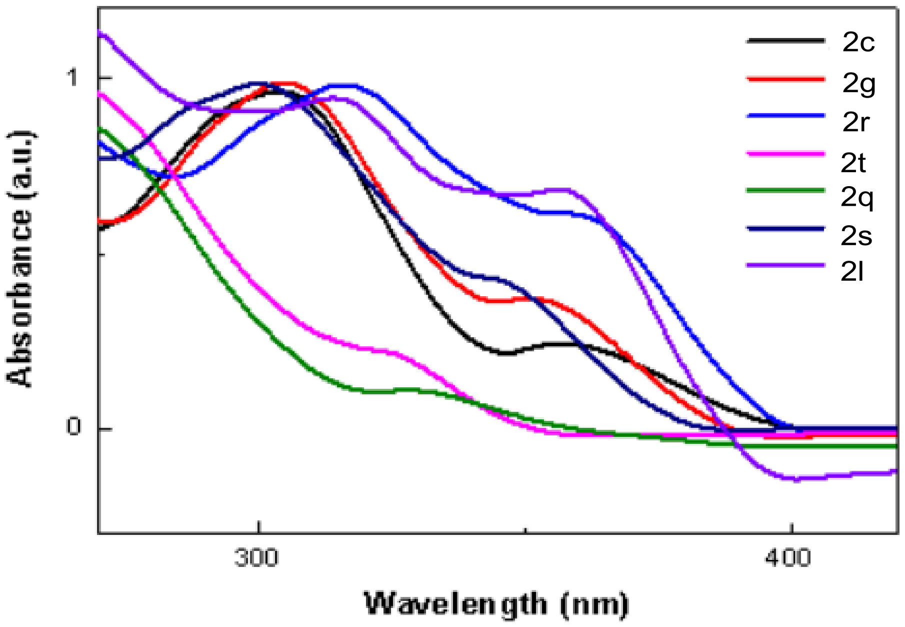

Figure S87. Fluorescence emission spectra of **2g** (5 μM) in THF/water mixtures (fw = 0 to 90%). λ_{ex}=300 nm, λ_{es}=550 nm. related to Figure 2

.

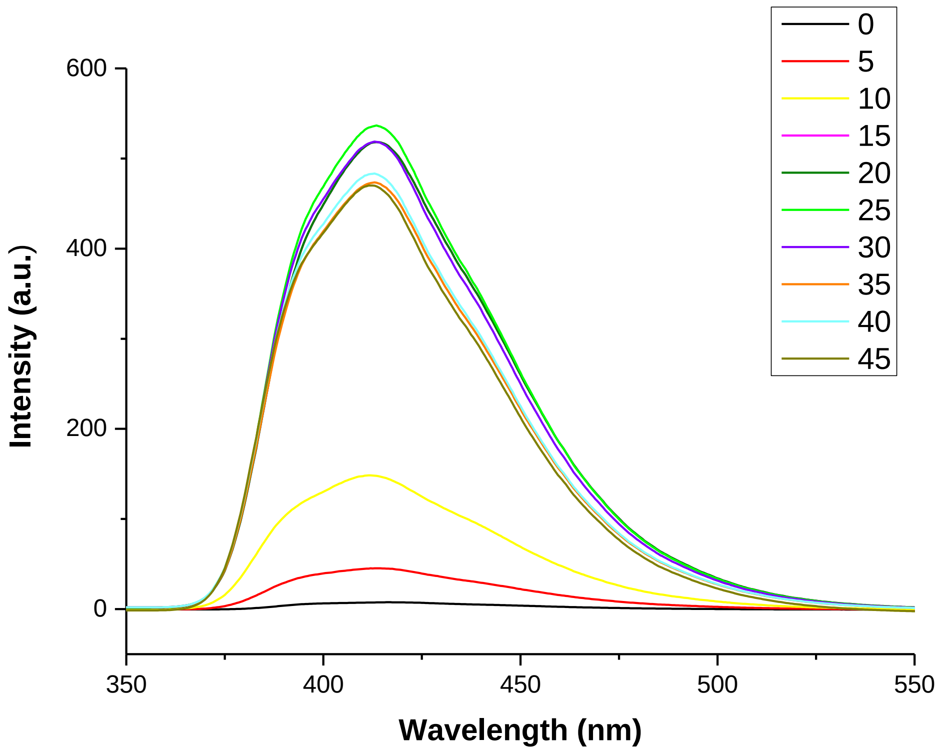

Figure S88. For racemic **2g** (AIE) related to Figure 2

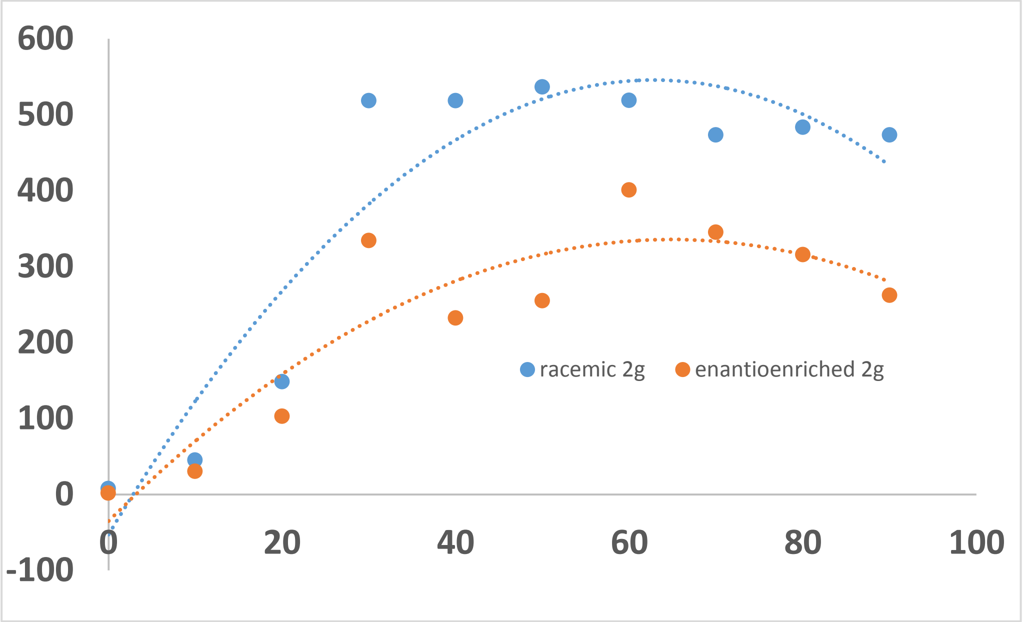

Figure S89. The photography for the AIE phenomena of **2g.** Related to Figure 2

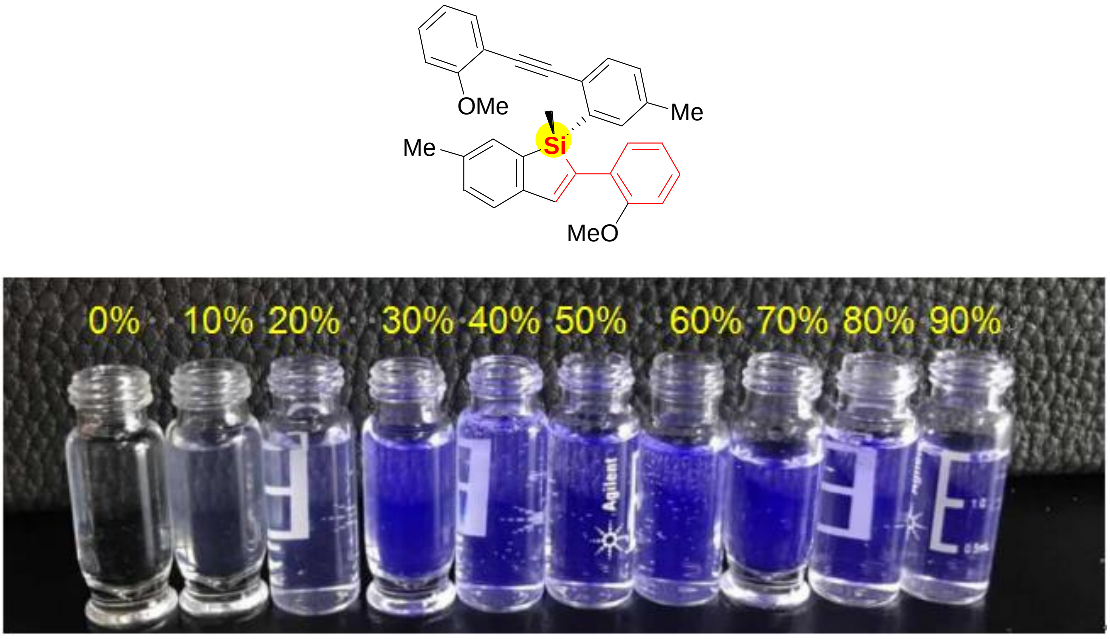

Figure S90. The industrial method for preparation of silicon rubber with blue blue-fluorescence because of the additive of alkyne-substituted benzosilole **2g** (10^{-4} w/w). Related to Figure 2

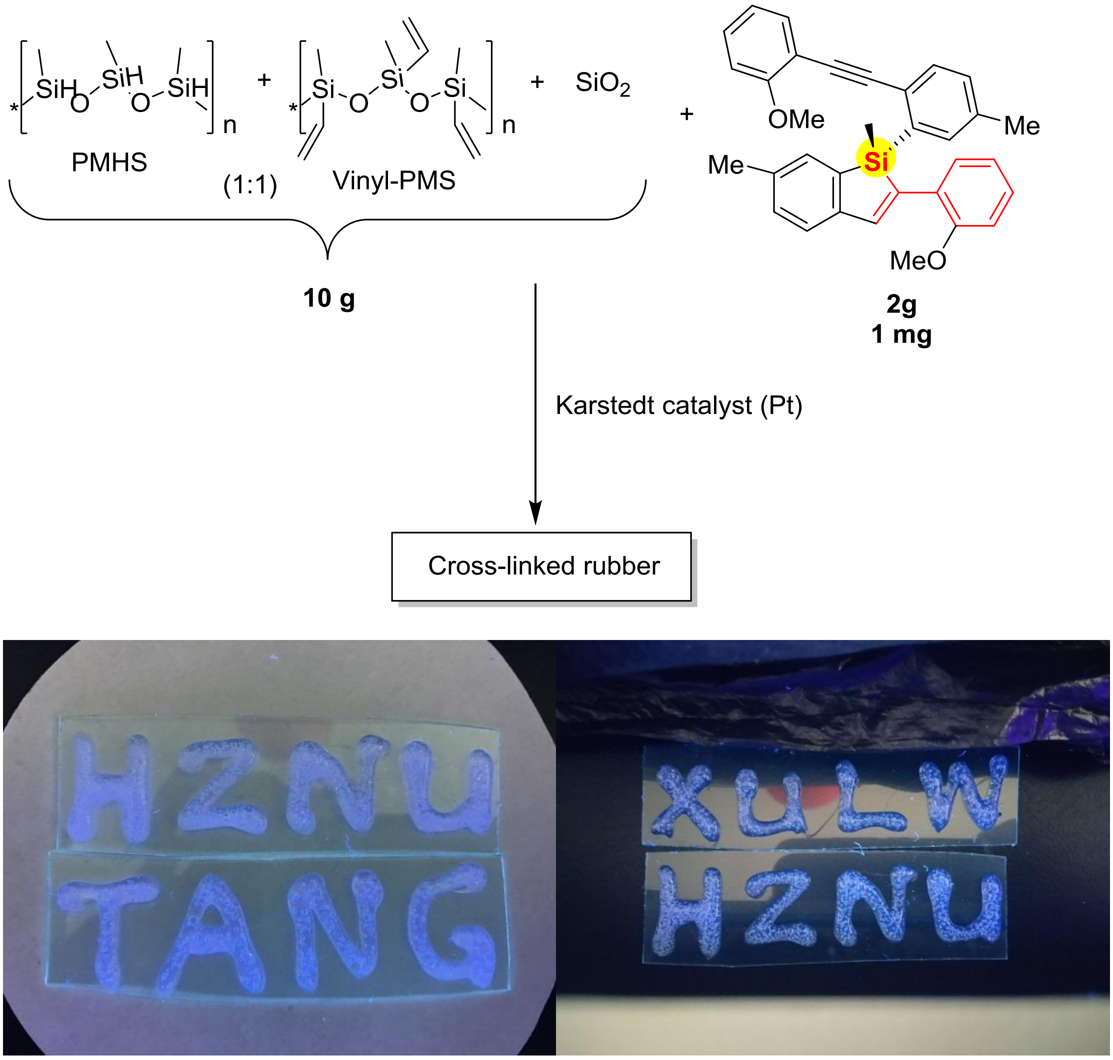

CPL spectra of product 2g:

Figure S91. CPL (upper panel) and DC (nether panel) spectra of (*S*) (black lines) and (*R*) (red lines) in $CHCl_3$ (1.0×10^{-3} M). Related to Figure 2

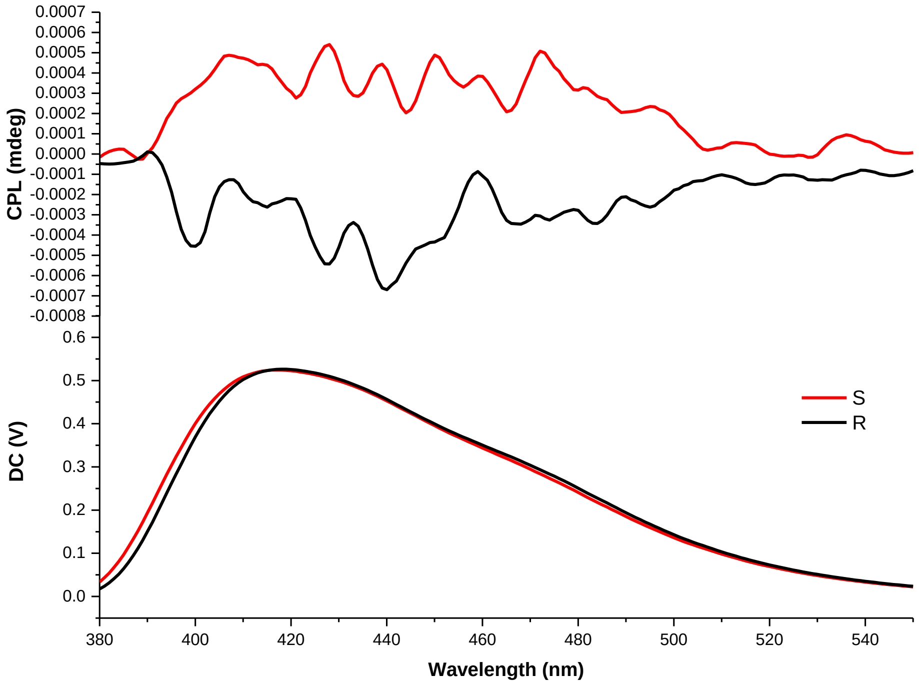

Table S92. HOMO and LUMO energy computed for product. Related to Figure 2

product	HOMO	LUMO	Δ（ev）
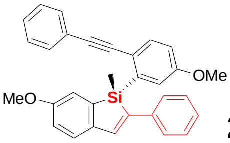 2c	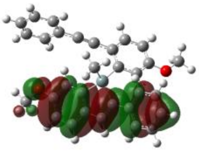 -4.529ev	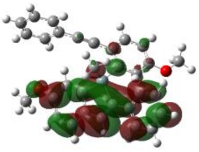 -1.795ev	2.734
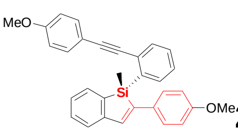 2g	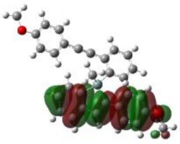 -4.570ev	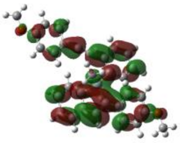 -1.857ev	2.713
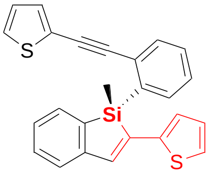 2r	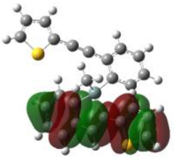 -4.751ev	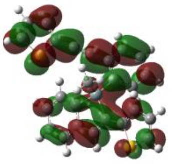 -2.174ev	2.577
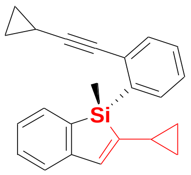 2t	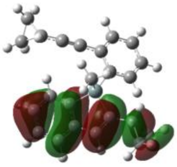 -4.867ev	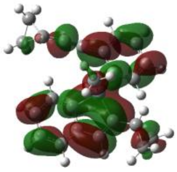 -1.575ev	3.292
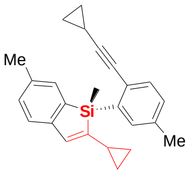 2q	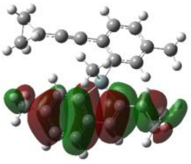 -4.738ev	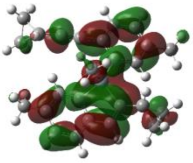 -1.480ev	3.258
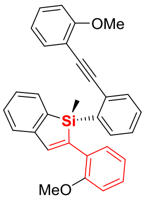 2S	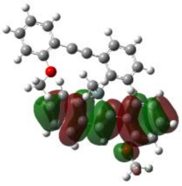 -4.640ev	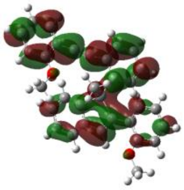 -1.883ev	2.757
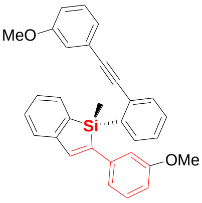 2l	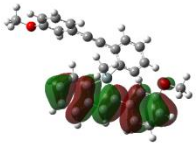 -4.818ev	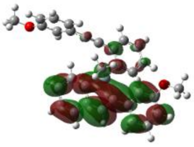 -1.920ev	2.898

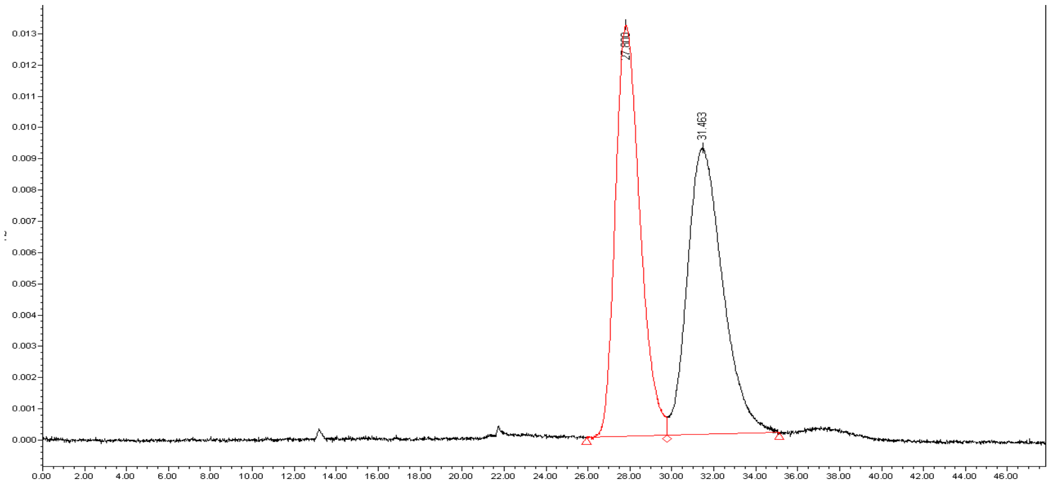

	Time/min	Area	Height	Area%
1	27.800	1075240	13152	50
2	31.463	1075240	9160	50

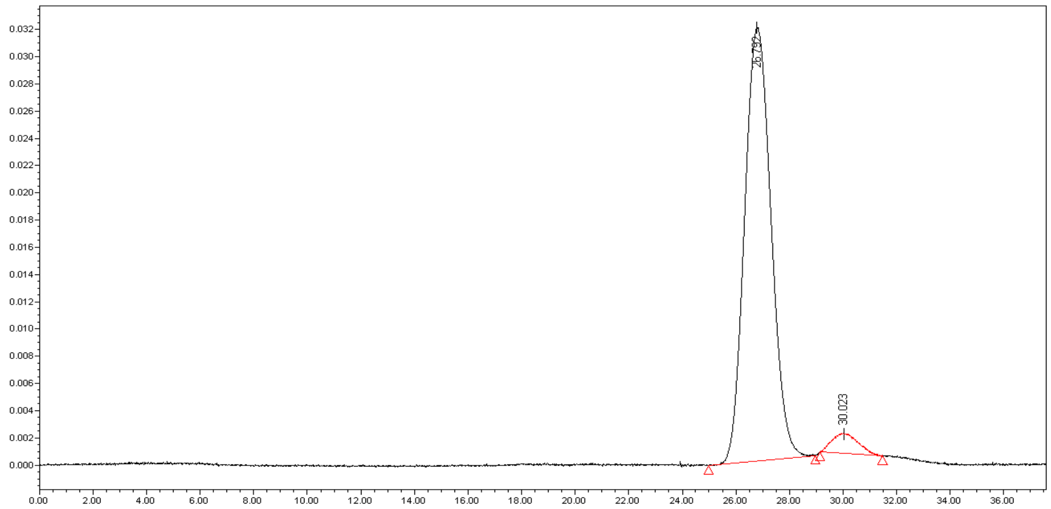

	Time/min	Area	Height	Area%
1	26.792	2193457	31842	95.51
2	30.023	103043	1485	4.49

Figure S93, the HPLC spectrum of compound **2a**, related to **Scheme 2**

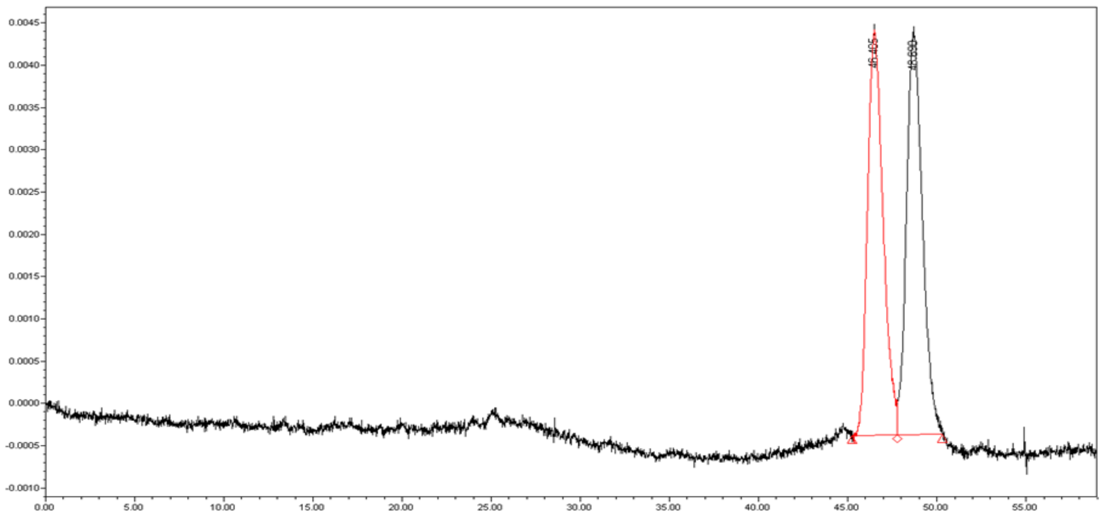

	Time/min	Area	Height	Area%
1	46.485	291019	4784	49.38
2	48.690	298330	4768	50.62

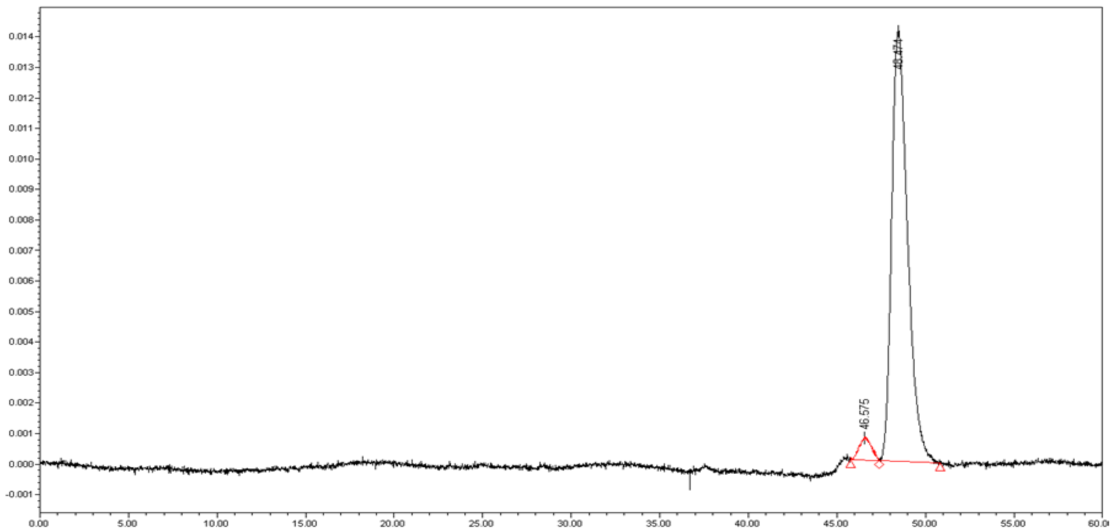

	Time/min	Area	Height	Area%
1	46.575	35478	739	3.86
2	48.474	883685	14102	96.14

Figure S94, the HPLC spectrum of compound **2b**, related to **Scheme 2**

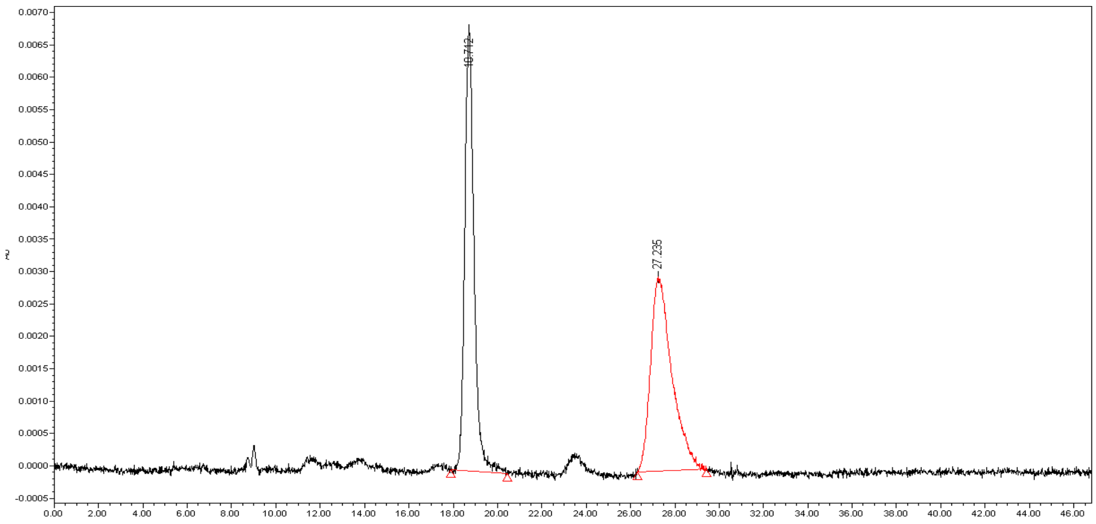

	Time/min	Area	Height	Area%
1	18.712	199777	6793	50.03
2	27.235	199505	2981	49.97

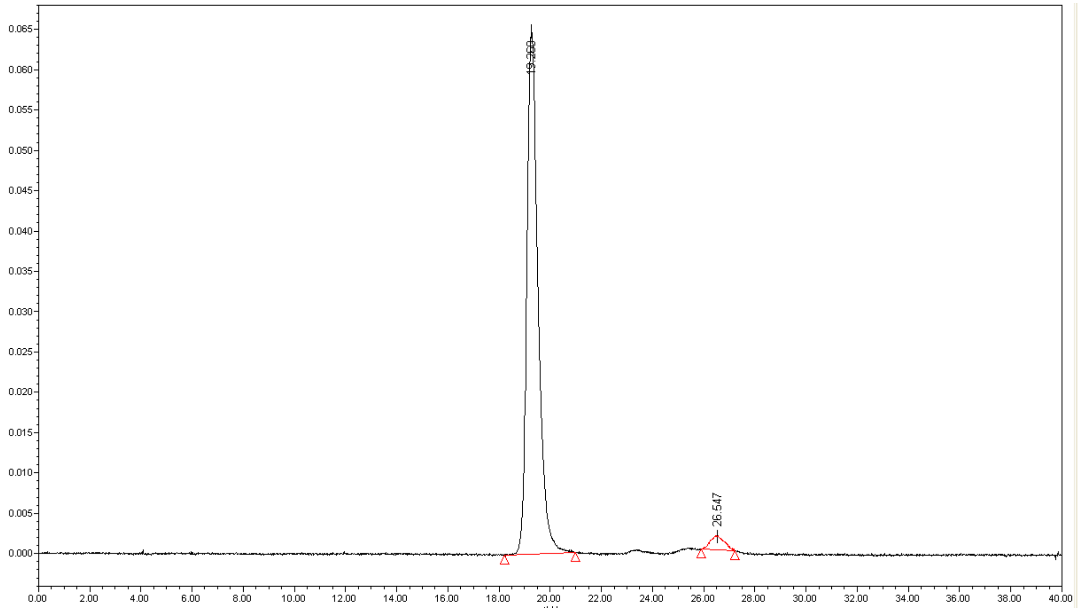

	Time/min	Area	Height	Area%
1	19.268	1979257	64815	96.69
2	26.547	68054	1716	3.32

Figure S95, the HPLC spectrum of compound **2c**, related to **Scheme 2**

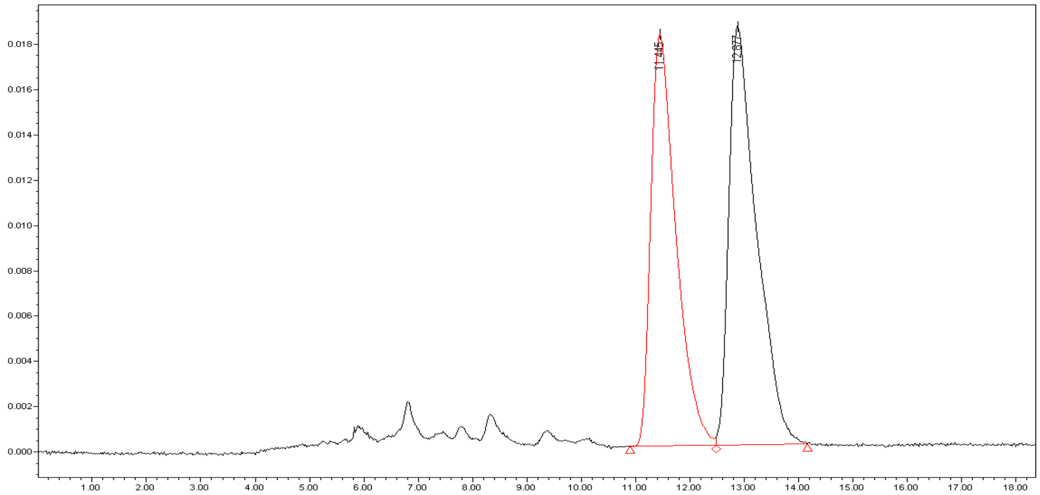

	Time/min	Area	Height	Area%
1	11.445	585900	18153	47.25
2	12.877	654006	18480	52.75

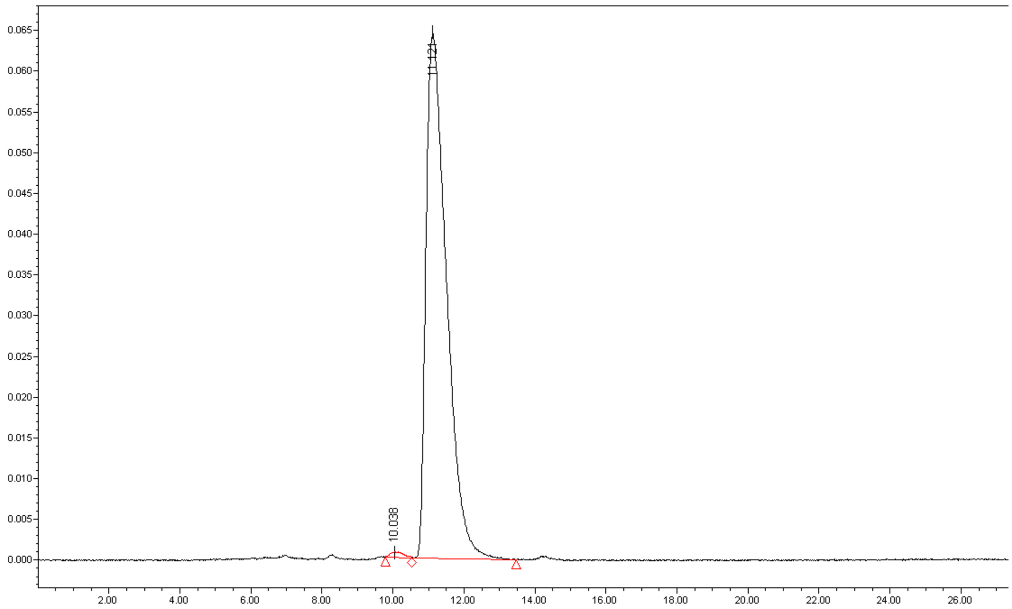

	Time/min	Area	Height	Area%
1	10.038	17535	673	0.68
2	11.121	2578652	64490	99.32

Figure S96, the HPLC spectrum of compound **2d**, related to **Scheme 2**

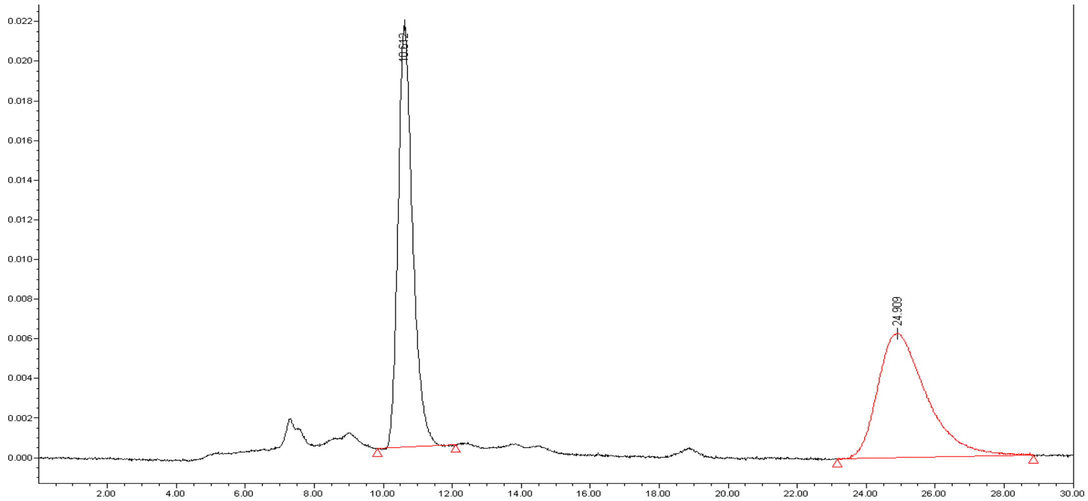

	Time/min	Area	Height	Area%
1	10.612	628213	21274	50.39
2	24.909	618583	6286	49.61

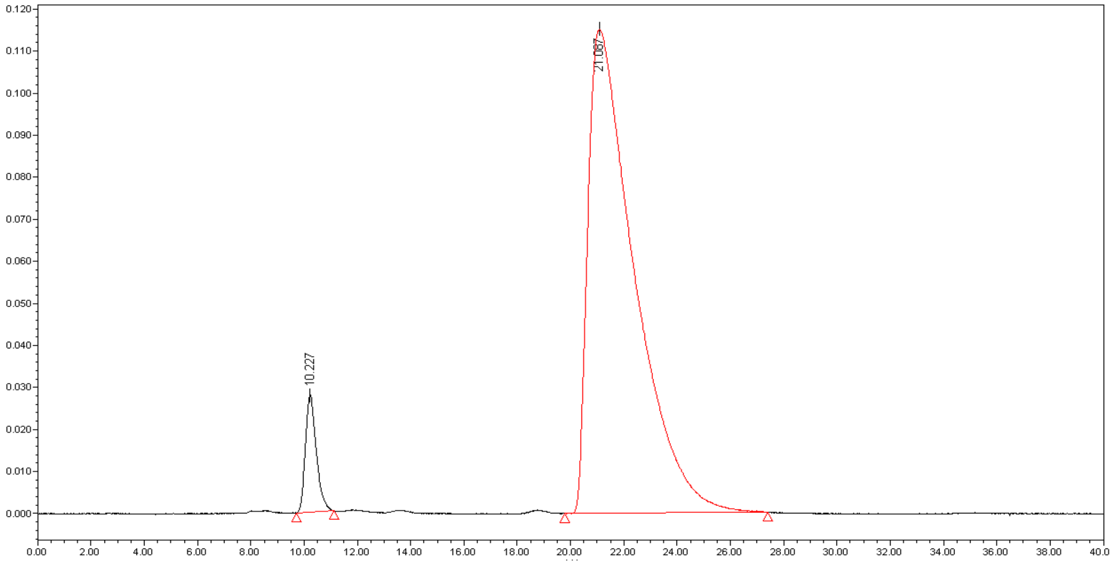

	Time/min	Area	Height	Area%
1	10.227	793482	27894	5.4
2	21.087	13896236	115139	94.6

Figure S97, the HPLC spectrum of compound **2e**, related to **Scheme 2**

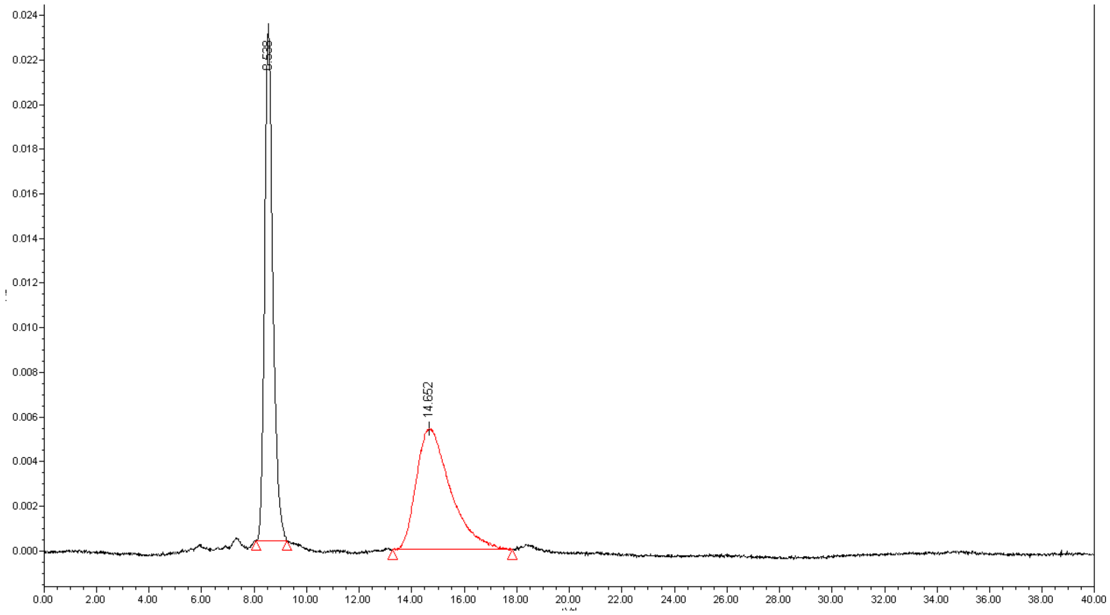

	Time/min	Area	Height	Area%
1	8.538	504871	22886	50.72
2	14.625	490480	5420	49.28

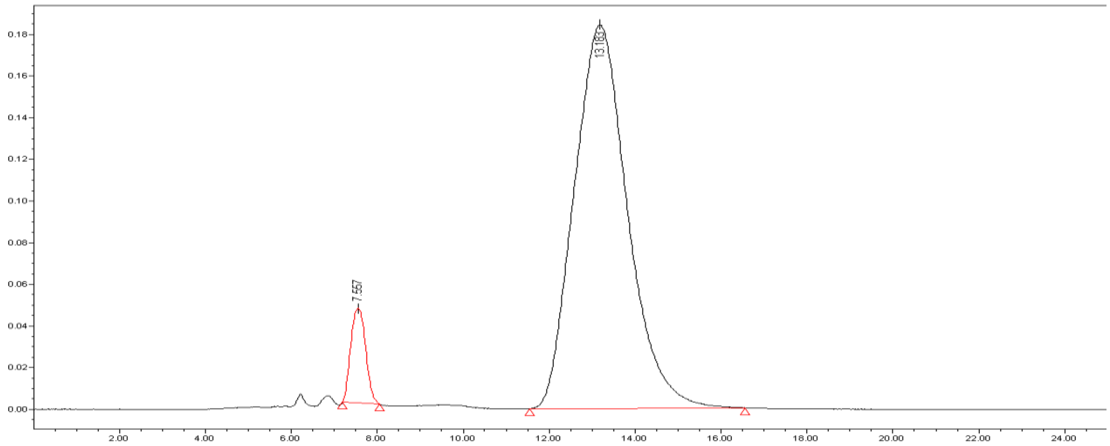

	Time/min	Area	Height	Area%
1	7.557	1098019	45399	6.47
2	13.183	15874214	184180	93.53

Figure S98, the HPLC spectrum of compound **2f**, related to **Scheme 2**

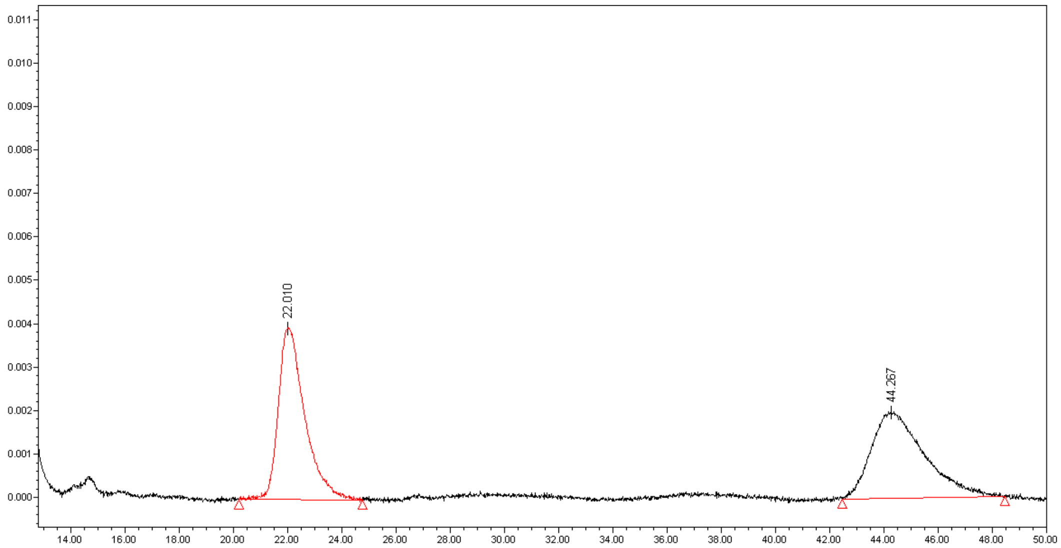

	Time/min	Area	Height	Area%
1	22.010	269297	3960	49.89
2	44.267	270455	1965	50.11

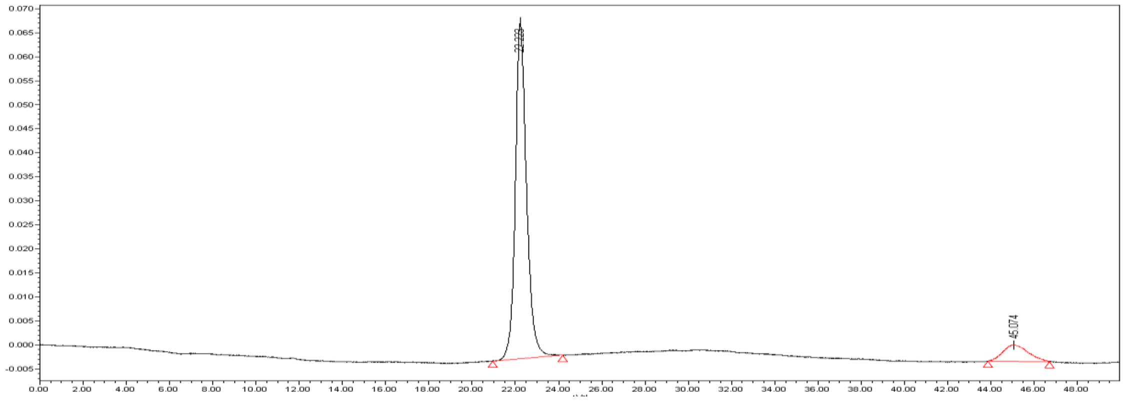

	Time/min	Area	Height	Area%
1	22.223	2515042	70131	90.16
2	45.074	273306	3394	9.84

Figure S99, the HPLC spectrum of compound **2g**, related to **Scheme 2**

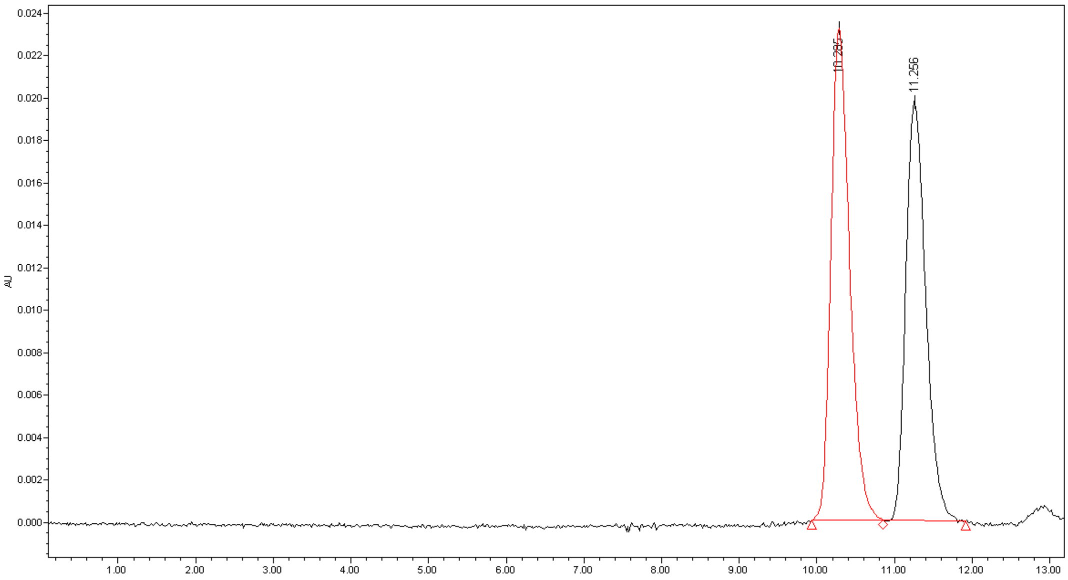

	Time/min	Area	Height	Area%
1	11.286	353752	20995	52.68
2	11.258	317785	17791	47.32

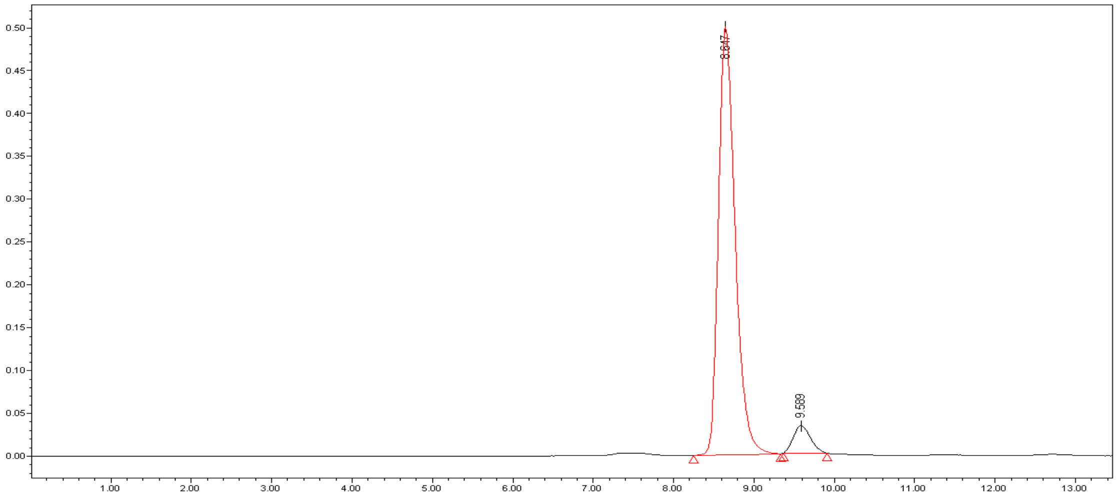

	Time/min	Area	Height	Area%
1	8.647	7203931	499663	93.88
2	9.589	469848	31878	6.12

Figure S100, the HPLC spectrum of compound **2h**, related to **Scheme 2**

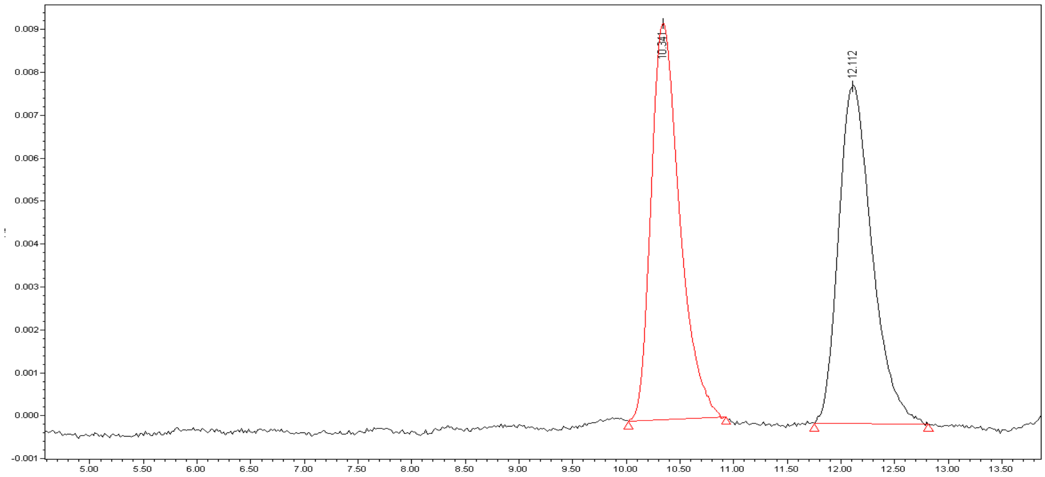

	Time/min	Area	Height	Area%
1	10.341	174103	9238	50.16
2	12.112	173006	7895	49.84

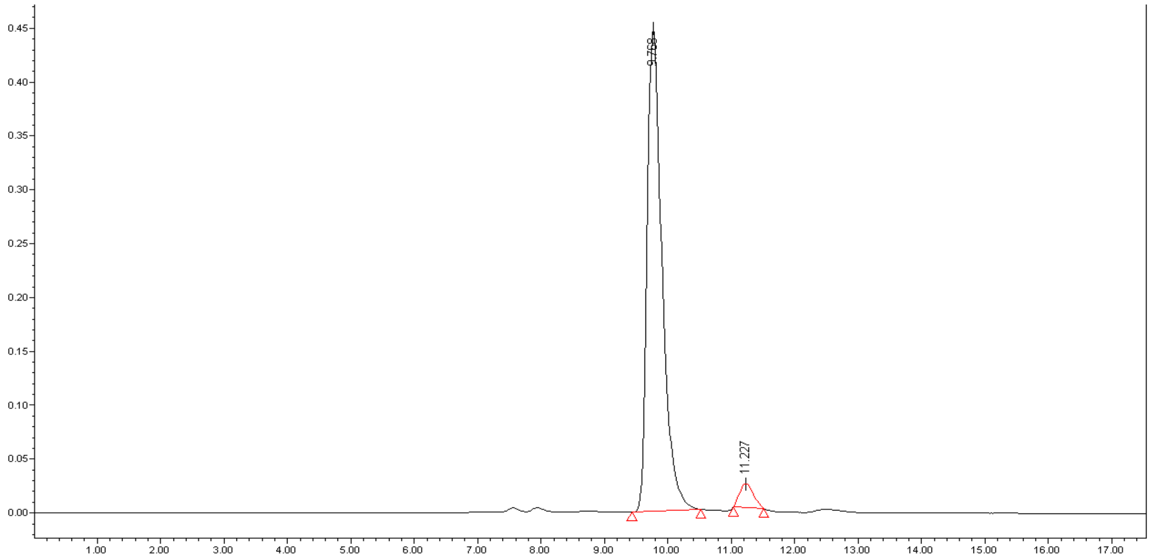

	Time/min	Area	Height	Area%
1	9.768	7356783	447901	95.66
2	11.227	333700	22215	4.34

Figure S101, the HPLC spectrum of compound **2i**, related to **Scheme 2**

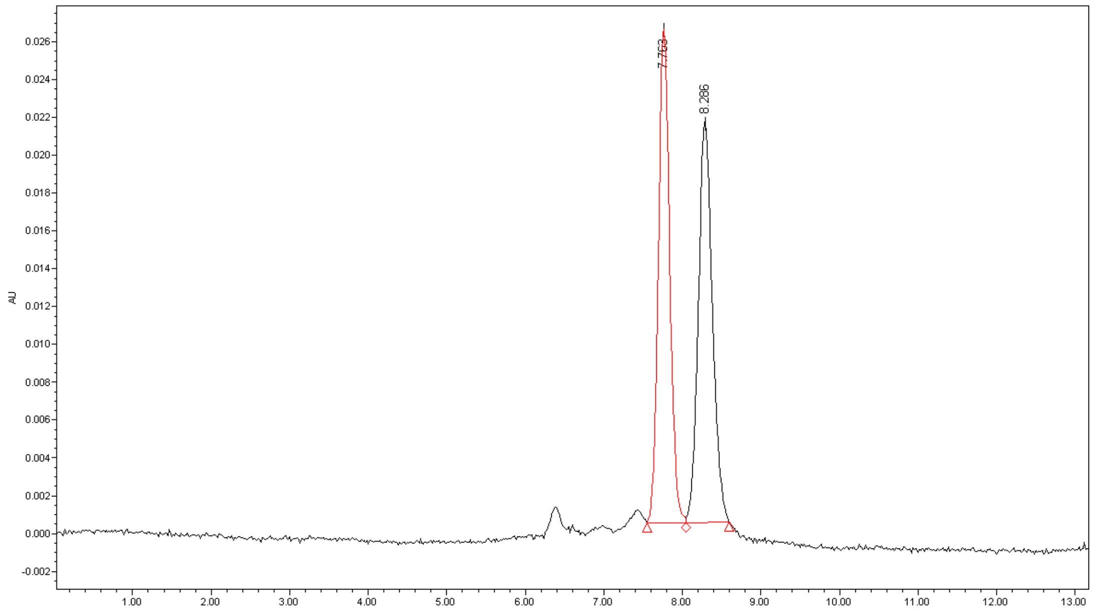

	Time/min	Area	Height	Area%
1	7.762	376725	35806	49.28
2	8.286	387792	29118	50.72

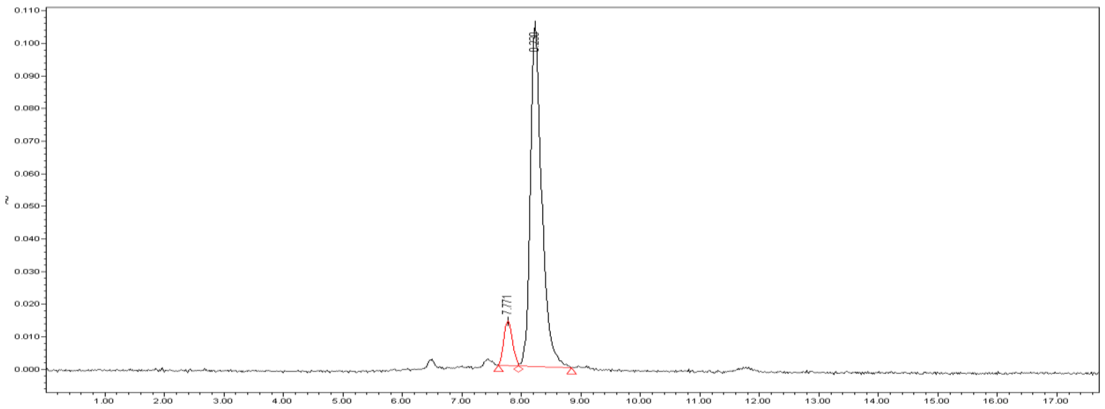

	Time/min	Area	Height	Area%
1	7.775	22408	2239	7.57
2	8.232	273573	20073	92.43

Figure S102, the HPLC spectrum of compound **2j**, related to **Scheme 2**

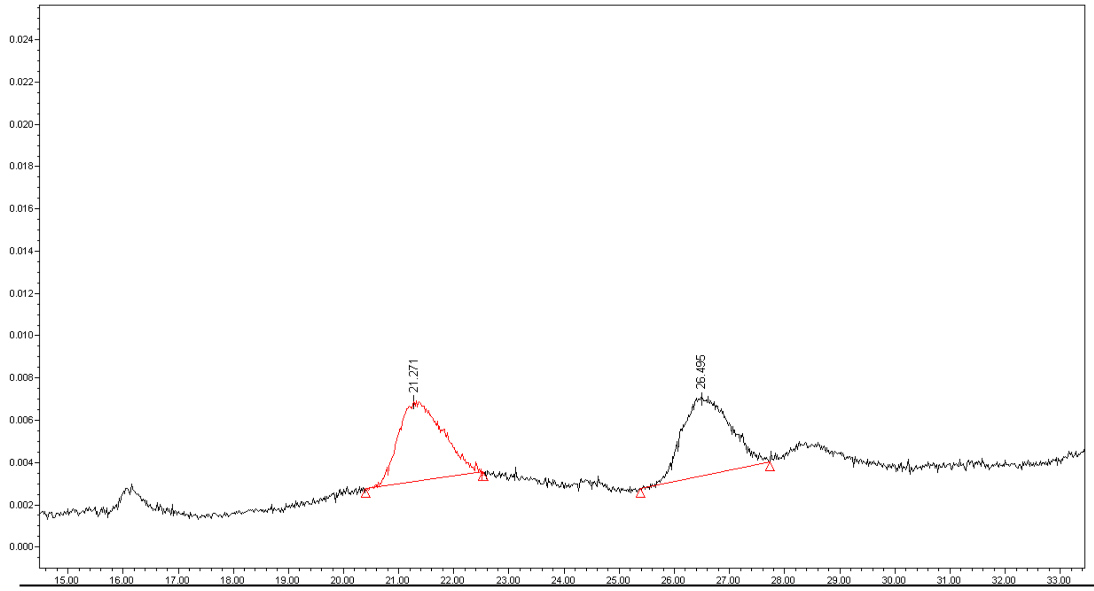

	Time/min	Area	Height	Area%
1	21.271	212218	3772	48.36
2	26.495	226588	3669	51.64

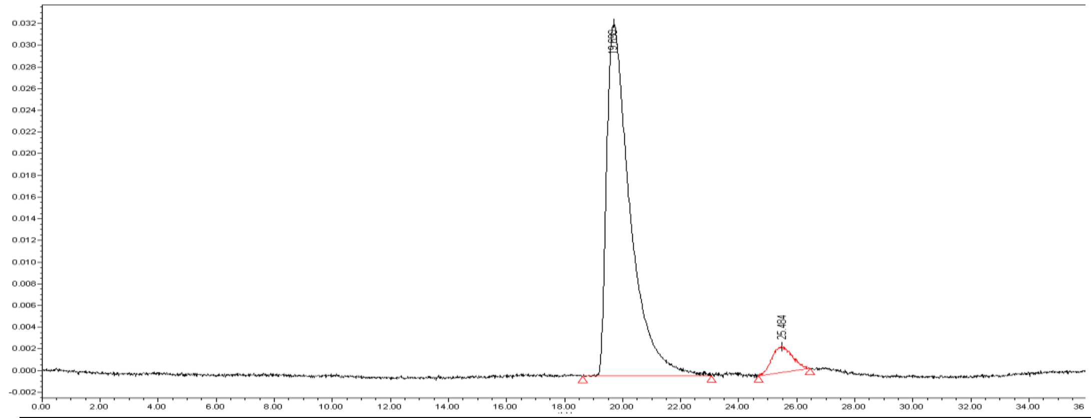

	Time/min	Area	Height	Area%
1	19.698	1824895	32485	93.99
2	25.383	116669	2388	6.01

Figure S103, the HPLC spectrum of compound **2k**, related to **Scheme 2**

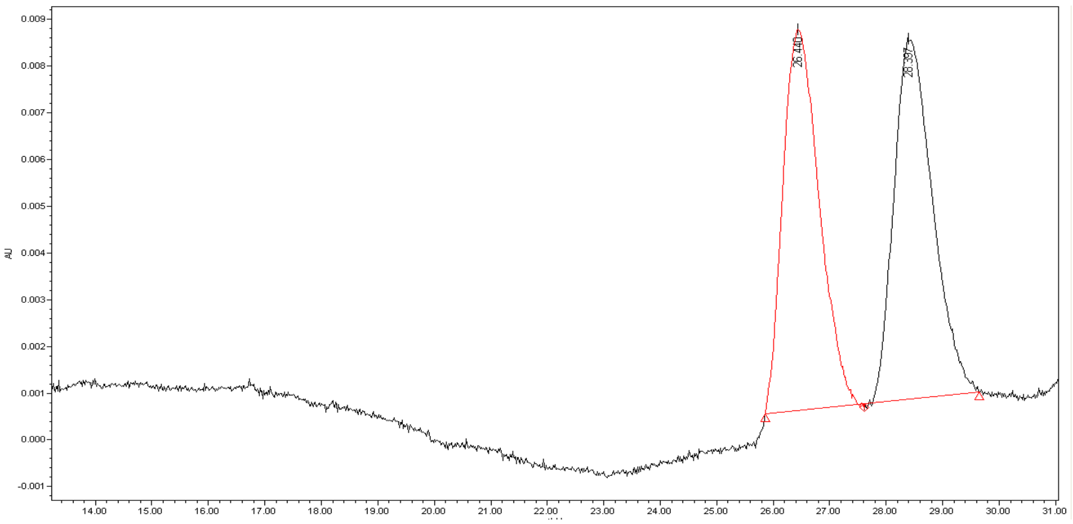

	Time/min	Area	Height	Area%
1	26.429	558438	12010	50.01
2	28.422	558275	11269	49.99

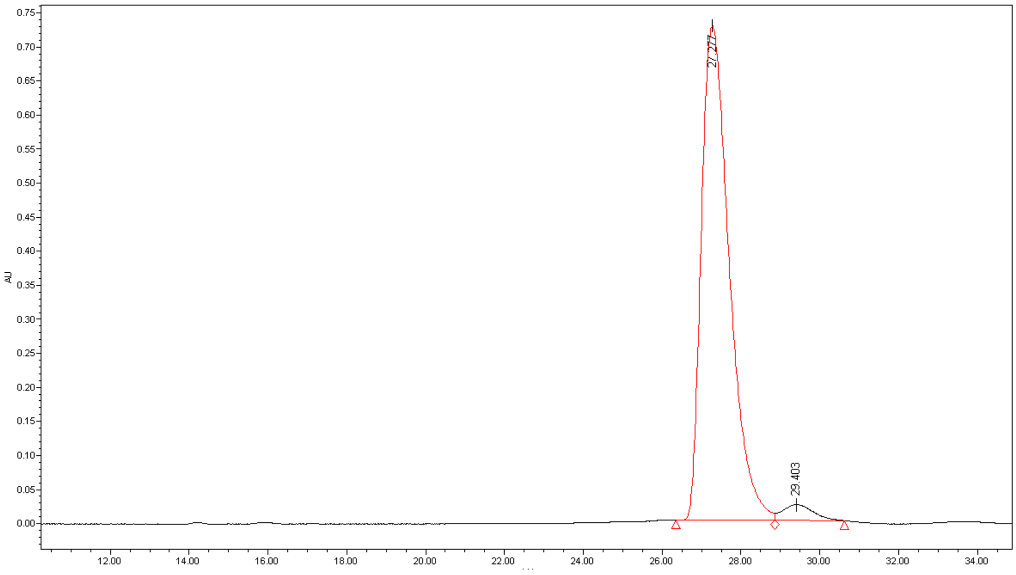

	Time/min	Area	Height	Area%
1	26.164	22488323	466399	95.46
2	28.709	1070176	19025	4.54

Figure S104, the HPLC spectrum of compound **2l**, related to **Scheme 2**

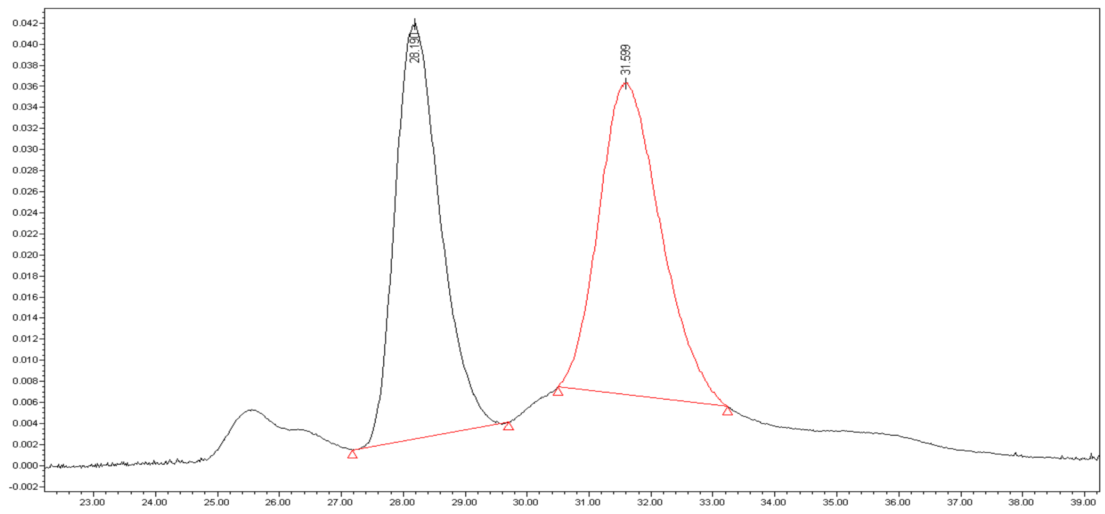

	Time/min	Area	Height	Area%
1	28.190	1993930	39346	49.06
2	31.599	2070135	29805	50.94

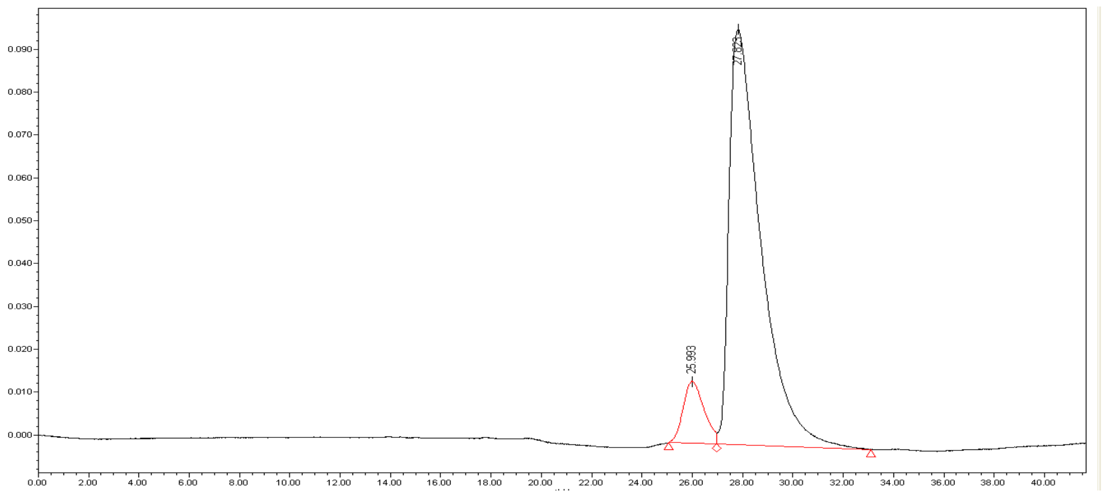

	Time/min	Area	Height	Area%
1	25.993	835540	14483	8.92
2	27.823	8531993	96905	91.08

Figure S105, the HPLC spectrum of compound **2m**, related to **Scheme 2**

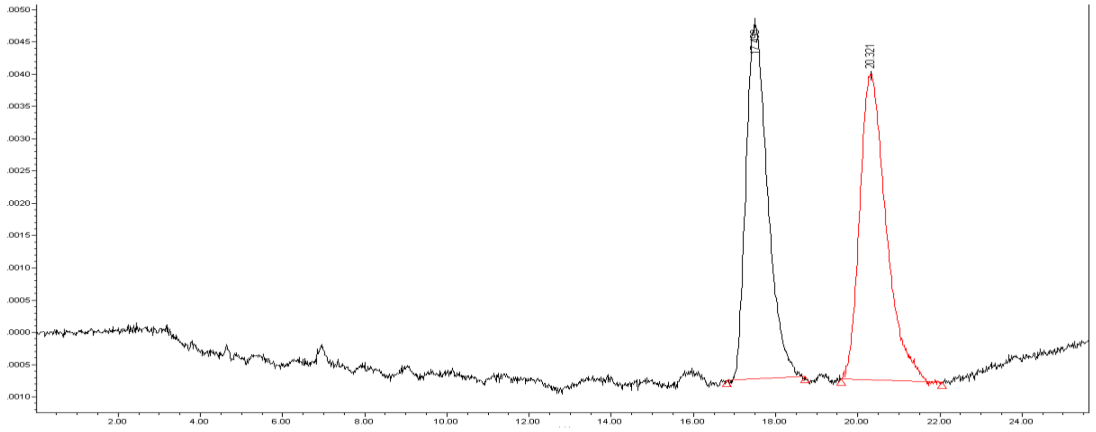

	Time/min	Area	Height	Area%
1	17.498	202433	5487	48.76
2	20.321	212717	4739	51.24

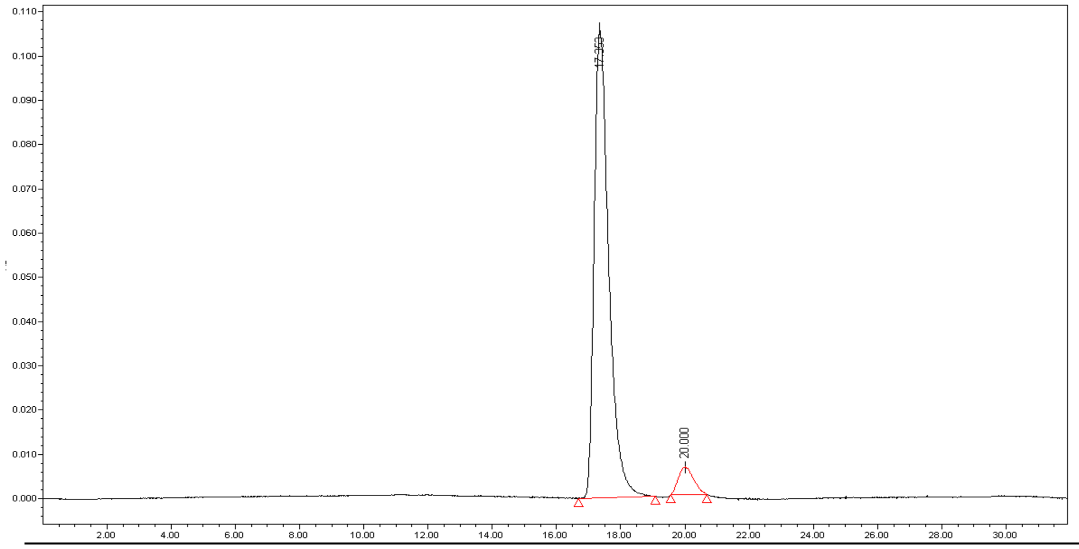

	Time/min	Area	Height	Area%
1	17.353	3337311	105949	92.24
2	20.000	204123	6238	5.76

Figure S106, the HPLC spectrum of compound **2n**, related to **Scheme 2**

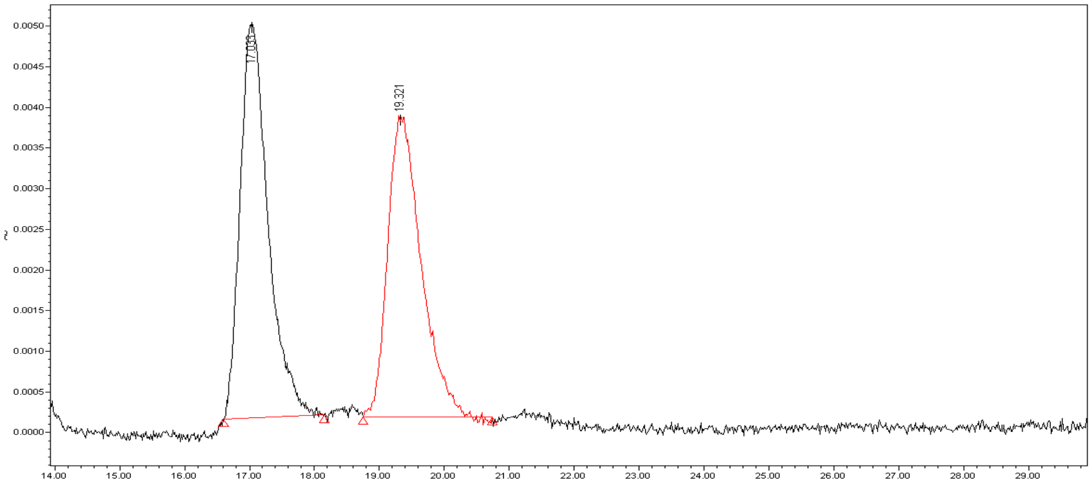

	Time/min	Area	Height	Area%
1	17.027	164795	5122	50.30
2	19.334	162811	3939	49.70

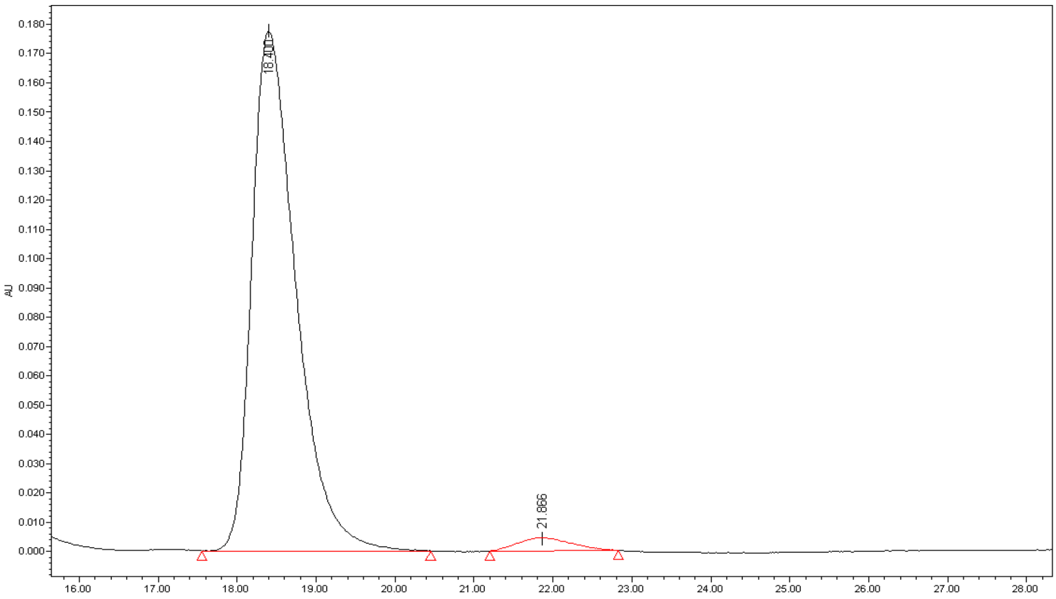

	Time/min	Area	Height	Area%
1	18.401	7131377	184341	97.26
2	21.860	200971	4212	2.74

Figure S107, the HPLC spectrum of compound **2o**, related to **Scheme 2**

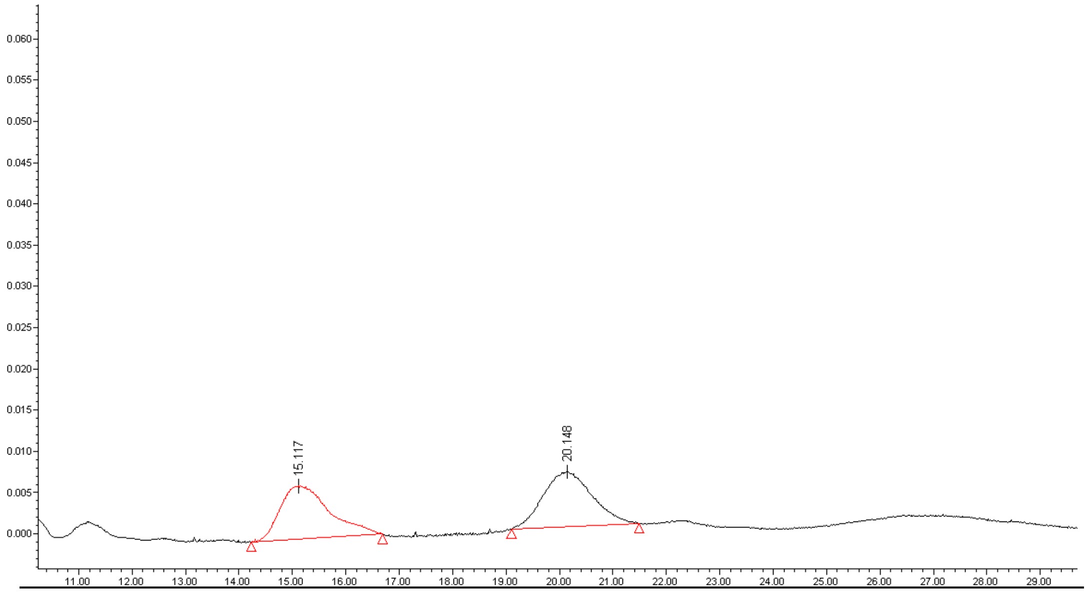

	Time/min	Area	Height	Area%
1	15.1117	410167	49.22	49.22
2	20.148	423245	6676	50.78

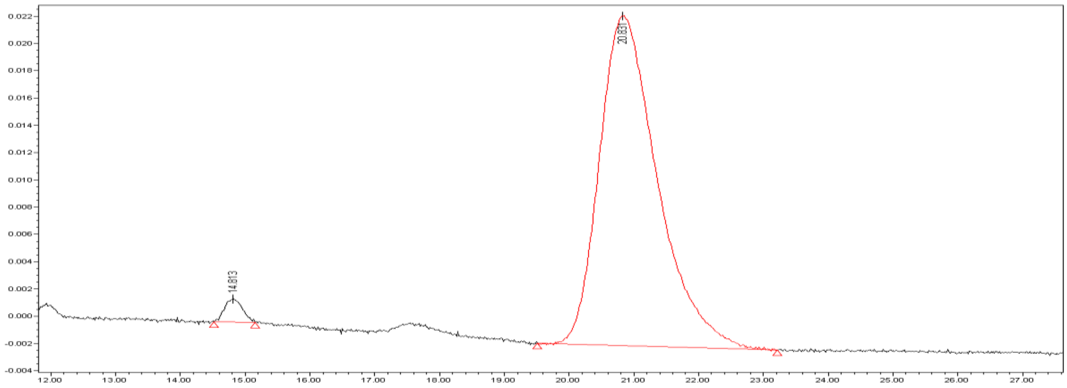

	Time/min	Area	Height	Area%
1	14.813	32464	1678	2.11
2	20.831	1506048	24186	97.89

Figure S108, the HPLC spectrum of compound **2p**, related to **Scheme 2**

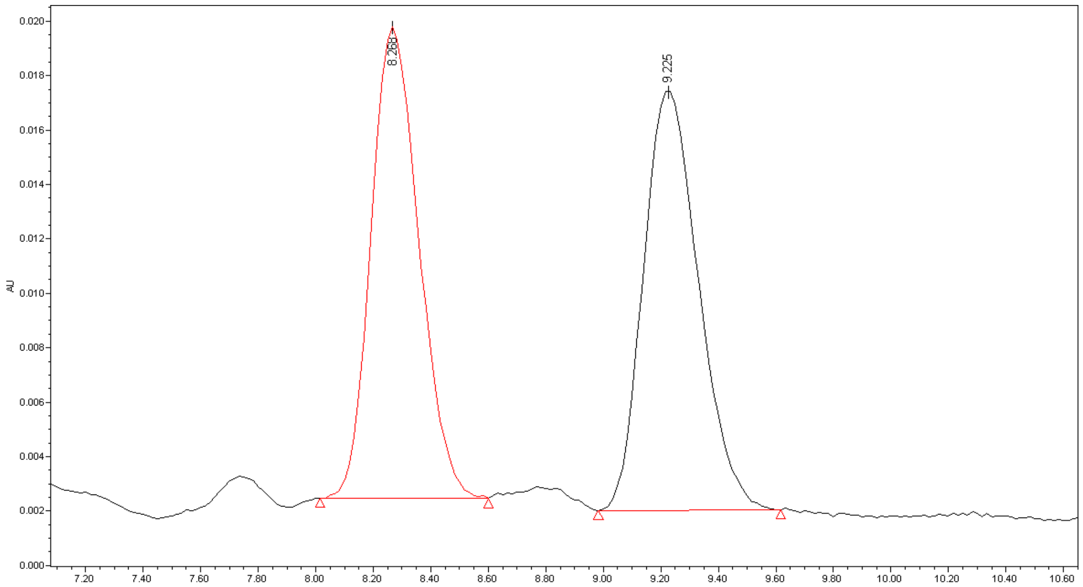

	Time/min	Area	Height	Area%
1	8.268	200472	17266	49.74
2	9.225	202602	15387	50.26

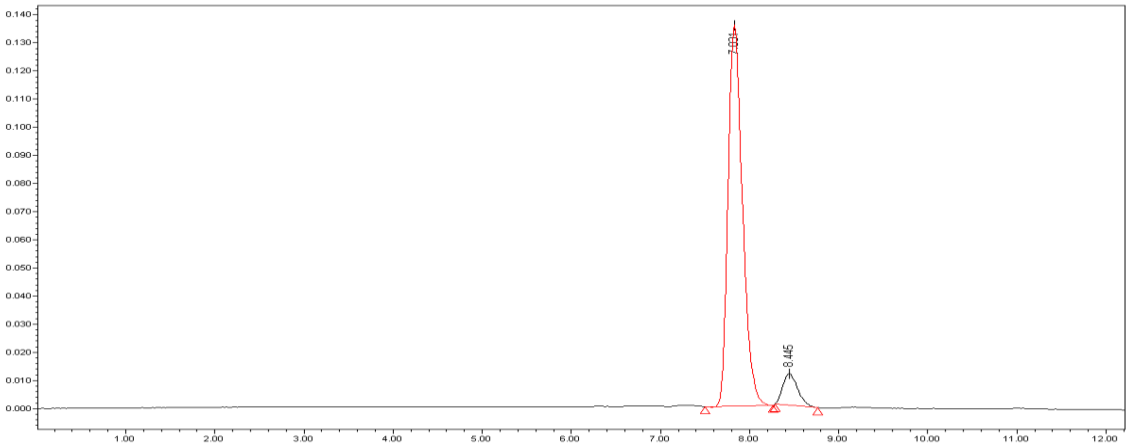

	Time/min	Area	Height	Area%
1	7.831	1527382	135633	92.00
2	8.445	132880	11364	8.00

Figure S109, the HPLC spectrum of compound **2q**, related to **Scheme 2**

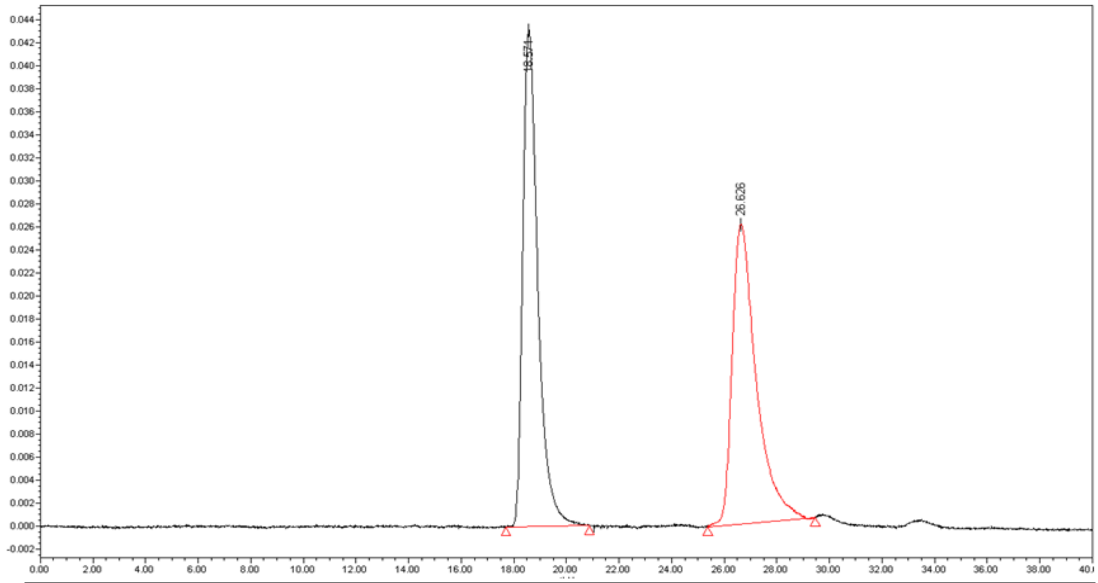

	Time/min	Area	Height	Area%
1	18.571	1716021	43100	50.95
2	26.626	1652184	26019	49.05

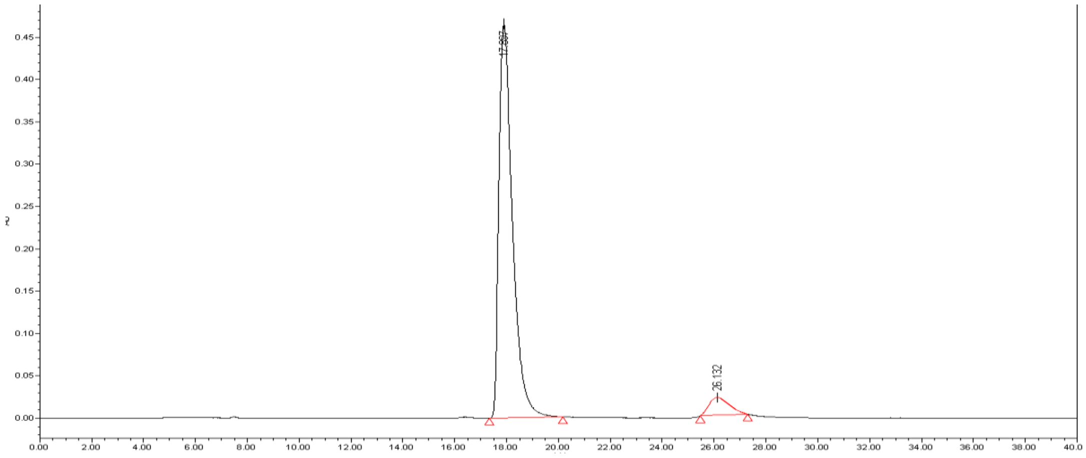

	Time/min	Area	Height	Area%
1	17.897	16892981	465377	93.60
2	26.132	1154284	21004	6.40

Figure S110, the HPLC spectrum of compound **2r**, related to **Scheme 2**

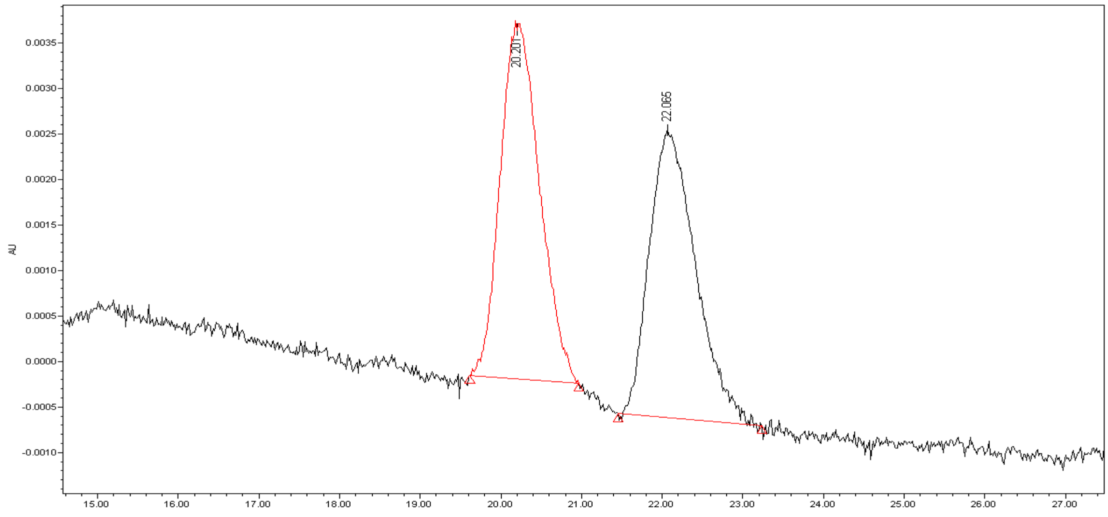

	Time/min	Area	Height	Area%
1	20.208	202251	5635	51.22
2	22.060	192607	4481	48.78

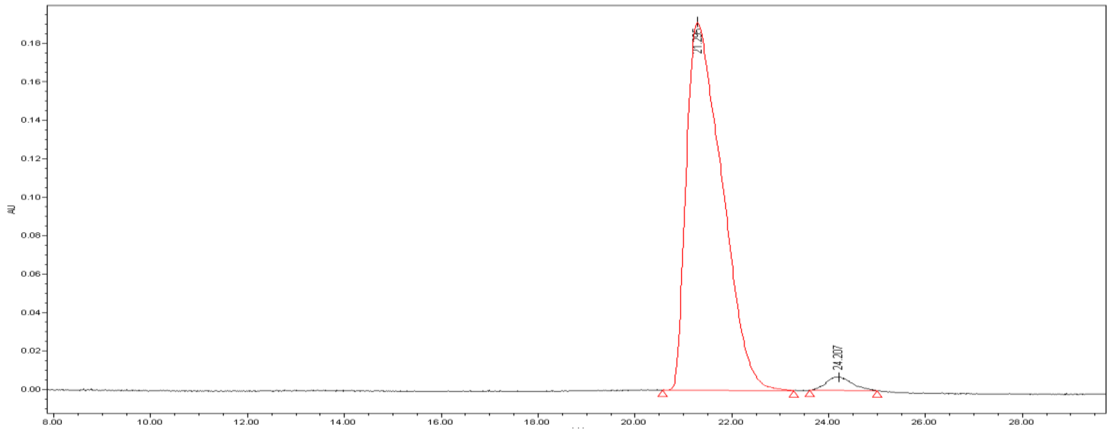

	Time/min	Area	Height	Area%
1	21.295	9808883	191494	97.19
2	24.207	283094	7215	2.81

Figure S111, the HPLC spectrum of compound **2s**, related to **Scheme 2**

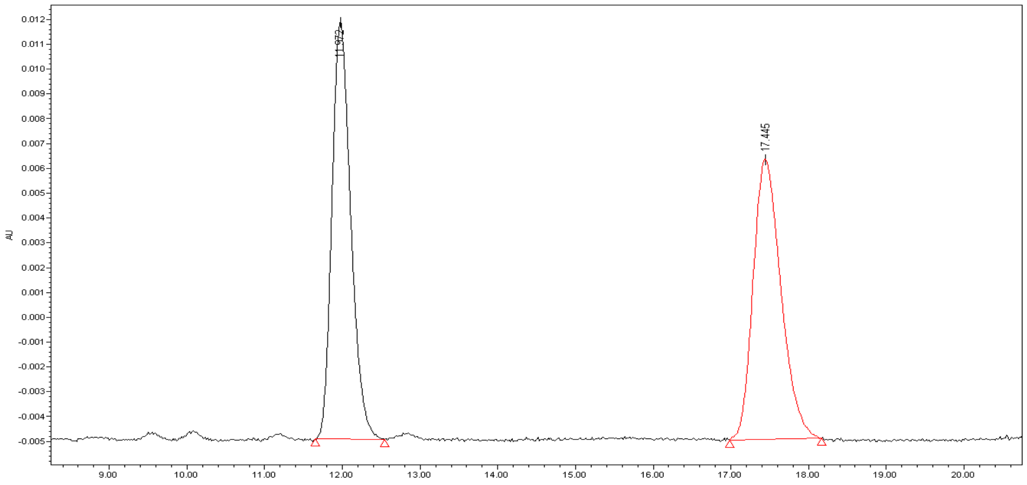

	Time/min	Area	Height	Area%
1	11.972	284419	16853	49.77
2	17.445	287023	11358	50.23

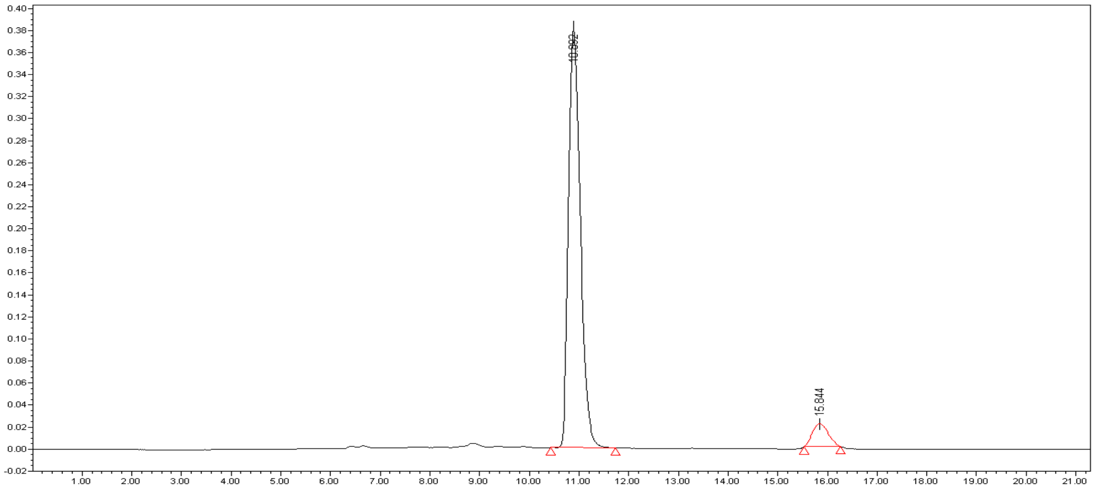

	Time/min	Area	Height	Area%
1	10.892	383689	383689	93.22
2	15.844	462586	20793	6.78

Figure S112, the HPLC spectrum of compound **2t**, related to **Scheme 2**

Transparent Methods

1. General information:

Unless specifically stated, all reagents were commercially obtained and where appropriate, purified prior to use. For example, all the aldehydes recrystallized or distilled prior to use. Dichloromethane, toluene, were freshly distilled from CaH_2, Ether (Et_2O), tetrahydrofuran (THF) and 1, 4-dioxane were dried and distilled from metal sodium and benzophenone. Alcohol solvents were dried and distilled from metal magnesium. Other commercially available reagents and solvents were used directly without purification. Reactions were monitored by thin layer chromatography (TLC) using silica gel plates. Flash column chromatography was performed over silica (300 - 400 mesh). ^{1}H, ^{13}C, ^{31}P, ^{19}F and ^{29}Si NMR spectra were recorded on a *Bruker* 400 MHz or 500 MHz spectrometer in $CDCl_3$. Multiplicities were given as: s (singlet); d (doublet); dd (doublets of doublet); t (triplet); q (quartet); td (triplet of doublets); tt (triplet of triplets) ddd (doublet of doublet of doublets) or m (multiplets). or m (multiplets). High resolution mass spectra (HRMS) of the products were obtained on a *Bruker* Daltonics micro TOF-spectrometer. HPLC was carried out with a *Agilent* 1260 infinity or Waters AcQuity UPLC using a chiralcel IA column, a chiral INA column (from *Phenomenex*) and a Chiralcel OD-H column.

2. General procedure for the synthesis of Si-tethered bisalkynes (Substrate 1)

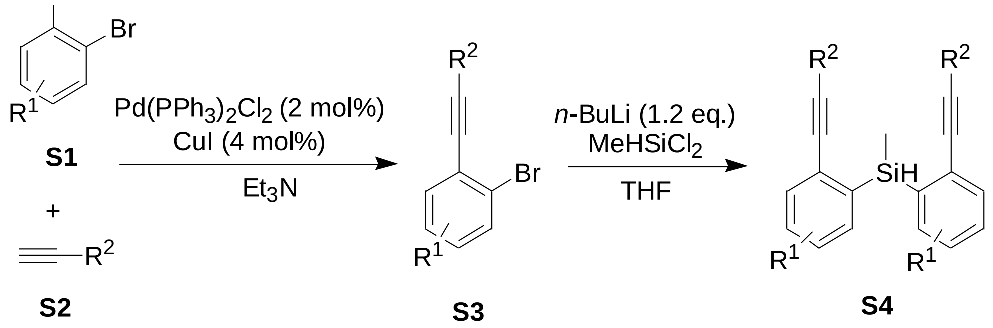

The synthesis of **S3**: A 50 mL single-necked, round-bottomed flask equipped with an egg-shaped magnetic stir bar is flame-dried under vacuum. After cooling to 23 ℃, $Pd(PPh_3)_2Cl_2$ (140 mg, 0.2 mmol, 2 mol %) and CuI (75 mg, 0.4 mmol, 4 mol %) is added, the reaction flask is put under an atmosphere of N_2, and **S1** (10 mmol), Et_3N (18 mL, 120 mmol, 1.8 equiv), and **S2** (10.2 g, 11 mmol, 1.1 equiv) is added via syringe resulting in a clear solution with a brown color. the reaction mixture is stirred at rt for 4 h. The solution was then diluted with EA (20 mL), and washed with water (2 × 20 mL) and brine (2 × 20 mL). The organics were then passed through a hydrophobic frit and concentrated under reduced pressure to give a yellow oil, which was purified by flash chromatography (silica gel, petroleum ether) to afford **S3**.

The synthesis of **S4** (substrate 1 in the text): A 50 mL single-necked, round-bottomed flask equipped with an egg-shaped magnetic stir bar is flame-dried under vacuum. After cooling to 23 ℃, **S3** is added, the reaction flask is put under an atmosphere of N_2, 5 ml (2.5 mmol) of a 2.5 M solution *n*-BuLi in hexanes was added at -78 ℃. The resulting solution was stirred at -78 ^{o}C for 1 h, and then 0.6 mL of $MeHSiCl_2$ (6 mmol, 0.6 equiv) was added slowly to the above mixture at the same temperature. The reaction mixture was stirred for 4 hours at rt. When the reaction is complete, it was quenched with saturated aqueous NH_4Cl (10 mL) and stirred vigorously for 5 minutes. The aqueous phase was extracted with ethyl acetate (3×40 mL). The combined organic layers were dried over Na_2SO_4 and concentrated under reduced pressure. Which was purified by flash chromatography (silica gel, petroleum ether) to afford **S4**.

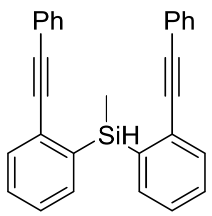

Methylbis(2-(phenylethynyl)phenyl)silane (1a)

White solid. mp 75.4-77.6 ^{o}C.

^{1}H NMR (400 MHz, CDCl$_3$) δ 7.57 (d, J = 7.5 Hz, 3H), 7.42 – 7.32 (m, 6H), 7.32 – 7.22 (m, 9H), 5.36 (q, J = 3.8 Hz, 1H), 0.82 (d, J = 3.8 Hz, 3H).

^{13}C NMR (100 MHz, CDCl$_3$) δ 137.9, 136.2, 132.1, 131.5, 129.6, 129.4, 128.4, 128.3, 127.7, 123.4, 93.0, 90.4, -5.0.

HRMS (ESI-TOF) m/z: [M+Na]$^+$ Calcd for $C_{29}H_{22}NaSi$, 421.1383; found 421.1390.

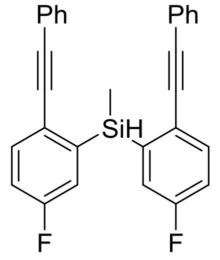

Bis(5-fluoro-2-(phenylethynyl)phenyl)(methyl)silane (1b)

White solid. mp 112.0-112.4 °C.

^{1}H NMR (400 MHz, CDCl$_3$) δ 7.55 (dd, J = 8.4, 5.2 Hz, 2H), 7.35 (dd, J = 6.7, 3.0 Hz, 4H), 7.30 (d, J = 5.3 Hz, 2H), 7.24 (s, 1H), 7.22 (d, J = 2.6 Hz, 1H), 7.06 (m, J = 8.5, 2.7 Hz, 2H), 5.30 (q, J = 3.6 Hz, 1H), 0.81 (d, J = 3.8 Hz, 3H).

^{13}C NMR (100 MHz, CDCl$_3$) δ 139.5, 136.2, 134.5, 132.7, 131.5, 129.2, 128.67, 128.4, 128.2, 123.6, 92.5, 90.6, 21.4, -4.7.

HRMS (ESI-TOF) m/z: [M+Na]$^+$ Calcd for $C_{29}H_{20}F_2NaSi$, 457.1195; found 457.1215.

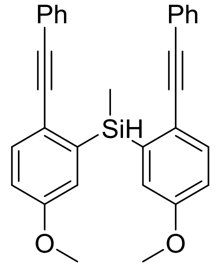

Bis(5-methoxy-2-(phenylethynyl)phenyl)(methyl)silane (1c)

White solid. mp 102.9-104.2 °C.

^{1}H NMR (400 MHz, CDCl$_3$) δ 7.51 (d, J = 8.5 Hz, 2H), 7.36 – 7.31 (m, 4H), 7.30 – 7.26 (m, 6H), 7.12 (d, J = 2.7 Hz, 2H), 6.88 (dd, J = 8.5, 2.7 Hz, 2H), 5.29 (q, J = 3.8 Hz, 1H), 3.65 (s, 6H), 0.82 (d, J = 3.8 Hz, 3H).

^{13}C NMR (100 MHz, CDCl$_3$) δ 159.0, 139.6, 133.7, 131.4, 128.4, 128.0, 123.7, 121.6, 121.31, 115.3, 91.4, 90.4, 55.2, -5.0.

HRMS (ESI-TOF) m/z: [M+Na]$^+$ Calcd for $C_{31}H_{26}NaO_2Si$, 481.1594; found 481.1611.

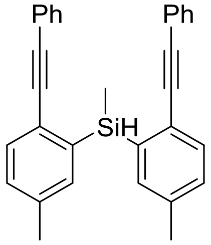

Methylbis(5-methyl-2-(phenylethynyl)phenyl)silane (1d)

Yellow oil.

^{1}H NMR (400 MHz, CDCl$_3$) δ 7.38 (s, 1H), 7.36 (s, 1H), 7.34 (d, J = 1.2 Hz, 2H), 7.28 – 7.24 (m, 3H), 7.22 – 7.20 (m, 3H), 7.19 (s, 4H), 7.08 (dd, J = 7.8, 1.2 Hz, 2H), 5.19 (q, J = 3.8 Hz, 1H), 2.12 (s, 6H), 0.73 (d, J = 3.9 Hz, 3H).

^{13}C NMR (100 MHz, CDCl$_3$) δ 137.8, 137.4, 137.1, 132.0, 131.5, 130.3, 128.3, 128.1, 126.2, 123.7, 92.1, 90.5, 21.5, -5.1.

HRMS (ESI-TOF) m/z: [M+Na]$^+$ Calcd for $C_{31}H_{26}NaSi$, 449.1696; found 449.1868.

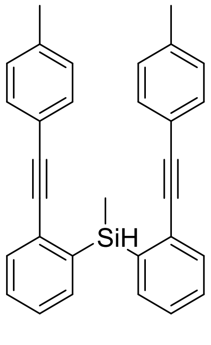

Methylbis(2-(*p*-tolylethynyl)phenyl)silane (1e)

Yellow solid. mp 88.1-92.2 ^{o}C.

^{1}H NMR (400 MHz, CDCl$_3$) δ 7.55 (dd, J = 5.1, 3.6 Hz, 4H), 7.36 (td, J = 7.6, 1.2 Hz, 2H), 7.26 – 7.21 (m, 6H), 7.09 (d, J = 8.0 Hz, 4H), 5.34 (q, J = 3.8 Hz, 1H), 2.34 (s, 6H), 0.81 (d, J = 3.9 Hz, 3H).

^{13}C NMR (100 MHz, CDCl$_3$) δ 138.5, 137.8, 136.2, 131.9, 131.4, 129.6, 129.5, 129.2, 127.5, 120.4, 93.2, 89.8, 21.6, -5.0.

HRMS (ESI-TOF) m/z: [M+H]$^+$ Calcd for $C_{31}H_{27}Si$, 427.1877; found 427.1861.

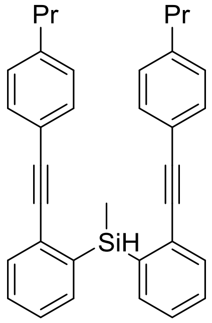

Methylbis(2-((4-propylphenyl)ethynyl)phenyl)silane (1f)

Yellow solid. mp 65.5-68.6 ^{o}C.

^{1}H NMR (400 MHz, CDCl$_3$) δ 7.56 (d, J = 7.9 Hz, 4H), 7.36 (t, J = 7.7 Hz, 2H), 7.26 (t, J = 4.0 Hz, 6H), 7.09 (d, J = 8.1 Hz, 4H), 5.34 (q, J = 3.8 Hz, 1H), 2.61 – 2.52 (m, 4H), 1.62 (dd, J = 15.1, 7.5 Hz, 4H), 0.93 (t, J = 7.3 Hz, 6H), 0.81 (d, J = 3.8 Hz, 3H).

^{13}C NMR (100 MHz, CDCl$_3$) δ 143.2, 137.8, 136.2, 132.0, 131.4, 129.6, 129.5, 128.6, 127.5, 120.6, 93.2, 89.9, 38.1, 24.5, 13.9, -4.9.

HRMS (ESI-TOF) m/z: [M+H]$^+$ Calcd for $C_{35}H_{35}Si$, 483.2503; found 483.2509.

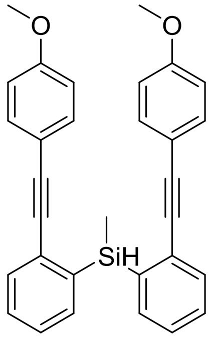

Bis(2-((4-methoxyphenyl)ethynyl)phenyl)(methyl)silane (1g)

White solid. mp 118.5-120.7 ^{o}C.

^{1}H NMR (400 MHz, CDCl$_3$) δ 7.61 – 7.52 (m, 4H), 7.36 (td, J = 7.6, 1.3 Hz, 2H), 7.28 (d, J = 2.0 Hz, 1H), 7.26 (s, 4H), 7.24 (d, J = 1.0 Hz, 1H), 6.81 (d, J = 8.9 Hz, 4H), 5.33 (q, J = 3.8 Hz, 1H), 3.80 (s, 6H), 0.80 (d, J = 3.9 Hz, 3H).

^{13}C NMR (100 MHz, CDCl$_3$) δ 159.7, 137.7, 136.2, 133.0, 131.8, 129.7, 129.5, 127.3, 115.6, 114.1, 93.0, 89.2, 55.4, -5.0.

HRMS (ESI-TOF) m/z: [M+H]$^+$ Calcd for $C_{31}H_{27}O_2Si$, 459.1775; found 459.1781.

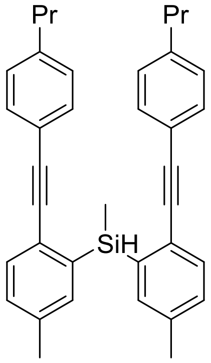

Methylbis(5-methyl-2-((4-propylphenyl)ethynyl)phenyl)silane (1h)

White solid. mp 105.5-106.3 °C.

^{1}H NMR (400 MHz, CDCl$_3$) δ 7.43 (s, 1H), 7.41 (s, 3H), 7.25 (s, 2H), 7.23 (s, 2H), 7.15 (d, *J* = 1.5 Hz, 1H), 7.13 (d, *J* = 1.3 Hz, 1H), 7.09 (d, *J* = 8.1 Hz, 4H), 5.24 (q, *J* = 3.8 Hz, 1H), 2.63 – 2.52 (m, 4H), 2.18 (s, 6H), 1.62 (d, *J* = 7.6 Hz, 4H), 0.93 (t, *J* = 7.3 Hz, 6H), 0.79 (d, *J* = 3.8 Hz, 3H).

^{13}C NMR (100 MHz, CDCl$_3$) δ 143.0, 137.7, 137.2, 137.1, 131.9, 131.4, 130.3, 128.5, 126.4, 120.9, 92.3, 89.9, 38.1, 24.5, 21.5, 13.9, -5.0.

HRMS (ESI-TOF) m/z: [M+H]$^+$ Calcd for $C_{37}H_{39}Si$, 511.2816; found 511.2821.

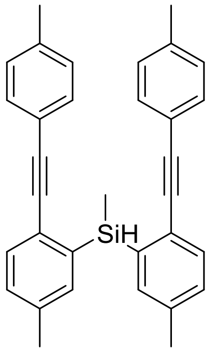

Methylbis(5-methyl-2-(*p*-tolylethynyl)phenyl)silane (1i)

Yellow solid. mp 126.0-130.1 °C.

^{1}H NMR (400 MHz, CDCl$_3$) δ 7.36 (s, 1H), 7.33 (d, *J* = 2.4 Hz, 3H), 7.15 (d, *J* = 8.3 Hz, 4H), 7.10 – 7.03 (m, 2H), 7.01 (d, *J* = 7.9 Hz, 4H), 5.17 (q, *J* = 3.7 Hz, 1H), 2.25 (s, 6H), 2.12 (s, 6H), 0.71 (d, *J* = 3.8 Hz, 3H).

^{13}C NMR (100 MHz, CDCl$_3$) δ 138.2, 138.0, 137.7, 137.6, 137.2, 137.2, 137.0, 135.4, 131.9, 131.8, 131.4, 131.1, 130.3, 130.1, 129.1, 127.2, 126.3, 125.6, 120.6, 96.1, 92.2, 91.6, 89.9, 21.6, 21.6, 21.5, -5.0.

HRMS (ESI-TOF) m/z: [M+Na]$^+$ Calcd for $C_{19}H_{30}N_{12}NaSi$, 477.2378; found 477.2360.

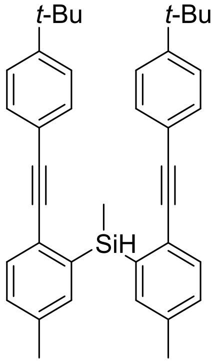

Bis(2-((4-(*tert*-butyl)phenyl)ethynyl)-5-methylphenyl)(methyl)silane (1j)

Yellow solid. mp 116.7-119.1 ^{o}C.

^{1}H NMR (400 MHz, CDCl$_3$) δ 7.44 (s, 1H), 7.42 (s, 1H), 7.40 (s, 2H), 7.29 (q, *J* = 8.5 Hz, 8H), 7.13 (d, *J* = 7.8 Hz, 2H), 5.25 (q, *J* = 3.7 Hz, 1H), 2.16 (s, 6H), 1.30 (s, 18H), 0.80 (d, *J* = 3.8 Hz, 3H).

^{13}C NMR (100 MHz, CDCl$_3$) δ 151.3, 137.8, 137.2, 137.1, 131.9, 131.3, 130.3, 126.4, 125.3, 120.7, 92.217, 879.927, 34.9, 31.3, 21.5, -5.0.

HRMS (ESI-TOF) m/z: [M+Na]$^+$ Calcd for $C_{39}H_{42}NaSi$, 561.2948; found 561.2960.

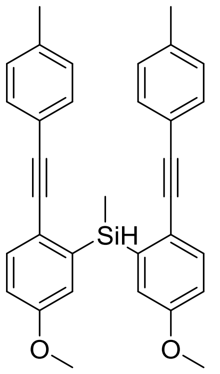

Bis(5-methoxy-2-(*p*-tolylethynyl)phenyl)(methyl)silane (1k)

Yellow solid. mp 117.3-118.1 ^{o}C.

^{1}H NMR (400 MHz, CDCl$_3$) δ 7.39 (s, 1H), 7.36 (s, 1H), 7.14 (s, 2H), 7.12 (s, 2H), 7.02 (d, J = 2.7 Hz, 2H), 6.96 (s, 2H), 6.94 (s, 2H), 6.75 (d, J = 2.7 Hz, 1H), 6.73 (d, J = 2.7 Hz, 1H), 5.20 (q, J = 3.7 Hz, 1H), 3.50 (s, 6H), 2.19 (s, 6H), 0.72 (d, J = 3.8 Hz, 3H).

^{13}C NMR (100 MHz, CDCl$_3$) δ 158.8, 139.5, 138.0, 133.5, 131.2, 129.1, 121.6, 121.5, 120.6, 115.2, 91.6, 89.8, 55.1, 21.5, -5.1.

HRMS (ESI-TOF) m/z: [M+Na]$^+$ Calcd for $C_{33}H_{30}NaO_2Si$, 509.1907; found 509.1920.

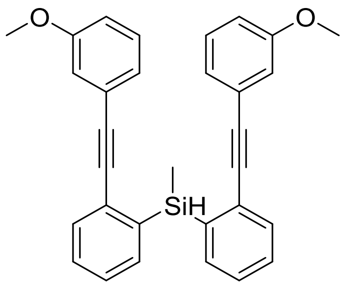

Bis(2-((3-methoxyphenyl)ethynyl)phenyl)(methyl)silane (1l)

Yellow solid. mp 92.9-94.2. ^{o}C.

^{1}H NMR (400 MHz, CDCl$_3$) δ 7.71 – 7.47 (m, 4H), 7.39 (dd, J = 10.6, 4.4 Hz, 2H), 7.33 – 7.27 (m, 2H), 7.24 – 7.17 (m, 2H), 7.03 – 6.93 (m, 2H), 6.92 – 6.82 (m, 4H), 5.38 (dd, J = 7.5, 3.7 Hz, 1H), 3.77 (s, 6H), 0.83 (d, J = 3.9 Hz, 3H).

^{13}C NMR (100 MHz, CDCl$_3$) δ 159.3, 137.8, 136.0, 131.9, 129.5, 129.3, 129.2 127.6, 124.2, 124.0, 115.9, 115.1, 92.8, 90.1, 55.3, -5.1.

HRMS (ESI-TOF) m/z: [M+Na]$^+$ Calcd for $C_{31}H_{26}NaO_2Si$, 481.1594; found 481.1611.

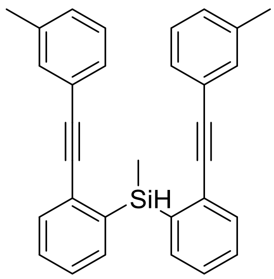

Methylbis(2-(*m*-tolylethynyl)phenyl)silane (1m)

Yellow oil

^{1}H NMR (400 MHz, CDCl$_3$) δ 7.61 (d, J = 7.9 Hz, 4H), 7.41 (td, J = 7.6, 1.1 Hz, 2H), 7.31 (dd, J = 11.0, 3.7 Hz, 2H), 7.21 (d, J = 6.1 Hz, 4H), 7.15 (dd, J = 8.3, 5.8 Hz, 4H), 5.41 (q, J = 3.8 Hz, 1H), 2.34 (s, 6H), 0.85 (d, J = 3.8 Hz, 3H).

^{13}C NMR (100 MHz, CDCl$_3$) δ 138.0, 137.9, 136.2, 132.1, 132.0, 129.6, 129.5, 129.2, 128.55, 128.3, 127.6, 123.2, 93.2, 90.1, 21.3, -5.0.

HRMS (ESI-TOF) m/z: [M+Na]$^+$ Calcd for $C_{31}H_{26}NaSi$, 449.1696; found 449.1710.

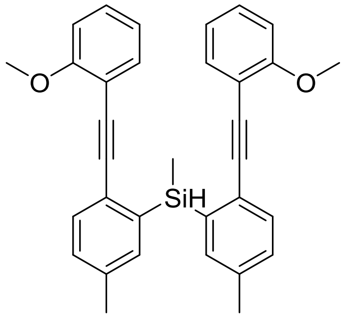

Bis(2-((2-methoxyphenyl)ethynyl)-5-methylphenyl)(methyl)silane (1n)

White solid. mp 102.3-103.8 °C.

^{1}H NMR (400 MHz, CDCl$_3$) δ 7.72 – 7.52 (m, 4H), 7.41 (d, J = 7.0 Hz, 2H), 7.33 (t, J = 7.3 Hz, 2H), 7.23 (d, J = 7.2 Hz, 2H), 7.05 – 6.85 (m, 4H), 5.88 – 5.02 (m, 1H), 3.90 (s, 6H), 2.27 (s, 6H), 1.02 (d, J = 2.1 Hz, 1H).

^{13}C NMR (100 MHz, CDCl$_3$) δ 159.9, 137.7, 137.1, 133.4, 132.0, 130.1, 129.5, 126.5, 120.4, 112.9, 110.6, 94.5, 88.5, 55.5, 21.3, -5.2.

HRMS (ESI-TOF) m/z: [M+Na]$^+$ Calcd for $C_{33}H_{30}NaO_2Si$, 509.1907; found 509.1919.

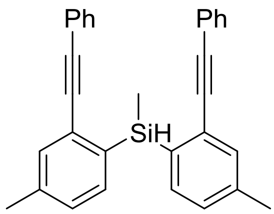

Methylbis(4-methyl-2-(phenylethynyl)phenyl)silane (1o)

Yellow oil.

^{1}H NMR (400 MHz, CDCl$_3$) δ 7.45 (d, *J* = 7.6 Hz, 2H), 7.42 (s, 2H), 7.35 (dd, *J* = 6.6, 3.1 Hz, 4H), 7.31 – 7.27 (m, 6H), 7.08 (d, *J* = 7.5 Hz, 2H), 5.33 (q, *J* = 3.7 Hz, 1H), 2.34 (s, 6H), 0.79 (d, *J* = 3.8 Hz, 3H).

^{13}C NMR (100 MHz, CDCl$_3$) δ 139.5, 136.2, 134.5, 132.7, 131.5, 129.2, 128.7, 128.4, 128.2, 123.6, 92.5, 90.6, 21.37, -4.7.

HRMS (ESI-TOF) m/z: [M+Na]$^+$ Calcd for $C_{31}H_{26}NaSi$, 449.1696; found 449.1703.

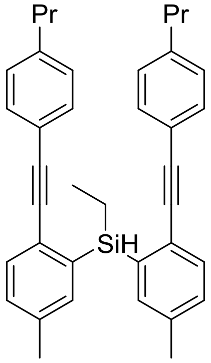

Ethylbis(5-methyl-2-((4-propylphenyl)ethynyl)phenyl)silane (1p)

Yellow oil.

^{1}H NMR (400 MHz, CDCl$_3$) δ 7.35 (s, 1H), 7.18 (d, *J* = 1.6 Hz, 2H), 7.04 (t, *J* = 9.5 Hz, 1H), 5.05 (t, *J* = 3.9 Hz, 1H), 2.58 – 2.44 (m, 1H), 2.10 (s, 1H), 1.54 (dd, *J* = 12.6, 5.0 Hz, 2H), 1.32 (ddd, *J* = 11.5, 7.7, 3.8 Hz, 1H), 1.37 – 1.25 (m, 1H), 1.02 (t, *J* = 7.8 Hz, 1H), 0.87 (dd, *J* = 9.8, 4.8 Hz, 2H).

^{13}C NMR (100 MHz, CDCl$_3$) δ 143.0, 137.4, 137.1, 137.0, 132.0, 131.4, 130.2, 128.5, 126.5, 120.9, 92.1, 90.0, 38.1, 24.5, 21.5, 13.9, 8.9, 3.6.

HRMS (ESI-TOF) m/z: [M+Na]$^+$ Calcd for $C_{38}H_{40}NaSi$, 547.2791; found 547.2784.

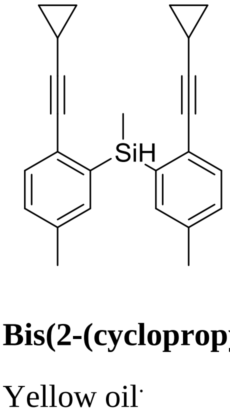

Bis(2-(cyclopropylethynyl)-5-methylphenyl)(methyl)silane (1q)

Yellow oil·

^{1}H NMR (400 MHz, CDCl$_3$) δ 7.22 (d, *J* = 7.5 Hz, 4H), 7.05 – 7.00 (m, 2H), 4.94 (q, *J* = 3.8 Hz, 1H), 2.22 (s, 6H), 1.29 – 1.18 (m, 2H), 0.68 – 0.62 (m, 4H), 0.59 (d, *J* = 3.8 Hz, 3H), 0.55 – 0.48 (m, 2H), 0.48 – 0.41 (m, 2H).

^{13}C NMR (100 MHz, CDCl$_3$) δ 136.7, 135.6, 135.3, 130.8, 129.1, 126.0, 95.2, 20.6, 7.3, 7.3, -0.7, -6.1.

HRMS (ESI-TOF) m/z: [M+Na]$^+$ Calcd for $C_{25}H_{26}NaSi$, 377.1696; found 377.1709.

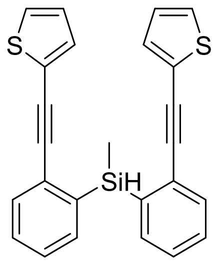

Methylbis(2-(thiophen-2-ylethynyl)phenyl)silane (1r)

White solid. mp 70.5-72.3 ^{o}C.

^{1}H NMR (400 MHz, CDCl$_3$) δ 7.53 (d, *J* = 7.3 Hz, 2H), 7.45 (d, *J* = 7.6 Hz, 2H), 7.28 (dd, *J* = 7.5, 6.7 Hz, 2H), 7.18 (dd, *J* = 13.9, 6.2 Hz, 4H), 7.03 (d, *J* = 3.6 Hz, 2H), 6.88 (dd, *J* = 5.0, 3.7 Hz, 2H), 5.19 (q, *J* = 3.6 Hz, 1H), 0.73 (d, *J* = 3.9 Hz, 3H).

^{13}C NMR (100 MHz, CDCl$_3$) δ 137.7, 136.2, 131.8, 129.6, 128.9, 127.8, 127.4, 127.2, 123.4, 94.1, 86.3, -5.1.

HRMS (ESI-TOF) m/z: [M+Na]$^+$ Calcd for $C_{25}H_{18}NaS_2Si$, 433.0511; found 433.0520.

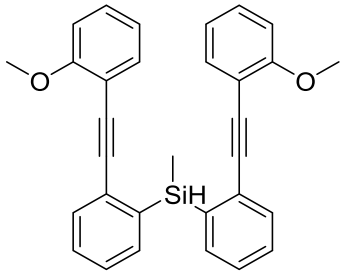

Bis(2-((2-methoxyphenyl)ethynyl)phenyl)(methyl)silane (1s)

Yellow oil.

^{1}H NMR (400 MHz, $CDCl_3$) δ 7.52 (dd, *J* = 14.5, 7.2 Hz, 4H), 7.27 (td, *J* = 7.6, 1.2 Hz, 2H), 7.24 – 7.11 (m, 6H), 6.79 (t, *J* = 8.3 Hz, 4H), 5.31 (q, *J* = 3.8 Hz, 1H), 3.75 (s, 6H), 0.79 (d, *J* = 3.9 Hz, 3H).

^{13}C NMR (100 MHz, $CDCl_3$) δ 160.1, 137.9, 136.3, 133.6, 132.2, 129.8, 129.7, 129.4, 127.4, 120.5, 112.8, 110.7, 94.4, 89.4, 55.7, -5.1.

HRMS (ESI-TOF) m/z: [M+Na]$^+$ Calcd for $C_{31}H_{26}NaO_2Si$, 481.1594; found 481.1609.

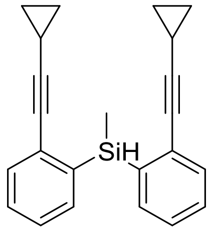

Bis(2-(cyclopropylethynyl)phenyl)(methyl)silane (1t)

Yellow oil.

^{1}H NMR (400 MHz, $CDCl_3$) δ 7.48 (d, *J* = 7.3 Hz, 2H), 7.44 (d, *J* = 7.6 Hz, 2H), 7.34 (td, *J* = 7.5, 1.3 Hz, 2H), 7.29 – 7.22 (m, 2H), 5.11 (q, *J* = 3.8 Hz, 1H), 1.36 (m, *J* = 9.9, 6.6, 4.2 Hz, 2H), 0.78 (m, *J* = 5.8, 3.0 Hz, 4H), 0.73 (d, *J* = 3.9 Hz, 3H), 0.69 – 0.63 (m, 2H), 0.60 – 0.53 (m, 2H).

^{13}C NMR (100 MHz, $CDCl_3$) δ 137.8, 135.8, 131.8, 130.1, 129.3, 126.8, 97.2, 8.4, 0.3, -5.2.

HRMS (ESI-TOF) m/z: [M+Na]$^+$ Calcd for $C_{23}H_{22}NaSi$, 349.1383; found 349.1400.

For the chiral Ligand (L8)[3]:

3-(diphenylphosphanyl)-2'-(hydroxy(*o*-tolyl)methyl)-[1,1'-binaphthalen]-2-ol

Colorless solid.

Optical rotation: $[\alpha]_D^{25}$ = +34.5 (c = 0.63, $CHCl_3$).

^{1}H NMR (400 MHz, $CDCl_3$) δ 7.80 (dd, J = 8.3, 3.0 Hz, 2H), 7.60 (t, J = 7.0 Hz, 2H), 7.35 (ddd, J = 22.6, 12.3, 5.7 Hz, 12H), 7.25 (d, J = 8.7 Hz, 1H), 7.22 – 7.14 (m, 4H),

7.10 – 7.00 (m, 3H), 6.84 (dd, J = 13.6, 7.9 Hz, 2H), 6.61 (s, 1H), 5.81 (s, 1H), 1.56 (s, 3H).

^{13}C NMR (100 MHz, $CDCl_3$) δ 153.00, 140.34, 139.76, 134.92, 134.44, 134.33, 134.14, 133.83, 133.64, 133.54, 133.10, 131.58, 130.14, 129.31, 128.82, 128.76, 128.75, 128.69, 128.24, 128.15, 127.27, 126.92, 126.65, 126.40, 126.35, 126.19, 125.94, 125.47, 124.91, 123.95, 71.45, 19.44.

3. General procedure for the synthesis of product 2

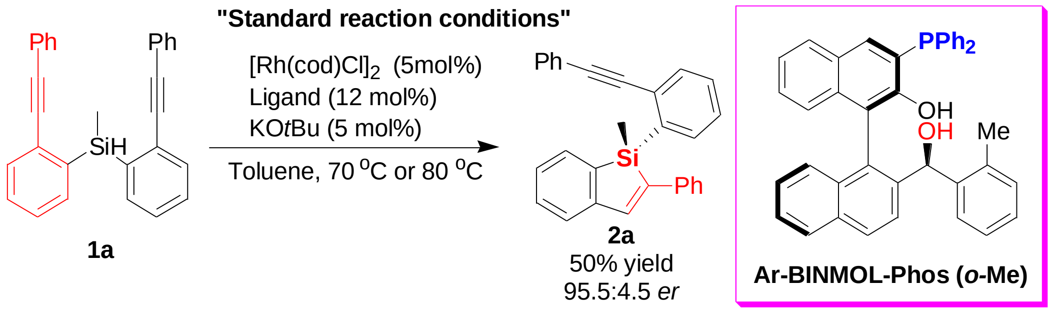

General procedure for the enantioselective synthesis of benzosilole 2 via Rh-catalyzed intramolecular hydrosilylation. A vial was charged with $[Rh(cod)Cl]_2$ (4.9 mg, 5 mol%), **L8** (13.8 mg, 12 mol%), KO*t*Bu (2.7 mg, 12 mol%) and evacuated under high vacuum and backfilled with N_2. Then toluene (1 mL) was added and stirred at room temperature for about 0.5 h. Subsequently, the methylbis(2-(phenylethynyl)phenyl)silane **1a** (79.6 mg, 0.2 mmol) were added to the reaction mixture and was stirred at 70 ℃. Upon reaction completion (72 h, TLC, eluent: hexane), the mixture was filtered over a plug of silica gel (washed with 20 mL EtOAc), and the filtrate was concentrated. The crude was purified by column chromatography to give the products **2**.

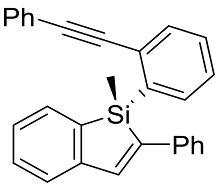

(*S*)-1-methyl-2-phenyl-1-(2-(phenylethynyl)phenyl)-1*H*-benzo[*b*]silole (2a)

The mobile phase for flash chromatography: hexane.Yellow oil. (40.1 mg, 50%).

Optical rotation: $[\alpha]_D^{25}$ = +221.5 (c = 0.63, $CHCl_3$).

^{1}H NMR (400 MHz, $CDCl_3$) δ 7.90 (d, J = 6.9 Hz, 1H), 7.70 (s, 1H), 7.58 (t, J = 7.0 Hz, 3H), 7.51 (dd, J = 6.5, 3.1 Hz, 2H), 7.46 (d, J = 7.4 Hz, 1H), 7.35 (dd, J = 5.0, 1.6 Hz, 3H), 7.33 – 7.30 (m, 3H), 7.29 – 7.20 (m, 3H), 7.19 – 7.11 (m, 2H).

^{13}C NMR (100 MHz, CDCl$_3$) δ 149.1, 143.4, 142.4, 138.8, 137.6, 137.4, 135.6, 132.8, 132.7, 132.6, 131.5, 131.3, 130.2, 129.8, 129.3, 128.8, 128.5, 128.2, 128.1, 127.8, 127.4, 127.2, 127.1, 127.0, 124.6, 123.2, 92.2, 91.3, -3.6.

HRMS: (ESI-TOF) m/z: [M+H]$^+$ Calcd for $C_{29}H_{23}Si$, 399.1564; found 399.1567.
HPLC: Enantiomeric excess was determined by HPLC with a Chiralcel OD-H column and a Phenomenex column (hexanes: 2-propanol = 99.5:0.5, 0.5 mL/min, 330 nm, 95.5:4.5 *er*); major enantiomer t_r = 26.8 min, minor enantiomer t_r = 30.0 min.

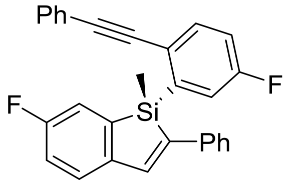

(*R*)-6-fluoro-1-(5-fluoro-2-(phenylethynyl)phenyl)-1-methyl-2-phenyl-1*H*-benzo[*b*]silole (2b)

The mobile phase for flash chromatography: hexane. Yellow oil. (32.1 mg, 37%).
Optical rotation: $[\alpha]_D^{25}$ = +153.6 (c = 0.33, CHCl$_3$).
^{1}H NMR (400 MHz, CDCl$_3$) δ 7.65 (s, 1H), 7.61 – 7.52 (m, 3H), 7.52 – 7.46 (m, 3H), 7.38 (dd, *J* = 5.9, 2.6 Hz, 3H), 7.34 (d, *J* = 7.8 Hz, 2H), 7.21 (dd, *J* = 8.2, 4.8 Hz, 1H), 7.14 (dd, *J* = 8.7, 2.8 Hz, 1H), 7.08 – 6.94 (m, 3H), 0.98 (s, 3H).
^{13}C NMR (100 MHz, CDCl$_3$) δ 163.8, 163.4, 161.3, 160.9, 145.0, 142.4, 141.8, 140.0, 140.0, 139.9, 138.4, 135.0, 134.9, 131.6, 129.0, 128.8, 128.7, 127.6, 126.9, 125.9, 125.9, 125.9, 125.4, 123.0, 122.5, 122.2, 120.1, 119.9, 117.4, 117.2, 117.0, 116.8, 92.1, 90.0, -3.9.
HRMS (ESI-TOF) m/z: [M+Na]$^+$ Calcd for $C_{29}H_{20}F_2NaSi$, 457.1195; found 457.1120.
HPLC: Enantiomeric excess was determined by HPLC with a Phenomenex column and a Chiralcel OD-H column (hexanes: 2-propanol = 95.5:0.5, 0.5 mL/min, 330 nm, 96.1:3.9 *er*); major enantiomer t_r = 46.6 min, minor enantiomer t_r = 48.5 min.

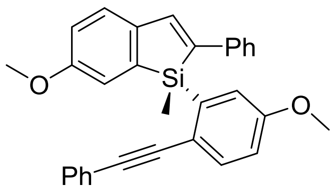

(*S*)-6-methoxy-1-(5-methoxy-2-(phenylethynyl)phenyl)-1-methyl-2-phenyl-1*H*-benzo[*b*]silole (2c)

The mobile phase for flash chromatography: hexane/ethyl acetate = 100:1. White solid. mp 74-76 ^{o}C. (55 mg, 60%).

Optical rotation: $[\alpha]_D^{25}$ = +391.5 (c = 1.63, $CHCl_3$).

^{1}H NMR (400 MHz, $CDCl_3$) δ 7.58 (s, 1H), 7.46 (t, *J* = 8.1 Hz, 6H), 7.24 (dd, *J* = 11.0, 7.3 Hz, 4H), 7.12 (dd, *J* = 7.6, 5.1 Hz, 3H), 6.88 (d, *J* = 2.7 Hz, 1H), 6.73 (ddd, *J* = 13.7, 8.4, 2.6 Hz, 2H), 3.45 (s, 3H), 3.42 (s, 3H), 0.93 (s, 3H).

^{13}C NMR (100 MHz, $CDCl_3$) δ 159.3, 159.0, 142.5, 142.0, 140.0, 140.0, 139.3, 134.3, 131.5, 128.9, 128.6, 128.3, 126.9, 126.9, 125.6, 124.8, 123.6, 121.2, 120.9, 118.4, 116.0, 115.8, 91.8, 90.9, 77.5, 76.8, 55.2, 55.0, -3.3.

HRMS (ESI-TOF) m/z: [M+Na]$^+$ Calcd for $C_{31}H_{26}NaO_2Si$, 481.1592; found 481.1581.

HPLC: Enantiomeric excess was determined by HPLC with a Chiralpak IA column (hexanes: 2-propanol = 99.3:0.7, 0.6 mL/min, 330 nm, 96.7:3.3 *er*); major enantiomer t_r = 19.3 min, minor enantiomer t_r = 26.5 min.

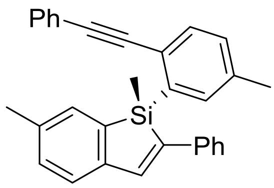

(*R*)-1,6-dimethyl-1-(5-methyl-2-(phenylethynyl)phenyl)-2-phenyl-1*H*-benzo[*b*]silole (2d)

The mobile phase for flash chromatography: hexane. Yellow oil. (34.9 mg, 41%).

Optical rotation: $[\alpha]_D^{25}$ = +223.5 (c = 0.67, $CHCl_3$).

^{1}H NMR (400 MHz, CDCl$_3$) δ 7.63 (s, 1H), 7.58 (s, 1H), 7.49 – 7.37 (m, 5H), 7.28 – 7.16 (m, 5H), 7.06 (ddd, J = 22.1, 15.3, 7.3 Hz, 5H), 2.14 (s, 3H), 2.08 (s, 3H), 0.86 (s, 3H).

^{13}C NMR (100 MHz, CDCl$_3$) δ 146.6, 142.5, 142.4, 139.2, 137.9, 137.9, 137.4, 136.8, 136.4, 133.9, 132.8, 131.6, 130.8, 130.7, 128.8, 128.5, 128.4, 127.0, 126.3, 124.5, 123.6, 91.7, 91.6, 21.6, 21.5, -3.5.

HRMS (ESI-TOF) m/z: [M+Na]$^+$ Calcd for $C_{31}H_{26}NaSi$, 449.1696; found 449.1708.

HPLC: Enantiomeric excess was determined by HPLC with a Chiralpak IA column (hexanes: 2-propanol = 99.5:0.5, 0.8 mL/min, 330 nm, 99.3:0.7 *er*); major enantiomer t_r = 10.0 min, minor enantiomer t_r = 11.1 min.

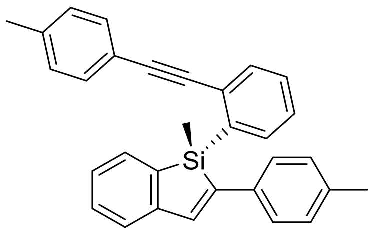

(*S*)-1-methyl-2-(*p*-tolyl)-1-(2-(*p*-tolylethynyl)phenyl)-1*H*-benzo[*b*]silole (2e)

The mobile phase for flash chromatography: hexane. Yellow oil. (34.9 mg, 41%).

Optical rotation: $[\alpha]_D^{25}$ = +321.5 (c = 1.33, CHCl$_3$).

^{1}H NMR (400 MHz, CDCl$_3$) δ 7.83 (d, J = 7.0 Hz, 1H), 7.60 (s, 1H), 7.50 (d, J = 7.7 Hz, 1H), 7.41 (d, J = 8.0 Hz, 2H), 7.36 (d, J = 8.1 Hz, 3H), 7.27 – 7.17 (m, 3H), 7.06 (ddd, J = 14.5, 9.9, 4.9 Hz, 6H), 2.31 (s, 3H), 2.27 (s, 3H), 0.89 (s, 3H).

^{13}C NMR (100 MHz, CDCl$_3$) δ 149.4, 143.4, 141.5, 138.8, 137.7, 137.5, 137.2, 136.0, 135.7, 132.9, 132.7, 131.6, 130.3, 129.8, 129.6, 129.6, 129.4, 127.8, 127.1, 127.0, 124.5, 120.3, 92.5, 90.9, 21.7, 21.4, -3.5.

HRMS (ESI-TOF) m/z: [M+Na]$^+$ Calcd for $C_{31}H_{26}NaSi$, 449.1686; found 449.1696.

HPLC: Enantiomeric excess was determined by HPLC with a Chiralpak IA column (hexanes: 2-propanol = 99.5:0.5, 0.8 mL/min, 330 nm, 94.6:5.4 *er*); major enantiomer t_r = 10.2 min, minor enantiomer t_r = 21.0 min.

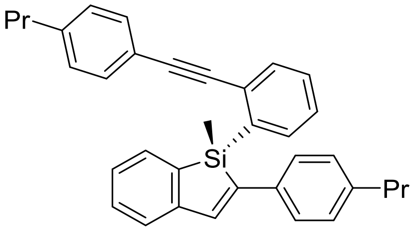

(*S*)-1-methyl-2-(4-propylphenyl)-1-(2-((4-propylphenyl)ethynyl)phenyl)-1*H*-benzo[*b*]silole (2f)

The mobile phase for flash chromatography: hexane. Yellow oil. (64.9 mg, 71%).

Optical rotation: $[\alpha]_D^{25}$ = +232.4 (c = 0.63, $CHCl_3$).

^{1}H NMR (400 MHz, $CDCl_3$) δ 7.83 (d, *J* = 7.0 Hz, 1H), 7.60 (s, 1H), 7.50 (d, *J* = 7.6 Hz, 1H), 7.42 (d, *J* = 8.1 Hz, 2H), 7.37 (t, *J* = 5.9 Hz, 3H), 7.27 – 7.17 (m, 3H), 7.13 – 7.01 (m, 6H), 2.57 – 2.46 (m, 4H), 1.57 (dt, *J* = 14.6, 7.3 Hz, 4H), 0.94 – 0.82 (m, 9H).

^{13}C NMR (100 MHz, $CDCl_3$) δ 149.4, 143.5, 143.4, 142.1, 141.6, 137.7, 137.6, 136.3, 135.8, 132.9, 132.7, 131.6, 130.3, 129.8, 129.6, 129.0, 128.8, 127.7, 127.1, 127.0, 124.5, 120.6, 92.6, 90.9, 38.2, 38.0, 24.6, 24.5, 14.1, 13.9, -3.4.

HRMS (ESI-TOF) m/z: $[M+Na]^+$ Calcd for $C_{35}H_{34}NaSi$, 505.2322; found 505.2313.

HPLC: Enantiomeric excess was determined by HPLC with a Chiralpak IA column (hexanes: 2-propanol = 99.5:0.5, 0.8 mL/min, 330 nm, 93.6:6.5 *er*); major enantiomer t_r = 7.5 min, minor enantiomer t_r = 13.1 min.

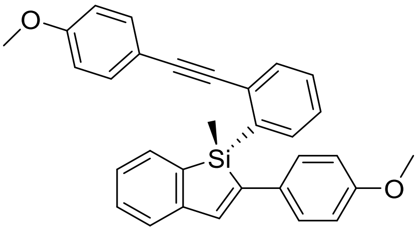

(*S*)-2-(4-methoxyphenyl)-1-(2-((4-methoxyphenyl)ethynyl)phenyl)-1-methyl-1*H*-benzo[*b*]silole (2g)

The mobile phase for flash chromatography: hexane/ethyl acetate = 100:1. Yellow solid. (52.3 mg, 57%).

Optical rotation: $[\alpha]_D^{25}$ = +321.5 (c = 1.67, $CHCl_3$). mp 64.9 °C.

^{1}H NMR (400 MHz, $CDCl_3$) δ 7.88 (d, *J* = 6.9 Hz, 1H), 7.61 – 7.41 (m, 8H), 7.33 – 7.24 (m, 2H), 7.17 – 7.09 (m, 2H), 6.89 (t, *J* = 8.7 Hz, 4H), 3.83 (s, 3H), 3.80 (s, 3H), 0.96 (s, 3H).

^{13}C NMR (100 MHz, $CDCl_3$) δ 159.9, 159.1, 149.6, 143.0, 140.3, 137.4, 137.4, 135.8, 135.7, 133.1, 132.9, 132.8, 132.5, 132.5, 131.6, 130.3, 129.8, 129.7, 128.9, 128., 127.6, 127.2, 126.8, 124.3, 92.4, 90.2, 55.5, 55.4, -3.4.

HRMS (ESI-TOF) m/z: $[M+Na]^+$ Calcd for $C_{31}H_{26}NaO_2Si$, 481.1584; found 481.1594.

HPLC: Enantiomeric excess was determined by HPLC with a Chiralpak IA column (hexanes: 2-propanol = 99.5:0.5, 0.8 mL/min, 330 nm, 90.2:9.8 *er*); major enantiomer t_r = 22.2 min, minor enantiomer t_r = 45.0 min.

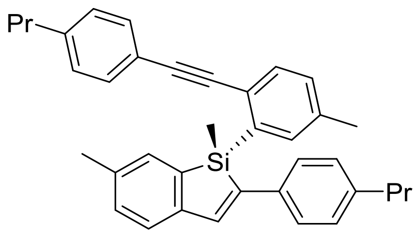

(*R*)-1,6-dimethyl-1-(5-methyl-2-((4-propylphenyl)ethynyl)phenyl)-2-(4-propylphenyl)-1*H*-benzo[*b*]silole (2h)

The mobile phase for flash chromatography: hexane. Yellow oil. (82.7 mg, 81%).

Optical rotation: $[\alpha]_D^{25}$ = +221.5 (c = 0.63, $CHCl_3$).

^{1}H NMR (400 MHz, CDCl$_3$) δ 7.65 (s, 1H), 7.57 (s, 1H), 7.43 – 7.34 (m, 5H), 7.17 (s, 1H), 7.11 – 6.99 (m, 7H), 2.67 – 2.41 (m, 4H), 2.15 (s, 3H), 2.09 (s, 3H), 1.63 – 1.50 (m, 4H), 0.87 (dt, J = 7.3, 3.0 Hz, 9H).

^{13}C NMR (100 MHz, CDCl$_3$) δ 146.8, 143.3, 142.4, 141.7, 141.4, 137.9, 137.6, 137.5, 136.6, 136.5, 136.5, 134.0, 132.7, 131.5, 130.8, 130.6, 128.9, 128.7, 127.0, 126.5, 124.3, 120.8, 91.9, 91.1, 38.1, 37.9, 24.6, 24.5, 21.6, 21.5, 14.0, 13.9, -3.4.

HRMS (ESI-TOF) m/z: [M+Na]$^+$ Calcd for $C_{37}H_{38}NaSi$, 533.2635; found 533.2623.

HPLC: Enantiomeric excess was determined by HPLC with a Phenomenex column (hexanes: 2-propanol = 99.5:0.5, 0.6 mL/min, 330 nm, 93.9:6.1 *er*); major enantiomer t_r = 8.6 min, minor enantiomer t_r = 9.6 min.

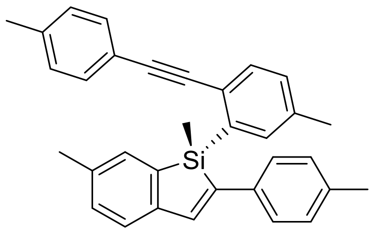

(*R*)-1,6-dimethyl-1-(5-methyl-2-(*p*-tolylethynyl)phenyl)-2-(*p*-tolyl)-1*H*-benzo[*b*]silole (2i)

The mobile phase for flash chromatography: hexane. Yellow oil. (54.5 mg, 60%).

Optical rotation: $[\alpha]_D^{25}$ = +231.1 (c = 1.33, CHCl$_3$).

^{1}H NMR (400 MHz, CDCl$_3$) δ 7.65 (s, 1H), 7.56 (s, 1H), 7.41 – 7.33 (m, 5H), 7.18 (s, 1H), 7.14 – 6.99 (m, 8H), 2.29 (s, 3H), 2.26 (s, 3H), 2.15 (s, 3H), 2.09 (s, 3H), 0.84 (s, 3H).

^{13}C NMR (100 MHz, CDCl$_3$) δ 146.8, 142.3, 141.4, 138.5, 137.9, 137.7, 137.5, 136.9, 136.5, 136.4, 136.3, 134.0, 132.7, 131.5, 130.8, 130.6, 129.5, 129.3, 127.0, 126.5, 124.3, 120.6, 91.8, 91.0, 21.7, 21.6, 21.5, 21.4, -3.5.

HRMS (ESI-TOF) m/z: [M+Na]$^+$ Calcd for $C_{33}H_{30}NaSi$, 477.2009; found 477.2019.

HPLC: Enantiomeric excess was determined by HPLC with a Phenomenex column (hexanes: 2-propanol = 99.5:0.5, 0.6 mL/min, 330 nm, 95.7:4.3 *er*); major enantiomer t_r = 9.7 min, minor enantiomer t_r = 11.2 min.

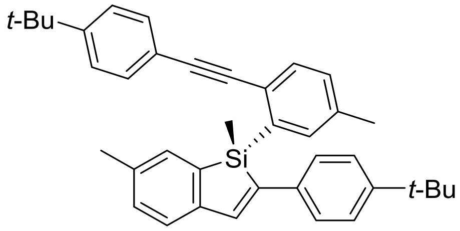

(*R*)-2-(4-(*tert*-butyl)phenyl)-1-(2-((4-(*tert*-butyl)phenyl)ethynyl)-5-methylphenyl)-1,6-dimethyl-1*H*-benzo[*b*]silole (2j)

The mobile phase for flash chromatography: hexane. Yellow oil. (54.8 mg, 51%).

Optical rotation: $[\alpha]_D^{25}$ = +211.9 (c = 0.67, $CHCl_3$).

^{1}H NMR (400 MHz, $CDCl_3$) δ 7.65 (s, 1H), 7.59 (s, 1H), 7.46 – 7.37 (m, 4H), 7.29 (dd, *J* = 15.2, 8.3 Hz, 4H), 7.19 (s, 1H), 7.17 (s, 1H), 7.07 (dt, *J* = 23.2, 7.5 Hz, 3H), 2.17 (s, 3H), 2.11 (s, 3H), 1.26 (s, 9H), 1.24 (s, 9H), 0.85 (s, 3H).

^{13}C NMR (100 MHz, $CDCl_3$) δ 151.6, 150.1, 146.8, 142.4, 141.6, 138.0, 137.7, 137.5, 136.6, 136.4, 136.3, 133.9, 132.7, 131.3, 130.8, 130.6, 126.8, 126.5, 125.7, 125.5, 124.3, 120.6, 91.9, 91.1, 35.9, 34.7, 31.5, 31.4, 21.6, 21.5, -3.4.

HRMS (ESI-TOF) m/z: $[M+Na]^+$ Calcd for $C_{39}H_{42}NaSi$, 561.2948; found 561.2934.

HPLC: Enantiomeric excess was determined by HPLC with a Chiralpak IA column (hexanes: 2-propanol = 99.5:0.5, 0.6 mL/min, 330 nm, 92.4:7.6 *er*); major enantiomer t_r = 7.8 min, minor enantiomer t_r = 8.2 min.

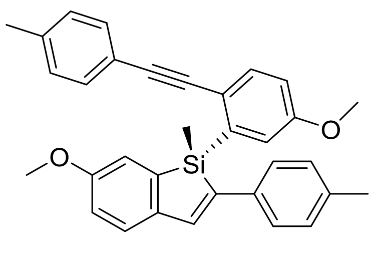

(*R*)-6-methoxy-1-(5-methoxy-2-(*p*-tolylethynyl)phenyl)-1-methyl-2-(*p*-tolyl)-1*H*-benzo[*b*]silole (2k)

The mobile phase for flash chromatography: hexane/ethyl acetate = 100:1. Yellow oil. (38.9 mg, 40%).

Optical rotation: $[\alpha]_D^{25}$ = +158.2 (c = 0.67, $CHCl_3$).

^{1}H NMR (400 MHz, CDCl$_3$) δ 7.56 (s, 1H), 7.49 (d, *J* = 2.6 Hz, 1H), 7.45 (d, *J* = 8.5 Hz, 1H), 7.39 (d, *J* = 1.7 Hz, 2H), 7.37 (d, *J* = 1.7 Hz, 2H), 7.14 – 7.09 (m, 2H), 7.09 – 7.04 (m, 3H), 6.89 (d, *J* = 2.7 Hz, 1H), 6.75 (ddd, *J* = 15.4, 8.4, 2.7 Hz, 2H), 3.49 (s, 3H), 3.46 (s, 3H), 2.30 (s, 3H), 2.27 (s, 3H), 0.91 (s, 3H).

^{13}C NMR (100 MHz, CDCl$_3$) δ 159.1, 158.8, 142.2, 141.5, 140.4, 139.6, 139.6, 138.4, 136.7, 136.4, 134.2, 131.4, 129.6, 129.4, 126.8, 125.4, 121.5, 121.0, 120.6, 118.4, 116.0, 115.7, 91.1, 91.0, 55.2, 55.1, 21.7, 21.3, -3.4.

HRMS (ESI-TOF) m/z: [M+Na]$^+$ Calcd for $C_{33}H_{30}NaO_2Si$, 509.1907; found 509.1905.

HPLC: Enantiomeric excess was determined by HPLC with a Phenomenex column (hexanes: 2-propanol = 99.5:0.5, 0.8 mL/min, 330 nm, 94.0:6.0 *er*); major enantiomer t_r = 19.7 min, minor enantiomer t_r = 25.4 min.

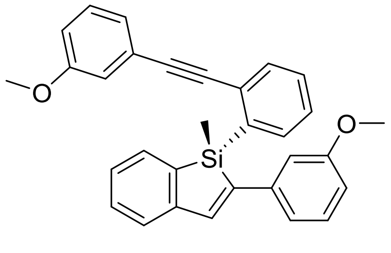

(*S*)-2-(3-methoxyphenyl)-1-(2-((3-methoxyphenyl)ethynyl)phenyl)-1-methyl-1*H*-benzo[*b*]silole (2l)

The mobile phase for flash chromatography: hexane/ethyl acetate = 100:1. Yellow oil. (36.7 mg, 40%).

Optical rotation: $[\alpha]_D^{25}$ = +149.2 (c = 1.33, $CHCl_3$).

^{1}H NMR (400 MHz, CDCl$_3$) δ 7.93 (d, J = 7.0 Hz, 1H), 7.81 – 7.73 (m, 2H), 7.51 (d, J = 7.7 Hz, 1H), 7.42 (dd, J = 7.6, 1.6 Hz, 1H), 7.26 (ddd, J = 9.3, 8.0, 2.4 Hz, 2H), 7.20 – 7.13 (m, 4H), 7.06 – 6.97 (m, 2H), 6.95 – 6.82 (m, 3H), 6.76 (d, J = 7.7 Hz, 1H), 3.80 (s, 3H), 3.38 (s, 3H), 0.87 (s, 3H).

^{13}C NMR (100 MHz, CDCl$_3$) δ 160.2, 156.4, 148.1, 142.8, 140.5, 139.4, 137.3, 134.7, 133.6, 132.8, 132.7, 129.9, 129.6, 128.8, 128.8, 128.5, 127.8, 127.3, 127.1, 126.0, 124.2, 121.0, 120.6, 112.9, 111.0, 110.8, 95.6, 88.3, 55.7, 54.3, -3.3.

HRMS (ESI-TOF) m/z: [M+Na]$^+$ Calcd for $C_{31}H_{26}NaO_2Si$, 481.1594; found 481.1584.

HPLC: Enantiomeric excess was determined by HPLC with a Phenomenex column (hexanes: 2-propanol = 99.6:0.4, 0.8 mL/min, 330 nm, 95.5:4.5 *er*); major enantiomer t_r = 26.2 min, minor enantiomer t_r = 28.7 min.

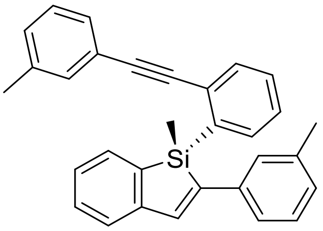

(*S*)-1-methyl-2-(*m*-tolyl)-1-(2-(*m*-tolylethynyl)phenyl)-1*H*-benzo[*b*]silole (2m)
The mobile phase for flash chromatography: hexane. Yellow oil. (46.9 mg, 55%).

Optical rotation: $[\alpha]_D^{25}$ = +272.7 (c = 0.99, CHCl$_3$).

^{1}H NMR (400 MHz, CDCl$_3$) δ 7.90 (d, J = 7.0 Hz, 1H), 7.70 (s, 1H), 7.58 (d, J = 7.6 Hz, 1H), 7.47 – 7.41 (m, 2H), 7.37 – 7.32 (m, 4H), 7.30 – 7.22 (m, 4H), 7.19 – 7.12 (m, 3H), 7.06 (d, J = 7.5 Hz, 1H), 2.36 (s, 3H), 2.34 (s, 3H), 0.98 (s, 3H).

^{13}C NMR (100 MHz, CDCl$_3$) δ 149.3, 143.7, 142.4, 138.9, 138.4, 138.3, 137.8, 137.5, 135.7, 132.9, 132.8, 132.2, 130.3, 129.8, 129.5, 129.5, 128.8, 128.5, 128.2, 127.9, 127.5, 127.1, 124.7, 124.6, 123.2, 92.5, 91.1, 21.7, 21.4, -3.4.

HRMS (ESI-TOF) m/z: [M+Na]$^+$ Calcd for $C_{31}H_{26}NaSi$, 449.1696; found 449.1685.

HPLC: Enantiomeric excess was determined by HPLC with a Chiralpak IA column (hexanes: 2-propanol = 99.5:0.5, 0.6 mL/min, 330 nm, 92.0:8.0 *er*); major enantiomer t_r = 26.0 min, minor enantiomer t_r = 27.8 min.

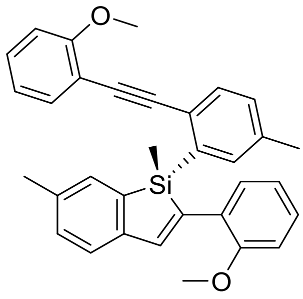

(*R*)-2-(2-methoxyphenyl)-1-(2-((2-methoxyphenyl)ethynyl)-5-methylphenyl)-1,6-dimethyl-1*H*-benzo[*b*]silole (2n)

The mobile phase for flash chromatography: hexane/ethyl acetate = 100:1. Yellow oil. (58.3 mg, 60%).

Optical rotation: $[\alpha]_D^{25}$ = +357.1 (c = 1.33, $CHCl_3$).

^{1}H NMR (400 MHz, $CDCl_3$) δ 7.82 – 7.65 (m, 3H), 7.42 (d, *J* = 8.1 Hz, 2H), 7.24 (td, *J* = 8.3, 1.7 Hz, 1H), 7.19 – 7.10 (m, 1H), 7.07 (d, *J* = 7.6 Hz, 2H), 6.98 (ddd, *J* = 6.7, 4.3, 1.1 Hz, 2H), 6.93 – 6.79 (m, 3H), 6.79 – 6.68 (m, 1H), 3.79 (s, 3H), 3.38 (s, 3H), 2.15 (s, 3H), 2.05 (s, 3H), 0.84 (s, 3H).

^{13}C NMR (100 MHz, $CDCl_3$) δ 160.1, 156.3, 145.6, 142.7, 140.6, 139.3, 137.1, 136.6, 136.4, 135.4, 133.7, 133.5, 132.7, 130.2, 129.7, 129.6, 128.2, 128.1, 126.1, 125.7, 124.0, 120.9, 120.6, 113.1, 110.9, 110.7, 95.9, 87.6, 55.7, 54.3, 21.6, -3.2.

HRMS (ESI-TOF) m/z: $[M+Na]^+$ Calcd for $C_{37}H_{38}NaSi$, 509.1907; found 509.1899.

HPLC: Enantiomeric excess was determined by HPLC with a Phenomenex column (hexanes: 2-propanol = 99.5:0.5, 0.8 mL/min, 330 nm, 92.2:5.8 *er*); major enantiomer t_r = 17.3 min, minor enantiomer t_r = 20.0 min.

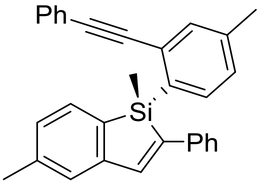

(*S*)-1,5-dimethyl-1-(4-methyl-2-(phenylethynyl)phenyl)-2-phenyl-1*H*-benzo[*b*]silole (2o)

The mobile phase for flash chromatography: hexane. Yellow oil. (41.7 mg, 49%).

Optical rotation: $[\alpha]_D^{25}$ = +201.6 (c = 0.65, $CHCl_3$).

^{1}H NMR (400 MHz, $CDCl_3$) δ 7.76 (d, *J* = 7.2 Hz, 1H), 7.66 (s, 1H), 7.57 (d, *J* = 7.3 Hz, 2H), 7.50 (dd, *J* = 6.6, 3.0 Hz, 2H), 7.43 (s, 1H), 7.36 (dd, *J* = 6.3, 3.4 Hz, 4H), 7.34 – 7.30 (m, 2H), 7.23 (s, 1H), 7.11 (s, 1H), 6.99 (t, *J* = 7.3 Hz, 2H), 2.31 (d, *J* = 4.3 Hz, 6H), 0.94 (s, 3H).

^{13}C NMR (100 MHz, $CDCl_3$) δ 149.6, 144.0, 142.4, 140.3, 139.9, 139.1, 135.8, 134.4, 134.1, 133.5, 132.8, 131.6, 129.3, 128.9, 128.9, 128.5, 128.5, 127.9, 127.2, 127.1, 125.7, 123.5, 91.9, 91.5, 21.7, 21.3, -3.3.

HRMS (ESI-TOF) m/z: [M+H]$^+$ Calcd for $C_{31}H_{27}Si$, 427.1877; found 427.1884.

HPLC: Enantiomeric excess was determined by HPLC with a Chiralpak IA column (hexanes: 2-propanol = 99.5:0.5, 0.5 mL/min, 330 nm, 97.3:2.7 *er*); major enantiomer t_r = 18.4 min, minor enantiomer t_r = 21.9 min.

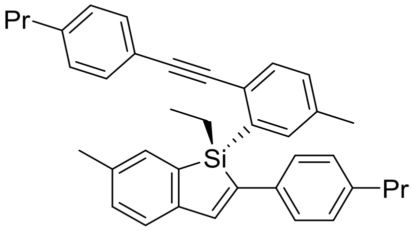

(*R*)-1-ethyl-6-methyl-1-(5-methyl-2-((4-propylphenyl)ethynyl)phenyl)-2-(4-propylphenyl)-1*H*-benzo[*b*]silole (2p)

The mobile phase for flash chromatography: hexane. Yellow oil. (42.0 mg, 41%).

Optical rotation: $[\alpha]_D^{25}$ = +97.8 (c = 0.67, $CHCl_3$).

^{1}H NMR (400 MHz, CDCl$_3$) δ 7.59 (d, J = 14.6 Hz, 1H), 7.40 (d, J = 8.1 Hz, 2H), 7.34 (d, J = 8.1 Hz, 2H), 7.21 – 7.17 (m, 1H), 7.15 (s, 1H), 7.06 (ddd, J = 15.3, 10.1, 4.2 Hz, 6H), 6.97 – 6.91 (m, 1H), 6.88 – 6.81 (m, 1H), 2.57 – 2.45 (m, 4H), 2.16 (s, 3H), 2.10 (s, 2H), 1.56 (dd, J = 15.9, 8.0 Hz, 4H), 1.32 (dd, J = 15.1, 7.8 Hz, 2H), 0.90 – 0.83 (m, 9H).

^{13}C NMR (100 MHz, CDCl$_3$) δ 147.4, 143.2, 142.2, 141.6, 141.5, 137.6, 136.9, 136.9, 136.8, 136.7, 136.4, 134.2, 132.8, 131.5, 130.7, 130.6, 128.9, 128.8, 128.7, 128.6, 127.0, 126.6, 126.6, 124.2, 120.9, 91.6, 91.1, 38.1, 37.9, 24.6, 24.5, 21.6, 21.5, 14.0, 13.9, 7.7, 5.0, 1.2.

HRMS (ESI-TOF) m/z: [M+Na]$^+$ Calcd for $C_{38}H_{40}NaSi$, 547.2791; found 547.2781.

HPLC: Enantiomeric excess was determined by HPLC with a Chiralcel AD-H column (hexanes: 2-propanol = 99.5:0.5, 0.6 mL/min, 330 nm, 97.9:2.1 *er*); major enantiomer t_r = 14.8 min, minor enantiomer t_r = 20.8 min.

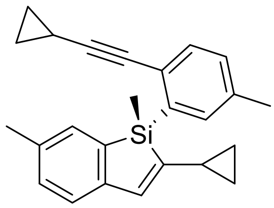

(R)-2-cyclopropyl-1-(2-(cyclopropylethynyl)-5-methylphenyl)-1,6-dimethyl-1*H*-benzo[*b*]silole (2q)

The mobile phase for flash chromatography: hexane. Yellow oil. (31.9 mg, 45%).

Optical rotation: $[\alpha]_D^{25}$ = +162.2 (c = 0.33, CHCl$_3$).

^{1}H NMR (400 MHz, CDCl$_3$) δ 7.42 (s, 1H), 7.28 (dd, J = 1.2, 0.5 Hz, 1H), 7.23 (d, J = 7.8 Hz, 1H), 7.16 (s, 1H), 7.03 – 6.97 (m, 2H), 6.94 (d, J = 7.6 Hz, 1H), 6.84 (s, 1H), 2.22 (s, 3H), 2.19 (s, 3H), 1.79 – 1.69 (m, 1H), 1.42 – 1.30 (m, 1H), 0.81 – 0.69 (m, 6H), 0.61 (s, 3H), 0.59 – 0.43 (m, 2H).

^{13}C NMR (100 MHz, CDCl$_3$) δ 149.2, 147.0, 140.3, 137.2, 137.1, 136.8, 135.9, 135.3, 133.5, 132.6, 130.5, 130.4, 127.0, 122.8, 95.3, 21.6, 21.5, 14.4, 8.6, 8.2, 8.1, 1.2, 0.6, -4.0.

HRMS (ESI-TOF) m/z: [M+Na]$^+$ Calcd for $C_{25}H_{26}NaSi$, 377.1696; found 377.1689.

HPLC: Enantiomeric excess was determined by HPLC with a Phenomenex column (hexanes: 2-propanol = 99.5:0.5, 0.6 mL/min, 330 nm, 92.0:8.0 *er*); major enantiomer t_r = 7.8 min, minor enantiomer t_r = 8.4 min.

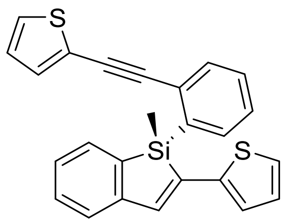

(*R*)-1-methyl-2-(thiophen-2-yl)-1-(2-(thiophen-2-ylethynyl)phenyl)-1*H*-benzo[*b*]silole (2r)

The mobile phase for flash chromatography: hexane. colorless solid. (61.7 mg, 75%).

Optical rotation: $[\alpha]_D^{25}$ = +66.5 (c = 0.33, CHCl$_3$). mp 143.5-146.4 ^{o}C.

^{1}H NMR (400 MHz, CDCl$_3$) δ 7.77 (d, *J* = 7.0 Hz, 1H), 7.47 (d, *J* = 7.6 Hz, 1H), 7.42 (d, *J* = 5.8 Hz, 2H), 7.26 – 7.14 (m, 5H), 7.12 – 7.09 (m, 2H), 7.04 (d, *J* = 0.7 Hz, 1H), 6.93 (dd, *J* = 5.0, 3.7 Hz, 1H), 6.90 – 6.83 (m, 2H), 0.84 (s, 3H).

^{13}C NMR (100 MHz, CDCl$_3$) δ 149.1, 144.1, 141.3, 137.0, 136.8, 135.7, 133.0, 132.4, 132.2, 130.5, 130.0, 129.0, 128.1, 127.9, 127.8, 127.4, 127.1, 126.7, 124.6, 124.5, 123.3, 94.9, 85.9, -3.8.

HRMS (ESI-TOF) m/z: [M+H]$^+$ Calcd for $C_{25}H_{19}S_2Si$, 411.0692; found 411.0678.

HPLC: Enantiomeric excess was determined by HPLC with a Phenomenex column (hexanes: 2-propanol = 99.5:0.5, 0.6 mL/min, 330 nm, 93.6:6.4 *er*); major enantiomer t_r = 17.9 min, minor enantiomer t_r = 26.1 min.

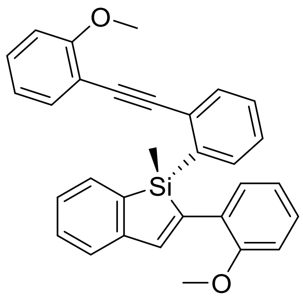

(*R*)-2-(2-methoxyphenyl)-1-(2-((2-methoxyphenyl)ethynyl)phenyl)-1-methyl-1*H*-benzo[*b*]silole (2s)

The mobile phase for flash chromatography: hexane/ethyl acetate = 100:1. Yellow oil. (20.7 mg, 30%).

Optical rotation: $[\alpha]_D^{25}$ = +117.9 (c = 0.67, $CHCl_3$).

^{1}H NMR (400 MHz, $CDCl_3$) δ 7.80 (d, J = 7.0 Hz, 1H), 7.60 (s, 1H), 7.51 (d, J = 7.4 Hz, 1H), 7.42 – 7.37 (m, 1H), 7.22 (dddd, J = 20.7, 10.7, 6.3, 2.1 Hz, 5H), 7.14 – 7.00 (m, 5H), 6.96 – 6.92 (m, 1H), 6.86 – 6.80 (m, 1H), 6.73 (dd, J = 8.1, 2.0 Hz, 1H), 3.73 (s, 3H), 3.70 (s, 3H), 0.89 (s, 3H).

^{13}C NMR (100 MHz, $CDCl_3$) δ 160.0, 159.5, 149.1, 143.5, 142.9, 140.4, 137.7, 137.4, 135.8, 132.9, 132.8, 130.3, 129.9, 129.8, 129.6, 129.3, 128.0, 127.3, 124.7, 124.3, 124.2, 120.1, 116.5, 115.1, 113.0, 112.2, 92.3, 91.1, 55.4, 55.3, -3.5.

HRMS (ESI-TOF) m/z: $[M+Na]^+$ Calcd for $C_{31}H_{26}NaO_2Si$, 481.1594; found 481.1609.

HPLC: Enantiomeric excess was determined by HPLC with a Phenomenex column (hexanes: 2-propanol = 99.3:0.7, 0.7 mL/min, 330 nm, 97.2:2.8 *er*); major enantiomer t_r = 21.3 min, minor enantiomer t_r = 24.2 min.

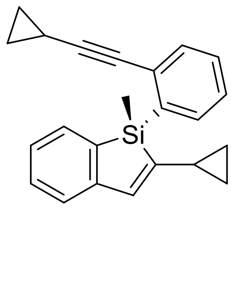

(*R*)-2-cyclopropyl-1-(2-(cyclopropylethynyl)phenyl)-1-methyl-1*H*-benzo[*b*]silole (2t)

The mobile phase for flash chromatography: hexane. Yellow oil. (44.4 mg, 68%).

Optical rotation: $[\alpha]_D^{25}$ = +77.8 (c = 1.33, $CHCl_3$).

^{1}H NMR (400 MHz, $CDCl_3$) δ 7.56 (d, *J* = 6.9 Hz, 1H), 7.46 (dd, *J* = 7.4, 0.8 Hz, 1H), 7.33 (d, *J* = 7.6 Hz, 1H), 7.19 (tdd, *J* = 8.1, 3.7, 1.6 Hz, 2H), 7.10 (td, *J* = 7.4, 1.1 Hz, 1H), 7.03 (t, *J* = 8.0 Hz, 2H), 6.86 (s, 1H), 1.82 – 1.69 (m, 1H), 1.35 (tt, *J* = 8.2, 5.1 Hz, 1H), 0.80 – 0.69 (m, 6H), 0.64 – 0.61 (m, 3H), 0.59 (dd, *J* = 8.9, 4.0 Hz, 1H), 0.50 – 0.44 (m, 1H).

^{13}C NMR (100 MHz, $CDCl_3$) δ 149.6, 148.7, 139.4, 136.1, 135.8, 134.2, 131.7, 131.5, 129.2, 129.1, 128.7, 126.0, 124.9, 122.1, 95.3, 13.5, 7.9, 7.3, 7.2, -0.5, -5.2.

HRMS (ESI-TOF) m/z: $[M+Na]^+$ Calcd for $C_{23}H_{22}NaSi$, 349.1383; found 349.1369.

HPLC: Enantiomeric excess was determined by HPLC with a Phenomenex column (hexanes: 2-propanol = 99.5:0.5, 0.6 mL/min, 330 nm, 93.2:6.8 *er*); major enantiomer t_r = 10.9 min, minor enantiomer t_r = 15.8 min.
